# Components and Delivery Formats of Cognitive Behavioral Therapy for Chronic Insomnia in Adults: A Systematic Review and Component Network Meta-Analysis

**DOI:** 10.1001/jamapsychiatry.2023.5060

**Published:** 2024-01-17

**Authors:** Yuki Furukawa, Masatsugu Sakata, Ryuichiro Yamamoto, Shun Nakajima, Shino Kikuchi, Mari Inoue, Masami Ito, Hiroku Noma, Hikari Nishimura Takashina, Satoshi Funada, Edoardo G. Ostinelli, Toshi A. Furukawa, Orestis Efthimiou, Michael Perlis

**Affiliations:** 1 Department of Neuropsychiatry, University of Tokyo Hospital, Tokyo, Japan; 2 Department of Health Promotion and Human Behavior, Kyoto University Graduate School of Medicine/School of Public Health, Kyoto, Japan; 3 College of Sociology, Department of Psychology and Humanities, Edogawa University, Nagareyama, Chiba, Japan; 4 National Center for Cognitive Behavior Therapy and Research, National Center of Neurology and Psychiatry, Tokyo, Japan; 5 International Institute for Integrative Sleep Medicine, University of Tsukuba, Ibaraki, Japan; 6 Core Laboratory, Center for Psycho-oncology and Palliative Care, Nagoya City University Graduate School of Medical Sciences, Nagoya, Aichi, Japan; 7 Graduate School of Medical Science, Kitasato University, Kanagawa, Japan; 8 Research Center for Child Mental Development, Chiba University, Chiba, Japan; 9 Department of Preventive Medicine and Public Health, Keio University School of Medicine, Tokyo, Japan; 10 Department of Psychiatry, University of Oxford, Oxford, United Kingdom; 11 Oxford Precision Psychiatry Lab, National Institute for Health and Care Research, Oxford Health Biomedical Research Centre, Oxford, United Kingdom; 12 Oxford Health National Health Service Foundation Trust, Warneford Hospital, Oxford, United Kingdom; 13 Department of Clinical Epidemiology, Kyoto University Graduate School of Medicine/School of Public Health, Kyoto, Japan; 14 Institute of Social and Preventive Medicine, University of Bern, Bern, Switzerland; 15 Institute of Primary Health Care, University of Bern, Bern, Switzerland; 16 Behavioral Sleep Medicine Program, Department of Psychiatry, School of Nursing, University of Pennsylvania, Philadelphia

## Abstract

**Importance:**

Chronic insomnia disorder is highly prevalent, disabling, and costly. Cognitive behavioral therapy for insomnia (CBT-I), comprising various educational, cognitive, and behavioral strategies delivered in various formats, is the recommended first-line treatment, but the effect of each component and delivery method remains unclear.

**Objective:**

To examine the association of each component and delivery format of CBT-I with outcomes.

**Data Sources:**

PubMed, Cochrane Central Register of Controlled Trials, PsycInfo, and International Clinical Trials Registry Platform from database inception to July 21, 2023.

**Study Selection:**

Published randomized clinical trials comparing any form of CBT-I against another or a control condition for chronic insomnia disorder in adults aged 18 years and older. Insomnia both with and without comorbidities was included. Concomitant treatments were allowed if equally distributed among arms.

**Data Extraction and Synthesis:**

Two independent reviewers identified components, extracted data, and assessed trial quality. Random-effects component network meta-analyses were performed.

**Main Outcomes and Measures:**

The primary outcome was treatment efficacy (remission defined as reaching a satisfactory state) posttreatment. Secondary outcomes included all-cause dropout, self-reported sleep continuity, and long-term remission.

**Results:**

A total of 241 trials were identified including 31 452 participants (mean [SD] age, 45.4 [16.6] years; 21 048 of 31 452 [67%] women). Results suggested that critical components of CBT-I are cognitive restructuring (remission incremental odds ratio [iOR], 1.68; 95% CI, 1.28-2.20) third-wave components (iOR, 1.49; 95% CI, 1.10-2.03), sleep restriction (iOR, 1.49; 95% CI, 1.04-2.13), and stimulus control (iOR, 1.43; 95% CI, 1.00-2.05). Sleep hygiene education was not essential (iOR, 1.01; 95% CI, 0.77-1.32), and relaxation procedures were found to be potentially counterproductive(iOR, 0.81; 95% CI, 0.64-1.02). In-person therapist-led programs were most beneficial (iOR, 1.83; 95% CI, 1.19-2.81). Cognitive restructuring, third-wave components, and in-person delivery were mainly associated with improved subjective sleep quality. Sleep restriction was associated with improved subjective sleep quality, sleep efficiency, and wake after sleep onset, and stimulus control with improved subjective sleep quality, sleep efficiency, and sleep latency. The most efficacious combination—consisting of cognitive restructuring, third wave, sleep restriction, and stimulus control in the in-person format—compared with in-person psychoeducation, was associated with an increase in the remission rate by a risk difference of 0.33 (95% CI, 0.23-0.43) and a number needed to treat of 3.0 (95% CI, 2.3-4.3), given the median observed control event rate of 0.14.

**Conclusions and Relevance:**

The findings suggest that beneficial CBT-I packages may include cognitive restructuring, third-wave components, sleep restriction, stimulus control, and in-person delivery but not relaxation. However, potential undetected interactions could undermine the conclusions. Further large-scale, well-designed trials are warranted to confirm the contribution of different treatment components in CBT-I.

## Key Points

**Question:**

What is the association of each component and delivery format of cognitive behavioral therapy for chronic insomnia with outcomes?

**Findings:**

This systematic review and component network meta-analysis including 241 trials found that cognitive restructuring, third-wave components, sleep restriction, stimulus control, and in-person format may be beneficial. Cognitive restructuring, third-wave components and in-person delivery were mainly associated with improved subjective sleep quality, while sleep restriction and stimulus control were associated both with improved sleep quality and self-reported sleep continuity.

**Meaning:**

The findings suggest that beneficial cognitive behavioral therapy for insomnia may include cognitive restructuring, third-wave components, sleep restriction, stimulus control, and in-person format.

## Teaser

This systematic review and component network meta-analysis evaluates associations between different components and delivery formats of cognitive behavior therapy for insomnia.

## Introduction

Chronic insomnia disorder is characterized by sleep continuity disturbance associated with considerable distress or impairments of daytime functioning. It occurs in 4% to 22% of the population in the US and is highly disabling.^1^ It is associated with both physical and psychiatric comorbidities^2^ and has a large economic burden.^3^ Cognitive behavioral therapy for insomnia (CBT-I) is the first-line treatment for chronic insomnia, based on its efficacy and safety profile.^4,5^

CBT-I is a structured, nonpharmacological treatment that uses several educational, cognitive, or behavioral strategies. Meta-analyses have shown that CBT-I as a package is associated with improved sleep quality in chronic insomnia with or without comorbidities.^2,6^ However, the specific roles of each individual component remain unclear, and it is unknown whether all components of CBT-I are required to ensure its benefit. Several randomized clinical trials have tried to examine individual component effects but combining such studies in pairwise meta-analyses can be misleading because they compared different components to different control conditions. Identifying the effect of each intervention is crucial, as it could lead to an intervention that maximizes treatment efficacy, minimizes treatment burden, and increases scalability.

Component network meta-analysis (cNMA) is an extension of standard NMA^7^ that can be used to disentangle the associations of different components included in multicomponent interventions with outcomes.^8,9^ In this study, we explored the association of each treatment component and delivery format of CBT-I for chronic insomnia disorder in adults with clinical outcomes using cNMA.

## Methods

The protocol was prospectively registered in PROSPERO (CRD42022324233) and can be found in eMethods 1 in Supplement 1. We report the results following the Preferred Reporting Items for Systematic reviews and Meta-Analyses (PRISMA) guideline extension for NMA.^10^

### Data Sources

#### Criteria for Considering Studies for This Review

We included all randomized clinical trials that compared any form of CBT-I against another form of CBT-I or a control condition in the treatment of adults with chronic insomnia. We included trials on patients of both sexes aged 18 years or older with chronic insomnia either diagnosed according to formal diagnostic criteria or by elevated scores on self-reported measures with satisfactory reliability and validity. We included patients with psychiatric or physical comorbidities. We regarded CBT-I broadly as a psychotherapy that involved 1 or more of the following cognitive or behavioral components: cognitive restructuring, constructive worry, third-wave components (mindfulness and acceptance and commitment therapy), sleep restriction, stimulus control, paradoxical intention, and relaxation (Table 1). Coadministration of pharmacological or other psychological therapies was allowed if equally distributed among the arms. We included various delivery formats, such as in-person contact and individual or group format. We disaggregated these delivery methods as components (Table 1). Control conditions included waiting list, no treatment, psychoeducation (sleep hygiene education), attention or psychological placebo control, and treatment as usual. Where multiple arms were reported in a single trial, we included only relevant arms that could be described with the components listed in Table 1. The definitions of the components were based on previous studies^11,12^ and content expert consensus among coauthors (M.S., R.Y., S.N., T.A.F., M.P.).

**Table 1.** List of Included Components, Delivery Methods, and Their Definitions

Intervention	Description
Educational components	
Sleep hygiene education	General explanation about sleep (eg, sleep biology, characteristics of healthy sleep, and changes in sleep patterns with aging) and general recommendations about lifestyle (eg, diet, exercise, and substance use) and environmental factors (eg, light, noise, and temperature) to improve sleep. This may include some elements of other components, such as stimulus control, but these should not be the predominant part of the intervention.
Sleep diary	Self-monitoring of important daily sleep-related information using a sleep diary.
Cognitive components	
Cognitive restructuring	Skills to identify, challenge, and change unhelpful beliefs about sleep that may disturb sleep. Sometimes simply called cognitive therapy. This may include behavioral experiments.
Third-wave components	Mindfulness and acceptance and commitment therapy. Mindfulness is a form of meditation emphasizing a nonjudgmental state of heightened or complete awareness of one’s thoughts, emotions, or experiences on a moment-to-moment basis. Acceptance and commitment therapy focuses on accepting the feelings and thoughts associated with insomnia through value-based behaviors.
Constructive worry	Skills to overcome worry in bed by writing down the worries and their solutions before going to bed.
Behavioral components	
Sleep restriction	Skills to improve sleep by limiting time in bed. First, time in bed is restricted to the average sleep duration plus 30 minutes, and then it is increased or decreased depending on sleep efficiency.
Stimulus control	Skills to reassociate the bed with sleep. Patients are instructed to wake up at the same time every morning, refrain from daytime napping, go to bed only when sleepy, get out of bed when unable to sleep, and use the bed and bedroom for sleep and sex only.
Relaxation	Structured exercises to reduce somatic tension (eg, abdominal breathing, progressive muscle relaxation, autogenic training) and cognitive arousal (eg, guided imagery training).
Paradoxical intention	Exercise to remain awake as long as possible after getting into bed.
Other	
Nonspecific treatment effect	Effect of an intervention due to the patients’ belief that they are receiving some form of treatment. We classified miscellaneous skills not covered in other sections and not expected to have a large effect (eg, quasi-desensitization) as having a nonspecific treatment effect.
Waiting component	Participants are aware that they can receive active treatment after a waiting phase. If patients allocated to the waiting list control condition received some other potentially therapeutic components, we considered both the waiting component and the therapeutic components to be present.
Conventional drug treatment	Rated positive when conventional drug treatment is present (drug treatment is part of the protocol treatment) or allowed (we will note the percentage of patients taking the drug). Because this component was always present or absent in each trial, its effect size could not be estimated.
Delivery methods	
Individual	Individual interaction with therapists, whether in person or remote.
Group	Interaction with therapists as a member of a group.
In person	In-person interaction with therapists.
Online therapeutic guidance^a^	Therapeutic guidance in addition to remote self-help interventions. This may be provided on a scheduled basis or as needed. Technical support only is not included. We coded this component separately from interaction with therapists to see if adding therapeutic guidance to remote self-help interventions was effective.
Human encouragement^a^	Reminders provided by human beings to proceed with the remote self-help treatment program via telephone or email. This should not contain any support related to the therapeutic contents. Peer support, such as online discussion groups, was regarded as part of this component.
Automated encouragement^a^	Automated reminders to proceed with the treatment program. This should not contain any support related to the therapeutic contents.

^a^ Online therapeutic guidance, human encouragement, and automated encouragement components were counted only for remote self-help interventions.

### Search Methods for Identification of Studies

We conducted a comprehensive literature search in PubMed, the Cochrane Central Register of Controlled Trials, and PsycInfo on May 14, 2022, and updated the search on July 21, 2023. We used a combination of index and free terms of psychological treatments and insomnia. We also searched the World Health Organization International Clinical Trials Registry Platform. We imposed no date, language, or publication status filters but included only trials that reported sufficient details in English language. We checked reference lists of review articles for additional potentially eligible records. eMethods 1 in Supplement 1 lists the search strings used for each database.

### Data Collection and Analysis

#### Selection of Studies

Pairs of reviewers (Y.F., M.S., S.K., S.F.) independently screened titles and abstracts of all potential studies. We then retrieved study reports and pairs of review authors (Y.F., M.S., R.Y., S.N., S.K., M. Inoue, M. Ito, H.N., H.N.T., S.F.) independently screened full texts, identified studies for inclusion, and recorded reasons for exclusion of ineligible studies. We resolved any disagreement through discussion or, if required, through consultation with a third reviewer. We assessed the interrater reliability of the full-text screening decisions with Cohen κ and percentage agreement.

### Data Items

Pairs of reviewers (Y.F., M.S., R.Y., S.N., S.K., M. Inoue, M. Ito, H.N., T.A.F.) extracted data from the included studies independently. We classified all identified treatment arms and their components according to definitions in Table 1 using all available information from publications and inquiries with the original investigators. We assessed included trials using the revised risk of bias tool by Cochrane.^13^ Any disagreement was resolved through discussion or involving a third reviewer if necessary. We measured the interrater reliability of the component identification and of the risk of bias assessment with κ and percentage agreement, and that of the extracted primary outcomes with intraclass correlation.

### Primary Outcome and Secondary Outcomes

The primary outcome was treatment remission (defined as reaching a satisfactory state at end point measured by any validated self-reported scale) posttreatment. We prioritized intention-to-treat analyses whenever possible. When original publications did not report the number of individuals who remitted, we imputed remission based on continuous outcomes using a previously validated method.^14^ We assessed the validity of this imputation method by computing the intraclass correlation using studies that reported the outcome as both continuous and dichotomous. Secondary outcomes included all-cause dropouts (as a proxy measure of acceptability); various self-reported sleep continuity, including sleep efficiency (%), total sleep time (minutes), sleep latency (minutes), and wake after sleep onset (minutes); and long-term remission.

### Statistical Analysis

We created a network diagram at the treatment level to visualize the available evidence. The basic assumption of NMA is transitivity.^7^ Transitivity implies that we can combine the direct evidence from A vs C and B vs C studies to learn (indirectly) about the comparison A vs B. However, this will be questionable if there are important differences in the distribution of the effect modifiers across treatment comparisons. To assess transitivity, we created box plots of trial and patient characteristics deemed to be possible effect modifiers (publication year, proportion of patients with primary insomnia, age, and baseline severity) and visually examined whether they were similarly distributed across treatment comparisons. If transitivity does not hold, we may see inconsistencies in the network. We checked consistency using global (design-by-treatment) and local (back-calculation) tests.^15,16^ We conducted a random-effects, frequentist NMA at the treatment level for the primary outcome. We visualized NMA results using psychoeducation as reference and ordering treatments according to *P* scores, which provide an overall ranking of treatments.^17^ We also created a league table showing the estimated relative effects between all treatments in the network.

We assessed heterogeneity by looking at the standard deviation of random effects (τ^2^), comparing it against empirical distributions,^18^ and creating prediction intervals.^19^ We assessed possible reporting bias and small-study effects using contour-enhanced funnel plots of comparisons with 10 or more trials. We assessed certainty of evidence using Confidence in Network Meta-Analysis (CINEMA).^20^

We subsequently created a network diagram at the component level and performed a frequentist random-effects cNMA. We used an additive model—that is, we assumed that the effect of a combination therapy is the sum of the effects of its components and that there are no interactions between them. For dropout, we only used studies comparing active treatments because dropout cannot be meaningfully defined for inactive control individuals. For each component, cNMA estimates the incremental odds ratio for dichotomous outcomes, the incremental mean difference for continuous outcomes measured on the same scale, and the incremental standardized mean difference for continuous outcomes using multiple measures and corresponding 95% CIs. These measures show the added benefit or harm of including each component into a combination of other components. We presented each estimate and corresponding 95% CI using a coloring scheme where colors denote beneficial or harmful effect and shading shows the strength of statistical evidence.^21^ We assessed heterogeneity by comparing the estimated τ^2^ with empirical distributions.^18^

We performed the following prespecified sensitivity analyses on the primary outcome to examine the influence of including studies with informal diagnostic criteria, comorbidities, high dropout rates, and high overall risk of bias. We conducted further post hoc sensitivity analyses by excluding studies using severity scales other than the Insomnia Severity Index, excluding delivery formats as components, treating insomnia severity as a continuous outcome, excluding small trials, excluding inactive arms (no treatment, treatment as usual, waiting list), using risk difference as the summary measure, and including 2-way interactions among the components using a bayesian model with penalized regression.^22^ We did post hoc subgroup analyses to examine the effect of possible effect modifiers (age, sex, hypnotic use, comorbidities, and baseline severity). It can be difficult to interpret the clinical significance of odds ratios^23,24^; thus we translated them into more clinically interpretable indices (risk difference and number needed to treat), assuming different control event rates (first quartile, median, and third quartile of the observed remission rate among the psychoeducation arms). We performed analyses in R version 4.2.3 (R Foundation) using netmeta, meta, and rjags packages.

## Results

### Studies and Participants

We identified 9878 records, assessed 1872 full texts, and included 241 trials with a total of 31 452 randomized participants (PRISMA flow diagram in eFigure 1 in Supplement 1). The interrater reliability of judgments for full-text screening was substantial, with κ of 0.70 (95% CI, 0.66-0.74) and percentage agreement of 85.1%. eMethods 2 in Supplement 1 lists the included and excluded trials.

Typical participants were middle-aged women with psychological or physical comorbidities and moderate insomnia symptoms (mean [SD] age, 45.4 [16.6] years, based on 236 trials; 21 048 of 31 452 [67%] female, reported in 232 trials; 180 of 241 trials [75%] included insomnia with comorbidities; mean [SD] baseline Insomnia Severity Index score, 16.8 [4.8], reported in 176 trials) (Table 2).

**Table 2.** Characteristics of Included Patients and Trials

	Value	No.
Patient characteristics		
Age, mean (SD), y	45.4 (16.6)	236 Trials
Sex, No. (%)		
Female	21 048/31 452 (67)	232 Trials
Male	10 404/31 452 (33)	
Hypnotic use, No. (%)	4259/20 704 (21)	167 Trials
Comorbidities, type	Primary insomnia	60 Trials
	Psychotic comorbidities	42 Trials
	Physical comorbidities	75 Trials
	Mixed	64 Trials
Baseline severity (ISI score), mean (SD)	16.8 (4.8)	163 Trials
Diagnosis	Formal operationalized criteria	146 Trials
	Elevated score	70 Trials
	Others	25 Trials
Trial characteristics		
Region	North America	120 Trials
	Europe	61 Trials
	Asia	34 Trials
	Oceania	14 Trials
	Middle East	8 Trials
	South America	3 Trials
	Africa	1 Trial
No. of arms	2	206 Trials
	3	26 Trials
	4	7 Trials
	5	1 Trial
	6	1 Trial
Randomization unit	Individual	224 Trials
	Cluster	2 Trials
Publication year, mean (range)	2015 (1980-2023)	241 Trials
Intervention	Total	528 Arms
Treatment level	Cognitive behavioral therapy	210 Arms
	Behavioral therapy	64 Arms
	Cognitive therapy	17 Arms
	Relaxation therapy	16 Arms
	Psychoeducation	105 Arms
	Attention or psychological placebo	5 Arms
	Treatment as usual	8 Arms
	No treatment	5 Arms
	Waiting list	98 Arms
Components		
Educational components	Sleep hygiene education	324 Arms
	Sleep diary	433 Arms
Cognitive components	Cognitive restructuring	203 Arms
	Third-wave components	58 Arms
	Constructive worry	21 Arms
Behavioral components	Sleep restriction	244 Arms
	Stimulus control	249 Arms
	Paradoxical intention	20 Arms
	Relaxation	174 Arms
Others	Nonspecific treatment effect	423 Arms
	Waiting component	102 Arms
Delivery methods	Individual	228 Arms
	Group	100 Arms
	In person	287 Arms
	Online therapeutic guidance	39 Arms
	Human encouragement	49 Arms
	Automatic encouragement	41 Arms

Abbreviation: ISI, Insomnia Severity Index.

**Figure 1.**
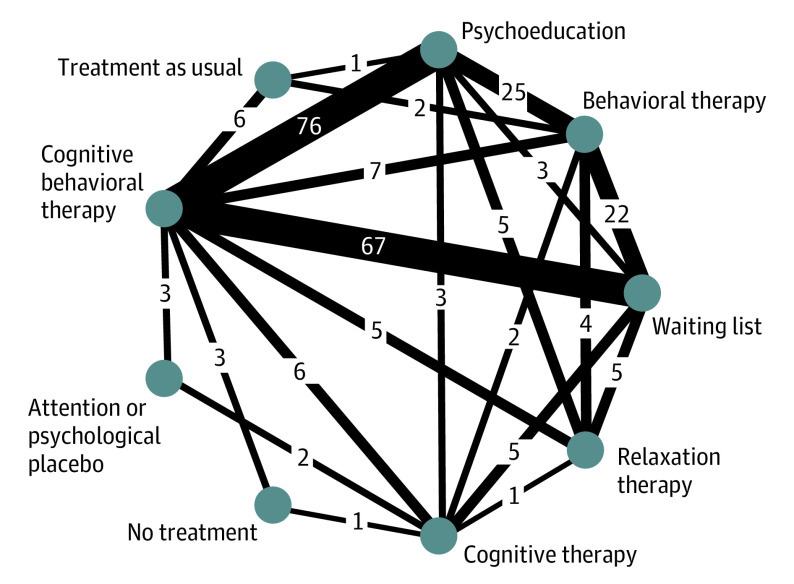
Network Diagram at Treatment Level The width of lines connecting treatments corresponds to the number of trials performing the corresponding comparison. This number is also given on each line. Absence of a line connecting 2 treatments means that this comparison was not performed in any study.

Operationalized formal diagnostic criteria were used in 146 trials. The 241 included trials had 528 arms (Figure 1). Most commonly included components in CBT arms were sleep hygiene education (198 of 210 [94%]), sleep diary (192 of 210 [91%]), cognitive restructuring (199 of 210 [95%]), sleep restriction (198 of 210 [94%]). and stimulus control (194 of 210 [92%]) (eTable 1 in Supplement 1).

Treatment duration ranged from 1 to 16 weeks (median, 6). Hypnotics were used in 4259 of 20 704 participants (21%). Publication years ranged from 1980 to 2023. eTable 2 in Supplement 1 tabulates characteristics of included trials. Interrater reliability of judgments for components ranged from moderate to almost perfect, with κ ranging from 0.43 to 0.85 and percentage agreement from 73.1% to 98.8% (eTable 3 in Supplement 1). Interrater reliability of extracted primary outcomes was almost perfect, with an intraclass correlation of 0.98 (95% CI, 0.98-0.98). Validity of the remission imputation method was confirmed, with an intraclass correlation of 0.96 (95% CI, 0.95-0.97) (eMethods 3 in Supplement 1).

The overall risk of bias according to the Cochrane revised risk of bias tool was low in 21 of 241 trials (9%), some concerns in 135 (56%), and high in 85 (35%). The interrater reliability for the overall risk of bias was fair, with a weighted κ of 0.33 (95% CI, 0.20-0.45) and percentage agreement of 53.4% (eMethods 4 in Supplement 1).

Assessment of transitivity (eFigure 2 in Supplement 1) showed that potential effect modifiers were evenly distributed across comparisons, except for the proportion of insomnia without comorbidities. We explored its impact in a sensitivity analysis.

### Treatment-Level Network Meta-Analysis

The network for the primary outcome at the treatment level was well connected (Figure 1). The global test did not show evidence of inconsistency. The back-calculation method identified 2 possibly inconsistent comparisons of 20, a proportion that would be empirically expected^25^ (eMethods 5 in Supplement 1).

**Figure 2.**
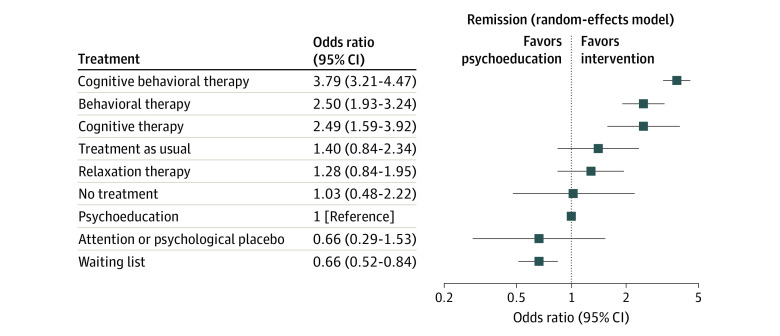
Treatments vs Psychoeducation as Estimated From Treatment-Level Network Meta-Analysis

Figure 2 shows the results of the treatment-level NMA, with treatments ranked according to their *P* scores. CBT was associated with the most increased likelihood of remission, followed by behavioral therapy and cognitive therapy, suggesting the benefits of both cognitive and behavioral skills. eTable 4 in Supplement 1 provides the league table.

Heterogeneity of the primary outcome was within the empirically expected range (τ^2^, 0.23; τ, 0.48; *I*^2^, 43% [95% CI, 34-52]).^18^ Prediction intervals did not change the overall interpretation of results (eFigure 3 in Supplement 1).

The contour-enhanced funnel plots suggested no publication or reporting biases. However, we found some evidence of possible small study effects, such as smaller studies showing consistently different results than bigger ones (eFigure 4 in Supplement 1). We explored the impact of this in a sensitivity analysis. Moderate certainty of evidence suggested the superiority of CBT, behavioral therapy and cognitive therapy over psychoeducation, and CBT over cognitive therapy, and low certainty of evidence CBT over behavioral therapy. Other comparisons were mainly of moderate to low certainty, except when no direct comparison was available and the certainty was considered very low (eTable 5 in Supplement 1).

### Component Network Meta-Analysis

**Figure 3.**
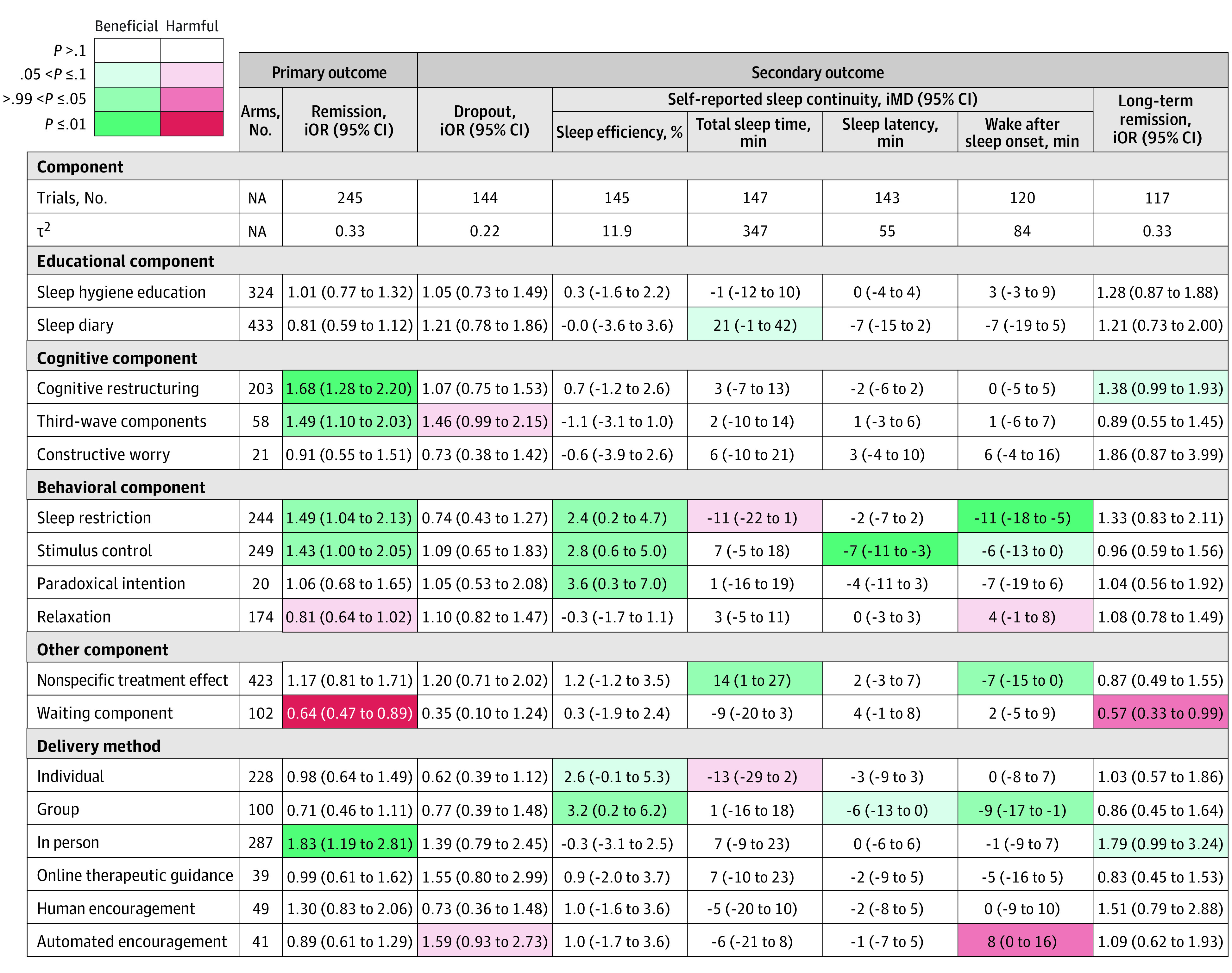
Estimated Associations for All Outcomes, Obtained From Component Network Meta-Analyses More details about the coloring scheme are provided in eFigure 6 in Supplement 1. τ^2^ shows variance of random effects (heterogeneity). For remission, iOR > 1.0 denotes beneficial components (increases remission) and iOR < 1.0 harmful components (decreases remission). For dropout, iOR < 1.0 denotes beneficial components (decreases dropout) and iOR > 1.0 harmful components (increases dropouts). For sleep efficiency, iMD > 0 denotes beneficial components (increases efficiency by X%) and iMD < 0 harmful components. For total sleep time, iMD > 0 denotes beneficial components (increases sleep time by X min) and iMD < 0 harmful components. For sleep latency, iMD < 0 denotes beneficial components (decreases latency by X min) and iMD > 0 harmful components. For wake after sleep onset, iMD < 0 denotes beneficial components (decreases wake time by X min) and iMD > 0 harmful components. iMD indicates incremental mean difference; iOR, incremental odds ratio.

The component-level network was well connected and densely populated (eFigure 5, eTable 3 in Supplement 1). Figure 3 shows estimated incremental odds ratios and mean differences for all components and outcomes. Heterogeneity for the primary outcome was within the empirically expected range (τ^2^, 0.33; τ, 0.58; *I*^2^, 50% [95% CI, 42-57]).^18^

The cNMA revealed that for the primary outcome, cognitive restructuring, third-wave components, sleep restriction, and stimulus control may be beneficial but relaxation might be detrimental. In-person contact with therapists may also be helpful. We found that the waiting component was associated with a decrease in remission rate, and thus, when used as the control, may inflate the perceived efficacy of the active arm. Including third-wave components may be associated with increased dropout.

In terms of self-reported sleep continuity, sleep restriction was associated with improved sleep efficiency and wake after sleep onset, and stimulus control with improved sleep efficiency and sleep latency. We did not find evidence of an association of cognitive restructuring, third-wave component, or in-person delivery with self-reported sleep continuity. Cognitive restructuring and in-person delivery may be beneficial for long-term remission (Figure 3).

Results of all sensitivity analyses largely agreed with our primary analyses (eFigure 7 in Supplement 1). cNMA using risk differences were in line with cNMA using odds ratios, albeit with greater heterogeneity. We found weak evidence of some 2-way interactions among the components; however, including interactions into the model did not materially change the overall conclusions. Subgroup analyses found no evidence that age, sex, hypnotic use, comorbidities, and baseline severity had an impact on the relative treatment effect (eFigure 8 in Supplement 1).

The first quartile, median, and third quartile of the remission rates among psychoeducation arms were 0.06, 0.14 and 0.26, respectively. A CBT-I package with the 4 beneficial treatment components in the in-person delivery format compared with in-person psychoeducation led to an odds ratio of 5.34 (95% CI, 3.56-8.01) and was associated with an increase in the remission rate by a risk difference of 0.19 (95% CI, 0.12-0.27) and number needed to treat of 5.3 (95% CI, 3.7-8.3), assuming a control event rate of 0.06; by a risk difference of 0.33 (95% CI, 0.23-0.43) and number needed to treat of 3.0 (95% CI, 2.3-4.3), assuming a control event rate of 0.14; and by a risk difference of 0.38 (95% CI, 0.28-0.47) and number needed to treat of 2.6 (95% CI, 2.1-3.6), assuming a control event rate of 0.26.

## Discussion

To our knowledge, this is the first systematic review and cNMA to evaluate CBT-I for chronic insomnia in adults with or without comorbidities. At the treatment level, we found that CBT was associated with the most increased likelihood of remission followed by behavioral therapy and cognitive therapy. At the component level, we found that cognitive restructuring, third-wave components, sleep restriction, and stimulus control were beneficial, while sleep hygiene and sleep diary were inert and relaxation potentially detrimental. Moreover, our results showed that various components may be associated with change in specific outcomes. Cognitive restructuring and third-wave components were beneficial for sleep quality, without significant changes in self-reported sleep continuity. On the other hand, sleep restriction was associated with improved wake after sleep onset and sleep efficiency, and stimulus control with improved sleep latency and sleep efficiency. With respect to the delivery format, in-person sessions may be recommended.

It is impossible to interpret the clinical significance of odds ratios by themselves.^26^ Risk differences and their corresponding numbers needed to treat are clinically more interpretable and meaningful.^24^ We can interpret the results from odds ratios by converting them into risk differences and numbers needed to treat assuming certain control event rates. For example, the CBT-I package including the 4 recommended treatment components in the in-person format, in comparison with psychoeducation in the in-person format, was associated with a risk difference of 0.33 (95% CI, 0.23-0.43) and therefore a number needed to treat of 3.0 (95% CI, 2.3 to 4.3), assuming an observed median control event rate of 0.14.

The study’s positive findings are particularly important as they may be the first demonstration that each component of CBT-I is associated with improved sleep quality. This supports the notion that behavioral therapy and cognitive therapy have different mechanisms of action.^27^ Cognitive restructuring and third-wave components may exert their effects by changing patient perception or beliefs about what constitutes severe, frequent, or intolerable symptomatology; sleep restriction may exert its effects via the manipulation of sleep homeostasis; and stimulus control may exert its effects by attenuating sleep effort or by instituting reconditioning. However, it should be noted that third-wave components are relatively new interventions and have only been explored in recent trials, and the effect sizes of novel treatments may be overestimated.^28^ Our findings may lead to personalized treatments for insomnia by matching individuals’ insomnia characteristics with appropriate techniques.

The null findings are also important. We did not find evidence of an association of sleep hygiene education and sleep diary, commonly included in CBT-I packages, with improved sleep quality. Our cNMA further found relaxation, also frequently included in CBT-I packages, to be potentially detrimental. The effectiveness of relaxation therapy as a package may be attributable to the nonspecific treatment effect and the inclusion of the in-person component rather than the specific efficacy of relaxation itself. In the treatment of insomnia, relaxation might lead to lying down longer while awake, which might counter sleep restriction or stimulus control. Such findings align with other cNMA results in mental disorders. For instance, a cNMA of CBT for panic disorder found muscle relaxation to be potentially harmful,^29^ and a cNMA of internet CBT for depression found relaxation to be potentially detrimental.^12^ For panic disorder, relaxation might have worked in the opposite direction of exposure, which is arguably the fundamental therapeutic mechanism for panic disorder.^12^ In the treatment of depression, relaxation might have discouraged behavioral activation, one of the principal therapeutic components for depression.^12^

Our results were in agreement with previous systematic reviews and meta-analyses in that individual or group in-person delivery formats are the most efficacious.^30^ However, our analyses did not find evidence of superiority of the in-person format against the self-help format with human encouragement (individual in-person format vs human encouragement). Given the prevalence and burden of chronic insomnia and the overwhelming need for safe and scalable remedies, self-help formats merit further investigation.

### Strengths and Limitations

The strengths of this study are as follows. First, we performed a comprehensive and up-to-date systematic review, allowing the inclusion of a large number of trials (n = 241). To our knowledge, this is by far the largest meta-analysis in the field of CBT-I and the largest cNMA in psychotherapies.^12,29^ Second, we used cutting-edge methods to assess the specific efficacies of various skills and delivery formats of CBT-I. Identification of the components is key in conducting the cNMA. We a priori made a table of the definitions of components (Table 1) and achieved moderate to almost-perfect interrater agreement (eTable 3 in Supplement 1).

Our study has several limitations. First, the overall quality of evidence for the network estimates at the treatment level varied from moderate to low or very low when only indirect comparisons were available. However, the quality of evidence at the component level was deemed to be moderate to low overall, as all incremental efficacies of components were based on some direct evidence. A sensitivity analysis confirmed that excluding high risk of bias studies did not change the overall findings. Second, the high dropout rate may have influenced the results. We conservatively considered those who dropped out from assessment as not remitting. We conducted a sensitivity analysis excluding trials with overall high dropout rates and results were consistent with the primary analyses. Third, our analysis considered components as either present or absent based on the descriptions provided in each study. In reality, components may have varied between programs in terms of their contents and the extent to which they were implemented. Fourth, the additivity assumption may not hold in practice. Even though the bayesian analyses examining 2-way interactions did not suggest any strong evidence against additivity, they may not be able to completely rule out meaningful interactions because the trials were not designed to test such interactions. Moreover, the contents of some components may not be completely mutually exclusive, although their procedures and putative mechanisms of action are sufficiently distinct to characterize and assess them as different components. Our analyses therefore should be regarded as hypothesis-generating and cannot be interpreted as conclusive. We need larger, better-designed trials to test whether certain combinations indeed outperform others. Our cNMA provides information on which components may deserve such further examination.

## Conclusions

The findings in this study suggest that the most beneficial and efficient CBT-I package may include cognitive restructuring, third-wave components, sleep restriction, and stimulus control in the individual or group in-person format. Sleep hygiene education may not be essential, while relaxation may be detrimental. To increase scalability, self-help programs with human encouragement warrant further trials. However, potential undetected interactions may compromise the conclusions. Further large-scale, well-designed trials are warranted to confirm the contribution of different treatment components in CBT-I.

## Notes

Supplement 1. eMethods 1. Protocol eMethods 2. Lists of excluded and included studies eMethods 3. Examination of the imputation method eMethods 4. The revised Cochrane risk of bias eMethods 5. Assessment of inconsistency for the primary outcome eTable 1. Components and delivery formats that are included in CBT eTable 2. Characteristics of included trials eTable 3. Evaluation of components eTable 4. League table for treatment-level network meta-analysis eTable 5. CINeMA eTable 6. PRISMA-NMA eFigure 1. Screening process and results eFigure 2. Assessment of transitivity eFigure 3. Prediction intervals eFigure 4. Assessment of publication bias and small study effects eFigure 5. Network diagram eFigure 6. The colouring scheme of the results eFigure 7. Sensitivity analyses eFigure 8. Subgroup analyses

Supplement 2. Data sharing statement
