# Supplementary material for: Components and Delivery Formats of Cognitive Behavioral Therapy for Chronic Insomnia in Adults: A Systematic Review and Component Network Meta-Analysis
Source: JAMA Psychiatry. 2024 Jan 17;81(4):357–65. doi: 10.1001/jamapsychiatry.2023.5060 (PMC10794978; doi:10.1001/jamapsychiatry.2023.5060)
Supplement: Supplement 1. — eMethods 1. Protocol eMethods 2. Lists of excluded and included studies eMethods 3. Examination of the imputation method eMethods 4. The revised Cochrane risk of bias eMethods 5. Assessment of inconsistency for the primary outcome eTable 1. Components and delivery formats that are included in CBT eTable 2. Characteristics of included trials eTable 3. Evaluation of components eTable 4. League table for treatment-level network meta-analysis eTable 5. CINeMA eTable 6. PRISMA-NMA eFigure 1. Screening process and results eFigure 2. Assessment of transitivity eFigure 3. Prediction intervals eFigure 4. Assessment of publication bias and small study effects eFigure 5. Network diagram eFigure 6. The colouring scheme of the results eFigure 7. Sensitivity analyses eFigure 8. Subgroup analyses [file jamapsychiatry-e235060-s001.pdf]

Supplemental Online Content

Furukawa Y, Sakata M, Yamamoto R, et al. Components and delivery formats of cognitive behavioral therapy for chronic insomnia in adults: a systematic review and component network meta-analysis. *JAMA Psychiatry*. Published online January 17, 2024. doi:10.1001/jamapsychiatry.2023.5060

eMethods 1. Protocol
eMethods 2. Lists of excluded and included studies
eMethods 3. Examination of the imputation method
eMethods 4. The revised Cochrane risk of bias
eMethods 5. Assessment of inconsistency for the primary outcome
eTable 1. Components and delivery formats that are included in CBT
eTable 2. Characteristics of included trials
eTable 3. Evaluation of components
eTable 4. League table for treatment-level network meta-analysis
eTable 5. CINeMA
eTable 6. PRISMA-NMA
eFigure 1. Screening process and results
eFigure 2. Assessment of transitivity
eFigure 3. Prediction intervals
eFigure 4. Assessment of publication bias and small study effects
eFigure 5. Network diagram
eFigure 6. The colouring scheme of the results
eFigure 7. Sensitivity analyses
eFigure 8. Subgroup analyses

This supplemental material has been provided by the authors to give readers additional information about their work.

© 2024 Furukawa Y et al. *JAMA Psychiatry.*

Table of Contents

eMethods 1. Protocol
1.1. Protocol as of 25 May, 2022
1.2. Changes from the protocol
1.3. List of all variables for which data were sought
1.4. Additive component network meta-analysis model
eMethods 2. Lists of excluded and included studies
2.1. List of the excluded studies with reasons (examples)
2.2. List of the ongoing trials and the terminated/withdrawn trials
2.2.1. List of the ongoing trials
2.2.2. List of the terminated/withdrawn trials
2.3. List of the included trials
eMethods 3. Examination of the imputation method
eMethods 4. The revised Cochrane risk of bias
4.1. Risk of Bias 2 interpretation
4.2 The inter-rater reliability of the assessment of risk of bias
4.3. Risk of Bias 2 of each trial
eMethods 5. Assessment of inconsistency for the primary outcome
eTable 1. Components and delivery formats that are included in CBT
eTable 2. Characteristics of included trials
eTable 3. Evaluation of components
3.1. Percentage agreement and kappa of components for each arm of included trials
3.2. Components of the included trials
3.3. Observed combinations of components and delivery formats
eTable 4. League table for treatment-level network meta-analysis
eTable 5. CINeMA
eTable 6. PRISMA-NMA
eFigure 1. Screening process and results
1.1. Flow diagram 2022 initial review
1.2. Flow diagram 2023 update
eFigure 2. Assessment of transitivity
eFigure 3. Prediction intervals
eFigure 4. Assessment of publication bias and small study effects
eFigure 5. Network diagram
eFigure 6. The colouring scheme of the results
eFigure 7. Sensitivity analyses
7.1. Pre-specified and post-hoc sensitivity analyses
7.2. NMA and cNMA using risk difference in comparison with odds ratio
7.3. Bayesian model to examine the effects of two-way interactions
eFigure 8. Subgroup analyses

© 2024 Furukawa Y et al. *JAMA Psychiatry.*

eMethods 1. Protocol

We prospectively registered the protocol in PROSPERO (CRD42022324233) on 10 May 2022. You can find the full text protocol on the preprint server (Furukawa Y, et al. medRxiv. 2022. doi: https://doi.org/10.1101/2022.06.02.22275890) Here we present the protocol as of 25 May, 2022 and the changes.

1.1. Protocol as of 25 May, 2022

First draft: 4 March, 2022

Last update before PROSPERO registration: 23 April, 2022

Last updated: 25 May, 2022

Dismantling cognitive-behavioral therapy for chronic insomnia: a protocol for a systematic review and component network meta-analysis

REVIEW QUESTION

What is the effect of each component of cognitive behavioral therapy for chronic insomnia?

BACKGROUND

Insomnia is highly prevalent and disabling.(Roth et al., 2011) Clinical practice guidelines recommend cognitive-behavioral therapy for insomnia (CBTI) as the first-line treatment.(Edinger et al., 2021; Qaseem et al., 2016) CBTI includes various combinations of many different components and its clinical benefits have been shown as a package,(Straten et al., 2018) whereas the effect of each component remains unclear. Finding the effect of each component will lead to an intervention that may maximize treatment benefit while reducing the number of components, thereby reducing the treatment burden, lowering the cost of CBTI and making it available to more people. Component network meta-analysis (CNMA) is an extension of standard network meta-analysis that can be used to disentangle the treatment effects of different components included in multicomponent interventions. (Rücker et al., 2020b) In this study, we will explore the effect of each component of CBTI with the use of CNMA.

METHODS

We will follow the Preferred Reporting Items for Systematic reviews and Meta-Analyses (PRISMA) guideline extension for NMA. (Hutton et al., 2015) The protocol is prospectively registered in PROSPERO (CRD42022324233).

Data sources

Criteria for considering studies for this review

Study design

© 2024 Furukawa Y et al. *JAMA Psychiatry.*

We will include all randomized controlled trials that compared any form of CBTI against another form of CBTI or a control condition in the treatment of adults with chronic insomnia. Cluster randomized trials will be included in meta-analyses in accordance with the Cochrane handbook recommendation. (Higgins et al., 2022) If the intra-cluster correlation coefficient is not clearly reported, we assume it to be 0.05.(Lin et al., 2018)

Participants

We will include studies on patients of both genders aged 18 years or older with insomnia either diagnosed according to formal diagnostic criteria (such as the Diagnostic and Statistical Manual of Mental Disorders, the International Classification of Diseases or the International Classification of Sleep Disorders) or judged so by clinical expertise (e.g. presence of significant symptoms). The effect of including studies without a formal diagnosis of insomnia will be tested in a sensitivity analysis. We will include patients with psychiatric or physical comorbidities. (Sateia, 2014) The effect of including such studies will be examined in a sensitivity analysis.

Interventions and controls

We regard CBTI broadly as a psychotherapy involving any one of the following cognitive or behavioral components. Table 1 describes the different components of interest and their definitions. Pharmacological co-administration will be allowed as long as it is stated that there were no systematic differences in drug administration between study arms. CBTI that clearly includes active components focusing on other symptoms, such as depression, anxiety or pain, will be excluded.

The control conditions of interest will include waiting list, no treatment, attention/psychological placebo control and treatment as usual. In this CNMA study, treatment as usual must include pharmacotherapy; watchful waiting will be classified as attention/psychological placebo even when it is termed ‘treatment as usual’ in some papers. When no treatment, attention/psychological placebo or treatment as usual are used while on the waiting list, such controls will be regarded as waiting list in the NMA but will be decomposed as appropriate in the CNMA. We will include interventions of any duration.

Where multiple arms are reported in a single trial, we will include only the relevant arms that can be described with the components listed in Table 1. We will lump the arms when they share the same components (e.g. same components delivered in 30 min per session against 60 min per session). We will exclude irrelevant arms such as active-drug, pill-placebo, exercise or bright light therapy with equipment.

Possible components and their combinations are shown in Table 1 and Table 2. The components and nodes were determined based on previous studies (Edinger et al., 2021; Furukawa et al., 2021) and content expert consensus among co-authors (MS, TAF, MP).

TABLE 1 List of included components and their definitions

Intervention	Description
**Educational components (EDU)**	
Sleep hygiene education (se)	General explanation about sleep (eg, sleep biology, characteristics of healthy sleep, changes in sleep patterns with aging, stress biology, and its impact on sleep) and general recommendations about lifestyle (eg, diet, exercise, substance use) and environmental factors (eg, light, noise,

Intervention	Description
	temperature) to improve sleep. This may include some elements of other components but those should not be the predominant part of the intervention.
Sleep diary (sd)	Self-monitoring of important daily sleep-related information using diary.
**Cognitive components (COG)**	
Cognitive restructuring (cr)	Skills to identify, challenge and change unhelpful beliefs about sleep that may disturb sleep. This may include behavioral experiments.
Mindfulness (mi)	A form of meditation emphasizing a nonjudgmental state of heightened or complete awareness of one’s thoughts, emotions, or experiences on a moment-to-moment basis.
Constructive worry (cw)	Skills not to worry in bed by writing down the worries and their solutions before going to bed.
Imagery rehearsal (ir)	Skills to identify, challenge and change nightmares that disturb sleep.
**Behavioral components (BEH)**	
Sleep restriction (sr)	Skills to improve sleep by limiting time in bed. First, time in bed is restricted to the average sleep duration and then it is increased or decreased depending on sleep efficiency.
Stimulus control (sc)	Skills to re-associate the bed with sleep. Patients are instructed to; wake up at the same time every morning, refrain from daytime napping, go to bed only when sleepy, get out of bed when unable to sleep, and use the bed/bedroom for sleep and sex only.
Relaxation (re)	Structured exercises designed to reduce somatic tension (eg, abdominal breathing, progressive muscle relaxation, autogenic training) and cognitive arousal (eg, guided imagery training).
Paradoxical intention (pi)	Exercise to remain awake as long as possible after getting into bed.
**Others**	
Waiting component (w)	Participants are aware that they can receive an active treatment after a waiting phase. If patients allocated to the waiting list control condition receive some other potentially therapeutic components, we will consider both the waiting component and the therapeutic components to be present.
Conventional drug treatment (dt)	Rated positive when conventional drug treatment is present (drug treatment is part of the protocol treatment) or allowed (we will note the percentage of patients on drug).
Non-specific treatment effect (ns)	Effect of an intervention due to the patients’ belief that they are receiving some form of treatment. Miscellaneous skills not covered in other

© 2024 Furukawa Y et al. *JAMA Psychiatry.*

Intervention	Description
	sections and not expected to have large effect (i.e. quasi-desensitization) are classified as having non-specific treatment effect.
Self-help, unguided, remote (NA)	Default delivery format is self-help, unguided and remote, as it requires the least human resource.
Human encouragement (he)	Reminders provided by human beings to proceed with the self-help, remote treatment program via telephone or email. This should not contain any support related to the therapeutic contents. Peer support such as discussion group will be regarded as this component. We will code this component separately from interaction with therapists (in, gp, ff) to see if adding human encouragement to self-help, remote interventions good enough to be effective.
Therapeutic guidance (tg)	Therapeutic guidance in addition to self-help, remote interventions. This may be provided on a scheduled basis or as-needed basis. Technical support only is not included. We will code this component separately from interaction with therapists (in, gp, ff) to see if adding therapeutic guidance to self-help, remote interventions good enough to be effective.
Individual (in)	Individual interaction with therapists. The combination (in + ff) means individual, face-to-face sessions are held. The combination (in – ff) individual remote sessions, such as via telephone or videoconference.
Group (gp)	Interaction with therapists as a member of a group.
Face-to-face (ff)	Face-to-face interaction with therapists.
Automatic encouragement (ae)	Automated reminders to proceed with the treatment program. This can be added both to the self-help interventions or the interventions including interactions with therapists. This should not contain any support related to the therapeutic contents.

© 2024 Furukawa Y et al. *JAMA Psychiatry.*

TABLE 2 Conceptualization of cognitive behavioral therapy for insomnia or control conditions from the component perspective

	**Possible combinations of components**
Cognitive behavioral therapy	+ ns ± EDU + COG + BEH ± dt ± he ± tg ± in ± gp ± ff ± ae
Cognitive therapy	+ ns ± EDU + COG ± dt ± he ± tg ± in ± gp ± ff ± ae
Behavioral therapy	+ ns ± EDU + BEH ± dt ± he ± tg ± in ± gp ± ff ± ae
Psychoeducation	+ ns + EDU ± dt ± he ± tg ± in ± gp ± ff ± ae
Waiting list	+ w ± ns ± EDU ± dt ± he ± tg ± in ± gp ± ff ± ae
Treatment as usual	+ ns + dt + in + ff
Attention or psychological placebo	+ ns ± dt ± he ± tg ± in ± gp ± ff ± ae
No treatment	± ff ± in

Components marked with a "+" are required. Components marked with "±" are optional. Components not mentioned cannot be included. Capitalized components (EDU, COG, BEH) mean at least one of the components in that group is required. ae=automatic encouragement. BEH=behavioral components. COG=cognitive components. dt=conventional drug treatment. EDU=educational components. ff=face-to-face. gp=group. he=human encouragement. in=individual. ns=non-specific treatment effect. tg=therapeutic guidance. w=waiting component.

Search methods for identification of studies

We will carry out a comprehensive literature search in PubMed, CENTRAL and PsycINFO. We will use a combination of index and free terms of psychological treatments and insomnia with filters (Eady et al., 2008) for randomized clinical trials. We will also search WHO International Clinical Trials Registry Platform. We will impose no date, language or publication status restriction. We will check the reference lists of review articles for additional potentially eligible records.

TABLE 3 Search strings for PubMed, Cochrane Central Register of Controlled Trials and PsycINFO

Database	Search strings
PubMed	(Psychotherapy [MH] OR psychotherap*[All Fields] OR "cognitive behavioural therapy"[All Fields] OR "cognitive behavioral therapy"[All Fields] OR "CBT"[All Fields] OR "CBTI"[All Fields] OR "CBT-I"[All Fields] OR "cognitive therapy"[All Fields] OR "behavioural therapy"[All Fields] OR "behavioral therapy"[All Fields] OR "sleep hygiene"[All Fields] OR "sleep education"[All Fields] OR "sleep diary"[All Fields] OR "cognitive restructuring"[All Fields] OR "mindfulness"[All Fields] OR "constructive worry"[All Fields] OR "imagery rehearsal"[All Fields] OR "relaxation"[All Fields] OR "sleep restriction"[All Fields] OR "stimulus control"[All Fields] OR "paradoxical intention"[All Fields])<br>AND<br>("sleep wake disorders"[MeSH Terms] OR insomnia[All Fields])<br>AND

© 2024 Furukawa Y et al. *JAMA Psychiatry.*

Database	Search strings
	(randomized controlled trial[pt] OR controlled clinical trial[pt] OR randomized[tiab] OR placebo[tiab] OR clinical trials as topic[mesh:noexp] OR randomly[tiab] OR trial[ti] NOT (animals[mh] NOT humans [mh]))
Cochrane Central Register of Controlled Trials	([mh Psychotherapy] OR psychotherap* OR "cognitive behavioural therapy" OR "cognitive behavioral therapy" OR "CBT" OR "CBTI" OR "CBT-I" OR "cognitive therapy" OR "behavioural therapy" OR "behavioral therapy" OR "sleep hygiene" OR "sleep education" OR "sleep diary" OR "cognitive restructuring" OR mindfulness OR "constructive worry" OR "imagery rehearsal" OR relaxation OR "sleep restriction" OR "stimulus control" OR "paradoxical intention")<br>AND<br>([mh "sleep wake disorders"] OR insomnia)
PsycINFO	(exp Psychotherapy/ OR psychotherap*.af. OR "cognitive behavioural therapy".af. OR "cognitive behavioral therapy".af. OR "CBT".af. OR "CBTI".af. OR "CBT-I".af. OR "cognitive therapy".af. OR "behavioural therapy".af. OR "behavioral therapy".af. OR "sleep hygiene".af. OR "sleep education".af. OR "sleep diary".af. OR "cognitive restructuring".af. OR mindfulness.af. OR "constructive worry".af. OR "imagery rehearsal".af. OR relaxation.af. OR "sleep restriction".af. OR "stimulus control".af. OR "paradoxical intention".af.)<br>AND<br>(exp "sleep wake disorders"/ OR insomnia.af.)<br>AND<br>("double-blind" OR "random* assigned" OR control)
WHO International Clinical Trials Registry Platform	(psychotherap* OR "cognitive behavioural therapy" OR "cognitive behavioral therapy" OR "CBT" OR "CBTI" OR "CBT-I" OR "cognitive therapy" OR "behavioural therapy" OR "behavioral therapy" OR "sleep hygiene" OR "sleep education" OR "sleep diary" OR "cognitive restructuring" OR mindfulness OR "constructive worry" OR "imagery rehearsal" OR relaxation OR "sleep restriction" OR "stimulus control" OR "paradoxical intention")<br>AND<br>("sleep wake disorders" OR insomnia)

Data collection and analysis

Selection of studies

Two review authors will independently screen titles and abstracts of all the potential studies we identify as a result of the search and code them as ‘retrieve’ or ‘do not retrieve’. We will retrieve the full text study reports/publications and two review authors will independently screen the full text and identify studies for inclusion and identify and record reasons for exclusion of the ineligible studies. We will resolve any disagreement through discussion or, if required, we will consult a third reviewer. We will identify publications from the same study so that each study rather than each report is the unit of analysis in the review. We will record the selection process in sufficient detail to complete a PRISMA flow diagram.

Data items

Two review authors will extract independently data from the included studies. Any disagreement will be resolved through discussion, or discussed with a third person if necessary. We will abstract the following information.

1. Characteristics of the studies

Name of the study, year of publication, country, study site (single or multi-center), study design (individually-randomized or cluster-randomized), population characteristics (mean age, number of women, definition of insomnia, number of patients with primary insomnia), intervention (components, delivery format, qualification of therapists [if applicable], duration), outcomes (scale used for the primary outcome)

2. Identification of components

Two independent reviewers will determine the classification of all identified arms and their components according to the definitions in Table 1, based on all available information including the publications, trial registry and inquiry with the original investigators if needed. Any disagreement will be solved by the two reviewers and, where necessary, in consultation with a third member of the review team. We will report the inter-rater agreement in terms of percentage agreement and kappa.

3. Risk of bias

We will use Cochrane Risk of Bias 2.0 tool (RoB2) (Sterne et al., 2019) to assess the risk of bias of the primary outcome. We will report the inter-rater agreement in terms of percentage agreement and kappa.

4. Data to calculate effect sizes

We will extract data to calculate effect sizes (the number of patients randomized to each arm, the number of patients assessed, the number of remitters, the scale used, the mean, standard deviation and the number assessed for continuous outcomes) When only change from baseline to endpoint is reported for continuous outcomes, we will use it instead of endpoint mean.(Costa et al., 2013)

Primary outcome and secondary outcomes

The primary outcome of interest in this study is treatment efficacy at four weeks post-treatment or at its closest time point. Intention-to-treat analysis will be prioritized whenever available. We will use the number of participants randomized as the denominator for dichotomous outcomes. We will use odds ratio for dichotomous outcomes, mean difference for continuous outcomes expressed in minutes and percent.

1. Efficacy: remission defined as reaching a satisfactory state at endpoint measured by any validated self-reported scale (dichotomous)

Secondary outcomes are as follows;

2. Acceptability: dropouts for any reason (dichotomous)

3. Sleep diary measures (continuous)

3.1. Sleep efficiency (SE, %)

3.2. Total sleep time (TST, min)

3.3. Sleep onset latency (SOL, min)

3.4. Wake after sleep onset (WASO, min)

4. Efficacy at long-term follow-up. (dichotomous, longest follow-up between 3 to 12 months)

Hierarchy of outcome measures

© 2024 Furukawa Y et al. *JAMA Psychiatry.*

For efficacy, we will prioritize the remission using the Insomnia Severity Index (7 or less points at endpoint) (Morin et al., 2011) and its imputed number. If it is not reported, we will use the following scales in this order: the remission using the Functional Outcomes of Sleep Questionnaire-10 (18 or more points at endpoint) (Weaver et al., 2007), and then its imputed number; remission using the Epworth Sleepiness Scale (10 or less points at endpoint) (Johns, 1990), and then its imputed number; remission using the Pittsburgh Sleep Quality Index (5 or less points at endpoint) (Cole et al., 2006), and then its imputed number; remission using the Athens Insomnia Scale (5 or less points at endpoint) (Okajima et al., 2020), and then its imputed number; remission using any other validated self-reported scales; remission using sleep diary measures (both SOL and WASO less than 30 minutes at endpoint. When SOL and WASO are reported only separately, we will prioritize WASO. When only SOL is reported, we will use SOL.) (Edinger et al., 2009; Medicine, 2014) and its imputed number.

When any of the measure is reported using another definition of remission than stated above, we will use the definition stated by the authors. When any of the measure is reported only in continuous values, we will impute remission using mean and standard deviation. We will test the validity of this imputation method using studies that report the outcome both in continuous and dichotomous manner. When any of the measure is reported only in standardized mean difference, we will convert it into odds ratio using a validated method. (Chinn, 2000)

Statistical analysis

We will perform the analysis in *R* (latest version, R foundation, Vienna, Austria) (R Core Team, 2020) using *netmeta* (latest version) package (Rücker et al., 2020a) to conduct component network meta-analysis and *meta* (latest version) package (Balduzzi et al., 2019) to synthesize the outcomes in placebo arms and to assess the publication bias.

We will first perform a network meta-analysis lumping arms that include both cognitive and behavioral components as “cognitive-behavioral therapy,” those that involve cognitive but not behavioral components as “cognitive therapy” and those that involve behavioral but not cognitive components as “behavioral therapy” to gain a first insight of the relative treatment effects. (Table 2) We will examine the transitivity assumption by creating a table of important trial and patient characteristics to see if potential effect modifiers (publication year, proportion of patients with primary insomnia, age) are similarly distributed among comparisons. We will check the consistency of the network using local and global inconsistency tests.

Then we will perform component-level network meta-analysis. We will use a model that assumes additivity of components, i.e. assuming that the effect of combination therapy is the sum of the effects of its components. Given the expected clinical and methodological heterogeneity of treatment effects among the studies, we will use the random-effects model.

Certainty of evidence

We will assess the certainty of evidence in network estimates of the primary outcome using CINeMA. (Nikolakopoulou et al., 2020)

Publication bias

© 2024 Furukawa Y et al. *JAMA Psychiatry.*

We will assess the presence of small study effects, including publication bias, in the evidence set by examining asymmetry in the contour-enhanced funnel plots of all active interventions (CBT, CT, BT) vs control condition (psychoeducation, waiting list, treatment as usual, attention or psychological placebo, no treatment) using the primary outcome.

Sensitivity analyses

1. Excluding studies without formal diagnosis of insomnia
2. Excluding studies focusing on patients with comorbidities (both physical and psychological)
3. Excluding studies with overall high dropout rate (20% or more)
4. Excluding studies at high overall risk of bias
5. Using completer-set analysis (dichotomous)

Patient and public involvement

There was no patient or public involvement in the development of this manuscript.

Acknowledgements

The views expressed are those of the authors and not necessarily those of affiliated organizations.

Registration

This protocol is prospectively registered in PROSPERO (CRD42022324233).

This research was prospectively registered (#2022033NIe), Ethical Committee, Faculty of Medicine, The University of Tokyo.

Changes from the protocol (since the first registration on PROSPERO)

25th May, 2022 (during screening, before data extraction). Although we stated in the protocol that "CBTI that clearly includes active components focusing on other symptoms, such as depression, anxiety or pain, will be excluded," in case the same intervention component was included in both arms, we decided to include the trial, as we will be able to see the additional benefit of CBTI.

Support

No financial support was used.

Declarations of interest

YF has received consultancy fee from Panasonic outside the submitted work.

MS reports personal fees from SONY outside the submitted work.

SF has a research grant from JSPS KAKENHI Grant Number JP 20K18964 and the KDDI Foundation.

SK has a research grant from Mental Health Okamoto Memorial Foundation and Fujiwara Memorial Foundation.

© 2024 Furukawa Y et al. *JAMA Psychiatry.*

TAF reports grants and personal fees from Mitsubishi-Tanabe, personal fees from SONY, grants and personal fees from Shionogi, outside the submitted work; In addition, TAF has a patent 2020-548587 concerning smartphone CBT apps pending, and intellectual properties for Kokoro-app licensed to Mitsubishi-Tanabe.
EGO has received research and consultancy fees from Angelini Pharma. EGO is supported by the National Institute for Health Research (NIHR) Research Professorship to Professor Andrea Cipriani (grant RP-2017-08-ST2-006), by the National Institute for Health Research (NIHR) Applied Research Collaboration (ARC) Oxford and Thames Valley, by the National Institute for Health Research (NIHR) Oxford cognitive health Clinical Research Facility and by the NIHR Oxford Health Biomedical Research Centre (grant BRC-1215-20005)
OE was supported by the Swiss National Science Foundation (Ambizione grant number 180083)
MP reports no competing interest.

REFERENCE

- American Academy of Sleep Medicine. 2014. International Classification of Sleep Disorders.
- Balduzzi S, Rücker G, Schwarzer G. How to perform a meta-analysis with R: a practical tutorial. *Évid Based Ment Heal* 2019;22:153.
- Chinn S. A simple method for converting an odds ratio to effect size for use in meta-analysis. *Stat Med.* 2000;19:3127–31.
- Cole JC, Motivala SJ, Buysse DJ, et al. Validation of a 3-Factor Scoring Model for the Pittsburgh Sleep Quality Index in Older Adults. *Sleep* 2006;29:112–6.
- Costa BR da, Nüesch E, Rutjes AW, et al. Combining follow-up and change data is valid in meta-analyses of continuous outcomes: a meta-epidemiological study. *J Clin Epidemiol* 2013;66:847–55.
- Eady AM, Wilczynski NL, Haynes RB. PsycINFO search strategies identified methodologically sound therapy studies and review articles for use by clinicians and researchers. *J Clin Epidemiol*. 2008;61:34–40.
- Edinger JD, Arnedt JT, Bertisch SM, et al. Behavioral and psychological treatments for chronic insomnia disorder in adults: an American Academy of Sleep Medicine clinical practice guideline. *J Clin Sleep Med*. 2021;17:255–62.
- Edinger JD, Olsen MK, Stechuchak KM, et al. Cognitive Behavioral Therapy for Patients with Primary Insomnia or Insomnia Associated Predominantly with Mixed Psychiatric Disorders: a Randomized Clinical Trial. *Sleep.* 2009;32:499–510.
- Furukawa TA, Suganuma A, Ostinelli EG, et al. Dismantling, optimising, and personalising internet cognitive behavioural therapy for depression: a systematic review and component network meta-analysis using individual participant data. *Lancet Psychiatry*. 2021;8:500–11.
- Higgins J, Eldridge S, Li T. Chapter 23: Including variants on randomized trials. In: Higgins J, Eldridge S, Li T, editors. Cochrane Handbook for Systematic Reviews of Interventions version 6.3, 2022.
- Hutton B, Salanti G, Caldwell DM, et al. The PRISMA Extension Statement for Reporting of Systematic Reviews Incorporating Network Meta-analyses of Health Care Interventions: Checklist and Explanations.

© 2024 Furukawa Y et al. *JAMA Psychiatry.*

Ann Intern Med. 2015;162:777–84.
- Johns MW. A New Method for Measuring Daytime Sleepiness: The Epworth Sleepiness Scale. *Sleep.* 1990;14:540–5.
- Lin C-Y, Strong C, Scott AJ, et al. A cluster randomized controlled trial of a theory-based sleep hygiene intervention for adolescents. *Sleep*. 2018;41.
- Morin CM, Belleville G, Bélanger L, Ivers H. The Insomnia Severity Index: Psychometric Indicators to Detect Insomnia Cases and Evaluate Treatment Response. *Sleep*. 2011;34:601–8.
- Nikolakopoulou A, Higgins JPT, Papakonstantinou T, et al. CINeMA: An approach for assessing confidence in the results of a network meta-analysis. *Plos Med*. 2020;17:e1003082.
- Okajima I, Miyamoto T, Ubara A, et al. Evaluation of Severity Levels of the Athens Insomnia Scale Based on the Criterion of Insomnia Severity Index. *Int J Environ Res Pu*. 2020;17:8789.
- Qaseem A, Kansagara D, Forciea MA, et al. Management of Chronic Insomnia Disorder in Adults: A Clinical Practice Guideline From the American College of Physicians. *Ann Intern Med*. 2016;165:125.
- R Core Team. R: A language and environment for statistical computing. R Foundation for Statistical Computing. 2020.
- Roth T, Coulouvrat C, Hajak G, et al. Prevalence and Perceived Health Associated with Insomnia Based on DSM-IV-TR; International Statistical Classification of Diseases and Related Health Problems, Tenth Revision; and Research Diagnostic Criteria/International Classification of Sleep Disorders, Second Edition Criteria: Results from the America Insomnia Survey. *Biol Psychiat*. 2011;69:592–600.
- Rücker G, Krahn U, König J, et al. netmeta: Network Meta-Analysis using Frequentist Methods. 2020a.
- Rücker G, Petropoulou M, Schwarzer G. Network meta-analysis of multicomponent interventions. *Biometrical J Biometrische Zeitschrift* 2020b;62:808–21.
- Sateia MJ. International Classification of Sleep Disorders-Third Edition. *Chest*. 2014;146:1387–94.
- Sterne JA, Savović J, Page MJ, et al. RoB 2: a revised tool for assessing risk of bias in randomised trials. *BMJ*. 2019;366:l4898.
- Van Straten A, van der Zweerde T, Kleiboer A, et al. Cognitive and behavioral therapies in the treatment of insomnia: A meta-analysis. *Sleep Med Rev.* 2018;38:3–16.
- Weaver TE, Maislin G, Dinges DF, et al. Relationship Between Hours of CPAP Use and Achieving Normal Levels of Sleepiness and Daily Functioning. *Sleep*. 2007;30:711–9.

© 2024 Furukawa Y et al. *JAMA Psychiatry.*

1.2. Changes from the protocol.

25th May 2022 During screening, before data extraction. Although we stated in the protocol that "CBTI that clearly includes active components focusing on other symptoms, such as depression, anxiety or pain, will be excluded," in case the same intervention component was included in both arms, we decided to include the trial, as we would be able to see the additional benefit of CBTI.

4th August 2022. During screening, before data extraction, we decided to add “acceptance and commitment therapy” as eligible components. We decided to lump “acceptance and commitment therapy” together with the “mindfulness” component and call them “third-wave components”. We decided to exclude “imagery rehearsal” because it was primarily examined among PTSD patients and including this element would violate the transitivity assumption, which is critical for conducting network meta-analysis.

6th September 2022. During screening, before data extraction, we decided to use the Sleep Condition Indicator (remission: 17 or more points at endpoint) as an outcome measure and prioritise it next to ISI.

5th January 2023. During data extraction trial, we noticed that using the post-treatment time point allows for a clearer distinction from the long-term effect than using the 4-week post-treatment time point. We therefore decided to use the post-treatment time point for the primary outcome.

10th March 2023. We decided to use the baseline severity to test the transitivity assumption in addition to publication year, proportion of patients with primary insomnia and age.

18th March 2023. We decided not to conduct completer set analysis because such information was often not available.

19th April 2023. We decided to use 85% or more as the remission threshold for sleep efficiency.

19th April 2023. We noticed that we classified the relaxation technique as a behavioural component and thus relaxation without sleep restriction/stimulus control/paradoxical intention was classified as BT, which is clinically unreasonable. We thus added relaxation therapy (RT) as a node in the treatment-level network.

23rd April 2023. We decided to conduct additional sensitivity analyses 1) focusing on trials using ISI as the outcome measure to see the influence of choice of measurements; 2) excluding delivery format components (ind, gp, ff, ae, he) to see the influence of extracting delivery methods as components; 3) treating insomnia severity as a continuous value instead of a binary outcome to examine the influence of dichotomizing the outcome; and 4) excluding arms with less than 10 participants to see the influence of small study effects.

16th August 2023. We performed a Bayesian cNMA with penalizing regression to examine the possible two-way interactions, and subgroup analyses to examine whether certain subgroups respond differently.

© 2024 Furukawa Y et al. *JAMA Psychiatry.*

5th October 2023. We performed sensitivity analyses using risk difference as the summary measure. We also performed a sensitivity analysis excuding inactive arms.

© 2024 Furukawa Y et al. *JAMA Psychiatry.*

1.3. List of all variables for which data were sought

variable	description
Year	Publication year of the primary report. If only the results from registry were available, we used the date when the results were posted.
Ind_clu	Unit of randomization. Individual or cluster.
ICC_for_cRCT	If it was a cluster RCT, we extracted Intra-cluster Correlation Coefficient. If it was not clearly stated, use "0.05".
Country	Countries where the trial took place.
Single_multi	Whether the recruitment took place at a single or multi centers.
Age_mean	Mean age of the patients in the arm
Age_sd	Standard deviation of the age.
Age_n	The number of patients used for calculating Age_mean.
F_n	Number of females in the arm
Hyp_n	Number of patients using hypnotics. If the drug treatment was part of the protocol treatment, "Hyp_n" was equal to "n" (all the participants). If the drug treatment was not part of the protocol treatment but just allowed, we sued the number at baseline. We prioritised prescribed drugs to OTC drugs.
Insomnia diagnosis	If more than one formal diagnostic criteria were mentioned, prioritise DSM, ICSD and then ICD.<br>1. formal_DSM<br>1. formal_ICSD<br>1. formal_ICD<br>1. formal_Edinger2004<br>2. informal_ISI<br>2. informal_SCI<br>2. informal_PSQI<br>2. informal_AIS<br>2. informal_other_self_reported_scales<br>3. others
Primary insomnia_n	The number of primary insomnia (insomnia without comorbidities) patients in the arm.
Comorbidities	0. primary_insomnia<br>1. primary_secondary_mixed<br>2. psy_MDD<br>2. psy_anxiety<br>2. psy_PTSD

© 2024 Furukawa Y et al. *JAMA Psychiatry.*

variable	description
	2. psy_schizophrenia_or_psychosis<br>2. psy_bipolar<br>2. psy_substance_dependence<br>2. psy_others<br>2. psy_mixed<br>3. phy_dialysis<br>3. phy_heart_failure<br>3. phy_diabetes<br>3. phy_fibromyalgia<br>3. phy_cancer<br>3. phy_steroid<br>3. phy_pregnancy_perinatal<br>3. phy_menopause<br>3. phy_others<br>3. phy_mixed<br>4. psy_phy_mixed
n	The number of patients **randomized** to the arm.
remission	The number of patients achieving remission at post-treatment or at its closest time point. If equidistant, we used the longer timeframe. (dichotomous)
def_remission_response	Definition of remission..
scale used	The scale used for the definition of remission. Prioritize; ISI > SCI > FOSQ > ESS > PSQI > AIS > other_self_reported_scales > WASO_SOL > WASO > SOL. We will use the definition stated by the author. Note that we used sleep diary measures (subjective), not polysomnography measures (objective).
Severity_bl_mean	Mean severity measured at baseline.
Severity_bl_sd	Standard deviation of the above.
Severity_bl_n	The number of patients measured their severity at baseline.
Severity_ch_mean	Extracted only when Severity_ep_mean, Severity _ep_sd and Sevrity_ep_n were not available.<br>Mean change of Severity from baseline to the endpoint (post-treatment or at its closest time point).
Severity_ch_sd	Extracted only when Severity_ep_mean, Severity _ep_sd and Sevrity_ep_n were not available.<br>Standard deviation of the above.

© 2024 Furukawa Y et al. *JAMA Psychiatry.*

variable	description
Severity_ch_n	Extracted only when Severity_ep_mean, Severity _ep_sd and Sevrity_ep_n were not available. The number of patients measured their severity change from baseline to the endpoint (post-treatment or at its closest time point).
Severity_ep_mean	Mean severity measured at post-treatment or at its closest time point.
Severity_ep_sd	Standard deviation of the above.
Severity_ep_n	The number of patients measured their severity at post-treatment or at its closest time point.
Severity_scale	The scale used for measuring the sleep-related severity. Prioritize; ISI > SCI> FOSQ > ESS > PSQI > AIS > other_self_reported_scales > WASO_SOL > WASO > SOL. We used the definition stated by the author.
dropout	The number of patients dropped out from the study for any reason (=the number of patients whose outcome data not available). Those who are lost to follow-up (assessment at post-treatment) were assumed to have dropped off.
SE_mean	Mean sleep efficiency in %.
SE_sd	Standard deviation of the above.
SE_n	The number of patients measured for the numbers above.
TST_mean	Mean total sleep time in minutes.
TST_sd	Standard deviation of the above.
TST_n	The number of patients measured for the numbers above.
SOL_mean	Mean sleep onset latency in minutes.
SOL_sd	Standard deviation of the above.
SOL_n	The number of patients measured for the numbers above.
WASO_mean	Mean wake after sleep onset in minutes.
WASO_sd	Standard deviation of the above.
WASO_n	The number of patients measured for the numbers above.
Sleep_diary_weeks	Timepoint when the sleep diary was measured
r_long	The number of patients achieving remission at long-term follow-up (longest follow-up between 3 to 12 months)
r_long_scale_used	Scales that were used for counting remission at long-term.
Severity_mean_long	Mean severity at long-term.
Severity_sd_long	Standard deviation of the above.
Severity_n_long	The number of patients measured for the numbers above.

© 2024 Furukawa Y et al. *JAMA Psychiatry.*

variable	description
Severity_ch_mean_long	Extracted this only when Severity_mean_long, Severity _ sd_long and Sevrity_n_long were not available.
Severity_ch_sd_long	Extracted this only when Severity_mean_long, Severity _ sd_long and Sevrity_n_long were not available.
Severity_ch_n_long	Extracted this only when Severity_mean_long, Severity _ sd_long and Sevrity_n_long were not available.
r_weeks_long	Timepoint of the measurement of the long-term follow-up in weeks.

© 2024 Furukawa Y et al. *JAMA Psychiatry.*

1.4. Additive component network meta-analysis model

Assume that trial i compares treatment X (comprising components $c_1 + c_2$) and treatment Y (comprising component $c_3 + c_4$). Then, this study estimates $d_1 + d_2 - d_3 - d_4$, where d_c shows the benefit (or harm) of adding component c to the treatment. Likewise, a study that compares $c_1 + c_2 + c_3$ versus c_3 estimates the combined effect of c_1 and c_2, i.e. $d_1 + d_2$, while the effect of c_3 is inestimable; the effect of c_1 alone and c_2 alone is also not estimable, only their combined effect can be estimated in this study.

In essence, the additive component NMA model assumes that for any given component c, the relative effects of $(c + X)$ vs. X are the same for any combination of components X (as long as this combination X does not include c). Thus, this model assumes that the following trials are estimating the same relative effects: $c_1 + c_2$ versus c_2; $c_1 + c_3$ versus c_3; $c_1 + c_2 + c_3$ versus $c_2 + c_3$; etc. According to the additive cNMA model, all these trials are estimating the effect of adding c_1 to a treatment X not including c_1.

© 2024 Furukawa Y et al. *JAMA Psychiatry.*

eMethods 2. Lists of excluded and included studies

2.1. List of the excluded studies with reasons (examples)

We excluded quasi-randomized trials, in which participants were not fully randomized or allocation was clearly not concealed.

- Sidani, S., Epstein, D. R., Fox, M., & Collins, L. Comparing the Effects of Single- and Multiple-Component Therapies for Insomnia on Sleep Outcomes. *Worldviews Evid.-Based Nurs*. 2019;16(3):195–203.
- Morgan, K., Dixon, S., Mathers, N., Thompson, J., & Tomeny, M. Psychological treatment for insomnia in the regulation of long-term hypnotic drug use. *Health technology assessment*. 2004;8(8):iii–68.
- Ellis JG, Cushing T, Germain A. Treating Acute Insomnia: A Randomized Controlled Trial of a "Single-Shot" of Cognitive Behavioral Therapy for Insomnia. *Sleep*. 2015;38(6):971-978.

We excluded trials when 1) the participants were clearly 17 years old or younger, or 2) some of the participants were 18 years old or older but the trial focused on adolescents. We included trials when some of the participants were 17 years old or younger but the majority of participants were 18 years old or older (mean – 2*SD >18).

- Hiscock H, Sciberras E, Mensah F, et al. Impact of a behavioural sleep intervention on symptoms and sleep in children with attention deficit hyperactivity disorder, and parental mental health: randomised controlled trial. *BMJ*. 2015;350:h68.
- Rogers VE, Zhu S, Ancoli-Israel S, Liu L, Mandrell BN, Hinds PS. A pilot randomized controlled trial to improve sleep and fatigue in children with central nervous system tumors hospitalized for high-dose chemotherapy. *Pediatr Blood Cancer*. 2019;66(8):e27814.

We included trials on patients with chronic insomnia disorder. We excluded trials when the eligibility criteria did not include significant distress or impairment in daytime functioning. (e.g. stating only “sleep problem” or “sleep disturbance”, even when they additionally used elevated scores on self-reported scales, or defining insomnia solely on sleep latency or wake time after sleep onset).

- Black, D. S., O'Reilly, G. A., Olmstead, R., Breen, E. C., & Irwin, M. R. Mindfulness meditation and improvement in sleep quality and daytime impairment among older adults with sleep disturbances: a randomized clinical trial. *JAMA internal medicine*. 2015;175(4):494–501.
- Malfliet, A., Bilterys, T., Van Looveren, E., Meeus, M., Danneels, L., Ickmans, K., Cagnie, B., Mairesse, O., Neu, D., Moens, M., Goubert, D., Kamper, S. J., & Nijs, J. The added value of cognitive behavioral therapy for insomnia to current best evidence physical therapy for chronic spinal pain: protocol of a randomized controlled clinical trial. *Brazilian journal of physical therapy*. 2019;23(1):62–70.

We included trials that used elevated scores on ISI for finding probable chronic insomnia disorder. Consequently, the minimum duration of the symptoms was 2 weeks and trials on patients suffering from sleep problems for less than 2 weeks were excluded.

© 2024 Furukawa Y et al. *JAMA Psychiatry.*

- Wang LN, Tao H, Zhao Y, Zhou YQ, Jiang XR. Optimal timing for initiation of biofeedback-assisted relaxation training in hospitalized coronary heart disease patients with sleep disturbances. *J Cardiovasc Nurs*. 2014;29(4):367-376.

We were interested in dismantling the effects of each component of CBT-I and therefore excluded trials when they included active components focusing on other symptoms and those components were not equally distributed among arms.

- Thiart H, Lehr D, Ebert DD, Sieland B, Berking M, Riper H. Log in and breathe out: efficacy and cost-effectiveness of an online sleep training for teachers affected by work-related strain--study protocol for a randomized controlled trial. *Trials*. 2013;14:169.

We focused on the effects of CBT-I for improving insomnia symptoms, so we excluded trials that aimed at tapering hypnotics.

- Baillargeon L, Landreville P, Verreault R, Beauchemin JP, Grégoire JP, Morin CM. Discontinuation of benzodiazepines among older insomniac adults treated with cognitive-behavioural therapy combined with gradual tapering: a randomized trial. *CMAJ*. 2003;169(10):1015-1020.

© 2024 Furukawa Y et al. *JAMA Psychiatry.*

2.2. List of the ongoing trials and the terminated/withdrawn trials

2.2.1. List of the ongoing trials

Ongoing trials

ACTRN12622000067774, ACTRN12622000158763, ACTRN12622000778785, ACTRN12622000940774, ACTRN12622001218785, ACTRN12623000092695, ChiCTR2200055342, IRCT20190304042912N3, ISRCTN10037794, ISRCTN10144646, ISRCTN16185698, ISRCTN91221906, KCT0007290, KCT0007292, NCT02272712, NCT03109210, NCT03208049, NCT03261674, NCT03322774, NCT03327519, NCT03603717, NCT03632889, NCT03727438, NCT03744156, NCT03804788, NCT03954210, NCT04024787, NCT04026048, NCT04100057, NCT04130529, NCT04136054, NCT04144231, NCT04252638, NCT04300218, NCT04308499, NCT04317742, NCT04350333, NCT04377009, NCT04445805, NCT04457674, NCT04463498, NCT04468776, NCT04498754, NCT04581603, NCT04621643, NCT04635085, NCT04646200, NCT04719143, NCT04721067, NCT04751851, NCT04806009, NCT04829539, NCT04831385, NCT04866914, NCT04876001, NCT04885205, NCT04898855, NCT04909229, NCT04949360, NCT04951466, NCT04975776, NCT04986007, NCT05000528, NCT05011929, NCT05015803, NCT05034159, NCT05065242, NCT05067569, NCT05069285, NCT05133908, NCT05136638, NCT05182372, NCT05194930, NCT05226078, NCT05226585, NCT05233800, NCT05244889, NCT05294991, NCT05310136, NCT05353296, NCT05356364, NCT05373537, NCT05387473, NCT05387473, NCT05402761, NCT05406414, NCT05456607, NCT05490550, NCT05516277, NCT05519982, NCT05541055, NCT05548907, NCT05551806, NCT05555108, NCT05558475, NCT05561829, NCT05565833, NCT05568381, NCT05572697, NCT05576090, NCT05582759, NCT05596318, NCT05604820, NCT05663034, NCT05683132, NCT05683145, NCT05710991, NCT05724498, NCT05747963, NCT05759065, NCT05769231, NCT05778812, NCT05780177, RBR-10b889rz

Recruitment completed, but results not yet available

ChiCTR2200058023, IRCT20220528055007N1, ISRCTN13837516, ISRCTN27620037, ISRCTN36198096, ISRCTN43900695, ISRCTN58986139, ISRCTN59953554, ISRCTN63989489, ISRCTN70652461, JPRN-UMIN000023999, KCT0007823, NCT00255905, NCT00303342, NCT00321451, NCT00540254, NCT00620789, NCT01019187, NCT01489982, NCT01704352, NCT01764035, NCT01794624, NCT01804907, NCT02092844, NCT02117388, NCT02259101, NCT02299193, NCT02508129, NCT02535923, NCT02658669, NCT02709980, NCT02720458, NCT02741336, NCT02743338, NCT02774642, NCT03041389, NCT03075683, NCT03177967, NCT03213132, NCT03302455, NCT03365024, NCT03366870, NCT03390114, NCT03438331, NCT03493958, NCT03534284, NCT03538574, NCT03826849, NCT04016428, NCT04147052, NCT04345068, NCT04409743, NCT04564807, NCT04566822, NCT05033418, NCT05087121, NCT05115604, NCT05416346, NCT05561790

Unknown status

© 2024 Furukawa Y et al. *JAMA Psychiatry.*

ACTRN12607000143426, ACTRN12618000359235, ACTRN12619001166167, ACTRN12620000394943, ACTRN12620001075976, ACTRN12621001395820, ChiCTR-INR-16008930, ChiCTR-TRC-12002535, ChiCTR1800015570, ChiCTR2000032960, ChiCTR2000036936, CTRI/2021/08/035427, CTRI/2021/09/036922, DRKS00010422, DRKS00021503, DRKS00025713, DRKS00026096, DRKS00026613, DRKS00026770, IRCT201206011483N3, IRCT2015091223955N2, IRCT2017061834608N2, IRCT20180131038582N1, IRCT20190417043300N2, IRCT20210714051894N1, ISRCTN10185189, ISRCTN13596153, ISRCTN17117237, JPRN-UMIN000007626, JPRN-UMIN000013401, JPRN-UMIN000044016, NCT00134108, NCT00170391, NCT00672217, NCT02773693, NCT03267537, NCT03268629, NCT03309527, NCT03461666, NCT03542604, NCT03560843, NCT03918057, NCT04013321, NCT04134949, NCT04242771, NCT04306835, NCT04585282, NCT04653155, NL7943, NTR5142, NTR7419

2.2.2. List of the terminated/withdrawn trials

Terminated

NCT01928173, NCT02102230, NCT02779023, NCT03250468, NCT03337061, NCT04059302, NCT05137457

© 2024 Furukawa Y et al. *JAMA Psychiatry.*

2.3. List of the included trials

Abbasi2016

- Abbasi S Ms, Alimohammadi N PhD, Pahlavanzadeh S Ms. Effectiveness of Cognitive Behavioral Therapy on the Quality of Sleep in Women with Multiple Sclerosis: A Randomized Controlled Trial Study. *Int J Community Based Nurs Midwifery*. 2016;4(4):320-328.
- IRCT2015012720833N1

Abdelaziz2021

- Abdelaziz EM, Elsharkawy NB, Mohamed SM. Efficacy of Internet-based cognitive behavioral therapy on sleeping difficulties in menopausal women: A randomized controlled trial. *Perspect Psychiatr Care*. 2021;10.1111/ppc.13005.
- NCT04719598

Ahorsu2020

- Ahorsu DK, Lin CY, Imani V, et al. Testing an app-based intervention to improve insomnia in patients with epilepsy: A randomized controlled trial. *Epilepsy Behav*. 2020;112:107371.
- NCT03683381

Alessi2016

- Alessi C, Martin JL, Fiorentino L, et al. Cognitive Behavioral Therapy for Insomnia in Older Veterans Using Nonclinician Sleep Coaches: Randomized Controlled Trial. *J Am Geriatr Soc*. 2016;64(9):1830-1838.
- Fung CH, Martin JL, Josephson K, et al. Efficacy of Cognitive Behavioral Therapy for Insomnia in Older Adults With Occult Sleep-Disordered Breathing. *Psychosom Med*. 2016;78(5):629-639.
- Yeung T, Martin JL, Fung CH, et al. Sleep Outcomes With Cognitive Behavioral Therapy for Insomnia Are Similar Between Older Adults With Low vs. High Self-Reported Physical Activity. *Front Aging Neurosci*. 2018;10:274.
- Fung CH, Martin JL, Josephson K, et al. Cognitive Expectancies for Hypnotic Use among Older Adult Veterans with Chronic Insomnia. *Clin Gerontol*. 2018;41(2):130-135.
- Dzierzewski JM, Martin JL, Fung CH, et al. CBT for late-life insomnia and the accuracy of sleep and wake perceptions: Results from a randomized-controlled trial. *J Sleep Res*. 2019;28(4):e12809.
- Fung CH, Martin JL, Josephson K, et al. Cognitive Expectancies for Hypnotic Use among Older Adult Veterans with Chronic Insomnia. *Clin Gerontol*. 2018;41(2):130-135.
- NCT00781963

Alshehri2020

© 2024 Furukawa Y et al. *JAMA Psychiatry.*

- Alshehri MM, Alenazi AM, Alothman SA, et al. Using Cognitive Behavioral Therapy for Insomnia in People with Type 2 Diabetes, Pilot RCT Part I: Sleep and Concomitant Symptom. *Behav Sleep Med.* 2021;19(5):652-671.
- Alshehri MM, Alothman SA, Alenazi AM, et al. The effects of cognitive behavioral therapy for insomnia in people with type 2 diabetes mellitus, pilot RCT part II: diabetes health outcomes. *BMC Endocr Disord*. 2020;20(1):136.
- Alshehri MM, Alenazi AM, Hoover JC, et al. Effect of Cognitive Behavioral Therapy for Insomnia on Insomnia Symptoms for Individuals With Type 2 Diabetes: Protocol for a Pilot Randomized Controlled Trial. *JMIR Res Protoc*. 2019;8(12):e14647.
- NCT03713996

Amra2022

- Amra B, Ghadiry F, Vaezi A, et al. Effect of one-shot cognitive behavioral therapy on insomnia and heart rate variability of health care workers at the time of COVID-19 pandemic: a randomized controlled trial. *Sleep Breath*. 2022;1-8.
- IRCT20171219037964N4

Arnedt2011

- Arnedt JT, Conroy DA, Armitage R, Brower KJ. Cognitive-behavioral therapy for insomnia in alcohol dependent patients: a randomized controlled pilot trial. *Behav Res Ther*. 2011;49(4):227-233.

Arnedt2021

- Arnedt JT, Conroy DA, Mooney A, Furgal A, Sen A, Eisenberg D. Telemedicine versus face-to-face delivery of cognitive behavioral therapy for insomnia: a randomized controlled noninferiority trial. *Sleep*. 2021;44(1):zsaa136.
- NCT03293745

Arnedt2023

- Arnedt JT, Conroy DA, Stewart H, et al. Cognitive behavioral therapy for insomnia to reduce cannabis use: Results from a pilot randomized controlled trial. *Drug Alcohol Depend.* 2023;246:109835. doi:10.1016/j.drugalcdep.2023.109835

Ashworth2015

- Ashworth DK, Sletten TL, Junge M, et al. A randomized controlled trial of cognitive behavioral therapy for insomnia: an effective treatment for comorbid insomnia and depression. *J Couns Psychol*. 2015;62(2):115-123.

Ayabe2018

© 2024 Furukawa Y et al. *JAMA Psychiatry.*

- Ayabe N, Okajima I, Nakajima S, et al. Effectiveness of cognitive behavioral therapy for pharmacotherapy-resistant chronic insomnia: a multi-center randomized controlled trial in Japan. *Sleep Med*. 2018;50:105-112.
- JPRN-UMIN000014297
- One of the co-authors confirmed that waiting-list component was present in the control group.

Ballou2020

- Ballou S, Katon J, Rangan V, et al. Brief Behavioral Therapy for Insomnia in Patients with Irritable Bowel Syndrome: A Pilot Study. *Dig Dis Sci*. 2020;65(11):3260-3270.

Bastein2004

- Bastien CH, Morin CM, Ouellet MC, Blais FC, Bouchard S. Cognitive-behavioral therapy for insomnia: comparison of individual therapy, group therapy, and telephone consultations. *J Consult Clin Psychol.* 2004;72(4):653-659.

Behera2023

- Behera CK, Reddy TK, Behera L, Birbaumer N, Ika K. A Meditation Based Cognitive Therapy (HMBCT) for Primary Insomnia: A Treatment Feasibility Pilot Study [published online ahead of print, 2023 Apr 27]. *Appl Psychophysiol Biofeedback.* 2023;10.1007/s10484-023-09586-2. doi:10.1007/s10484-023-09586-2

Bernstein2017

- Bernstein AM, Allexandre D, Bena J, et al. "Go! to Sleep": A Web-Based Therapy for Insomnia. *Telemed J E Health*. 2017;23(7):590-599.
- NCT01440777

Bothelius2013

- Bothelius K, Kyhle K, Espie CA, Broman JE. Manual-guided cognitive-behavioural therapy for insomnia delivered by ordinary primary care personnel in general medical practice: a randomized controlled effectiveness trial. *J Sleep Res*. 2013;22(6):688-696.
- NCT01655797

Bramoweth2020

- Bramoweth AD, Lederer LG, Youk AO, Germain A, Chinman MJ. Brief Behavioral Treatment for Insomnia vs. Cognitive Behavioral Therapy for Insomnia: Results of a Randomized Noninferiority Clinical Trial Among Veterans. *Behav Ther*. 2020;51(4):535-547.
- Bramoweth AD, Germain A, Youk AO, Rodriguez KL, Chinman MJ. A hybrid type I trial to increase Veterans' access to insomnia care: study protocol for a randomized controlled trial. *Trials*. 2018;19(1):73.
- NCT02724800

© 2024 Furukawa Y et al. *JAMA Psychiatry.*

Broomfield2003

- Broomfield NM, Espie CA. Initial Insomnia And Paradoxical Intention: An Experimental Investigation Of Putative Mechanisms Using Subjective And Actigraphic Measurement Of Sleep. *Behavioural and Cognitive Psychotherapy*. 2003;31(3):313-324.

Buysse2011

- Buysse DJ, Germain A, Moul DE, et al. Efficacy of brief behavioral treatment for chronic insomnia in older adults [published correction appears in JAMA Intern Med. 2019 Aug 1;179(8):1152]. *Arch Intern Med*. 2011;171(10):887-895.
- Errors in Table Headings. *JAMA Intern Med*. 2019;179(8):1152. doi:10.1001/jamainternmed.2019.1927
- Germain A, Moul DE, Franzen PL, et al. Effects of a brief behavioral treatment for late-life insomnia: preliminary findings. *J Clin Sleep Med*. 2006;2(4):403-406.
- Troxel WM, Conrad TS, Germain A, Buysse DJ. Predictors of treatment response to brief behavioral treatment of insomnia (BBTI) in older adults. *J Clin Sleep Med*. 2013;9(12):1281-1289.
- Tyagi S, Resnick NM, Perera S, Monk TH, Hall MH, Buysse DJ. Behavioral treatment of insomnia: also effective for nocturia. *J Am Geriatr Soc*. 2014;62(1):54-60.
- Tyagi S, Resnick NM, Perera S, Monk TH, Hall MH, Buysse DJ. Behavioral treatment of chronic insomnia in older adults: does nocturia matter?. *Sleep*. 2014;37(4):681-687.
- Wilckens KA, Hall MH, Nebes RD, Monk TH, Buysse DJ. Changes in Cognitive Performance Are Associated with Changes in Sleep in Older Adults With Insomnia. *Behav Sleep Med.* 2016;14(3):295-310.
- NCT00177203

Cai2023

- Cai ZZ, Lin R, Wang XX, Yan YJ, Li H. Effects of mindfulness in patients with mild cognitive impairment with insomnia: A double-blind randomized controlled trial. *Geriatr Nurs.* 2022;47:239-246. doi:10.1016/j.gerinurse.2022.08.001

Cape2016

- Cape J, Leibowitz J, Whittington C, Espie CA, Pilling S. Group cognitive behavioural treatment for insomnia in primary care: a randomized controlled trial. *Psychol Med.* 2016;46(5):1015-1025.
- ISRCTN17064995

Carney2017

- Carney CE, Edinger JD, Kuchibhatla M, et al. Cognitive Behavioral Insomnia Therapy for Those With Insomnia and Depression: A Randomized Controlled Clinical Trial. *Sleep*. 2017;40(4):zsx019.
- NCT0062079

Carrera1980

- Carrera RN, Elenewski JJ. Implosive therapy as a treatment for insomnia. *J Clin Psychol.* 1980;36(3):729-734.

Casault2015

- Casault L, Savard J, Ivers H, Savard MH. A randomized-controlled trial of an early minimal cognitive-behavioural therapy for insomnia comorbid with cancer. *Behav Res Ther*. 2015;67:45-54.

Chakravorty2019

- Chakravorty S, Morales KH, Arnedt JT, et al. Cognitive Behavioral Therapy for Insomnia in Alcohol-Dependent Veterans: A Randomized, Controlled Pilot Study. *Alcohol Clin Exp Res*. 2019;43(6):1244-1253.
- NCT01603381

Chan2021

- Chan CS, Wong CYF, Yu BYM, Hui VKY, Ho FYY, Cuijpers P. Treating depression with a smartphone-delivered self-help cognitive behavioral therapy for insomnia: a parallel-group randomized controlled trial. *Psychological Medicine*. 2021:1-15.
- Hui VK, Wong CY, Ma EK, Ho FY, Chan CS. Treating depression with a smartphone-delivered self-help cognitive behavioral therapy for insomnia: study protocol for a parallel group randomized controlled trial. *Trials*. 2020;21(1):843.
- NCT04228146

Chao2021

- Chao LL, Kanady JC, Crocker N, et al. Cognitive behavioral therapy for insomnia in veterans with gulf war illness: Results from a randomized controlled trial. *Life Sci*. 2021;279:119147.
- NCT02782780

Chapoutot2020

- Chapoutot M, Peter-Derex L, Schoendorff B, Faivre T, Bastuji H, Putois B. Telehealth-delivered CBT-I programme enhanced by acceptance and commitment therapy for insomnia and hypnotic dependence: A pilot randomized controlled trial. *J Sleep Res*. 2021;30(1):e13199.

Cheng2019

- Cheng P, Kalmbach DA, Tallent G, Joseph CL, Espie CA, Drake CL. Depression prevention via digital cognitive behavioral therapy for insomnia: a randomized controlled trial. *Sleep*. 2019;42(10):zsz150.
- Cheng P, Luik AI, Fellman-Couture C, et al. Efficacy of digital CBT for insomnia to reduce depression across demographic groups: a randomized trial. *Psychol Med*. 2019;49(3):491-500.

© 2024 Furukawa Y et al. *JAMA Psychiatry.*

- Cheng P, Kalmbach DA, Castelan AC, Murugan N, Drake CL. Depression prevention in digital cognitive behavioral therapy for insomnia: Is rumination a mediator?. *J Affect Disord*. 2020;273:434-441.
- Cheng P, Casement MD, Kalmbach DA, Castelan AC, Drake CL. Digital cognitive behavioral therapy for insomnia promotes later health resilience during the coronavirus disease 19 (COVID-19) pandemic. *Sleep*. 2021;44(4):zsaa258.
- Cheng P, Casement MD, Kalmbach DA, Cuamatzi Castelan A, Drake CL. Self-efficacy in Insomnia Symptom Management after Digital CBT-I Mediates Insomnia Severity during the COVID-19 Pandemic. *Behav Sleep Med*. 2022;20(5):638-648.
- Cheng P, Kalmbach DA, Hsieh HF, Castelan AC, Sagong C, Drake CL. Improved resilience following digital cognitive behavioral therapy for insomnia protects against insomnia and depression one year later [published online ahead of print, 2022 Mar 8]. *Psychol Med*. 2022;1-11.
- NCT02988375

Christensen2016

- Christensen H, Batterham PJ, Gosling JA, et al. Effectiveness of an online insomnia program (SHUTi) for prevention of depressive episodes (the GoodNight Study): a randomised controlled trial. *Lancet Psychiatry.* 2016;3(4):333-341.
- Gosling JA, Glozier N, Griffiths K, et al. The GoodNight study--online CBT for insomnia for the indicated prevention of depression: study protocol for a randomised controlled trial. *Trials.* 2014;15:56.
- Batterham PJ, Christensen H, Mackinnon AJ, et al. Trajectories of change and long-term outcomes in a randomised controlled trial of internet-based insomnia treatment to prevent depression. *BJPsych Open*. 2017;3(5):228-235.
- Gosling JA, Batterham P, Ritterband L, et al. Online insomnia treatment and the reduction of anxiety symptoms as a secondary outcome in a randomised controlled trial: The role of cognitive-behavioural factors. *Aust N Z J Psychiatry*. 2018;52(12):1183-1193.
- ACTRN12611000121965

Chun2023

- Chung KF, Lee CT, Au CH, et al. Cognitive behavioural therapy for insomnia as an early intervention of mood disorders with comorbid insomnia: A randomized controlled trial [published online ahead of print, 2023 May 16]. *Early Interv Psychiatry*. 2023;10.1111/eip.13435. doi:10.1111/eip.13435
- NCT03000894

Craner2022

- Craner JR, Flegge LG, Gabelman RB. Treatment of Insomnia within an Interdisciplinary Pain Rehabilitation Program: A Randomized Trial. *Int J Clin Health Psychol*. 2022;22(2):100295.

Currie2000

© 2024 Furukawa Y et al. *JAMA Psychiatry.*

- Currie SR, Wilson KG, Pontefract AJ, deLaplante L. Cognitive-behavioral treatment of insomnia secondary to chronic pain. *J Consult Clin Psychol*. 2000;68(3):407-416.
- Currie, SR. Cognitive-behavioural treatment of insomnia secondary to chronic pain. 2000. Dissertation

Currie2004

- Currie SR, Clark S, Hodgins DC, El-Guebaly N. Randomized controlled trial of brief cognitive-behavioural interventions for insomnia in recovering alcoholics. *Addiction*. 2004;99(9):1121-1132.

Daren2012

- Daren, Andrew Michael. Brief behavioral treatment of insomnia in patients with orofacial pain. 2012. Dissertation

Dean2019

- Dean GE, Weiss C, Jungquist CR, et al. Nurse-Delivered Brief Behavioral Treatment for Insomnia in Lung Cancer Survivors: A Pilot RCT. *Behav Sleep Med.* 2020;18(6):774-786.

Dekker2020

- Dekker K, Benjamins JS, Maksimovic T, et al. Combined Internet-Based Cognitive-Behavioral and Chronobiological Intervention for Insomnia: A Randomized Controlled Trial. *Psychother Psychosom*. 2020;89(2):117-118.
- Dekker K, Benjamins JS, Van Straten A, Hofman WF, Van Someren EJ. Effectiveness of internet-supported cognitive behavioral and chronobiological interventions and effect moderation by insomnia subtype: study protocol of a randomized controlled trial. *Trials*. 2015;16:292.
- NTR4010

Dirksen2008

- Dirksen SR, Epstein DR. Efficacy of an insomnia intervention on fatigue, mood and quality of life in breast cancer survivors. *J Adv Nurs*. 2008;61(6):664-675.

Drake2019

- Drake CL, Kalmbach DA, Arnedt JT, et al. Treating chronic insomnia in postmenopausal women: a randomized clinical trial comparing cognitive-behavioral therapy for insomnia, sleep restriction therapy, and sleep hygiene education. *Sleep*. 2019;42(2):zsy217.
- Kalmbach DA, Cheng P, Arnedt JT, et al. Treating insomnia improves depression, maladaptive thinking, and hyperarousal in postmenopausal women: comparing cognitive-behavioral therapy for insomnia (CBTI), sleep restriction therapy, and sleep hygiene education. *Sleep Med*. 2019;55:124-134.

© 2024 Furukawa Y et al. *JAMA Psychiatry.*

- Kalmbach DA, Cheng P, Arnedt JT, et al. Improving Daytime Functioning, Work Performance, and Quality of Life in Postmenopausal Women With Insomnia: Comparing Cognitive Behavioral Therapy for Insomnia, Sleep Restriction Therapy, and Sleep Hygiene Education. *J Clin Sleep Med*. 2019;15(7):999-1010.
- Kalmbach DA, Cheng P, Roth T, Sagong C, Drake CL. Objective sleep disturbance is associated with poor response to cognitive and behavioral treatments for insomnia in postmenopausal women. *Sleep Med*. 2020;73:82-92.
- Cheng P, Kalmbach D, Fellman-Couture C, Arnedt JT, Cuamatzi-Castelan A, Drake CL. Risk of excessive sleepiness in sleep restriction therapy and cognitive behavioral therapy for insomnia: a randomized controlled trial. *J Clin Sleep Med*. 2020;16(2):193-198.
- NCT01933295

Dyrberg2022
- Dyrberg H, Bjorvatn B, Larsen ER. Cognitive Behavioral Therapy for Chronic Insomnia in Outpatients with Major Depression-A Randomised Controlled Trial. *J Clin Med*. 2022;11(19):5845.
- NCT02678702

Edinger2001
- Edinger JD, Wohlgemuth WK, Radtke RA, Marsh GR, Quillian RE. Cognitive behavioral therapy for treatment of chronic primary insomnia: a randomized controlled trial. *JAMA*. 2001;285(14):1856-1864.
- Edinger JD, Wohlgemuth WK, Radtke RA, Marsh GR, Quillian RE. Does cognitive-behavioral insomnia therapy alter dysfunctional beliefs about sleep? *Sleep.* 2001;24(5):591-599.
- Krystal AD, Edinger JD. Sleep EEG predictors and correlates of the response to cognitive behavioral therapy for insomnia. *Sleep*. 2010;33(5):669-677.
- Edinger JD, Carney CE, Wohlgemuth WK. Pretherapy cognitive dispositions and treatment outcome in cognitive behavior therapy for insomnia. *Behav Ther.* 2008;39(4):406-416.

Edinger2003
- Edinger JD, Sampson WS. A primary care "friendly" cognitive behavioral insomnia therapy. *Sleep*. 2003;26(2):177-182.

Edinger2005
- Edinger JD, Wohlgemuth WK, Krystal AD, Rice JR. Behavioral insomnia therapy for fibromyalgia patients: a randomized clinical trial. *Arch Intern Med*. 2005;165(21):2527-2535.
- NCT00000397

Edinger2007
- Edinger JD, Wohlgemuth WK, Radtke RA, Coffman CJ, Carney CE. Dose-response effects of cognitive-behavioral insomnia therapy: a randomized clinical trial. *Sleep*. 2007;30(2):203-212.

© 2024 Furukawa Y et al. *JAMA Psychiatry.*

- Edinger JD, Carney CE, Wohlgemuth WK. Pretherapy cognitive dispositions and treatment outcome in cognitive behavior therapy for insomnia. *Behav Ther*. 2008;39(4):406-416.
- Bathgate CJ, Edinger JD, Krystal AD. Insomnia Patients With Objective Short Sleep Duration Have a Blunted Response to Cognitive Behavioral Therapy for Insomnia. *Sleep.* 2017;40(1):zsw012.

Edinger2009

- Edinger JD, Olsen MK, Stechuchak KM, et al. Cognitive behavioral therapy for patients with primary insomnia or insomnia associated predominantly with mixed psychiatric disorders: a randomized clinical trial. *Sleep*. 2009;32(4):499-510.
- NCT00105872

ElRafihiFerreira2021

- El Rafihi-Ferreira R, Morin CM, Toscanini AC, et al. Acceptance and commitment therapy-based behavioral intervention for insomnia: a pilot randomized controlled trial. *Braz J Psychiatry*. 2021;43(5):504-509.
- RBR-7nc5wq

ElRafihiFerreira2022

- El Rafihi-Ferreira R, Morin CM, Hasan R, Brasil IS, Zago Ribeiro JH Júnior, Cecília Toscanini A. A Pilot Randomized Controlled Trial (RCT) of Acceptance and Commitment Therapy Versus Cognitive Behavioral Therapy for Chronic Insomnia [published online ahead of print, 2022 May 10]. *Behav Sleep Med*. 2022;1-15.
- RBR-7nc5wq

Epstein2007

- Epstein DR, Dirksen SR. Randomized trial of a cognitive-behavioral intervention for insomnia in breast cancer survivors. *Oncol Nurs Forum*. 2007;34(5):E51-E59.

Epstein2012

- Epstein DR, Sidani S, Bootzin RR, Belyea MJ. Dismantling multicomponent behavioral treatment for insomnia in older adults: a randomized controlled trial. *Sleep*. 2012;35(6):797-805.
- NCT01154023

Espie1989

- Espie CA, Brooks DN, Lindsay WR. An evaluation of tailored psychological treatment of insomnia. *J Behav Ther Exp Psychiatry*. 1989;20(2):143-153.
- Espie, CA. A controlled comparative investigation of psychological treatments for chronic sleep-onset insomnia. 1987. Dissertation

© 2024 Furukawa Y et al. *JAMA Psychiatry.*

Espie2001

- Espie CA, Inglis SJ, Tessier S, Harvey L. The clinical effectiveness of cognitive behaviour therapy for chronic insomnia: implementation and evaluation of a sleep clinic in general medical practice. *Behav Res Ther*. 2001;39(1):45-60.

Espie2007

- Espie CA, MacMahon KM, Kelly HL, et al. Randomized clinical effectiveness trial of nurse-administered small-group cognitive behavior therapy for persistent insomnia in general practice. *Sleep*. 2007;30(5):574-584.
- NCT00170417

Espie2008

- Espie CA, Fleming L, Cassidy J, et al. Randomized controlled clinical effectiveness trial of cognitive behavior therapy compared with treatment as usual for persistent insomnia in patients with cancer. *J Clin Oncol*. 2008;26(28):4651-4658.
- Fleming L, Randell K, Harvey CJ, Espie CA. Does cognitive behaviour therapy for insomnia reduce clinical levels of fatigue, anxiety and depression in cancer patients? *Psychooncology.* 2014;23(6):679-684.

Espie2012

- Espie CA, Kyle SD, Williams C, et al. A randomized, placebo-controlled trial of online cognitive behavioral therapy for chronic insomnia disorder delivered via an automated media-rich web application. *Sleep*. 2012;35(6):769-781.
- Espie CA, Kyle SD, Miller CB, Ong J, Hames P, Fleming L. Attribution, cognition and psychopathology in persistent insomnia disorder: outcome and mediation analysis from a randomized placebo-controlled trial of online cognitive behavioural therapy. *Sleep Med*. 2014;15(8):913-917.
- Henry AL, Miller CB, Emsley R, et al. Insomnia as a mediating therapeutic target for depressive symptoms: A sub-analysis of participant data from two large randomized controlled trials of a digital sleep intervention. *J Sleep Res*. 2021;30(1):e13140.
- ISRCTN44615689

Espie2019

- Espie CA, Emsley R, Kyle SD, et al. Effect of Digital Cognitive Behavioral Therapy for Insomnia on Health, Psychological Well-being, and Sleep-Related Quality of Life: A Randomized Clinical Trial. *JAMA Psychiatry*. 2019;76(1):21-30.
- Luik AI, Marsden A, Emsley R, et al. Long-term benefits of digital cognitive behavioural therapy for insomnia: Follow-up report from a randomized clinical trial. *J Sleep Res*. 2020;29(4):e13018.

© 2024 Furukawa Y et al. *JAMA Psychiatry.*

- Song Y, Kelly MR, Fung CH, et al. Change in Dysfunctional Sleep-Related Beliefs is Associated with Changes in Sleep and Other Health Outcomes Among Older Veterans With Insomnia: Findings From a Randomized Controlled Trial. *Ann Behav Med*. 2022;56(1):35-49.
- ISRCTN60530898

Falloon2015

- Falloon K, Elley CR, Fernando A 3rd, Lee AC, Arroll B. Simplified sleep restriction for insomnia in general practice: a randomised controlled trial. *Br J Gen Pract*. 2015;65(637):e508-e515.
- ACTRN12609000127202

Farsani2021

- Moradi Farsani H, Afshari P, Sadeghniiat Haghighi K, Gholamzadeh Jefreh M, Abedi P, Haghighizadeh MH. The effect of group cognitive behavioural therapy for insomnia in postmenopausal women. *J Sleep Res.* 2021;30(5):e13345.
- IRCT20180918041065N1

Felder2020

- Felder JN, Epel ES, Neuhaus J, Krystal AD, Prather AA. Efficacy of Digital Cognitive Behavioral Therapy for the Treatment of Insomnia Symptoms Among Pregnant Women: A Randomized Clinical Trial [published correction appears in JAMA Psychiatry. 2020 Jul 1;77(7):768]. *JAMA Psychiatry*. 2020;77(5):484-492.
- Errors in Conflict of Interest Disclosures. JAMA Psychiatry. 2020;77(7):768.
- Felder JN, Epel ES, Neuhaus J, Krystal AD, Prather AA. Randomized controlled trial of digital cognitive behavior therapy for prenatal insomnia symptoms: effects on postpartum insomnia and mental health. *Sleep.* 2022;45(2):zsab280.
- NCT02805998

Fernando2013

- Fernando A 3rd, Arroll B, Falloon K. A double-blind randomised controlled study of a brief intervention of bedtime restriction for adult patients with primary insomnia. *J Prim Health Care*. 2013;5(1):5-10.

Feuerstein2017

- Feuerstein S, Hodges SE, Keenaghan B, Bessette A, Forselius E, Morgan PT. Computerized Cognitive Behavioral Therapy for Insomnia in a Community Health Setting. *J Clin Sleep Med*. 2017;13(2):267-274.

Ford2022

© 2024 Furukawa Y et al. *JAMA Psychiatry.*

- Ford ME, Geurtsen GJ, Groet E, Rambaran Mishre RD, Van Bennekom CAM, Van Someren EJW. A blended eHealth intervention for insomnia following acquired brain injury: a randomised controlled trial. *J Sleep Res*. 2022;e13629.
- Ford ME, Geurtsen GJ, Groet E, Van Bennekom CAM, Van Someren EJW. A blended eHealth intervention for insomnia following acquired brain injury: study protocol for a randomized controlled trial. *Trials*. 2020;21(1):861.
- NL6895; NTR7082

Fowler2009

- Fowler, Margaret S. Treatment of late-life insomnia in female participants living in a rural community using a multi-component self-help intervention. 2009. *Dissertation*.

Freeman2015

- Freeman D, Waite F, Startup H, et al. Efficacy of cognitive behavioural therapy for sleep improvement in patients with persistent delusions and hallucinations (BEST): a prospective, assessor-blind, randomised controlled pilot trial. *Lancet Psychiatry.* 2015;2(11):975-983.
- Freeman D, Startup H, Myers E, et al. The effects of using cognitive behavioural therapy to improve sleep for patients with delusions and hallucinations (the BEST study): study protocol for a randomized controlled trial. *Trials*. 2013;14:214.
- Tsiachristas A, Waite F, Freeman D, Luengo-Fernandez R. Cost-effectiveness of cognitive-behavioural therapy for sleep disorder added to usual care in patients with schizophrenia: the BEST study. *BJPsych Open*. 2018;4(3):126-135.
- ISRCTN 33695128

Freeman2017

- Freeman D, Sheaves B, Goodwin GM, et al. The effects of improving sleep on mental health (OASIS): a randomised controlled trial with mediation analysis. *Lancet Psychiatry*. 2017;4(10):749-758.
- Freeman D, Sheaves B, Goodwin GM, et al. Effects of cognitive behavioural therapy for insomnia on the mental health of university students: study protocol for a randomized controlled trial. *Trials*. 2015;16:236.
- Henry AL, Miller CB, Emsley R, et al. Insomnia as a mediating therapeutic target for depressive symptoms: A sub-analysis of participant data from two large randomized controlled trials of a digital sleep intervention. *J Sleep Res*. 2021;30(1):e13140.
- Henry AL, Miller CB, Emsley R, et al. Insomnia as a mediating therapeutic target for depressive symptoms: A sub-analysis of participant data from two large randomized controlled trials of a digital sleep intervention. *J Sleep Res*. 2021;30(1):e13140.
- ISRCTN61272551

Fucito2014

© 2024 Furukawa Y et al. *JAMA Psychiatry.*

- Fucito LM, Redeker NS, Ball SA, Toll BA, Ikomi JT, Carroll KM. Integrating a Behavioural Sleep Intervention into Smoking Cessation Treatment for Smokers with Insomnia: A Randomised Pilot Study. *J Smok Cessat*. 2014;9(1):31-38.

Garcia2018

- Garcia MC, Kozasa EH, Tufik S, Mello LEAM, Hachul H. The effects of mindfulness and relaxation training for insomnia (MRTI) on postmenopausal women: a pilot study. *Menopause*. 2018;25(9):992-1003.
- NCT01593436

Garland2014

- Garland SN, Carlson LE, Stephens AJ, Antle MC, Samuels C, Campbell TS. Mindfulness-based stress reduction compared with cognitive behavioral therapy for the treatment of insomnia comorbid with cancer: a randomized, partially blinded, noninferiority trial. *J Clin Oncol*. 2014;32(5):449-457.
- Garland SN, Rouleau CR, Campbell T, Samuels C, Carlson LE. The Comparative Impact of Mindfulness-Based Cancer Recovery (MBCR) and Cognitive Behavior Therapy for Insomnia (CBT-I) on Sleep and Mindfulness in Cancer Patients. *Explore (NY)*. 2015;11(6):445-454.
- NCT01335776

Gebara2019

- Gebara MA, DiNapoli EA, Lederer LG, et al. Brief behavioral treatment for insomnia in older adults with late-life treatment-resistant depression and insomnia: a pilot study. *Sleep Biol Rhythms*. 2019;17(3):287-295.

Gehrman2020

- Gehrman P, Barilla H, Medvedeva E, et al. Randomized trial of telehealth delivery of cognitive-behavioral treatment for insomnia vs. in-person treatment in veterans with PTSD. *J Affect Disord.* 2020;1:100018.
- NCT01686438

Gehrman2021

- Gehrman P, Gunter P, Findley J, et al. Randomized Noninferiority Trial of Telehealth Delivery of Cognitive Behavioral Treatment of Insomnia Compared to In-Person Care. *J Clin Psychiatry*. 2021;82(5):20m13723.
- NCT03328585

Germain2014

- Germain A, Richardson R, Stocker R, et al. Treatment for insomnia in combat-exposed OEF/OIF/OND military veterans: preliminary randomized controlled trial. *Behav Res Ther*. 2014;61:78-88.
- NCT00840255

© 2024 Furukawa Y et al. *JAMA Psychiatry.*

Glieselmann2019

- Gieselmann A, Pietrowsky R. The effects of brief chat-based and face-to-face psychotherapy for insomnia: a randomized waiting list controlled trial. *Sleep Med*. 2019;61:63-72.
- DRKS00010522

Glozier2019

- Cockayne NL, Christensen HM, Griffiths KM, et al. The Sleep Or Mood Novel Adjunctive therapy (SOMNA) trial: a study protocol for a randomised controlled trial evaluating an internet-delivered cognitive behavioural therapy program for insomnia on outcomes of standard treatment for depression in men. *BMC Psychiatry*. 2015;15:16.
- Glozier N, Christensen H, Griffiths KM, et al. Adjunctive Internet-delivered cognitive behavioural therapy for insomnia in men with depression: A randomised controlled trial. *Aust N Z J Psychiatry*. 2019;53(4):350-360.
- ACTRN12612000985886

Greeff1998

- Greeff AP, Conradie WS. Use of progressive relaxation training for chronic alcoholics with insomnia. *Psychol Rep*. 1998;82(2):407-412.

Guarnaccia2023

- Guarnaccia JB, Njike VY, Dutton A, et al. A pilot, randomized, placebo-controlled study of mindfulness meditation in treating insomnia in multiple sclerosis. *BMC Neurol*. 2023;23(1):263. Published 2023 Jul 11. doi:10.1186/s12883-023-03309-0
- NCT03949296

Hagatun2019

- Hagatun S, Vedaa Ø, Nordgreen T, et al. The Short-Term Efficacy of an Unguided Internet-Based Cognitive-Behavioral Therapy for Insomnia: A Randomized Controlled Trial With a Six-Month Nonrandomized Follow-Up. *Behav Sleep Med*. 2019;17(2):137-155.
- Hagatun S, Vedaa Ø, Harvey AG, et al. Internet-delivered cognitive-behavioral therapy for insomnia and comorbid symptoms. *Internet Interv*. 2018;12:11-15.
- Lien M, Bredeli E, Sivertsen B, et al. Short and long-term effects of unguided internet-based cognitive behavioral therapy for chronic insomnia in morning and evening persons: a post-hoc analysis. *Chronobiol Int*. 2019;36(10):1384-1398.
- Vedaa Ø, Hagatun S, Kallestad H, et al. Long-Term Effects of an Unguided Online Cognitive Behavioral Therapy for Chronic Insomnia. *J Clin Sleep Med*. 2019;15(1):101-110.
- NCT02261272

© 2024 Furukawa Y et al. *JAMA Psychiatry.*

Hall2022

- Hall DL, Arditte Hall KA, Gorman MJ, et al. The Survivorship Sleep Program (SSP): A synchronous, virtual cognitive behavioral therapy for insomnia pilot program among cancer survivors. *Cancer*. 2022;128(7):1532-1544.
- NCT04566068

Ham2020

- Ham OK, Lee BG, Choi E, Choi SJ. Efficacy of Cognitive Behavioral Treatment for Insomnia: A Randomized Controlled Trial. *West J Nurs Res*. 2020;42(12):1104-1112.

Harris2012

- Harris J, Lack L, Kemp K, Wright H, Bootzin R. A randomized controlled trial of intensive sleep retraining (ISR): a brief conditioning treatment for chronic insomnia. *Sleep*. 2012;35(1):49-60.

Harris2019

- Harris KM, Schiele SE, Emery CF. Pilot randomized trial of brief behavioral treatment for insomnia in patients with heart failure. *Heart Lung*. 2019;48(5):373-380.
- Harris, KM. Insomnia, cognitive impairment, and decision-making among patients with heart failure: A randomized study of brief behavioral treatment for insomnia. Doctoral dissertation, Ohio State University, 2018.
- NCT03636880

Harvey2014

- Harvey AG, Bélanger L, Talbot L, et al. Comparative efficacy of behavior therapy, cognitive therapy, and cognitive behavior therapy for chronic insomnia: a randomized controlled trial. *J Consult Clin Psychol.* 2014;82(4):670-683.
- Eidelman P, Talbot L, Ivers H, Bélanger L, Morin CM, Harvey AG. Change in Dysfunctional Beliefs About Sleep in Behavior Therapy, Cognitive Therapy, and Cognitive-Behavioral Therapy for Insomnia. *Behav Ther*. 2016;47(1):102-115.
- Bélanger L, Harvey AG, Fortier-Brochu É, et al. Impact of comorbid anxiety and depressive disorders on treatment response to cognitive behavior therapy for insomnia. *J Consult Clin Psychol.* 2016;84(8):659-667.
- Harvey AG, Dong L, Bélanger L, Morin CM. Mediators and treatment matching in behavior therapy, cognitive therapy and cognitive behavior therapy for chronic insomnia. *J Consult Clin Psychol.* 2017;85(10):975-987.
- Dong L, Soehner AM, Bélanger L, Morin CM, Harvey AG. Treatment agreement, adherence, and outcome in cognitive behavioral treatments for insomnia. *J Consult Clin Psychol*. 2018;86(3):294-299.

© 2024 Furukawa Y et al. *JAMA Psychiatry.*

- Lancee J, Harvey AG, Morin CM, Ivers H, van der Zweerde T, Blanken TF. Network Intervention Analyses of cognitive therapy and behavior therapy for insomnia: Symptom specific effects and process measures. *Behav Res Ther.* 2022;153:104100.
- NCT00869934

Harvey2015

- Harvey AG, Soehner AM, Kaplan KA, et al. Treating insomnia improves mood state, sleep, and functioning in bipolar disorder: a pilot randomized controlled trial. *J Consult Clin Psychol*. 2015;83(3):564-577.
- Lee JY, Harvey AG. Memory for therapy in bipolar disorder and comorbid insomnia. *J Consult Clin Psychol.* 2015;83(1):92-102.

Ho2014

- Ho FY, Chung KF, Yeung WF, Ng TH, Cheng SK. Weekly brief phone support in self-help cognitive behavioral therapy for insomnia disorder: Relevance to adherence and efficacy. *Behav Res Ther.* 2014;63:147-156.
- Yeung WF, Chung KF, Ho FY, Ho LM. Predictors of dropout from internet-based self-help cognitive behavioral therapy for insomnia. *Behav Res Ther*. 2015;73:19-24.
- Chung KF, Ho FY, Yeung WF. Psychometric Comparison of the Full and Abbreviated Versions of the Dysfunctional Beliefs and Attitudes about Sleep Scale. *J Clin Sleep Med*. 2016;12(6):821-828.
- NCT01719120

Ho2021

- Ho FY, Choi WT, Yeung WF, Lam HK, Lau WY, Chung KF. The efficacy of integrated cognitive behavioral therapy (CBT) and acupressure versus CBT for insomnia: a three-arm pilot randomized controlled trial. *Sleep Med*. 2021;87:158-167.
- NCT03291301

Holmqvist2014

- Holmqvist M, Vincent N, Walsh K. Web- vs. telehealth-based delivery of cognitive behavioral therapy for insomnia: a randomized controlled trial. *Sleep Med*. 2014;15(2):187-195.
- NCT01162655

Horsch2017

- Horsch CH, Lancee J, Griffioen-Both F, et al. Mobile Phone-Delivered Cognitive Behavioral Therapy for Insomnia: A Randomized Waitlist Controlled Trial. *J Med Internet Res*. 2017;19(4):e70.
- NTR5560

© 2024 Furukawa Y et al. *JAMA Psychiatry.*

Hou2014

- Hou Y, Hu P, Liang Y, Mo Z. Effects of cognitive behavioral therapy on insomnia of maintenance hemodialysis patients. *Cell Biochem Biophys*. 2014;69(3):531-537.

Jacob1993

- Jacobs GD, Rosenberg PA, Friedman R, et al. Multifactor behavioral treatment of chronic sleep-onset insomnia using stimulus control and the relaxation response. A preliminary study. *Behav Modif.* 1993;17(4):498-509.

Jacobs2004

- Jacobs GD, Pace-Schott EF, Stickgold R, Otto MW. Cognitive behavior therapy and pharmacotherapy for insomnia: a randomized controlled trial and direct comparison. *Arch Intern Med*. 2004;164(17):1888-1896.
- NCT00005753

JanssonFrojmark2012a

- Jansson-Fröjmark M, Lind M, Sunnhed R. Don't worry, be constructive: a randomized controlled feasibility study comparing behaviour therapy singly and combined with constructive worry for insomnia. *Br J Clin Psychol*. 2012;51(2):142-157.

JanssonFrojmark2012b

- Jansson-Fröjmark M, Linton SJ, Flink IK, Granberg S, Danermark B, Norell-Clarke A. Cognitive-behavioral therapy for insomnia co-morbid with hearing impairment: a randomized controlled trial. *J Clin Psychol Med Settings.* 2012;19(2):224-234.

Jarnefelt2020

- Järnefelt H, Härmä M, Sallinen M, et al. Cognitive behavioural therapy interventions for insomnia among shift workers: RCT in an occupational health setting. *Int Arch Occup Environ Health*. 2020;93(5):535-550.
- NCT02523079

Javaheri2020

- Javaheri S, Reid M, Drerup M, Mehra R, Redline S. Reducing Coronary Heart Disease Risk Through Treatment of Insomnia Using Web-Based Cognitive Behavioral Therapy for Insomnia: A Methodological Approach. *Behav Sleep Med*. 2020;18(3):334-344.
- NCT02848859

Jernelov2012

© 2024 Furukawa Y et al. *JAMA Psychiatry.*

- Jernelöv S, Lekander M, Blom K, et al. Efficacy of a behavioral self-help treatment with or without therapist guidance for co-morbid and primary insomnia--a randomized controlled trial. *BMC Psychiatry.* 2012;12:5.
- Jernelöv S, Blom K, Hentati Isacsson N, et al. Very long-term outcome of cognitive behavioral therapy for insomnia: one- and ten-year follow-up of a randomized controlled trial. *Cogn Behav Ther.* 2022;51(1):72-88.
- Blom K, Hentati Isacsson N, Forsell E, et al. An investigation and replication of sleep-related cognitions, acceptance and behaviours as predictors of short- and long-term outcome in cognitive behavioural therapy for insomnia. *J Sleep Res*. 2021;30(5):e13376.
- NCT01105052

Johann2020

- Johann AF, Hertenstein E, Feige B, et al. Cognitive behavioural therapy for insomnia does not appear to have a substantial impact on early markers of cardiovascular disease: A preliminary randomized controlled trial. *J Sleep Res*. 2020;29(5):e13102.

Jungquist2010

- Jungquist CR, O'Brien C, Matteson-Rusby S, et al. The efficacy of cognitive-behavioral therapy for insomnia in patients with chronic pain. *Sleep Med*. 2010;11(3):302-309.
- Jungquist CR, Tra Y, Smith MT, et al. The durability of cognitive behavioral therapy for insomnia in patients with chronic pain. *Sleep Disord*. 2012;2012:679648.

Kaku2012

- Kaku A, Nishinoue N, Takano T, et al. Randomized controlled trial on the effects of a combined sleep hygiene education and behavioral approach program on sleep quality in workers with insomnia. *Ind Health*. 2012;50(1):52-59.

Kaldo2015

- Kaldo V, Jernelöv S, Blom K, et al. Guided internet cognitive behavioral therapy for insomnia compared to a control treatment - A randomized trial. *Behav Res Ther*. 2015;71:90-100.
- Blom K, Jernelöv S, Rück C, Lindefors N, Kaldo V. Three-Year Follow-Up of Insomnia and Hypnotics after Controlled Internet Treatment for Insomnia. *Sleep*. 2016;39(6):1267-1274.
- Kraepelien, M., Blom, K., Jernelöv, S. et al. Weekly Self-Ratings of Treatment Involvement and Their Relation to Symptom Reduction in Internet Cognitive Behavioral Therapy for Insomnia. *Cogn Ther Res*. 2021:45;262–271.
- Blom K, Hentati Isacsson N, Forsell E, et al. An investigation and replication of sleep-related cognitions, acceptance and behaviours as predictors of short- and long-term outcome in cognitive behavioural therapy for insomnia. *J Sleep Res*. 2021;30(5):e13376.

© 2024 Furukawa Y et al. *JAMA Psychiatry.*

- Blom K. Guided internet-based treatment for insomnia and depression. *Dissertation.* 2022.

Kaldo2020

- Kaldo V, Bothelius K, Blom K, et al. An open-ended primary-care group intervention for insomnia based on a self-help book - A randomized controlled trial and 4-year follow-up. *J Sleep Res*. 2020;29(1):e12881.

Kallestad2021

- Kallestad H, Scott J, Vedaa Ø, et al. Mode of delivery of Cognitive Behavioral Therapy for Insomnia: a randomized controlled non-inferiority trial of digital and face-to-face therapy. *Sleep*. 2021;44(12):zsab185.
- Kjørstad K, Sivertsen B, Vedaa Ø, et al. The Effect of Reducing Insomnia Severity on Work- and Activity-Related Impairment. *Behav Sleep Med.* 2021;19(4):505-515.
- Scott J, Vedaa Ø, Sivertsen B, Langsrud K, Kallestad H. Using network intervention analysis to explore associations between participant expectations of and difficulties with cognitive behavioural therapy for insomnia and clinical outcome: A proof of principle study. *J Psychiatr Res*. 2022;148:73-83.
- NCT02044263

Kalmbach2020

- Kalmbach DA, Cheng P, O'Brien LM, et al. A randomized controlled trial of digital cognitive behavioral therapy for insomnia in pregnant women. *Sleep Med*. 2020;72:82-92.
- Kalmbach DA, Cheng P, Roth T, et al. Examining Patient Feedback and the Role of Cognitive Arousal in Treatment Non-response to Digital Cognitive-behavioral Therapy for Insomnia during Pregnancy. *Behav Sleep Med*. 2022;20(2):143-163.
- NCT03596879

Kapella2011

- Kapella MC, Herdegen JJ, Perlis ML, et al. Cognitive behavioral therapy for insomnia comorbid with COPD is feasible with preliminary evidence of positive sleep and fatigue effects. *Int J Chron Obstruct Pulmon Dis*. 2011;6:625-635.
- NCT01072292

Kapella2022

- Kapella M, Steffen A, Prasad B, et al. Therapy for insomnia with chronic obstructive pulmonary disease: a randomized trial of components. *J Clin Sleep Med*. 2022;10.5664/jcsm.10210.
- Kapella MC, Herdegen JJ, Laghi F, Steffen AD, Carley DW. Efficacy and mechanisms of behavioral therapy components for insomnia coexisting with chronic obstructive pulmonary disease: study protocol for a randomized controlled trial. *Trials*. 2016;17(1):258.
- NCT01973647

© 2024 Furukawa Y et al. *JAMA Psychiatry.*

Kennet2021

- Kennett L, Bei B, Jackson ML. A Randomized Controlled Trial to Examine the Feasibility and Preliminary Efficacy of a Digital Mindfulness-Based Therapy for Improving Insomnia Symptoms. *Mindfulness*. 2021;12(10):2460-2472.
- ACTRN12620000398909

Krieger2019

- Krieger T, Urech A, Duss SB, et al. A randomized controlled trial comparing guided internet-based multi-component treatment and internet-based guided sleep restriction treatment to care as usual in insomnia. *Sleep Med*. 2019;62:43-52.
- NCT03110263

Kuhn2022

- Kuhn E, Miller KE, Puran D, et al. A Pilot Randomized Controlled Trial of the Insomnia Coach Mobile App to Assess Its Feasibility, Acceptability, and Potential Efficacy. *Behav Ther*. 2022;53(3):440-457.

Kyle2020

- Kyle SD, Hurry MED, Emsley R, et al. The effects of digital cognitive behavioral therapy for insomnia on cognitive function: a randomized controlled trial. *Sleep*. 2020;43(9):zsaa034.
- ISRCTN89237370

Lami2018

- Lami, M.J., Martínez, M.P., Miró, E. et al. Efficacy of Combined Cognitive-Behavioral Therapy for Insomnia and Pain in Patients with Fibromyalgia: A Randomized Controlled Trial. *Cogn Ther Res*. 2018;42:63–79.

Lancee2012

- Lancee J, van den Bout J, van Straten A, Spoormaker VI. Internet-delivered or mailed self-help treatment for insomnia?: a randomized waiting-list controlled trial. *Behav Res Ther*. 2012;50(1):22-29.
- NCT00851188

Lancee2013

- Lancee J, van den Bout J, Sorbi MJ, van Straten A. Motivational support provided via email improves the effectiveness of internet-delivered self-help treatment for insomnia: a randomized trial. *Behav Res Ther*. 2013;51(12):797-805.
- Lancee J, Sorbi MJ, Eisma MC, van Straten A, van den Bout J. The effect of support on internet-delivered treatment for insomnia: does baseline depression severity matter?. *Behav Ther.* 2014;45(4):507-516.
- NCT01456637

© 2024 Furukawa Y et al. *JAMA Psychiatry.*

Lancee2015

- Lancee J, Eisma MC, van Straten A, Kamphuis JH. Sleep-Related Safety Behaviors and Dysfunctional Beliefs Mediate the Efficacy of Online CBT for Insomnia: A Randomized Controlled Trial. *Cogn Behav Ther*. 2015;44(5):406-422.
- NCT01955928

Lancee2016

- Lancee J, van Straten A, Morina N, Kaldo V, Kamphuis JH. Guided Online or Face-to-Face Cognitive Behavioral Treatment for Insomnia: A Randomized Wait-List Controlled Trial. *Sleep*. 2016;39(1):183-191.
- Lancee J, Effting M, van der Zweerde T, van Daal L, van Straten A, Kamphuis JH. Cognitive processes mediate the effects of insomnia treatment: evidence from a randomized wait-list controlled trial. *Sleep Med*. 2019;54:86-93.
- NCT01955850

Lappalainen2019

- Lappalainen P, Langrial S, Oinas-Kukkonen H, Muotka J, Lappalainen R. ACT for sleep - Internet-delivered self-help ACT for sub-clinical and clinical insomnia: A randomized controlled trial. *Journal of Contextual Behavioral Science*, 2019;12:119–127.

Latocha2022

- Latocha KM, Løppenthin KB, Østergaard M, et al. The effect of group-based cognitive behavioural therapy for insomnia in patients with rheumatoid arthritis: A randomised controlled trial [published online ahead of print, 2022 Aug 11]. *Rheumatology*. 2022;keac448.
- Latocha KM, Løppenthin KB, Østergaard M, et al. Cognitive behavioural therapy for insomnia in patients with rheumatoid arthritis: protocol for the randomised, single-blinded, parallel-group Sleep-RA trial. *Trials*. 2020;21(1):440.
- NCT03766100

Leerssen2022

- Leerssen J, Lakbila-Kamal O, Dekkers LMS, et al. Treating Insomnia with High Risk of Depression Using Therapist-Guided Digital Cognitive, Behavioral, and Circadian Rhythm Support Interventions to Prevent Worsening of Depressive Symptoms: A Randomized Controlled Trial. *Psychother Psychosom*. 2022;91(3):168-179.
- Leerssen J, Foster-Dingley JC, Lakbila-Kamal O, et al. Internet-guided cognitive, behavioral and chronobiological interventions in depression-prone insomnia subtypes: protocol of a randomized controlled prevention trial. *BMC Psychiatry*. 2020;20(1):163.
- NL7359

Lichstein2000

- Lichstein KL, Wilson NM, Johnson CT. Psychological treatment of secondary insomnia. *Psychol Aging.* 2000;15(2):232-240.

Lichstein2001

- Lichstein KL, Riedel BW, Wilson NM, Lester KW, Aguillard RN. Relaxation and sleep compression for late-life insomnia: a placebo-controlled trial. *J Consult Clin Psychol*. 2001;69(2):227-239.

Liu2022

- Liu Q, Wang C, Wang Y, et al. Mindfulness-based stress reduction with acupressure for sleep quality in breast cancer patients with insomnia undergoing chemotherapy: A randomized controlled trial. *Eur J Oncol Nurs*. 2022;61:102219. doi:10.1016/j.ejon.2022.102219
- ChiCTR190002561

Lopez2019

- Lopez R, Evangelista E, Barateau L, et al. French Language Online Cognitive Behavioral Therapy for Insomnia Disorder: A Randomized Controlled Trial. *Front Neurol.* 2019;10:1273.
- NCT02539862

Lorenz2019

- Lorenz N, Heim E, Roetger A, Birrer E, Maercker A. Randomized Controlled Trial to Test the Efficacy of an Unguided Online Intervention with Automated Feedback for the Treatment of Insomnia. *Behav Cogn Psychother*. 2019;47(3):287-302.
- Heim E, Rötger A, Lorenz N, Maercker A. Working alliance with an avatar: How far can we go with internet interventions?. *Internet Interv.* 2018;11:41-46.
- NCT02629913

Lovato2014

- Lovato N, Lack L, Wright H, Kennaway DJ. Evaluation of a brief treatment program of cognitive behavior therapy for insomnia in older adults. *Sleep*. 2014;37(1):117-126.
- Lovato N, Micic G, Lack L. Sleep misestimation among older adults suffering from insomnia with short and normal objective sleep duration and the effects of cognitive behavior therapy. *Sleep*. 2021;44(5):zsaa250.
- ACTRN12620000883910

Low2020

© 2024 Furukawa Y et al. *JAMA Psychiatry.*

- Low T, Conduit R, Varma P, Meaklim H, Jackson ML. Treating subclinical and clinical symptoms of insomnia with a mindfulness-based smartphone application: A pilot study. *Internet Interv*. 2020;21:100335.

Maguen2021

- Maguen S, Gloria R, Huggins J, et al. Brief behavioral treatment for insomnia improves psychosocial functioning in veterans: results from a randomized controlled trial. *Sleep.* 2021;44(3):zsaa205.
- Holder N, Kanady JC, Straus LD, et al. Reducing Barriers to Behavioral Treatments for Insomnia: A Qualitative Examination of Veterans' Perspectives of BBTI. *Behav Sleep Med*. 2022;20(1):37-49.
- Ranney RM, Gloria R, Metzler TJ, Huggins J, Neylan TC, Maguen S. Brief behavioral treatment for insomnia decreases trauma-related nightmare frequency in veterans. *J Clin Sleep Med*. 2022;18(7):1831-1839.
- NCT02571452

Majd2020

- Rajabi Majd N, Broström A, Ulander M, et al. Efficacy of a Theory-Based Cognitive Behavioral Technique App-Based Intervention for Patients With Insomnia: Randomized Controlled Trial. *J Med Internet Res*. 2020;22(4):e15841.
- NCT03605732

Manber2008

- Manber R, Edinger JD, Gress JL, San Pedro-Salcedo MG, Kuo TF, Kalista T. Cognitive behavioral therapy for insomnia enhances depression outcome in patients with comorbid major depressive disorder and insomnia. *Sleep*. 2008;31(4):489-495.
- NCT00149825

Manber2016

- Manber R, Buysse DJ, Edinger J, et al. Efficacy of Cognitive-Behavioral Therapy for Insomnia Combined With Antidepressant Pharmacotherapy in Patients With Comorbid Depression and Insomnia: A Randomized Controlled Trial. *J Clin Psychiatry.* 2016;77(10):e1316-e1323.
- Edinger JD, Manber R, Buysse DJ, et al. Are Patients with Childhood Onset of Insomnia and Depression More Difficult to Treat Than Are Those with Adult Onsets of These Disorders? A Report from the TRIAD Study. *J Clin Sleep Med*. 2017;13(2):205-213.
- Bei B, Asarnow LD, Krystal A, Edinger JD, Buysse DJ, Manber R. Treating insomnia in depression: Insomnia related factors predict long-term depression trajectories. *J Consult Clin Psychol*. 2018;86(3):282-293.

© 2024 Furukawa Y et al. *JAMA Psychiatry.*

- Asarnow LD, Bei B, Krystal A, et al. Circadian Preference as a Moderator of Depression Outcome Following Cognitive Behavioral Therapy for Insomnia Plus Antidepressant Medications: A Report From the TRIAD Study. *J Clin Sleep Med*. 2019;15(4):573-580.
- NCT00767624

Manber2019

- Manber R, Bei B, Simpson N, et al. Cognitive Behavioral Therapy for Prenatal Insomnia: A Randomized Controlled Trial. *Obstet Gynecol*. 2019;133(5):911-919.

Mao2018

- Mao H, Wu J, Xu Y, Liu Y, Tang X. Effectiveness of sleep self-management group intervention in Chinese patients with insomnia disorder. *Perspect Psychiatr Care*. 2018;54(2):156-161.

Marino2001

- Marino, A. Treating chronic insomnia: A cognitive-behavioural group therapy approach. Disseration. 2001.

Maritinez2014

- Martínez MP, Miró E, Sánchez AI, et al. Cognitive-behavioral therapy for insomnia and sleep hygiene in fibromyalgia: a randomized controlled trial. *J Behav Med*. 2014;37(4):683-697.

Marks2023

- Marks E, Hallsworth C, Vogt F, Klein H, McKenna L. Cognitive behavioural therapy for insomnia (CBTi) as a treatment for tinnitus-related insomnia: a randomised controlled trial. *Cogn Behav Ther*. 2023;52(2):91-109. doi:10.1080/16506073.2022.2084155
- NCT03386123

Matthews2014

- Matthews EE, Berger AM, Schmiege SJ, et al. Cognitive behavioral therapy for insomnia outcomes in women after primary breast cancer treatment: a randomized, controlled trial. *Oncol Nurs Forum*. 2014;41(3):241-253.

McCrae2007

- McCrae CS, McGovern R, Lukefahr R, Stripling AM. Research Evaluating Brief Behavioral Sleep Treatments for Rural Elderly (RESTORE): a preliminary examination of effectiveness. *Am J Geriatr Psychiatry*. 2007;15(11):979-982.

McCrae2019

© 2024 Furukawa Y et al. *JAMA Psychiatry.*

- McCrae CS, Williams J, Roditi D, et al. Cognitive behavioral treatments for insomnia and pain in adults with comorbid chronic insomnia and fibromyalgia: clinical outcomes from the SPIN randomized controlled trial. *Sleep*. 2019;42(3):zsy234.
- Mundt JM, Crew EC, Krietsch K, et al. Measuring Treatment Outcomes in Comorbid Insomnia and Fibromyalgia: Concordance of Subjective and Objective Assessments. *J Clin Sleep Med*. 2016;12(2):215-223.
- McCrae CS, Mundt JM, Curtis AF, et al. Gray Matter Changes Following Cognitive Behavioral Therapy for Patients With Comorbid Fibromyalgia and Insomnia: A Pilot Study. *J Clin Sleep Med*. 2018;14(9):1595-1603.
- McCrae CS, Curtis AF, Miller MB, et al. Effect of cognitive behavioural therapy on sleep and opioid medication use in adults with fibromyalgia and insomnia. *J Sleep Res*. 2020;29(6):e13020.
- NCT02001077

McCra2020

- McCrae CS, Curtis AF, Williams JM, et al. Effects of Brief Behavioral Treatment for Insomnia on Daily Associations between Self-Reported Sleep and Objective Cognitive Performance in Older Adults. *Behav Sleep Med*. 2020;18(5):577-588.
- Chan WS, Williams J, Dautovich ND, et al. Night-to-Night Sleep Variability in Older Adults With Chronic Insomnia: Mediators and Moderators in a Randomized Controlled Trial of Brief Behavioral Therapy (BBT-I). *J Clin Sleep Med*. 2017;13(11):1243-1254.
- McCrae CS, Curtis AF, Nair N, et al. Impact of a brief behavioral treatment for insomnia (BBTi) on metacognition in older adults. *Sleep Med*. 2021;80:286-293.
- NCT02967185

McCurry2016

- McCurry SM, Guthrie KA, Morin CM, et al. Telephone-Based Cognitive Behavioral Therapy for Insomnia in Perimenopausal and Postmenopausal Women With Vasomotor Symptoms: A MsFLASH Randomized Clinical Trial. *JAMA Intern Med*. 2016;176(7):913-920.
- Kauffman RP. Telephone-based CBT reduced insomnia severity more than menopause education in menopausal women. *Ann Intern Med*. 2016;165(6):JC30.
- NCT01936441

McCurry2021

- McCurry SM, Zhu W, Von Korff M, et al. Effect of Telephone Cognitive Behavioral Therapy for Insomnia in Older Adults With Osteoarthritis Pain: A Randomized Clinical Trial. *JAMA Intern Med*. 2021;181(4):530-538.

© 2024 Furukawa Y et al. *JAMA Psychiatry.*

- McCurry SM, Von Korff M, Morin CM, et al. Telephone interventions for co-morbid insomnia and osteoarthritis pain: The OsteoArthritis and Therapy for Sleep (OATS) randomized trial design. *Contemp Clin Trials*. 2019;87:105851.
- Yeung K, Zhu W, McCurry SM, et al. Cost-effectiveness of telephone cognitive behavioral therapy for osteoarthritis-related insomnia. *J Am Geriatr Soc*. 2022;70(1):188-199.
- Vitiello MV, Zhu W, Von Korff M, et al. Long-term improvements in sleep, pain, depression, and fatigue in older adults with comorbid osteoarthritis pain and insomnia. *Sleep*. 2022;45(2):zsab231.
- NCT02946957

Means2000

- Means MK, Lichstein KL, Epperson MT, Johnson CT. Relaxation therapy for insomnia: nighttime and day time effects. *Behav Res Ther*. 2000;38(7):665-678.

Milby1993

- Milby JB, Williams V, Hall JN, Khuder S, McGill T, Wooten V. Effectiveness of combined triazolam-behavioral therapy for primary insomnia. *Am J Psychiatry.* 1993;150(8):1259-1260.

Miller2021

- Miller MB, Deroche CB, Freeman LK, et al. Cognitive behavioral therapy for insomnia among young adults who are actively drinking: a randomized pilot trial. *Sleep*. 2021;44(2):zsaa171.
- Miller MB, Freeman L, Park CJ, et al. Insomnia treatment effects among young adult drinkers: Secondary outcomes of a randomized pilot trial. *Alcohol Clin Exp Res.* 2021;45(5):1136-1148. doi:10.1111/acer.14603
- Miller MB, Carpenter RW, Freeman LK, Curtis AF, Yurasek AM, McCrae CS. Cannabis use as a moderator of cognitive behavioral therapy for insomnia. *J Clin Sleep Med*. 2022;18(4):1047-1054.
- NCT03627832

Miller2023

- Miller MB, Carpenter RW, Freeman LK, et al. Effect of Cognitive Behavioral Therapy for Insomnia on Alcohol Treatment Outcomes Among US Veterans: A Randomized Clinical Trial [published online ahead of print, 2023 Jun 21]. *JAMA Psychiatry*. 2023;e231971. doi:10.1001/jamapsychiatry.2023.1971
- NCT03806491

Mimeault1999

- Mimeault V, Morin CM. Self-help treatment for insomnia: bibliotherapy with and without professional guidance. *J Consult Clin Psychol*. 1999;67(4):511-519.

Miro2011

© 2024 Furukawa Y et al. *JAMA Psychiatry.*

- Miró E, Lupiáñez J, Martínez MP, et al. Cognitive-behavioral therapy for insomnia improves attentional function in fibromyalgia syndrome: a pilot, randomized controlled trial. *J Health Psychol*. 2011;16(5):770-782.

Morawetz1989
- Morawetz, D. Behavioral self-help treatment for insomnia: A controlled evaluation. *Behavior Therapy.* 1989;20(3):365-379.

Morgan2012
- Morgan K, Gregory P, Tomeny M, David BM, Gascoigne C. Self-help treatment for insomnia symptoms associated with chronic conditions in older adults: a randomized controlled trial. *J Am Geriatr Soc*. 2012;60(10):1803-1810.

Morin1988
- Morin CM, Azrin NH. Behavioral and cognitive treatments of geriatric insomnia. *J Consult Clin Psychol.* 1988;56(5):748-753.

Morin1993
- Morin CM, Kowatch RA, Barry T, Walton E. Cognitive-behavior therapy for late-life insomnia. *J Consult Clin Psychol*. 1993;61(1):137-146.

Morin1999
- Morin CM, Colecchi C, Stone J, Sood R, Brink D. Behavioral and pharmacological therapies for late-life insomnia: a randomized controlled trial. *JAMA*. 1999;281(11):991-999.
- Morin CM, Blais F, Savard J. Are changes in beliefs and attitudes about sleep related to sleep improvements in the treatment of insomnia?. *Behav Res Ther*. 2002;40(7):741-752.

NCT00127790
- Treatment of Insomnia Secondary to Chronic Pain

NCT01011218
- Management of Insomnia in Breast Cancer Patients

NCT01987089
- The Efficacy of CBT-I in Alcoholics & Its Effects on Remission & Relapse

NCT02613364

© 2024 Furukawa Y et al. *JAMA Psychiatry.*

- Yoga, Survivorship Health Education, and Cognitive Behavioral Therapy in Reducing Insomnia in Cancer Survivors (YOCAS-II)

NCT03964974

- Reducing Cannabis Use for Sleep Among Adults Using Medical Cannabis (CannSleep)

NorellClarke2015

- Norell-Clarke A, Jansson-Fröjmark M, Tillfors M, Holländare F, Engström I. Group cognitive behavioural therapy for insomnia: Effects on sleep and depressive symptomatology in a sample with comorbidity. *Behav Res Ther*. 2015;74:80-93.
- Norell-Clarke A, Tillfors M, Jansson-Fröjmark M, Holländare F, Engström I. How Does Cognitive Behavioral Therapy for Insomnia Work? An Investigation of Cognitive Processes and Time in Bed as Outcomes and Mediators in a Sample With Insomnia and Depressive Symptomatology. *International Journal of Cognitive Therapy.* 2017;10(4):304-329.
- Norell-Clarke A, Tillfors M, Jansson-Fröjmark M, Holländare F, Engström I. Does Mid-Treatment Insomnia Severity Mediate between Cognitive Behavioural Therapy for Insomnia and Post-Treatment Depression? An Investigation in a Sample with Comorbid Insomnia and Depressive Symptomatology. *Behav Cogn Psychother.* 2018;46(6):726-737.

Okajima2020

- Okajima I, Akitomi J, Kajiyama I, Ishii M, Murakami H, Yamaguchi M. Effects of a Tailored Brief Behavioral Therapy Application on Insomnia Severity and Social Disabilities Among Workers With Insomnia in Japan: A Randomized Clinical Trial. *JAMA Netw Open*. 2020;3(4):e202775.

Okajima2021

- Okajima I, Tanizawa N, Harata M, et al. Can an E-Mail-Delivered CBT for Insomnia Validated in the West Be Effective in the East? A Randomized Controlled Trial. *Int J Environ Res Public Health*. 2021;19(1):186.
- Ubara A, Tanizawa N, Harata M, et al. How Does E-mail-Delivered Cognitive Behavioral Therapy Work for Young Adults (18-28 Years) with Insomnia? Mediators of Changes in Insomnia, Depression, Anxiety, and Stress. *Int J Environ Res Public Health*. 2022;19(8):4423.
- UMIN000043685

Ong2018

- Ong JC, Xia Y, Smith-Mason CE, Manber R. A randomized controlled trial of mindfulness meditation for chronic insomnia: Effects on daytime symptoms and cognitive-emotional arousal. *Mindfulness*. 2018;9(6):1702–1712.

- Ong JC, Hedeker D, Wyatt JK, Manber R. Examining the Variability of Sleep Patterns during Treatment for Chronic Insomnia: Application of a Location-Scale Mixed Model. *J Clin Sleep Med.* 2016;12(6):797-804.
- Goldstein MR, Turner AD, Dawson SC, et al. Increased high-frequency NREM EEG power associated with mindfulness-based interventions for chronic insomnia: Preliminary findings from spectral analysis. *J Psychosom Res*. 2019;120:12-19.
- NCT00768781

Ong2020

- Ong JC, Crawford MR, Dawson SC, et al. A randomized controlled trial of CBT-I and PAP for obstructive sleep apnea and comorbid insomnia: main outcomes from the MATRICS study. *Sleep.* 2020;43(9):zsaa041.
- Crawford MR, Turner AD, Wyatt JK, Fogg LF, Ong JC. Evaluating the treatment of obstructive sleep apnea comorbid with insomnia disorder using an incomplete factorial design. *Contemp Clin Trials.* 2016;47:146-152.
- Tu AY, Crawford MR, Dawson SC, et al. A randomized controlled trial of cognitive behavioral therapy for insomnia and PAP for obstructive sleep apnea and comorbid insomnia: effects on nocturnal sleep and daytime performance. *J Clin Sleep Med*. 2022;18(3):789-800.
- NCT01785303

Oswald2022

- Oswald LB, Morales-Cruz J, Eisel SL, et al. Pilot randomized controlled trial of eHealth cognitive-behavioral therapy for insomnia among Spanish-speaking breast cancer survivors. *J Behav Med*. 2022;45(3):503-508.
- NCT04101526

Padron2022

- Padron A, McCrae CS, Robinson ME, et al. Impacts of Cognitive Behavioral Therapy for Insomnia and Pain on Sleep in Women with Gynecologic Malignancies: A Randomized Controlled Trial. *Behav Sleep Med*. 2022;20(4):460-476.
- NCT02609880

Palesh2018

- Palesh O, Scheiber C, Kesler S, et al. Feasibility and acceptability of brief behavioral therapy for cancer-related insomnia: effects on insomnia and circadian rhythm during chemotherapy: a phase II randomised multicentre controlled trial. *Br J Cancer*. 2018;119(3):274-281.
- Palesh O, Scheiber C, Kesler S, et al. Secondary outcomes of a behavioral sleep intervention: A randomized clinical trial. *Health Psychol*. 2019;38(3):196-205.

© 2024 Furukawa Y et al. *JAMA Psychiatry.*

- NCT02002533

Palesh2020

- Palesh O, Solomon N, Hofmeister E, et al. A novel approach to management of sleep-associated problems in patients with breast cancer (MOSAIC) during chemotherapy : A pilot study. *Sleep*. 2020;43(10):zsaa070.

Pallesen2003

- Pallesen S, Nordhus IH, Kvale G, et al. Behavioral treatment of insomnia in older adults: an open clinical trial comparing two interventions. *Behav Res Ther*. 2003;41(1):31-48.

Patel2017

- Patel S, Ojo O, Genc G, et al. *A C*omputerized *Co*gnitive behavioral therapy *R*andomized, Controll*ed*, pilot trial for insomnia in *P*arkinson *D*isease (*ACCORD-PD*). *J Clin Mov Disord*. 2017;4:16.

Perlis2004

- Perlis ML, Smith MT, Orff H, et al. The effects of modafinil and cognitive behavior therapy on sleep continuity in patients with primary insomnia. *Sleep*. 2004;27(4):715-725.

Perrault2022

- Perrault AA, Pomares FB, Smith D, et al. Effects of cognitive behavioral therapy for insomnia on subjective and objective measures of sleep and cognition. *Sleep Med*. 2022;97:13-26.
- ISRCTN13983243

Pigeon2017

- Pigeon WR, Funderburk J, Bishop TM, Crean HF. Brief cognitive behavioral therapy for insomnia delivered to depressed veterans receiving primary care services: A pilot study. *J Affect Disord*. 2017;217:105-111.

Pigeon2019

- Pigeon WR, Funderburk JS, Cross W, Bishop TM, Crean HF. Brief CBT for insomnia delivered in primary care to patients endorsing suicidal ideation: a proof-of-concept randomized clinical trial. *Transl Behav Med*. 2019;9(6):1169-1177.
- Bishop TM, Crean HF, Funderburk JS, Pigeon WR. Initial Session Effects of Brief Cognitive Behavioral Therapy for Insomnia: A Secondary Analysis of A Small Randomized Pilot Trial. *Behav Sleep Med*. 2021;19(6):769-782.
- NCT02248675

© 2024 Furukawa Y et al. *JAMA Psychiatry.*

Pigeon2022

- Pigeon WR, Crean HF, Cerulli C, Gallegos AM, Bishop TM, Heffner KL. A Randomized Clinical Trial of Cognitive-Behavioral Therapy for Insomnia to Augment Posttraumatic Stress Disorder Treatment in Survivors of Interpersonal Violence. *Psychother Psychosom*. 2022;91(1):50-62.
- Pigeon WR, Heffner KL, Crean H, et al. Responding to the need for sleep among survivors of interpersonal violence: A randomized controlled trial of a cognitive-behavioral insomnia intervention followed by PTSD treatment. *Contemp Clin Trials.* 2015;45(Pt B):252-260.
- NCT01743339

Pillai2015

- Pillai V, Anderson JR, Cheng P, et al. The Anxiolytic Effects of Cognitive Behavior Therapy for Insomnia: Preliminary Results from a Web-delivered Protocol. *J Sleep Med Disord*. 2015;2(2):1017.

Prados2020

- Prados G, Miró E, Martínez MP, Sánchez AI, Lami MJ, Cáliz R. Combined cognitive-behavioral therapy for fibromyalgia: Effects on polysomnographic parameters and perceived sleep quality. *Int J Clin Health Psychol*. 2020;20(3):232-242.

Quintiliani2020

- Quintiliani MI, Imperatori C, Testani E, et al. Usefulness of psychoeducational intervention in chronic insomnia: an actigraphic study. *J Ment Health*. 2020;29(1):20-26.

Redeker2015

- Redeker NS, Jeon S, Andrews L, Cline J, Jacoby D, Mohsenin V. Feasibility and Efficacy of a Self-Management Intervention for Insomnia in Stable Heart Failure. *J Clin Sleep Med*. 2015;11(10):1109-1119.
- Redeker NS, Conley S, Anderson G, et al. Effects of Cognitive Behavioral Therapy for Insomnia on Sleep, Symptoms, Stress, and Autonomic Function Among Patients With Heart Failure. *Behav Sleep Med*. 2020;18(2):190-202.
- Redeker NS, Jeon S, Andrews L, Cline J, Mohsenin V, Jacoby D. Effects of Cognitive Behavioral Therapy for Insomnia on Sleep-Related Cognitions Among Patients With Stable Heart Failure. *Behav Sleep Med*. 2019;17(3):342-354.
- NCT02827799

Redeker2022

- Redeker NS, Yaggi HK, Jacoby D, et al. Cognitive behavioral therapy for insomnia has sustained effects on insomnia, fatigue, and function among people with chronic heart failure and insomnia: the HeartSleep Study. *Sleep*. 2022;45(1):zsab252.

© 2024 Furukawa Y et al. *JAMA Psychiatry.*

- Redeker NS, Knies AK, Hollenbeak C, et al. Cognitive behavioral therapy for insomnia in stable heart failure: Protocol for a randomized controlled trial. *Contemp Clin Trials*. 2017;55:16-23.
- Gaffey AE, Jeon S, Conley S, et al. Perceived Stress, Subjective, and Objective Symptoms of Disturbed Sleep in Men and Women with Stable Heart Failure. *Behav Sleep Med*. 2021;19(3):363-377.
- O'Connell M, Jeon S, Conley S, Linsky S, Redeker NS. Sleep, Stress, and Symptoms Among People With Heart Failure During the COVID-19 Pandemic. *J Cardiovasc Nurs*. 2022;10.1097/JCN.0000000000000906.
- NCT02660385

Ritterband2009

- Ritterband LM, Thorndike FP, Gonder-Frederick LA, et al. Efficacy of an Internet-based behavioral intervention for adults with insomnia. *Arch Gen Psychiatry*. 2009;66(7):692-698.
- Thorndike FP, Ritterband LM, Gonder-Frederick LA, Lord HR, Ingersoll KS, Morin CM. A randomized controlled trial of an internet intervention for adults with insomnia: effects on comorbid psychological and fatigue symptoms. *J Clin Psychol*. 2013;69(10):1078-1093.
- Shaffer KM, Hedeker D, Morin CM, Ingersoll K, Thorndike F, Ritterband LM. Intraindividual variability in sleep schedule: effects of an internet-based cognitive-behavioral therapy for insomnia program and its relation with symptom remission. *Sleep*. 2020;43(12):zsaa115.
- Shaffer KM, Finkelstein EA, Camacho F, Ingersoll KS, Thorndike F, Ritterband LM. Effects of an Internet-Based Cognitive Behavioral Therapy for Insomnia Program on Work Productivity: A Secondary Analysis. *Ann Behav Med*. 2021;55(6):592-599.
- NCT00328250

Ritterband2012

- Ritterband LM, Bailey ET, Thorndike FP, Lord HR, Farrell-Carnahan L, Baum LD. Initial evaluation of an Internet intervention to improve the sleep of cancer survivors with insomnia. *Psychooncology.* 2012;21(7):695-705.

Ritterband2017

- Ritterband LM, Thorndike FP, Ingersoll KS, et al. Effect of a Web-Based Cognitive Behavior Therapy for Insomnia Intervention With 1-Year Follow-up: A Randomized Clinical Trial. *JAMA Psychiatry*. 2017;74(1):68-75.
- Chow PI, Ingersoll KS, Thorndike FP, et al. Cognitive mechanisms of sleep outcomes in a randomized clinical trial of internet-based cognitive behavioral therapy for insomnia. *Sleep Med*. 2018;47:77-85.
- Chow PI, Gonzalez BD, Ingersoll KS, et al. A secondary analysis of the role of geography in engagement and outcomes in a clinical trial of an efficacious Internet intervention for insomnia. *Internet Interv.* 2019;18:100294.

© 2024 Furukawa Y et al. *JAMA Psychiatry.*

- Shaffer KM, Camacho F, Lord HR, et al. Do treatment effects of a web-based cognitive behavioral therapy for insomnia intervention differ for users with and without pain interference? A secondary data analysis. *J Behav Med*. 2020;43(3):503-510.
- NCT01438697

Robabeh2015

- Robabeh S Md, Jafar MM Md, Sharareh H Md, Maryam HR Md, Masoumeh E Ma. The Effect of Cognitive Behavior Therapy in Insomnia due to Methadone Maintenance Therapy: A Randomized Clinical Trial. *Iran J Med Sci*. 2015;40(5):396-403.

Roscoe2015

- Roscoe JA, Garland SN, Heckler CE, et al. Randomized placebo-controlled trial of cognitive behavioral therapy and armodafinil for insomnia after cancer treatment. *J Clin Oncol*. 2015;33(2):165-171.
- Garland SN, Roscoe JA, Heckler CE, et al. Effects of armodafinil and cognitive behavior therapy for insomnia on sleep continuity and daytime sleepiness in cancer survivors. *Sleep Med*. 2016;20:18-24.
- Peoples AR, Garland SN, Perlis ML, et al. Effects of cognitive behavioral therapy for insomnia and armodafinil on quality of life in cancer survivors: a randomized placebo-controlled trial. *J Cancer Surviv*. 2017;11(3):401-409.
- Kamen C, Garland SN, Heckler CE, et al. Social Support, Insomnia, and Adherence to Cognitive Behavioral Therapy for Insomnia After Cancer Treatment. *Behav Sleep Med*. 2019;17(1):70-80.
- NCT01091974

Rosen2000

- Rosen RC, Lewin DS, Goldberg L, Woolfolk RL. Psychophysiological insomnia: combined effects of pharmacotherapy and relaxation-based treatments. *Sleep Med*. 2000;1(4):279-288.

Rybarczyk2005

- Rybarczyk B, Stepanski E, Fogg L, Lopez M, Barry P, Davis A. A placebo-controlled test of cognitive-behavioral therapy for comorbid insomnia in older adults. *J Consult Clin Psychol.* 2005;73(6):1164-1174.
- Lund HG, Rybarczyk BD, Perrin PB, Leszczyszyn D, Stepanski E. The discrepancy between subjective and objective measures of sleep in older adults receiving CBT for comorbid insomnia. *J Clin Psychol.* 2013;69(10):1108-1120.
- Vitiello MV, Rybarczyk B, Von Korff M, Stepanski EJ. Cognitive behavioral therapy for insomnia improves sleep and decreases pain in older adults with co-morbid insomnia and osteoarthritis. *J Clin Sleep Med.* 2009;5(4):355-362.

Sadler2018

© 2024 Furukawa Y et al. *JAMA Psychiatry.*

- Sadler P, McLaren S, Klein B, Harvey J, Jenkins M. Cognitive behavior therapy for older adults with insomnia and depression: a randomized controlled trial in community mental health services. *Sleep*. 2018;41(8):10.1093/sleep/zsy104.
- Sadler P, McLaren S, Klein B, Jenkins M, Harvey J. Cognitive behaviour therapy for older adults experiencing insomnia and depression in a community mental health setting: Study protocol for a randomised controlled trial. *Trials*. 2015;16:538.
- Sadler P, McLaren S, Klein B, Jenkins M. Cognitive behaviour therapy for insomnia and depression: qualitative reflections from older adults who participated in a randomised controlled trial. *Aging Ment Health*. 2020;24(6):932-938.
- ACTRN12615000067572

Sandlund2017

- Sandlund C, Hetta J, Nilsson GH, Ekstedt M, Westman J. Improving insomnia in primary care patients: A randomized controlled trial of nurse-led group treatment. *Int J Nurs Stud*. 2017;72:30-41.
- Sandlund C, Hetta J, Nilsson GH, Ekstedt M, Westman J. Impact of group treatment for insomnia on daytime symptomatology: Analyses from a randomized controlled trial in primary care. *Int J Nurs Stud*. 2018;85:126-135.
- NCT01731223

Sato2019

- Sato D, Yoshinaga N, Nagai E, Nagai K, Shimizu E. Effectiveness of Internet-Delivered Computerized Cognitive Behavioral Therapy for Patients With Insomnia Who Remain Symptomatic Following Pharmacotherapy: Randomized Controlled Exploratory Trial. *J Med Internet Res*. 2019;21(4):e12686.
- Sato D, Yoshinaga N, Nagai E, Hanaoka H, Sato Y, Shimizu E. Randomised controlled trial on the effect of internet-delivered computerised cognitive-behavioural therapy on patients with insomnia who remain symptomatic following hypnotics: a study protocol. *BMJ Open*. 2018;8(1):e018220.
- JPRN-UMIN000021509

Sato2022

- Sato D, Sekizawa Y, Sutoh C, et al. Effectiveness of Unguided Internet-Based Cognitive Behavioral Therapy and the Three Good Things Exercise for Insomnia: 3-Arm Randomized Controlled Trial. *J Med Internet Res.* 2022;24(2):e28747.
- JPRN-UMIN000034927

Savard2005

- Savard J, Simard S, Ivers H, Morin CM. Randomized study on the efficacy of cognitive-behavioral therapy for insomnia secondary to breast cancer, part I: Sleep and psychological effects. *J Clin Oncol*. 2005;23(25):6083-6096.

© 2024 Furukawa Y et al. *JAMA Psychiatry.*

- Savard J, Simard S, Ivers H, Morin CM. Randomized study on the efficacy of cognitive-behavioral therapy for insomnia secondary to breast cancer, part II: Immunologic effects. *J Clin Oncol*. 2005;23(25):6097-6106.

Savard2014

- Savard J, Ivers H, Savard MH, Morin CM. Is a video-based cognitive behavioral therapy for insomnia as efficacious as a professionally administered treatment in breast cancer? Results of a randomized controlled trial. *Sleep*. 2014;37(8):1305-1314.
- Savard J, Ivers H, Savard MH, Morin CM. Long-Term Effects of Two Formats of Cognitive Behavioral Therapy for Insomnia Comorbid with Breast Cancer. *Sleep*. 2016;39(4):813-823.
- Savard J, Savard MH, Ivers H. Moderators of Treatment Effects of a Video-Based Cognitive-Behavioral Therapy for Insomnia Comorbid With Cancer. *Behav Sleep Med*. 2018;16(3):294-309.
- Savard J, Ivers H, Morin CM, Lacroix G. Video cognitive-behavioral therapy for insomnia in cancer patients: A cost-effective alternative. *Psychooncology*. 2021;30(1):44-51.
- NCT00674830

Schuffelen2023

- Schuffelen J, Maurer LF, Lorenz N, Rötger A, Pietrowsky R, Gieselmann A. The clinical effects of digital cognitive behavioral therapy for insomnia in a heterogenous study sample: Results from a randomized controlled trial [published online ahead of print, 2023 Jul 10]. *Sleep*. 2023;zsad184. doi:10.1093/sleep/zsad184
- DRKS00024477

Sgoifo2017

- Sgoifo A, Bignamini A, La Mantia L, et al. Integrated Imaginative Distention Therapy to Cope with Fatigue. DIMMI SI Study: The First Randomized Controlled Trial in Multiple Sclerosis. *Neurol Ther*. 2017;6(2):213-223.
- NCT02290990

Sheaves2018

- Sheaves B, Freeman D, Isham L, et al. Stabilising sleep for patients admitted at acute crisis to a psychiatric hospital (OWLS): an assessor-blind pilot randomised controlled trial. *Psychol Med*. 2018;48(10):1694-1704.
- ISRCTN15324584

Shimamoto2022

© 2024 Furukawa Y et al. *JAMA Psychiatry.*

- Shimamoto T, Furihata R, Nakagami Y, et al. Providing Brief Personalized Therapies for Insomnia Among Workers Using a Sleep Prompt App: Randomized Controlled Trial. *J Med Internet Res*. 2022;24(7):e36862.
- UMIN000042263

Shin2023

- Shin JW, Kim S, Shin YJ, Park B, Park S. Comparison of Acceptance and Commitment Therapy (ACT) and Cognitive Behavior Therapy (CBT) for Chronic Insomnia: A Pilot Randomized Controlled Trial. *Nat Sci Sleep*. 2023;15:523-531. Published 2023 Jul 5. doi:10.2147/NSS.S409981
- KCT0008296

Short2021

- Short NA, Zvolensky MJ, Schmidt NB. A pilot randomized clinical trial of Brief Behavioral Treatment for Insomnia to reduce problematic cannabis use among trauma-exposed young adults. *J Subst Abuse Treat*. 2021;131:108537.

Siebmanns2021

- Siebmanns S, Johansson P, Ulander M, Johansson L, Andersson G, Broström A. The effect of nurse-led Internet-based cognitive behavioural therapy for insomnia on patients with cardiovascular disease: A randomized controlled trial with 6-month follow-up. *Nurs Open*. 2021;8(4):1755-1768.
- NCT03938805

Siengsukon2020

- Siengsukon CF, Alshehri M, Williams C, Drerup M, Lynch S. Feasibility and treatment effect of cognitive behavioral therapy for insomnia in individuals with multiple sclerosis: A pilot randomized controlled trial. *Mult Scler Relat Disord.* 2020;40:101958.
- Williams-Cooke C, LeSuer L, Drerup M, Siengsukon C. The Impact of Cognitive Behavioral Therapy for Insomnia on Sleep Log and Actigraphy Outcomes in People with Multiple Sclerosis: A Secondary Analysis. *Nat Sci Sleep*. 2021;13:1865-1874.
- NCT03216889

Siengsukon2021

- Siengsukon CF, Beck ES Jr, Drerup M. Feasibility and Treatment Effect of a Web-Based Cognitive Behavioral Therapy for Insomnia Program in Individuals with Multiple Sclerosis: A Pilot Randomized Controlled Trial. *Int J MS Care.* 2021;23(3):107-113.
- NCT03783585

Simeit2004

- Simeit R, Deck R, Conta-Marx B. Sleep management training for cancer patients with insomnia. *Support Care Cancer*. 2004;12(3):176-183.

Smith2015

- Smith MT, Finan PH, Buenaver LF, et al. Cognitive-behavioral therapy for insomnia in knee osteoarthritis: a randomized, double-blind, active placebo-controlled clinical trial. *Arthritis Rheumatol*. 2015;67(5):1221-1233.
- Salwen JK, Smith MT, Finan PH. Mid-Treatment Sleep Duration Predicts Clinically Significant Knee Osteoarthritis Pain reduction at 6 months: Effects From a Behavioral Sleep Medicine Clinical Trial. *Sleep.* 2017;40(2):zsw064.
- Lerman SF, Finan PH, Smith MT, Haythornthwaite JA. Psychological interventions that target sleep reduce pain catastrophizing in knee osteoarthritis. *Pain*. 2017;158(11):2189-2195.
- NCT00592449

Smitherman2016

- Smitherman TA, Walters AB, Davis RE, et al. Randomized Controlled Pilot Trial of Behavioral Insomnia Treatment for Chronic Migraine With Comorbid Insomnia. *Headache*. 2016;56(2):276-291.

Soeffing2008

- Soeffing JP, Lichstein KL, Nau SD, et al. Psychological treatment of insomnia in hypnotic-dependant older adults. *Sleep Med.* 2008;9(2):165-171.

Song2020

- Song ML, Park KM, Motamedi GK, Cho YW. Cognitive behavioral therapy for insomnia in restless legs syndrome patients. *Sleep Med*. 2020;74:227-234.

Speed2022

- Speed TJ, Hanks L, Turner G, et al. A comparison of cognitive behavioral therapy for insomnia to standard of care in an outpatient substance use disorder clinic embedded within a therapeutic community: a RE-AIM framework evaluation. *Trials*. 2022 Nov;23(1):965.
- NCT03208855

Store2023

- Støre SJ, Tillfors M, Wästlund E, Angelhoff C, Andersson G, Norell-Clarke A. The effects of a sleep robot intervention on sleep, depression and anxiety in adults with insomnia-A randomized waitlist-controlled trial. *J Sleep Res*. 2023;32(3):e13758. doi:10.1111/jsr.13758
- ISCRTN35134834

© 2024 Furukawa Y et al. *JAMA Psychiatry.*

Strom2004

- Ström L, Pettersson R, Andersson G. Internet-based treatment for insomnia: a controlled evaluation. *J Consult Clin Psychol*. 2004;72(1):113-120.

Sunnheld2020

- Sunnhed R, Hesser H, Andersson G, et al. Comparing internet-delivered cognitive therapy and behavior therapy with telephone support for insomnia disorder: a randomized controlled trial. *Sleep*. 2020;43(2):zsz245.
- Blanken TF, Jansson-Fröjmark M, Sunnhed R, Lancee J. Symptom-specific effects of cognitive therapy and behavior therapy for insomnia: A network intervention analysis. *J Consult Clin Psychol*. 2021;89(4):364-370.
- NCT02984670

Sveen2021

- Sveen J, Jernelöv S, Pohlkamp L, Kreicbergs U, Kaldo V. Feasibility and preliminary efficacy of guided internet-delivered cognitive behavioral therapy for insomnia after the loss of a child to cancer: Randomized controlled trial. *Internet Interv*. 2021;25:100409.
- NCT02886052

Sweetman2019

- Sweetman A, Lack L, Catcheside PG, et al. Cognitive and behavioral therapy for insomnia increases the use of continuous positive airway pressure therapy in obstructive sleep apnea participants with comorbid insomnia: a randomized clinical trial. *Sleep*. 2019;42(12):zsz178.
- Sweetman A, McEvoy RD, Smith S, et al. The effect of cognitive and behavioral therapy for insomnia on week-to-week changes in sleepiness and sleep parameters in patients with comorbid insomnia and sleep apnea: a randomized controlled trial. *Sleep*. 2020;43(7):zsaa002.
- Sweetman A, Lack L, McEvoy RD, et al. Cognitive behavioural therapy for insomnia reduces sleep apnoea severity: a randomised controlled trial. *ERJ Open Res*. 2020;6(2):00161-2020.
- Sweetman A, Lechat B, Catcheside PG, et al. Polysomnographic Predictors of Treatment Response to Cognitive Behavioral Therapy for Insomnia in Participants With Co-morbid Insomnia and Sleep Apnea: Secondary Analysis of a Randomized Controlled Trial. *Front Psychol*. 2021;12:676763.
- Sweetman A, Lack L, McEvoy RD, et al. Effect of depression, anxiety, and stress symptoms on response to cognitive behavioral therapy for insomnia in patients with comorbid insomnia and sleep apnea: a randomized controlled trial. *J Clin Sleep Med*. 2021;17(3):545-554.
- ACTRN12613001178730

Talbot2014

- Talbot LS, Maguen S, Metzler TJ, et al. Cognitive behavioral therapy for insomnia in posttraumatic stress disorder: a randomized controlled trial. *Sleep*. 2014;37(2):327-341.
- Kanady JC, Talbot LS, Maguen S, et al. Cognitive Behavioral Therapy for Insomnia Reduces Fear of Sleep in Individuals With Posttraumatic Stress Disorder. *J Clin Sleep Med*. 2018;14(7):1193-1203.
- NCT00881647

Taylor2014
- Taylor DJ, Zimmerman MR, Gardner CE, et al. A pilot randomized controlled trial of the effects of cognitive-behavioral therapy for insomnia on sleep and daytime functioning in college students. *Behav Ther.* 2014;45(3):376-389.
- Zimmerman, MR. A randomized clinical trial of Cognitive-behavioral therapy for insomnia in a college student population. 2011. *Dissertation.*

Taylor2017
- Taylor DJ, Peterson AL, Pruiksma KE, et al. Internet and In-Person Cognitive Behavioral Therapy for Insomnia in Military Personnel: A Randomized Clinical Trial. *Sleep*. 2017;40(6):10.1093/sleep/zsx075.
- Pruiksma KE, Hale WJ, Mintz J, et al. Predictors of Cognitive Behavioral Therapy for Insomnia (CBTi) Outcomes in Active-Duty U.S. Army Personnel. *Behav Ther*. 2020;51(4):522-534.

Taylor2018
- Taylor DJ, Peterson AL, Pruiksma KE, et al. Impact of cognitive behavioral therapy for insomnia disorder on sleep and comorbid symptoms in military personnel: a randomized clinical trial. *Sleep*. 2018;41(6):10.1093/sleep/zsy069.
- NCT01549899

Ustinov2013
- Ustinov, Y. Treatment of insomnia in veterans with trauma-related disorders: A brief group cognitive-behavioral intervention. 2013. Dissertation.

VanDerZweerde2019
- van der Zweerde T, van Straten A, Effting M, Kyle SD, Lancee J. Does online insomnia treatment reduce depressive symptoms? A randomized controlled trial in individuals with both insomnia and depressive symptoms. *Psychol Med*. 2019;49(3):501-509.
- NTR6049

VanDerZweerde2020

© 2024 Furukawa Y et al. *JAMA Psychiatry.*

- Van der Zweerde T, Lancee J, Slottje P, Bosmans JE, Van Someren EJW, van Straten A. Nurse-Guided Internet-Delivered Cognitive Behavioral Therapy for Insomnia in General Practice: Results from a Pragmatic Randomized Clinical Trial. *Psychother Psychosom*. 2020;89(3):174-184.
- van der Zweerde T, Lancee J, Slottje P, et al. Cost-effectiveness of i-Sleep, a guided online CBT intervention, for patients with insomnia in general practice: protocol of a pragmatic randomized controlled trial. *BMC Psychiatry*. 2016;16:85.
- NTR 5202

VanStraten2014

- van Straten A, Emmelkamp J, de Wit J, et al. Guided Internet-delivered cognitive behavioural treatment for insomnia: a randomized trial. *Psychol Med*. 2014;44(7):1521-1532.
- NTR2132

Vedaa2020

- Vedaa Ø, Kallestad H, Scott J, et al. Effects of digital cognitive behavioural therapy for insomnia on insomnia severity: a large-scale randomised controlled trial. *Lancet Digit Health*. 2020;2(8):e397-e406.
- Kallestad H, Vedaa Ø, Scott J, et al. Overcoming insomnia: protocol for a large-scale randomised controlled trial of online cognitive behaviour therapy for insomnia compared with online patient education about sleep. *BMJ Open*. 2018;8(8):e025152.
- Vestergaard CL, Vedaa Ø, Simpson MR, et al. The effect of sleep-wake intraindividual variability in digital cognitive behavioral therapy for insomnia: a mediation analysis of a large-scale RCT. *Sleep*. 2021;44(10):zsab118.
- Kjørstad K, Sivertsen B, Vedaa Ø, et al. The effects of digital CBT-I on work productivity and activity levels and the mediational role of insomnia symptoms: Data from a randomized controlled trial with 6-month follow-up. *Behav Res Ther*. 2022;153:104083.
- NCT02558647

Verma2022

- Verma S, Quin N, Astbury L, et al. Treating postpartum insomnia: a three arm randomised controlled trial of cognitive behavioural therapy and light dark therapy [published online ahead of print, 2022 Sep 9]. Psychol Med. 2022;1-11.
- Verma S, Rajaratnam SMW, Davey M, Wiley JF, Bei B. Cognitive Behavioural Therapy and Light Dark Therapy for Maternal Postpartum Insomnia Symptoms: Protocol of a Parallel-Group Randomised Controlled Efficacy Trial. Front Glob Womens Health. 2021;1:591677.
- ACTRN12618000842268

Vincent2009

© 2024 Furukawa Y et al. *JAMA Psychiatry.*

- Vincent N, Lewycky S. Logging on for better sleep: RCT of the effectiveness of online treatment for insomnia. *Sleep*. 2009;32(6):807-815.
- Vincent N, Walsh K. Hyperarousal, sleep scheduling, and time awake in bed as mediators of outcome in computerized cognitive-behavioral therapy (cCBT) for insomnia. *Behav Res Ther*. 2013;51(3):161-166.
- Vincent N, Walsh K, Lewycky S. Sleep locus of control and computerized cognitive-behavioral therapy (cCBT). *Behav Res Ther.* 2010;48(8):779-783.

Vitiello2013

- Vitiello MV, McCurry SM, Shortreed SM, et al. Cognitive-behavioral treatment for comorbid insomnia and osteoarthritis pain in primary care: the lifestyles randomized controlled trial. *J Am Geriatr Soc*. 2013;61(6):947-956.
- Von Korff M, Vitiello MV, McCurry SM, et al. Group interventions for co-morbid insomnia and osteoarthritis pain in primary care: the lifestyles cluster randomized trial design. *Contemp Clin Trials*. 2012;33(4):759-768.
- Vitiello MV, McCurry SM, Shortreed SM, et al. Short-term improvement in insomnia symptoms predicts long-term improvements in sleep, pain, and fatigue in older adults with comorbid osteoarthritis and insomnia. *Pain*. 2014;155(8):1547-1554.
- Balderson BH, McCurry SM, Vitiello MV, Shortreed SM, Rybarczyk BD, Keefe FJ, Korff MV. Information without Implementation: A Practical Example for Developing a Best Practice Education Control Group. *Behav Sleep Med*. 2016 Sep-Oct;14(5):514-27.
- Koffel E, Vitiello MV, McCurry SM, Rybarczyk B, Von Korff M. Predictors of Adherence to Psychological Treatment for Insomnia and Pain: Analysis from a Randomized Trial. *Clin J Pain*. 2018;34(4):375-382.
- Koffel E, Kuhn E, Petsoulis N, et al. A randomized controlled pilot study of CBT-I Coach: Feasibility, acceptability, and potential impact of a mobile phone application for patients in cognitive behavioral therapy for insomnia. *Health Informatics J*. 2018;24(1):3-13.
- NCT01142349

Wagley2013

- Wagley JN, Rybarczyk B, Nay WT, Danish S, Lund HG. Effectiveness of abbreviated CBT for insomnia in psychiatric outpatients: sleep and depression outcomes. *J Clin Psychol*. 2013;69(10):1043-1055.
- Wagley, J Nile Efficacy of a brief intervention for insomnia among psychiatric outpatients. 2010. Dissertation.

Wang2016

- Wang J, Wei Q, Wu X, Zhong Z, Li G. Brief behavioral treatment for patients with treatment-resistant insomnia. *Neuropsychiatr Dis Treat*. 2016;12:1967-1975.

Watanabe2011

- Watanabe N, Furukawa TA, Shimodera S, et al. Brief behavioral therapy for refractory insomnia in residual depression: an assessor-blind, randomized controlled trial. *J Clin Psychiatry*. 2011;72(12):1651-1658.
- Shimodera S, Watanabe N, Furukawa TA, et al. Change in quality of life after brief behavioral therapy for insomnia in concurrent depression: analysis of the effects of a randomized controlled trial. *J Clin Sleep Med*. 2014;10(4):433-439.
- NCT00610259

Watanabe2023

- Watanabe Y, Kuroki T, Ichikawa D, Ozone M, Uchimura N, Ueno T. Effect of smartphone-based cognitive behavioral therapy app on insomnia: a randomized, double-blind study. *Sleep*. 2023;46(3):zsac270. doi:10.1093/sleep/zsac270
- jRCT2032210071

Wilkund2022

- Wiklund T, Molander P, Lindner P, Andersson G, Gerdle B, Dragioti E. Internet-Delivered Cognitive Behavioral Therapy for Insomnia Comorbid With Chronic Pain: Randomized Controlled Trial. *J Med Internet Res*. 2022;24(4):e29258.
- NCT03425942

Woldeamanuel2021

- Woldeamanuel YW, Blayney DW, Jo B, et al. Headache outcomes of a sleep behavioral intervention in breast cancer survivors: Secondary analysis of a randomized clinical trial. Cancer. 2021;127(23):4492-4503.
- NCT02165839

Wong2016

- Wong MY, Ree MJ, Lee CW. Enhancing CBT for Chronic Insomnia: A Randomised Clinical Trial of Additive Components of Mindfulness or Cognitive Therapy. *Clin Psychol Psychother*. 2016;23(5):377-385.
- Lee CW, Ree MJ, Wong MY. Effective Insomnia Treatments: Investigation of Processes in Mindfulness and Cognitive Therapy. *Behaviour Change*. 2018;35(2):71-90.

Wong2017

- Wong SY, Zhang DX, Li CC, et al. Comparing the Effects of Mindfulness-Based Cognitive Therapy and Sleep Psycho-Education with Exercise on Chronic Insomnia: A Randomised Controlled Trial. *Psychother Psychosom*. 2017;86(4):241-253.

Wong2021

© 2024 Furukawa Y et al. *JAMA Psychiatry.*

- Wong KY, Chung KF, Au CH. Low-Intensity Cognitive Behavioral Therapy for Insomnia as the Entry of the Stepped-Care Model in the Community: A Randomized Controlled Trial. *Behav Sleep Med*. 2021;19(3):378-394.
- NCT03736694

Wu2006

- Wu R, Bao J, Zhang C, Deng J, Long C. Comparison of sleep condition and sleep-related psychological activity after cognitive-behavior and pharmacological therapy for chronic insomnia. *Psychother Psychosom*. 2006;75(4):220-228.

Xing2020

- Xing J, Wu X, Liu H, et al. Effects of Electroacupuncture Therapy and Cognitive Behavioral Therapy in Chronic Insomnia: A Randomized Controlled Study. *Evid Based Complement Alternat Med*. 2020:5630130.

Yamamoto2016

- Yamamoto M, Somemura H, Nakamura S, et al. Efficacy of sleep education program based on principles of cognitive behavioral therapy to alleviate workers' distress. *Sleep and biological rhythms.* 2016;14(2):211-219.

Yang2022

- Yang YY, Jun S. The Effects of Cognitive Behavioral Therapy for Insomnia among College Students with Irritable Bowel Syndrome: A Randomized Controlled Trial. *Int J Environ Res Public Health*. 2022;19(21):14174. Published 2022 Oct 29. doi:10.3390/ijerph192114174

Yang2023

- Yang CM, Lu YL, Lee HC, et al. Can adding personalized rule-based feedback improve the therapeutic effect of self-help digital cognitive behavioral therapy for insomnia in young adults?. *Sleep Med*. 2023;107:36-45. doi:10.1016/j.sleep.2023.04.012

Zachariades2012

- Zachariades, Fotini. A CBT self-management approach for insomnia among people with chronic pain: A randomized controlled trial. 2013. Disseration.

Zachariae2018

- Zachariae R, Amidi A, Damholdt MF, et al. Internet-Delivered Cognitive-Behavioral Therapy for Insomnia in Breast Cancer Survivors: A Randomized Controlled Trial. *J Natl Cancer Inst.* 2018;110(8):880-887.
- NCT02444026

© 2024 Furukawa Y et al. *JAMA Psychiatry.*

Zakiei2021

- Zakiei A, Khazaie H, Rostampour M, et al. Acceptance and Commitment Therapy (ACT) Improves Sleep Quality, Experiential Avoidance, and Emotion Regulation in Individuals with Insomnia-Results from a Randomized Interventional Study. *Life (Basel)*. 2021;11(2):133.

Zhang 2015

- Zhang JX, Liu XH, Xie XH, et al. Mindfulness-based stress reduction for chronic insomnia in adults older than 75 years: a randomized, controlled, single-blind clinical trial. *Explore*. 2015;11(3):180-185.

Zhang2019

- Zhang H, Li Y, Li M, Chen X. A randomized controlled trial of mindfulness-based stress reduction for insomnia secondary to cervical cancer: Sleep effects. Appl Nurs Res. 2019;48:52-57.
- Zhang H, Li Y, Li M, Chen X. RETRACTED: A randomized controlled trial of mindfulness-based stress reduction for insomnia secondary to cervical cancer: Effects on sleep. *Cancer Rep (Hoboken)*. 2019;2(5):e1190.
- ChiCTR1800018571
- We rated the overall risk of bias due to the existence of the retracted paper above.

Zhang2023

- Zhang C, Liu Y, Guo X, Liu Y, Shen Y, Ma J. Digital Cognitive Behavioral Therapy for Insomnia Using a Smartphone Application in China: A Pilot Randomized Clinical Trial. *JAMA Netw Open.* 2023;6(3):e234866. Published 2023 Mar 1. doi:10.1001/jamanetworkopen.2023.4866
- NCT04779372

Zhao2020

- Zhao Y, Liu JE, Lewis FM, et al. Effects of mindfulness-based cognitive therapy on breast cancer survivors with insomnia: A randomised controlled trial. *Eur J Cancer Care (Engl)*. 2020;29(5):e13259.

Zhou2022a

- Zhou ES, Ritterband LM, Bethea TN, Robles YP, Heeren TC, Rosenberg L. Effect of Culturally Tailored, Internet-Delivered Cognitive Behavioral Therapy for Insomnia in Black Women: A Randomized Clinical Trial. *JAMA Psychiatry*. 2022;79(6):538-549.
- NCT03613519

Zhou2022b

© 2024 Furukawa Y et al. *JAMA Psychiatry.*

- Zhou K, Kong J, Wan Y, Zhang X, Liu X, Qu B, Wang B, Xue R. Positive impacts of e-aid cognitive behavioural therapy on the sleep quality and mood of nurses on site during the COVID-19 pandemic. *Sleep Breath*. 2022 Jan 8:1–5.

© 2024 Furukawa Y et al. *JAMA Psychiatry.*

eMethods 3. Examination of the imputation method

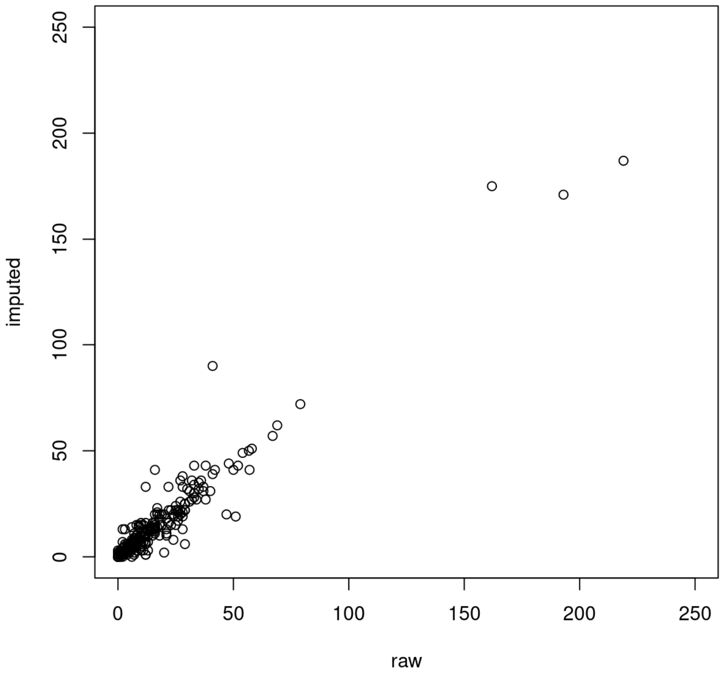

Single Score Intraclass Correlation

Model: twoway
Type : consistency

Subjects = 283
Raters = 2
ICC(C,1) = 0.956

F-Test, H0: r0 = 0 ; H1: r0 > 0
F(282,282) = 44.5 , p = 1.32e-152

95%-Confidence Interval for ICC Population Values:
0.945 < ICC < 0.965

© 2024 Furukawa Y et al. *JAMA Psychiatry.*

eMethods 4. The revised Cochrane risk of bias

4.1. Risk of Bias 2 interpretation

We evaluated the risk of bias about the primary outcome, not the study quality, using the revised Cochrane risk-of-bias tool for randomized trials (RoB2). Here, we describe how we interpreted the signalling questions in each domain.

Domain 1. Risk of bias arising from the randomization process

1.1 Was the allocation sequence random?

1.2 Was the allocation sequence concealed until participants were enrolled and assigned to interventions?

We excluded studies where sequence generation was not clearly random, or where the allocation was clearly not concealed, were excluded.

1.3 Did baseline differences between intervention groups suggest a problem with the randomization process?

We evaluated if there were baseline differences in age, gender, and the primary outcome measure.

Domain 2. Risk of bias due to deviations from intended interventions

2.1. Were participants aware of their assigned intervention during the trial?

2.2. Were carers and people delivering the interventions aware of participants' assigned intervention during the trial?

In most cases, yes.

2.3. Were there deviations from the intended intervention that arose because of the trial context?

The term “trial context” refers to effects of recruitment and engagement activities on trial participants and when trial personnel undermine the implementation of the trial protocol in ways that would not happen outside the trial (e.g. daily practice). We expect non-adherence to the active treatment to occur outside the trials, too. We rated Yes only when deviations were more often in non-active arms, such as waiting list, treatment as usual or no treatment.

2.4 Were these deviations likely to have affected the outcome?

Yes.

2.5 Were these deviations from intended intervention balanced between groups?

If the proportions of deviations from intended intervention differed more than 10%, we rated No.

2.6 Was an appropriate analysis used to estimate the effect of assignment to intervention?

We used ITT or mITT in the primary analysis so we rated Yes.

Domain 3. Risk of bias due to missing outcome data

3.1 Were data for this outcome available for all, or nearly all, participants randomized?

In the guidance document of RoB2, it is stated that the proportion required for dichotomous outcomes depends on the risk of the event. As we expect the control event rate of 10-20% and the experimental event rate of 40-60%, we decided to use 10% as the threshold.

© 2024 Furukawa Y et al. *JAMA Psychiatry.*

3.2 Is there evidence that the result was not biased by missing outcome data?

We rated yes when appropriate sensitivity analyses were conducted and they showed the result was not likely to be biased by missing outcome data.

3.3 Could missingness in the outcome depend on its true value?

Yes.

3.4 Is it likely that missingness in the outcome depended on its true value?

We rated yes when the proportions of missing data differed significantly (larger than 10%) between the arms or when the reasons for missing differed between the arms.

Domain 4. Risk of bias in measurement of the outcome

4.1 Was the method of measuring the outcome inappropriate?

Probably not.

4.2 Could measurement or ascertainment of the outcome have differed between intervention groups?

Probably not.

4.3 Were outcome assessors aware of the intervention received by study participants?

Yes.

4.4 Could assessment of the outcome have been influenced by knowledge of intervention received?

We rated no when more than two active comparators were used.

4.5 Is it likely that assessment of the outcome was influenced by knowledge of intervention received?

Probably not.

Domain 5. Risk of bias in selection of the reported result

5.1 Were the data that produced this result analyzed in accordance with a pre-specified analysis plan that was finalized before unblinded outcome data were available for analysis?

Is the numerical result being assessed likely to have been selected, on the basis of the results, from...

5.2. ... multiple eligible outcome measurements within the outcome domain?

5.3 ... multiple eligible analyses of the data?

We predefined in the protocol the hierarchy of outcome measures. We rated low risk of bias for the domain 5 when the trial reported the top priority outcome (ISI remission), or it reported ISI (continuous value) in a way that predefined in the statistical analysis plan. In most cases, details of statistical analysis plans were unavailable and we therefore rated some concerns.

© 2024 Furukawa Y et al. *JAMA Psychiatry.*

4.2 **The inter-rater reliability of the assessment of risk of bias**

Overall risk of bias

	Low	Some	High
Low	3	11	2
Some	16	59	32
High	2	25	39

Percentage agreement 53.4%

Weighed kappa 0.33

Domain 1. Risk of bias arising from the randomization process

	Low	Some	High
Low	72	12	3
Some	17	67	6
High	1	10	1

Percentage agreement 74.1%

Weighed kappa 0.55

Domain 2. Risk of bias due to deviations from intended interventions

	Low	Some	High
Low	143	18	9
Some	6	3	2
High	6	1	1

Percentage agreement 77.8%

Weighed kappa 0.13

Domain 3. Risk of bias due to missing outcome data

	Low	Some	High
Low	70	19	17
Some	15	14	5
High	10	16	23

Percentage agreement 56.6%

Weighed kappa 0.39

Domain 4. Risk of bias in measurement of the outcome

	Low	Some	High
Low	56	28	1
Some	26	73	1
High	2	1	1

Percentage agreement 68.8%

Weighed kappa 0.36

Domain 5. Risk of bias in selection of the reported result

	Low	Some	High
Low	59	30	1
Some	32	60	2
High	1	4	0

Percentage agreement 63.0%

Weighed kappa 0.31

© 2024 Furukawa Y et al. *JAMA Psychiatry.*

The overall agreement of the assessment was fair, which was not worse than the interrater reliability reported by systematic review experts.

- Minozzi S, Cinquini M, Gianola S, Gonzalez-Lorenzo M, Banzi R. The revised Cochrane risk of bias tool for randomized trials (RoB 2) showed low interrater reliability and challenges in its application. *J Clin Epidemiol*. 2020;126:37-44.

4.3. Risk of Bias 2 of each trial

trial	D1	D2	D3	D4	D5	Overall
Abbasi2016	S	L	L	S	S	S
Abdelaziz2021	S	L	S	S	S	H
Ahorsu2020	L	L	L	S	S	S
Alessi2016	L	L	L	L	H	H
Alshehri2020	L	L	L	L	S	S
Amra2022	L	H	H	S	S	H
Arnedt2011	S	L	H	L	L	H
Arnedt2021	S	L	L	L	L	S
Arnedt2023	L	L	L	L	L	L
Ashworth2015	S	L	L	S	L	S
Ayabe2018	L	L	L	S	L	S
Ballou2020	L	L	H	S	S	H
Bastein2004	S	L	L	L	S	S
Behera2023	S	L	L	L	S	S
Bernstein2017	S	H	H	S	S	H
Bothelius2013	L	L	H	S	H	H
Bramoweth2020	L	L	H	L	L	H
Broomfield2003	S	L	S	H	S	H
Buysse2011	S	L	L	S	S	S
Cai2022	L	L	L	S	L	S
Cape2016	L	L	S	L	L	S
Carney2017	S	L	H	L	L	H
Carrera1980	S	L	L	S	S	S
Casault2015	L	L	H	S	L	H
Chakravorty2019	L	L	L	S	L	S
Chan2021	L	L	H	S	L	H
Chao2021	L	L	S	S	L	S

© 2024 Furukawa Y et al. *JAMA Psychiatry.*

trial	D1	D2	D3	D4	D5	Overall
Chapoutot2020	L	L	L	S	S	S
Cheng2019	L	L	H	L	L	H
Christensen2016	L	L	L	L	L	L
Chung2023	S	L	L	S	L	S
Craner2022	L	L	H	L	L	H
Currie2000	S	L	L	S	S	S
Currie2004	S	S	S	L	S	H
Daren2012	H	L	L	L	H	H
Dean2019	S	L	L	L	S	S
Dekker2020	L	L	L	L	S	S
Dirksen2008	S	L	L	S	S	S
Drake2019	L	L	L	H	L	H
Dyrberg2022	L	L	H	S	L	H
Edinger2001	S	L	L	L	S	S
Edinger2003	S	L	H	S	S	H
Edinger2005	S	L	H	L	S	H
Edinger2007	L	H	H	S	S	H
Edinger2009	S	L	L	L	L	S
ElRafahiFerreira2021	L	L	H	L	S	H
ElRafahiFerreira2022	L	L	L	L	L	L
Epstein2007	S	L	S	L	S	S
Eptein2012	S	L	S	L	L	S
Espie1989	S	L	S	L	S	S
Espie2001	S	H	H	S	S	H
Espie2007	L	L	L	S	S	S
Espie2008	L	L	S	S	S	S
Espie2012	L	L	L	L	S	S
Espie2019	L	L	L	S	L	S
Falloon2015	L	L	L	S	S	S
Farsani2021	L	L	L	S	S	S
Felder2020	L	L	S	S	L	S
Fernando2013	L	L	L	L	S	S
Feuerstein2017	S	L	S	L	L	S
Ford2022	L	L	L	S	L	S
Fowler2009	L	L	L	S	S	S
Freeman2015	L	L	L	S	L	S
Freeman2017	L	L	L	S	L	S

© 2024 Furukawa Y et al. *JAMA Psychiatry.*

trial	D1	D2	D3	D4	D5	Overall
Fucito2014	H	L	S	L	S	H
Garcia2018	L	S	S	S	L	S
Garland2014	L	L	L	L	L	L
Gebara2019	S	L	L	S	S	S
Gehrman2020	L	L	L	L	S	S
Gehrman2021	L	L	L	L	S	S
Germain2014	S	L	S	S	L	S
Gieselmann2019	S	L	S	L	S	S
Glozier2019	L	L	L	L	L	L
Greeff1998	S	L	L	S	S	S
Guarnaccia2023	S	L	S	S	S	H
Hagatun2019	S	L	L	L	L	S
Hall2022	S	L	L	L	S	S
Ham2020	S	L	H	S	S	H
Harris2012	S	L	L	L	S	S
Harris2019	S	L	L	L	S	S
Harvey2014	L	L	L	L	L	L
Harvey2015	L	L	S	L	L	S
Ho2014	L	L	S	S	S	S
Ho2021	L	L	S	S	L	S
Holmqvist2014	S	L	L	L	S	S
Horsch2017	L	L	H	S	L	H
Hou2014	S	H	L	S	S	H
Jacobs1993	S	L	L	L	S	S
Jacobs2004	S	L	H	L	S	H
JanssonFrojmark2012a	L	L	L	L	L	L
JanssonFrojmark2012b	L	L	L	S	L	S
Jarnefelt2020	L	L	H	L	S	H
Javaheri2020	S	L	S	S	S	H
Jernelov2012	S	L	L	S	L	S
Johann2020	S	L	L	S	S	S
Jungquist2010	S	L	L	L	S	S
Kaku2012	S	H	L	L	S	H
Kaldo2015	S	L	L	L	L	S
Kaldo2020	S	L	H	S	L	H
Kallestad2021	S	L	L	L	L	S
Kalmbach2020	S	L	L	L	L	S

© 2024 Furukawa Y et al. *JAMA Psychiatry.*

trial	D1	D2	D3	D4	D5	Overall
Kapella2011	S	L	L	L	S	S
Kapella2022	S	L	L	L	L	S
Kennet2021	S	L	H	S	L	H
Krieger2019	L	L	H	S	L	H
Kuhn2022	L	L	L	S	S	S
Kyle2020	L	L	H	S	L	H
Lami2018	S	L	S	L	S	S
Lancee2012	S	L	H	S	S	H
Lancee2013	S	L	S	L	L	S
Lancee2015	S	L	L	S	L	S
Lancee2016	S	L	L	S	L	S
Lappalainen2019	S	S	H	S	S	H
Latocha2022	L	H	L	L	L	H
Leerssen2022	L	L	L	L	S	S
Lichstein2000	S	H	H	S	S	H
Lichstein2001	S	L	H	L	S	H
Liu2022	L	L	S	S	S	S
Lopez2019	S	L	H	L	S	H
Lorenz2019	S	L	L	S	L	S
Lovato2014	S	L	L	S	L	S
Low2020	S	L	L	L	S	S
Maguen2021	L	L	L	L	L	L
Majd2020	S	L	L	L	L	S
Manber2008	L	L	L	L	L	L
Manber2016	L	L	H	L	L	H
Manber2019	L	L	L	L	L	L
Mao2018	S	L	L	L	S	S
Marino2001	S	S	H	S	L	H
Marks2023	L	L	L	L	L	L
Martinez2014	L	L	S	S	S	S
Matthews2014	L	L	S	L	L	S
McCrae2007	S	L	L	S	S	S
McCrae2019	L	L	S	S	S	S
McCrae2020	L	L	H	S	S	H
McCurry2016	S	L	H	L	L	H
McCurry2021	L	L	S	L	L	S
Means2000	S	L	L	S	S	S

© 2024 Furukawa Y et al. *JAMA Psychiatry.*

trial	D1	D2	D3	D4	D5	Overall
Milby1993	S	L	L	L	S	S
Miller2021	S	L	H	L	L	H
Miller2023	L	L	L	L	L	L
Mimeault1999	S	L	H	S	L	H
Miro2011	S	L	S	S	S	H
Morawetz1989	S	S	H	S	S	H
Morgan2012	H	L	H	S	L	H
Morin1988	S	L	L	S	S	S
Morin1993	S	L	L	S	S	S
Morin1999	S	L	H	S	L	H
NCT00127790-1	S	L	L	S	L	S
NCT01011218-2	S	S	S	L	L	S
NCT01987089	L	L	L	L	L	L
NCT02613364	S	L	S	L	L	S
NCT03964974	S	S	H	L	L	H
NorellClarke2015	L	L	H	L	L	H
Okajima2020	L	L	H	S	L	H
Okajima2021	S	L	S	S	L	S
Ong2018	L	L	H	S	L	H
Ong2020	L	L	S	S	L	S
Oswald2022	L	L	L	S	L	S
Padron2022	S	L	H	S	S	H
Palesh2018	S	L	H	S	L	H
Palesh2020	S	L	L	L	L	S
Patel2017	L	L	H	S	L	H
Perlis2004	S	L	H	L	S	H
Perrault2022	S	L	H	S	L	H
Pigeon2017	S	L	S	S	L	S
Pigeon2019	S	L	S	S	L	S
Pigeon2022	L	L	H	S	L	H
Pillai2015	S	L	H	S	L	H
Prados2020	S	L	S	S	S	H
Quintiliani2020	S	L	L	S	L	S
Redeker2015	S	L	S	L	L	S
Redeker2022	L	L	L	L	L	L
Ritterband2009	S	L	L	S	L	S
Ritterband2012	S	L	L	S	S	S

© 2024 Furukawa Y et al. *JAMA Psychiatry.*

trial	D1	D2	D3	D4	D5	Overall
Ritterband2017	L	L	L	L	L	L
Robabeh2015	S	L	L	L	S	S
Roscoe2015	L	L	H	L	S	H
Rosen2000	S	L	H	S	S	H
Rybarczyk2005	L	L	L	L	S	S
Sadler2018	L	L	L	L	L	L
Sandlund2017	L	L	S	S	S	S
Sato2019	S	L	L	S	S	S
Sato2022	S	L	H	S	S	H
Savard2005	S	L	H	S	L	H
Savard2014	L	L	H	S	S	H
Schuffelen2023	L	L	L	S	L	S
Sgoifo2017	L	L	L	S	S	S
Sheaves2018	L	L	L	L	L	L
Shimamoto2022	L	L	L	S	S	S
Shin2023	L	L	H	L	L	H
Short2021	S	L	S	S	S	H
Siebmnns2021	L	L	L	S	S	S
Siengsukon2020	S	L	L	L	S	S
Siengsukon2021	S	L	L	L	S	S
Simeit2004	S	L	L	L	H	H
Smith2015	S	L	L	L	S	S
Smitherman2016	L	L	S	L	S	S
Soeffing2008	S	S	L	L	H	H
Song2020	S	H	H	L	S	H
Speed2022	L	L	H	L	L	H
Store2022	L	L	L	S	L	S
Strom2004	S	L	H	S	S	H
Sunnheld2020	S	L	L	L	L	S
Sveen2021	L	L	S	L	L	S
Sweetman2019	S	L	L	S	H	H
Talbot2014	L	L	L	S	L	S
Taylor2014	L	L	S	S	L	S
Taylor2017	S	L	L	L	S	S
Taylor2018	S	L	L	S	S	S
Ustinov2013	S	L	H	S	H	H
VanDerZweerde2019	L	L	S	S	S	S

© 2024 Furukawa Y et al. *JAMA Psychiatry.*

trial	D1	D2	D3	D4	D5	Overall
VanDerZweerde2020	L	L	S	S	L	S
VanStraten2014	L	L	L	S	S	S
Vedaa2020	L	L	S	L	L	S
Verma2022	L	L	L	S	L	S
Vincent2009	S	L	L	S	S	S
Vitiello2013	S	L	L	L	L	S
Wagley2013	S	L	L	S	S	S
Wang2016	L	L	L	L	S	S
Watanabe2011	L	L	L	S	L	S
Watanabe2023	L	L	L	L	L	L
Wilkund2022	L	L	S	L	S	S
Woldeamanuel2021	L	L	L	L	L	L
Wong2016	S	L	L	L	S	S
Wong2017	L	L	L	L	L	L
Wong2021	L	L	H	L	L	H
Wu2006	S	L	S	L	S	S
Xing2020	L	L	L	L	L	L
Yamamoto2016	S	L	S	S	S	H
Yang2022	S	L	L	S	S	S
Yang2023	S	L	H	S	S	H
Zachariades2012	S	L	H	S	S	H
Zachariae2018	L	L	L	S	L	S
Zakiei2021	S	L	S	L	S	S
Zhang2015	S	L	L	S	S	S
Zhang2019	S	L	L	S	S	H
Zhang2023	S	L	L	L	S	S
Zhao2020	L	L	L	S	L	S
Zhou2022a	S	L	L	L	L	S
Zhou2022b	S	L	S	S	S	H

D1 = Risk of bias arising from the randomization process; D2 = Risk of bias due to deviations from intended interventions; D3 = Risk of bias due to missing outcome data; D4 = Risk of bias in measurement of the outcome; D5 = Risk of bias in selection of the reported result; H = high risk; L = low risk; S = some concern.
The overall risk of bias of the primary outcome for a study was judged “low” only when the study was judged to be at low risk of bias for ALL the five domains of potential biases arising from the randomization process, due to deviations from intended interventions, due to missing

© 2024 Furukawa Y et al. *JAMA Psychiatry.*

outcome data, in measurement of the outcome or selection of reported results, “some concerns” when one to three domains were judged to have some concerns and none at high risk, and “high” when the study was judged to be at high risk in one or more domains or to have some concerns in four or more domains

© 2024 Furukawa Y et al. *JAMA Psychiatry.*

eMethods 5. Assessment of inconsistency for the primary outcome

Statistical heterogeneity of the treatment-level NMA was estimated to be $\tau\tau 2$=0.23. The global, design-by-treatment test for inconsistency gave Q=38.08, with 32 degrees of freedom, p-value=0.21. The local approach to inconsistency (back-calculation method) gave the following results:

```
> # local
> netsplit(net_nma) # here you can check which (and how many) loops show inconsistencies between direct and indirect TEs.
```

Separate indirect from direct evidence (SIDE) using back-calculation method

Random effects model:

comparison	k	prop	nma	direct	indir.	RoR	z	p-value
BT:CBT	7	0.20	0.6593	0.5670	0.6840	0.8290	-0.58	0.5597
BT:CT	2	0.31	1.0018	1.3145	0.8848	1.4855	0.77	0.4408
BT:NT	0	0	2.4198	.	2.4198	.	.	.
BT:PE	25	0.45	2.4972	3.4942	1.8979	1.8411	2.29	0.0219
BT:PLB	0	0	3.7586	.	3.7586	.	.	.
BT:RT	4	0.31	1.9548	2.5841	1.7273	1.4961	0.84	0.3997
BT:TAU	2	0.29	1.7778	1.5835	1.8640	0.8495	-0.28	0.7803
BT:WL	22	0.51	3.7827	3.0080	4.7980	0.6269	-1.76	0.0790
CBT:CT	6	0.37	1.5195	2.3620	1.1736	2.0126	1.51	0.1319
CBT:NT	3	0.90	3.6704	3.1675	14.0191	0.2259	-1.16	0.2443
CBT:PE	76	0.86	3.7879	3.5367	5.8504	0.6045	-2.05	0.0399
CBT:PLB	3	0.58	5.7012	6.3044	4.9581	1.2715	0.28	0.7776
CBT:RT	5	0.40	2.9651	3.7418	2.5379	1.4744	0.91	0.3616
CBT:TAU	6	0.74	2.6966	2.9621	2.0562	1.4406	0.64	0.5238
CBT:WL	67	0.79	5.7377	6.0149	4.8019	1.2526	0.92	0.3580
CT:NT	1	0.13	2.4155	8.8000	1.9883	4.4259	1.16	0.2443
CT:PE	3	0.17	2.4928	3.5629	2.3186	1.5367	0.70	0.4862
CT:PLB	2	0.49	3.7519	3.3223	4.2244	0.7865	-0.28	0.7776
CT:RT	1	0.09	1.9513	1.2000	2.0492	0.5856	-0.53	0.5968
CT:TAU	0	0	1.7746	.	1.7746	.	.	.
CT:WL	5	0.25	3.7759	8.3082	2.8877	2.8771	1.98	0.0480
NT:PE	0	0	1.0320	.	1.0320	.	.	.
NT:PLB	0	0	1.5533	.	1.5533	.	.	.
NT:RT	0	0	0.8078	.	0.8078	.	.	.

© 2024 Furukawa Y et al. *JAMA Psychiatry.*

NT:TAU	0	0	0.7347	.	0.7347	.	.	.
NT:WL	0	0	1.5632	.	1.5632	.	.	.
PLB:PE	0	0	0.6644	.	0.6644	.	.	.
RT:PE	5	0.26	1.2775	1.9158	1.1083	1.7286	1.12	0.2633
TAU:PE	1	0.05	1.4047	4.5000	1.3133	3.4264	1.07	0.2824
WL:PE	3	0.03	0.6602	0.2327	0.6802	0.3422	-1.43	0.1522
PLB:RT	0	0	0.5201	.	0.5201	.	.	.
PLB:TAU	0	0	0.4730	.	0.4730	.	.	.
PLB:WL	0	0	1.0064	.	1.0064	.	.	.
RT:TAU	0	0	0.9095	.	0.9095	.	.	.
RT:WL	5	0.21	1.9351	2.2117	1.8674	1.1844	0.32	0.7511
TAU:WL	0	0	2.1278	.	2.1278	.	.	.

Legend:

comparison - Treatment comparison

k - Number of studies providing direct evidence

prop - Direct evidence proportion

nma - Estimated treatment effect (OR) in network meta-analysis

direct - Estimated treatment effect (OR) derived from direct evidence

indir. - Estimated treatment effect (OR) derived from indirect evidence

RoR - Ratio of Ratios (direct versus indirect)

z - z-value of test for disagreement (direct versus indirect)

p-value - p-value of test for disagreement (direct versus indirect)

© 2024 Furukawa Y et al. *JAMA Psychiatry.*

eTable 1. Components and delivery formats that are included in CBT

	**Components**	CBT n=210
		n (%)
Educational components	Sleep hygiene education	198 (94)
	Sleep diary	192 (91)
Cognitive components	Cognitive restructuring	199 (95)
	Third wave components	44 (21)
	Constructive worry	21 (10)
Behavioral components	Sleep restriction	198 (94)
	Stimulus control	194 (92)
	Paradoxical intention	18 (9)
	Relaxation	139 (66)
Others	Non-specific treatment effect	210 (100)
	Waiting component	3 (1)
Delivery methods	Individual	91 (43)
	Group	54 (26)
	Face-to-face	121 (58)
	Online therapeutic guidance	31 (15)
	Human encouragement	36 (17)
	Automatic encouragement	30 (14)

© 2024 Furukawa Y et al. *JAMA Psychiatry.*

eTable 2. Characteristics of included trials

We found 206 two-arm trials; 26 three-arm trials; 7 four-arm trials; 1 five-arm trial; and 1 six-arm trial. We divided 5 four-arm trials into 2 two-armed trials each in the analysis so that concomitant treatments were equally distributed among arms. We divided a five-arm trial into two trials in the analysis because it reported different outcome measures for subgroups.

trial	Arm	treatment	Year	Country	Age_m	Age_sd	Age_n	F_n	Insomnia diagnosis	Comorbidities	n	r
Abbasi2016	Intervention	CBT	2016	Iran	35.3	5.3	36	36	2. informal_PSQI	3. phy_others	36	2
Abbasi2016	Control	WL	2016	Iran	33.2	8.9	36	36	2. informal_PSQI	3. phy_others	36	0
Abdelaziz2021	Study	CBT	2021	Saudi Arabia	53.9	4.1	40	40	2. informal_ISI	3. phy_menopause	49	16
Abdelaziz2021	Control	PE	2021	Saudi Arabia	52.2	4.3	40	40	2. informal_ISI	3. phy_menopause	49	6
Ahorsu2020	CBT-I	CBT	2020	Iran	38.4	13.5	160	97	2. informal_ISI	3. phy_others	160	9
Ahorsu2020	PE	WL	2020	Iran	38.0	9.9	160	90	2. informal_ISI	3. phy_others	160	2
Alessi2016	Individual	CBT	2016	USA	72.1	7.9	54	2	1. formal_ICSD	4. psy_phy_mixed	54	33
Alessi2016	Group	CBT	2016	USA	72.1	7.9	52	2	1. formal_ICSD	4. psy_phy_mixed	52	32
Alessi2016	Control	PE	2016	USA	72.4	7.3	53	1	1. formal_ICSD	4. psy_phy_mixed	53	21
Alshehri2020	CBT-I	CBT	2020	USA	61.9	6.5	14	10	2. informal_ISI	3. phy_diabetes	14	8
Alshehri2020	HE	PE	2020	USA	59.4	9.5	14	7	2. informal_ISI	3. phy_diabetes	14	2
Amra2022	Intervention	CBT	2022	Iran	34.6	9.5	31	25	2. informal_ISI	4. psy_phy_mixed	32	18
Amra2022	Control	NT	2022	Iran	36.6	6.9	26	17	2. informal_ISI	4. psy_phy_mixed	32	4
Arnedt2011	CBTI-AD	CBT	2011	USA	46.2	8.9	9	3	2. informal_ISI	2. psy_substance_dependence	9	5
Arnedt2011	BPT	PE	2011	USA	46.1	12.0	8	3	2. informal_ISI	2. psy_substance_dependence	8	2
Arnedt2021	CBT-TM	CBT	2021	USA	43.7	17.4	33	23	1. formal_ICSD	4. psy_phy_mixed	33	14

© 2024 Furukawa Y et al. *JAMA Psychiatry.*

trial	Arm	treatment	Year	Country	Age_m	Age_sd	Age_n	F_n	Insomnia diagnosis	Comorbidities	n	r
Arnedt2021	CBT-F2F	CBT	2021	USA	50.9	14.5	32	23	1. formal_ICSD	4. psy_phy_mixed	32	16
Arnedt2023	CBT-CB-TM	CBT	2023	USA	36.7	12.0	30	23	2. informal_ISI	2. psy_substance_dependence	30	13
Arnedt2023	SHE-TM	PE	2023	USA	38.7	13.7	27	20	2. informal_ISI	2. psy_substance_dependence	27	4
Ashworth2015	CBT-I	CBT	2015	Australia	37.1	12.8	21	13	1. formal_DSM	2. psy_MDD	21	12
Ashworth2015	Self-help	CBT	2015	Australia	36.4	13.8	20	12	1. formal_DSM	2. psy_MDD	21	2
Ayabe2018	CBT-I + TAU	BT	2018	Japan	61.2	13.8	24	14	1. formal_DSM	0. primary_insomnia	24	6
Ayabe2018	TAU	WL	2018	Japan	58.5	16.6	27	16	1. formal_DSM	0. primary_insomnia	27	2
Ballou2020	BBT	BT	2020	USA	49.0	18.0	10	9	2. informal_ISI	3. phy_others	13	4
Ballou2020	Control	WL	2020	USA	49.0	18.0	12	10	2. informal_ISI	3. phy_others	12	0
Bastein2004	Group	CBT	2004	Canada	40.0	10.4	16	11	3. others	0. primary_insomnia	16	10
Bastein2004	Telephone	CBT	2004	Canada	41.6	9.5	14	7	3. others	0. primary_insomnia	14	4
Bastein2004	Individual	CBT	2004	Canada	43.8	10.0	15	11	3. others	0. primary_insomnia	15	7
Behera2023	HMBCT	CBT	2023	India	25	4.68	26	0	1. formal_Edinger2004	1. primary_secondary_mixed	26	16
Behera2023	Control	BT	2023	India	24	3.01	22	0	1. formal_Edinger2004	1. primary_secondary_mixed	22	0
Bernstein2017	Intervention	CBT	2017	USA	54.9	13.0	43	38	1. formal_DSM	0. primary_insomnia	43	15
Bernstein2017	Control	WL	2017	USA	53.6	12.3	45	36	1. formal_DSM	0. primary_insomnia	45	2
Bothelius2013	CBTI	CBT	2013	Sweden	48.1	13.2	40	33	1. formal_Edinger2004	1. primary_secondary_mixed	40	12

© 2024 Furukawa Y et al. *JAMA Psychiatry.*

trial	Arm	treatment	Year	Country	Age_m	Age_sd	Age_n	F_n	Insomnia diagnosis	Comorbidities	n	r
Bothelius2013	WL	WL	2013	Sweden	53.0	9.4	38	30	1. formal_Edinger2004	1. primary_secondary_mixed	38	2
Bramoweth2020	BBTI	BT	2020	USA	56.8	13.7	31	2	1. formal_DSM	1. primary_secondary_mixed	31	7
Bramoweth2020	CBTI	CBT	2020	USA	53.4	15.2	32	4	1. formal_DSM	1. primary_secondary_mixed	32	4
Broomfield2003	PI	BT	2003	UK	26.0	*	17	9	1. formal_ICSD	0. primary_insomnia	17	6
Broomfield2003	C	PE	2003	UK	24.4	*	17	10	1. formal_ICSD	0. primary_insomnia	17	3
Buysse2011	BBTI	BT	2011	USA	72.5	6.6	39	26	1. formal_DSM	0. primary_insomnia	42	35
Buysse2011	IC	WL	2011	USA	70.8	7.8	40	28	1. formal_DSM	0. primary_insomnia	40	35
Cai2022	Mindfulness	CT	2022	China	80	8.0	38	25	2. informal_PSQI	2. psy_others	38	33
Cai2022	Health education	WL	2022	China	80	10.8	37	31	2. informal_PSQI	2. psy_others	37	19
Cape2016	CBT-I	CBT	2016	UK	42.2	14.9	119	64	3. others	2. psy_mixed	119	48
Cape2016	TAU	PE	2016	UK	42.2	13.5	120	79	3. others	2. psy_mixed	120	35
Carney2017	CBT+AD	CBT	2017	Canada, USA	45.0	9.4	36	20	1. formal_Edinger2004	2. psy_MDD	36	8
Carney2017	AD+SH	PE	2017	Canada, USA	40.1	11.4	35	22	1. formal_Edinger2004	2. psy_MDD	35	2
Carrera1980	Ocean tape	RT	1980	USA	*	*	*	5	3. others	0. primary_insomnia	16	5
Carrera1980	Wait-control	WL	1980	USA	*	*	*	12	3. others	0. primary_insomnia	23	4
Casault2015	mCBT-I	CBT	2015	Canada	56.9	10.8	20	19	2. informal_ISI	3. phy_cancer	20	14
Casault2015	CTL	WL	2015	Canada	57.0	9.4	18	16	2. informal_ISI	3. phy_cancer	18	4

© 2024 Furukawa Y et al. *JAMA Psychiatry.*

trial	Arm	treatment	Year	Country	Age_m	Age_sd	Age_n	F_n	Insomnia diagnosis	Comorbidities	n	r
Chakravorty2019	CBT-I	CBT	2019	USA	52.0	7.0	11	0	2. informal_ISI	2. psy_substance_dependence	11	7
Chakravorty2019	MO	PE	2019	USA	56.0	6.0	11	0	2. informal_ISI	2. psy_substance_dependence	11	2
Chan2021	Tx	CBT	2021	Hong Kong	27.3	7.3	167	109	1. formal_ICD	2. psy_MDD	167	25
Chan2021	WL	WL	2021	Hong Kong	27.3	7.2	153	124	1. formal_ICD	2. psy_MDD	153	6
Chao2021	CBT-I	CBT	2021	USA	55.0	7.0	39	11	1. formal_DSM	2. psy_others	39	12
Chao2021	Waitlist	WL	2021	USA	54.0	6.0	46	10	1. formal_DSM	2. psy_others	46	1
Chapoutot2020	ACT-E-CBT	CBT	2020	France	48.0	10.0	15	12	1. formal_ICSD	2. psy_substance_dependence	16	4
Chapoutot2020	WLC	WL	2020	France	48.0	10.0	15	12	1. formal_ICSD	2. psy_substance_dependence	16	0
Cheng2019	dCBT-I	CBT	2019	USA	44.5	15.8	358	279	1. formal_DSM	1. primary_secondary_mixed	946	193
Cheng2019	Sleep education	PE	2019	USA	45.7	15.1	300	240	1. formal_DSM	1. primary_secondary_mixed	439	42
Christensen2016	SHUTi	CBT	2016	Australia	43.0	12.2	574	422	3. others	0. primary_insomnia	574	293
Christensen2016	HealthWatch	PE	2016	Australia	42.5	12.2	575	423	3. others	0. primary_insomnia	575	123

© 2024 Furukawa Y et al. *JAMA Psychiatry.*

trial	Arm	treatment	Year	Country	Age_m	Age_sd	Age_n	F_n	Insomnia diagnosis	Comorbidities	n	r
Chung2023	mindfulness	CT	2023	Hong Kong	47.95	11.63	102	78	1. formal_DSM	2. psy_others	102	9
Chung2023	Health education	WL	2023	Hong Kong	45.63	11.75	98	66	1. formal_DSM	2. psy_others	98	9
Craner2022	IPRP-UC	CBT	2022	USA	48.8	16.7	24	20	2. informal_ISI	3. phy_others	24	7
Craner2022	IPRP+CBT-I	CBT	2022	USA	53.4	15.0	26	21	2. informal_ISI	3. phy_others	55	4
Currie2000	CBT	CBT	2000	Canada	45.0	8.0	32	*	1. formal_DSM	4. psy_phy_mixed	32	7
Currie2000	WLC	WL	2000	Canada	45.0	8.0	28	*	1. formal_DSM	4. psy_phy_mixed	28	1
Currie2004	Self-help manual	CBT	2004	Canada	43.3	10.9	20	6	1. formal_DSM	2. psy_substance_dependence	20	3
Currie2004	Individual therapy	CBT	2004	Canada	43.3	10.9	20	6	1. formal_DSM	2. psy_substance_dependence	20	6
Currie2004	Wait-list	WL	2004	Canada	43.3	10.9	20	6	1. formal_DSM	2. psy_substance_dependence	20	1
Daren2012	Intervention	BT	2012	USA	52.2	18.8	5	3	1. formal_DSM	4. psy_phy_mixed	5	3
Daren2012	Control	PE	2012	USA	52.2	18.8	4	2	1. formal_DSM	4. psy_phy_mixed	4	1
Dean2019	Experimental	BT	2019	USA	65.6	7.3	16	10	2. informal_ISI	3. phy_cancer	21	9
Dean2019	Control	PE	2019	USA	65.9	8.1	14	9	2. informal_ISI	3. phy_cancer	19	1
Dekker2020	ICBTI w 1-4	CBT	2020	Netherlands	51.3	11.5	87	65	1. formal_DSM	1. primary_secondary_mixed	87	16
Dekker2020	ICBTI w 6-9	WL	2020	Netherlands	50.6	11.0	88	73	1. formal_DSM	1. primary_secondary_mixed	88	3

© 2024 Furukawa Y et al. *JAMA Psychiatry.*

trial	Arm	treatment	Year	Country	Age_m	Age_sd	Age_n	F_n	Insomnia diagnosis	Comorbidities	n	r
Dirksen2008	CBT-I	BT	2008	USA	57.2	9.9	34	40	3. others	3. phy_cancer	40	3
Dirksen2008	CC	PE	2008	USA	59.2	10.7	38	41	3. others	3. phy_cancer	41	2
Drake2019	SRT	BT	2019	USA	56.8	5.4	50	50	1. formal_DSM	3. phy_menopause	52	19
Drake2019	CBTI	CBT	2019	USA	55.3	5.9	50	50	1. formal_DSM	3. phy_menopause	52	27
Drake2019	SHE	PE	2019	USA	57.2	5.6	50	50	1. formal_DSM	3. phy_menopause	50	2
Dyrberg2022	CBT-I	CBT	2022	Denmark	36.1	*	22	14	1. formal_DSM	2. psy_MDD	23	3
Dyrberg2022	TAU	WL	2022	Denmark	38.3	*	19	14	1. formal_DSM	2. psy_MDD	24	2
Edinger2001	CBT	CBT	2001	USA	55.8	12.2	25	11	1. formal_DSM	0. primary_insomnia	25	16
Edinger2001	Placebo therapy	PE	2001	USA	55.7	9.5	25	13	1. formal_DSM	0. primary_insomnia	25	5
Edinger2001	Relaxation training	RT	2001	USA	54.5	10.2	25	11	1. formal_DSM	0. primary_insomnia	25	8
Edinger2003	ACBT	BT	2003	USA	51.0	13.7	10	1	1. formal_DSM	0. primary_insomnia	10	2
Edinger2003	SHC	PE	2003	USA	51.0	13.7	10	1	1. formal_DSM	0. primary_insomnia	10	0
Edinger2005	CBT	CBT	2005	USA	50.1	6.9	18	17	1. formal_DSM	3. phy_fibromyalgia	18	10
Edinger2005	SH	PE	2005	USA	46.5	9.0	18	17	1. formal_DSM	3. phy_fibromyalgia	18	13
Edinger2005	UC	WL	2005	USA	48.3	9.1	11	11	1. formal_DSM	3. phy_fibromyalgia	11	2
Edinger2007	CBT combined	BT	2007	USA	55.9	10.0	75	39	1. formal_DSM	0. primary_insomnia	75	32
Edinger2007	WL	WL	2007	USA	52.4	7.3	11	4	1. formal_DSM	0. primary_insomnia	11	0
Edinger2009	CBT	CBT	2009	USA	54.4	13.9	41	6	1. formal_Edinger2004	1. primary_secondary_mixed	41	19
Edinger2009	SH	PE	2009	USA	54.0	13.7	40	5	1. formal_Edinger2004	1. primary_secondary_mixed	40	10

© 2024 Furukawa Y et al. *JAMA Psychiatry.*

trial	Arm	treatment	Year	Country	Age_m	Age_sd	Age_n	F_n	Insomnia diagnosis	Comorbidities	n	r
ElRafahiFerreira2021	ACT-BBI-I	CBT	2021	Brazil	40.2	10.5	22	13	1. formal_DSM	1. primary_secondary_mixed	22	5
ElRafahiFerreira2021	CBT-I	CBT	2021	Brazil	40.2	10.5	23	14	1. formal_DSM	1. primary_secondary_mixed	23	8
ElRafahiFerreira2022	CBT-I	CBT	2022	Brazil	43.3	10.0	17	12	1. formal_DSM	1. primary_secondary_mixed	18	10
ElRafahiFerreira2022	ACT-I	CT	2022	Brazil	44.2	11.4	18	14	1. formal_DSM	1. primary_secondary_mixed	19	4
Epstein2007	Multicomponent intervention	BT	2007	USA	57.1	9.8	34	34	1. formal_DSM	3. phy_cancer	40	4
Epstein2007	Control	PE	2007	USA	59.1	10.6	38	38	1. formal_DSM	3. phy_cancer	41	8
Eptein2012	SCT	BT	2012	USA	71.0	8.3	44	31	3. others	0. primary_insomnia	44	11
Eptein2012	SRT	BT	2012	USA	68.0	8.3	44	25	3. others	0. primary_insomnia	44	8
Eptein2012	MCI	BT	2012	USA	67.2	6.6	41	27	3. others	0. primary_insomnia	41	16
Eptein2012	WLC	WL	2012	USA	69.5	8.3	50	29	3. others	0. primary_insomnia	50	2
Espie1989	Paradoxical intention	BT	1989	UK	43.7	15.8	15	12	3. others	0. primary_insomnia	18	6
Espie1989	Stimulus control	BT	1989	UK	44.9	14.4	14	10	3. others	0. primary_insomnia	17	10
Espie1989	Placebo	PE	1989	UK	42.4	15.3	14	10	3. others	0. primary_insomnia	17	2
Espie1989	Progressive relaxation	RT	1989	UK	48.1	10.6	14	7	3. others	0. primary_insomnia	17	1
Espie1989	No treatment	WL	1989	UK	45.3	20.9	13	8	3. others	0. primary_insomnia	16	1
Espie2001	CBT	CBT	2001	UK	51.4	17.0	74	48	1. formal_ICSD	0. primary_insomnia	74	21
Espie2001	SMC	WL	2001	UK	51.4	17.0	65	47	1. formal_ICSD	0. primary_insomnia	65	4

© 2024 Furukawa Y et al. *JAMA Psychiatry.*

trial	Arm	treatment	Year	Country	Age_m	Age_sd	Age_n	F_n	Insomnia diagnosis	Comorbidities	n	r
Espie2007	CBT	CBT	2007	UK	54.4	15.4	107	72	1. formal_DSM	1. primary_secondary_mixed	107	16
Espie2007	TAU	PE	2007	UK	54.1	14.4	94	65	1. formal_DSM	1. primary_secondary_mixed	94	5
Espie2008	CBT	CBT	2008	UK	60.5	12.4	100	69	1. formal_DSM	3. phy_cancer	100	40
Espie2008	TAU	PE	2008	UK	58.0	11.9	50	34	1. formal_DSM	3. phy_cancer	50	13
Espie2012	CBT	CBT	2012	UK	50.7	13.8	55	40	1. formal_DSM	0. primary_insomnia	55	31
Espie2012	IRT	WL	2012	UK	47.3	13.0	55	42	1. formal_DSM	0. primary_insomnia	55	11
Espie2012	TAU	WL	2012	UK	49.1	13.7	54	38	1. formal_DSM	0. primary_insomnia	54	7
Espie2019	dCBT	CBT	2019	UK	48.4	13.9	853	654	1. formal_DSM	4. psy_phy_mixed	858	362
Espie2019	SHE	PE	2019	UK	47.7	13.6	858	675	1. formal_DSM	4. psy_phy_mixed	853	96
Falloon2015	SSR	BT	2015	NZ	55.4	12.7	46	39	1. formal_Edinger2004	0. primary_insomnia	46	19
Falloon2015	Control	PE	2015	NZ	51.8	13.4	51	36	1. formal_Edinger2004	0. primary_insomnia	51	8
Farsani2021	CBT-I	CBT	2021	Iran	51.4	3.0	22	22	1. formal_DSM	3. phy_menopause	23	12
Farsani2021	Control	WL	2021	Iran	52.4	3.5	23	23	1. formal_DSM	3. phy_menopause	23	0
Felder2020	digital CBTI	CBT	2020	USA	33.9	3.4	105	105	1. formal_DSM	3. phy_pregnancy_perinatal	105	38
Felder2020	Standard treatment	WL	2020	USA	33.2	4.0	103	103	1. formal_DSM	3. phy_pregnancy_perinatal	103	26
Fernando2013	intervention	BT	2013	New Zealand	58.0	*	22	15	1. formal_DSM	0. primary_insomnia	22	16
Fernando2013	control	PE	2013	New Zealand	53.0	*	23	13	1. formal_DSM	0. primary_insomnia	23	8
Feuerstein2017	cb-CBT-I	CBT	2017	USA	48.0	10.0	16	9	2. informal_ISI	2. psy_mixed	18	1
Feuerstein2017	Sleep Diary	PE	2017	USA	50.0	10.0	18	10	2. informal_ISI	2. psy_mixed	16	0

© 2024 Furukawa Y et al. *JAMA Psychiatry.*

trial	Arm	treatment	Year	Country	Age_m	Age_sd	Age_n	F_n	Insomnia diagnosis	Comorbidities	n	r
Ford2022	eCBT-I	CBT	2022	Netherlands	53.0	14.0	27	15	1. formal_DSM	3. phy_others	27	11
Ford2022	TAU	WL	2022	Netherlands	56.0	16.0	25	17	1. formal_DSM	3. phy_others	25	1
Fowler2009	CBT	CBT	2009	Canada	67.7	5.9	22	22	3. others	1. primary_secondary_mixed	24	9
Fowler2009	SC	PE	2009	Canada	68.2	5.1	23	23	3. others	1. primary_secondary_mixed	23	0
Freeman2015	CBT	CBT	2015	UK	39.6	11.6	24	8	2. informal_ISI	2. psy_schizophrenia_or_psychosis	24	8
Freeman2015	standard care	PE	2015	UK	42.2	13.5	26	8	2. informal_ISI	2. psy_schizophrenia_or_psychosis	26	2
Freeman2017	Treatment	CBT	2017	UK	24.8	7.7	1891	1361	2. informal_SCI	1. primary_secondary_mixed	1891	271
Freeman2017	Contrl	TAU	2017	UK	24.6	7.6	1864	1315	2. informal_SCI	1. primary_secondary_mixed	1864	172
Fucito2014	CBT-I+SC	CBT	2014	USA	51.9	15.1	7	4	1. formal_DSM	2. psy_substance_dependence	9	3
Fucito2014	SC	PE	2014	USA	51.2	10.7	10	5	1. formal_DSM	2. psy_substance_dependence	10	2
Garcia2018	Intervention	CBT	2018	Brazil	55.2	6.1	19	19	1. formal_DSM	3. phy_menopause	21	18

© 2024 Furukawa Y et al. *JAMA Psychiatry.*

trial	Arm	treatment	Year	Country	Age_m	Age_sd	Age_n	F_n	Insomnia diagnosis	Comorbidities	n	r
Garcia2018	Control	PLB	2018	Brazil	56.7	4.0	11	11	1. formal_DSM	3. phy_menopause	14	0
Garland2014	CBT-I	CBT	2014	Canada	58.7	10.5	40	32	1. formal_DSM	3. phy_cancer	47	20
Garland2014	MBSR	CBT	2014	Canada	60.3	12.2	32	20	1. formal_DSM	3. phy_cancer	64	11
Gebara2019	Immediate BBTI	BT	2019	USA	64.0	2.5	6	2	1. formal_DSM	2. psy_MDD	6	1
Gebara2019	Delayed BBTI	WL	2019	USA	66.2	5.4	5	1	1. formal_DSM	2. psy_MDD	5	1
Gehrman2020	In Person	CBT	2020	USA	54.6	12.5	46	5	2. informal_ISI	2. psy_PTSD	47	4
Gehrman2020	Telehealth	CBT	2020	USA	55.6	12.1	49	4	2. informal_ISI	2. psy_PTSD	49	2
Gehrman2021	In-person	CBT	2021	USA	33.7	10.6	20	13	1. formal_DSM	1. primary_secondary_mixed	20	16
Gehrman2021	Telehealth	CBT	2021	USA	33.1	10.0	21	13	1. formal_DSM	1. primary_secondary_mixed	21	15
Gehrman2021	Waitlist	WL	2021	USA	31.2	8.7	19	13	1. formal_DSM	1. primary_secondary_mixed	19	0
Germain2014	BBTI-MV	BT	2014	USA	40.9	12.0	20	5	1. formal_ICSD	1. primary_secondary_mixed	20	9
Germain2014	Information Control	WL	2014	USA	35.9	11.2	20	1	1. formal_ICSD	1. primary_secondary_mixed	20	5
Gieselmann2019	F2F	BT	2019	Germany	39.3	14.5	27	13	1. formal_DSM	0. primary_insomnia	28	7
Gieselmann2019	Chat	BT	2019	Germany	39.7	11.2	23	13	1. formal_DSM	0. primary_insomnia	23	12
Gieselmann2019	WL	WL	2019	Germany	42.7	11.7	22	12	1. formal_DSM	0. primary_insomnia	22	2
Glozier2019	SHUTi	CBT	2019	Australia	58.6	6.3	45	0	2. informal_ISI	2. psy_MDD	45	14
Glozier2019	Control	PE	2019	Australia	58.1	6.1	42	0	2. informal_ISI	2. psy_MDD	42	6

© 2024 Furukawa Y et al. *JAMA Psychiatry.*

trial	Arm	treatment	Year	Country	Age_m	Age_sd	Age_n	F_n	Insomnia diagnosis	Comorbidities	n	r
Greeff1998	Treatment	RT	1998	South Africa	45.5	9.5	11	0	3. others	2. psy_substance_dependence	11	3
Greeff1998	Control	WL	1998	South Africa	45.5	9.5	11	0	3. others	2. psy_substance_dependence	11	1
Guarnaccia2023	MBS-I	CT	2023	USA	50.8	10.1	24	20	2. informal_ISI	3. phy_others	27	6
Guarnaccia2023	SH	PE	2023	USA	50.9	10.8	26	21	2. informal_ISI	3. phy_others	26	1
Hagatun2019	SHUTi	CBT	2019	Norway	45.0	12.4	95	61	1. formal_DSM	0. primary_insomnia	95	40
Hagatun2019	Patient education	PE	2019	Norway	44.8	13.7	86	61	1. formal_DSM	0. primary_insomnia	86	5
Hall2022	CBTI	CBT	2022	USA	51.2	17.1	20	18	1. formal_DSM	1. primary_secondary_mixed	20	8
Hall2022	EUC	PE	2022	USA	55.3	10.3	20	16	1. formal_DSM	1. primary_secondary_mixed	20	2
Ham2020	Experimetal	CBT	2020	South Korea	53.8	6.6	24	28	2. informal_other_self_reported_scales	1. primary_secondary_mixed	28	9
Ham2020	Control	PE	2020	South Korea	55.5	4.4	20	30	2. informal_other_self_reported_scales	1. primary_secondary_mixed	30	1
Harris2012-1	ISR+SCT	BT	2012	Australia	36.9	11.5	20	17	3. others	0. primary_insomnia	20	7
Harris2012-1	ISR	PE	2012	Australia	38.5	13.3	19	11	3. others	0. primary_insomnia	19	2
Harris2012-2	SCT	BT	2012	USA	42.2	13.1	20	15	3. others	0. primary_insomnia	20	2
Harris2012-2	CONTROL	PE	2012	USA	43.8	13.8	20	13	3. others	0. primary_insomnia	20	0

© 2024 Furukawa Y et al. *JAMA Psychiatry.*

trial	Arm	treatment	Year	Country	Age_m	Age_sd	Age_n	F_n	Insomnia diagnosis	Comorbidities	n	r
Harris2019	BI	BT	2019	USA	55.7	12.2	12	8	2. informal_ISI	3. phy_heart_failure	12	5
Harris2019	SMC	PE	2019	USA	55.6	10.8	11	8	2. informal_ISI	3. phy_heart_failure	11	1
Harvey2014	BT	BT	2014	USA, Canada	48.5	13.6	63	40	1. formal_DSM	4. psy_phy_mixed	63	22
Harvey2014	CBT	CBT	2014	USA, Canada	46.9	11.3	60	32	1. formal_DSM	4. psy_phy_mixed	60	33
Harvey2014	CT	CT	2014	USA, Canada	46.7	12.8	65	45	1. formal_DSM	4. psy_phy_mixed	65	18
Harvey2015	CBTI-BP	CBT	2015	USA	37.7	12.4	30	19	1. formal_DSM	2. psy_bipolar	30	17
Harvey2015	PE	RT	2015	USA	35.5	9.3	28	17	1. formal_DSM	2. psy_bipolar	28	3
Ho2014	SHS	CBT	2014	Hong Kong	36.9	13.0	103	73	3. others	1. primary_secondary_mixed	103	16
Ho2014	SH	CBT	2014	Hong Kong	38.6	11.8	104	70	3. others	1. primary_secondary_mixed	104	20
Ho2014	WL	WL	2014	Hong Kong	39.9	12.7	105	79	3. others	1. primary_secondary_mixed	105	10
Ho2021	CBT	CBT	2021	Hong Kong	43.1	14.3	13	11	2. informal_ISI	1. primary_secondary_mixed	13	3
Ho2021	WL	WL	2021	Hong Kong	36.6	11.7	13	9	2. informal_ISI	1. primary_secondary_mixed	13	0
Holmqvist2014	Telehealth-based	CBT	2014	Canada	*	*	*	27	1. formal_Edinger2004	1. primary_secondary_mixed	34	18
Holmqvist2014	Web-based	CBT	2014	Canada	*	*	*	28	1. formal_Edinger2004	1. primary_secondary_mixed	39	24

© 2024 Furukawa Y et al. *JAMA Psychiatry.*

trial	Arm	treatment	Year	Country	Age_m	Age_sd	Age_n	F_n	Insomnia diagnosis	Comorbidities	n	r
Horsch2017	App	BT	2017	Netherland	39.0	13.0	79	45	1. formal_DSM	1. primary_secondary_mixed	74	17
Horsch2017	WI	WL	2017	Netherland	41.0	13.9	74	49	1. formal_DSM	1. primary_secondary_mixed	79	6
Hou2014	Treatment	BT	2014	China	54.5	13.8	51	31	2. informal_PSQI	3. phy_others	52	0
Hou2014	Control	NT	2014	China	52.4	14.5	47	25	2. informal_PSQI	3. phy_others	51	0
Jacobs1993	SC	BT	1993	USA	36.7	*	10	8	3. others	0. primary_insomnia	10	6
Jacobs1993	MF	BT	1993	USA	36.7	*	10	8	3. others	0. primary_insomnia	10	9
Jacobs2004	Combination	CBT	2004	USA	49.1	9.6	18	12	1. formal_DSM	0. primary_insomnia	18	6
Jacobs2004	Pharmacotherapy	PE	2004	USA	45.4	9.3	15	11	1. formal_DSM	0. primary_insomnia	15	2
JanssonFrojmark2012a	BT	BT	2012	Sweden	55.2	10.7	11	6	1. formal_Edinger2004	0. primary_insomnia	11	0
JanssonFrojmark2012a	BT+CW	CBT	2012	Sweden	57.8	14.7	10	5	1. formal_Edinger2004	0. primary_insomnia	11	0
JanssonFrojmark2012b	CBT-I	CBT	2012	Sweden	57.8	6.6	17	10	1. formal_DSM	3. phy_others	17	4
JanssonFrojmark2012b	WLC	WL	2012	Sweden	53.6	10.4	15	10	1. formal_DSM	3. phy_others	15	0
Jarnefelt2020	Group-based CBT-I	CBT	2020	Finland	45.0	10.4	26	18	1. formal_ICD	3. phy_others	30	5
Jarnefelt2020	Self-help-based CBT-I	CBT	2020	Finland	41.0	10.4	27	21	1. formal_ICD	3. phy_others	29	4
Jarnefelt2020	Sleep hygiene education	PE	2020	Finland	47.0	10.4	22	17	1. formal_ICD	3. phy_others	24	5

© 2024 Furukawa Y et al. *JAMA Psychiatry.*

trial	Arm	treatment	Year	Country	Age_m	Age_sd	Age_n	F_n	Insomnia diagnosis	Comorbidities	n	r
Javaheri2020	Intervention	CBT	2020	USA	70.3	10.0	18	11	2. informal_ISI	3. phy_heart_failure	18	6
Javaheri2020	Control	PE	2020	USA	72.9	9.2	16	14	2. informal_ISI	3. phy_heart_failure	16	3
Jernelov2012	Bibliotherapy with support	CBT	2012	Sweden	50.8	11.8	44	33	1. formal_Edinger2004	1. primary_secondary_mixed	44	27
Jernelov2012	Bibliotherapy	CBT	2012	Sweden	47.4	13.3	45	36	1. formal_Edinger2004	1. primary_secondary_mixed	45	11
Jernelov2012	Wait list	WL	2012	Sweden	45.4	16.0	44	40	1. formal_Edinger2004	1. primary_secondary_mixed	44	1
Johann2020	Treatment	CBT	2020	Germany	40.8	14.0	23	14	1. formal_DSM	0. primary_insomnia	23	14
Johann2020	Control	WL	2020	Germany	41.2	15.1	23	15	1. formal_DSM	0. primary_insomnia	23	3
Jungquist2010	Treatment	CBT	2010	USA	52.0	9.9	19	16	3. others	3. phy_others	19	15
Jungquist2010	Control	PE	2010	USA	43.0	10.7	9	8	3. others	3. phy_others	9	2
Kaku2012	Intervention	BT	2012	Japan	35.6	9.8	82	9	2. informal_AIS	0. primary_insomnia	111	24
Kaku2012	Control	WL	2012	Japan	37.0	10.2	69	12	2. informal_AIS	0. primary_insomnia	112	7
Kaldo2015	Control	CBT	2015	Sweden	49.0	15.6	75	57	1. formal_Edinger2004	1. primary_secondary_mixed	75	11
Kaldo2015	Treatment	CBT	2015	Sweden	47.0	15.2	73	59	1. formal_Edinger2004	1. primary_secondary_mixed	73	31
Kaldo2020	Seflhelp book-group	CBT	2020	Sweden	52.2	10.7	20	17	1. formal_Edinger2004	1. primary_secondary_mixed	20	8
Kaldo2020	CAU	WL	2020	Sweden	57.9	10.8	20	15	1. formal_Edinger2004	1. primary_secondary_mixed	20	2
Kallestad2021	FtF CBT-I	CBT	2021	Norway	41.3	12.5	52	41	1. formal_DSM	1. primary_secondary_mixed	52	26

© 2024 Furukawa Y et al. *JAMA Psychiatry.*

trial	Arm	treatment	Year	Country	Age_m	Age_sd	Age_n	F_n	Insomnia diagnosis	Comorbidities	n	r
Kallestad2021	dCBT-I	CBT	2021	Norway	41.4	10.5	49	35	1. formal_DSM	1. primary_secondary_mixed	49	8
Kalmbach2020	CBT-I	CBT	2020	USA	28.9	4.1	46	46	2. informal_ISI	3. phy_pregnancy_perinatal	46	16
Kalmbach2020	Control	PE	2020	USA	29.2	4.1	45	45	2. informal_ISI	3. phy_pregnancy_perinatal	45	8
Kapella2011	CBT-I	CBT	2011	USA	65.0	9.0	9	2	2. informal_ISI	3. phy_others	9	2
Kapella2011	WE	PE	2011	USA	60.0	10.0	9	2	2. informal_ISI	3. phy_others	9	1
Kapella2022	CBT-I+AC or COPD-ED	CBT	2022	USA	64.0	8.0	55	20	2. informal_ISI	3. phy_others	55	16
Kapella2022	AC+AC or COPD-ED	PE	2022	USA	65.0	9.0	54	20	2. informal_ISI	3. phy_others	54	6
Kennet2021	Intervention	CBT	2021	Australia	27.6	10.4	14	14	2. informal_ISI	0. primary_insomnia	18	9
Kennet2021	Waitlist	WL	2021	Australia	31.5	13.6	13	12	2. informal_ISI	0. primary_insomnia	14	1
Krieger2019	SRT	BT	2019	Switzerland	46.6	17.5	41	28	1. formal_ICSD	0. primary_insomnia	41	10
Krieger2019	MCT	CBT	2019	Switzerland	42.2	12.4	42	26	1. formal_ICSD	0. primary_insomnia	42	16
Krieger2019	CAU	WL	2019	Switzerland	45.2	12.4	21	17	1. formal_ICSD	0. primary_insomnia	21	1
Kuhn2022	Insomnia Coach	CBT	2022	USA	44.0	7.6	25	14	2. informal_ISI	1. primary_secondary_mixed	25	3
Kuhn2022	Wait-list	WL	2022	USA	45.0	8.1	25	7	2. informal_ISI	1. primary_secondary_mixed	25	1

© 2024 Furukawa Y et al. *JAMA Psychiatry.*

trial	Arm	treatment	Year	Country	Age_m	Age_sd	Age_n	F_n	Insomnia diagnosis	Comorbidities	n	r
Kyle2020	dCBT	CBT	2020	UK	52.5	11.2	205	175	1. formal_DSM	1. primary_secondary_mixed	205	42
Kyle2020	WLC	WL	2020	UK	52.4	11.7	205	180	1. formal_DSM	1. primary_secondary_mixed	205	4
Lami2018	CBT-IP	CBT	2018	Spain	49.7	8.4	38	42	1. formal_DSM	3. phy_fibromyalgia	42	1
Lami2018	CBT-P	PLB	2018	Spain	49.4	6.4	34	42	1. formal_DSM	3. phy_fibromyalgia	42	1
Lancee2012	Electronic+Pen	CBT	2012	Netherland	51.7	12.1	417	298	1. formal_DSM	1. primary_secondary_mixed	422	162
Lancee2012	Waitlist	WL	2012	Netherland	51.9	12.2	200	136	1. formal_DSM	1. primary_secondary_mixed	202	28
Lancee2013	No support	CBT	2013	Netherland	47.4	11.8	133	98	1. formal_DSM	1. primary_secondary_mixed	133	25
Lancee2013	Support	CBT	2013	Netherland	49.3	13.2	129	99	1. formal_DSM	1. primary_secondary_mixed	129	52
Lancee2015	CBT-I	CBT	2015	Netherland	47.5	14.4	36	30	1. formal_DSM	1. primary_secondary_mixed	36	12
Lancee2015	Wait-list	WL	2015	Netherland	50.0	13.7	27	20	1. formal_DSM	1. primary_secondary_mixed	27	3
Lancee2016	f2f	CBT	2016	Netherland	38.5	13.1	30	22	1. formal_DSM	1. primary_secondary_mixed	30	22
Lancee2016	Online	CBT	2016	Netherland	41.2	14.1	30	26	1. formal_DSM	1. primary_secondary_mixed	30	6
Lancee2016	WL	WL	2016	Netherland	45.1	13.7	30	25	1. formal_DSM	1. primary_secondary_mixed	30	2
Lappalainen2019	iACT	CBT	2019	Finland	56.1	11.1	43	32	2. informal_ISI	0. primary_insomnia	43	37

© 2024 Furukawa Y et al. *JAMA Psychiatry.*

trial	Arm	treatment	Year	Country	Age_m	Age_sd	Age_n	F_n	Insomnia diagnosis	Comorbidities	n	r
Lappalainen2019	WLC	WL	2019	Finland	50.8	15.3	40	21	2. informal_ISI	0. primary_insomnia	43	26
Latocha2022	CBTI	BT	2022	Denmark	60.0	10.0	31	28	1. formal_ICSD	3. phy_others	31	11
Latocha2022	UC	PE	2022	Denmark	57.0	11.0	31	27	1. formal_ICSD	3. phy_others	31	0
Leerssen2022-1	CBT-I	CBT	2022	Netherland d	47.4	13.3	31	23	1. formal_DSM	0. primary_insomnia	31	9
Leerssen2022-1	NT	PE	2022	Netherland d	47.9	12.2	35	25	1. formal_DSM	0. primary_insomnia	35	2
Leerssen2022-2	CBT-I+CRS	CBT	2022	Netherland d	47.9	12.8	32	24	1. formal_DSM	0. primary_insomnia	32	7
Leerssen2022-2	CRS	PE	2022	Netherland d	49.2	13.5	34	25	1. formal_DSM	0. primary_insomnia	34	5
Lichstein2000	Treatment	BT	2000	USA	67.1	6.1	23	11	1. formal_ICSD	4. psy_phy_mixed	23	17
Lichstein2000	Control	WL	2000	USA	70.1	6.8	21	10	1. formal_ICSD	4. psy_phy_mixed	21	11
Lichstein2001	Placebo	PE	2001	USA	68.0	5.9	23	15	1. formal_ICSD	0. primary_insomnia	29	15
Lichstein2001	Relaxation	RT	2001	USA	68.1	8.3	27	21	1. formal_ICSD	0. primary_insomnia	30	19
Liu2022-1	Control	NT	2022	China	51.41	10.1	34	32	2. informal_PSQI	3. phy_cancer	34	1
Liu2022-1	MBSR	CT	2022	China	48.58	8.48	38	36	2. informal_PSQI	3. phy_cancer	38	8
Liu2022-2	Acupressure	PLB	2022	China	53.06	8.29	36	36	2. informal_PSQI	3. phy_cancer	36	9
Liu2022-2	MBSR + Acupressure	CT	2022	China	52.67	8.19	39	39	2. informal_PSQI	3. phy_cancer	39	15
Lopez2019	eCBT-I	CBT	2019	France	46.0	14.9	23	19	1. formal_DSM	0. primary_insomnia	23	4
Lopez2019	mPT	PE	2019	France	45.0	17.6	23	15	1. formal_DSM	0. primary_insomnia	23	1
Lorenz2019	Treatment	CBT	2019	Switzerland,	41.7	17.3	29	21	1. formal_DSM	0. primary_insomnia	29	14

© 2024 Furukawa Y et al. *JAMA Psychiatry.*

trial	Arm	treatment	Year	Country	Age_m	Age_sd	Age_n	F_n	Insomnia diagnosis	Comorbidities	n	r
Lorenz2019	Control	WL	2019	Austria, Germany Switzerland, Austria, Germany	44.0	20.1	27	18	1. formal_DSM	0. primary_insomnia	27	3
Lovato2014	CBT-I	CBT	2014	Austraria	64.0	*	86	43	2. informal_PSQI	0. primary_insomnia	86	33
Lovato2014	Waitlist	WL	2014	Austraria	64.0	*	32	16	2. informal_PSQI	0. primary_insomnia	32	1
Low2020	Mindfulness	CT	2020	Australia	36.4	11.7	12	10	2. informal_ISI	0. primary_insomnia	12	6
Low2020	PMR	RT	2020	Australia	36.4	11.7	11	10	2. informal_ISI	0. primary_insomnia	11	5
Maguen2021	BBTI	BT	2021	USA	49.5	15.7	46	9	1. formal_DSM	1. primary_secondary_mixed	46	21
Maguen2021	PMRT	RT	2021	USA	50.0	15.3	45	8	1. formal_DSM	1. primary_secondary_mixed	45	8
Majd2020	CBTI	CBT	2020	Iran	36.2	5.9	156	84	1. formal_DSM	0. primary_insomnia	156	28
Majd2020	PE	PE	2020	Iran	35.3	5.8	156	90	1. formal_DSM	0. primary_insomnia	156	5
Manber2008	EsCIT+CBTI	CBT	2008	USA	49.5	13.6	15	8	1. formal_DSM	2. psy_MDD	15	5
Manber2008	EsCIT+CTRL	PE	2008	USA	47.8	13.4	15	10	1. formal_DSM	2. psy_MDD	15	1
Manber2016	CBTI	CBT	2016	USA	48.3	12.7	75	53	1. formal_DSM	2. psy_MDD	75	31
Manber2016	CTRL	PE	2016	USA	45.0	12.3	75	57	1. formal_DSM	2. psy_MDD	75	15
Manber2019	CBTI	CBT	2019	USA	33.4	5.2	89	96	1. formal_DSM	3. phy_pregnancy_perinatal	96	57
Manber2019	CTRL	PE	2019	USA	32.6	4.9	90	98	1. formal_DSM	3. phy_pregnancy_perinatal	98	47
Mao2018	Intervention	CBT	2018	China	43.3	12.0	52	38	1. formal_DSM	0. primary_insomnia	52	4

© 2024 Furukawa Y et al. *JAMA Psychiatry.*

trial	Arm	treatment	Year	Country	Age_m	Age_sd	Age_n	F_n	Insomnia diagnosis	Comorbidities	n	r
Mao2018	Control	PE	2018	China	42.5	9.9	52	36	1. formal_DSM	0. primary_insomnia	52	1
Marino2001	CBTI	CBT	2001	Canada	41.5	*	35	22	1. formal_ICSD	0. primary_insomnia	35	0
Marino2001	WL	WL	2001	Canada	41.5	*	35	24	1. formal_ICSD	0. primary_insomnia	35	0
Marks2023	CBTI	CBT	2023	UK	54.0	12.59	33	14	2. informal_ISI	3. phy_others	33	18
Marks2023	ABC	RT	2023	UK	52.5	14.44	34	18	2. informal_ISI	3. phy_others	34	5
Marks2023	SSG	PE	2023	UK	51.0	10.37	35	17	2. informal_ISI	3. phy_others	35	0
Martinez2014	CBT-I	CBT	2014	Spain	46.5	6.3	30	30	1. formal_DSM	3. phy_fibromyalgia	32	2
Martinez2014	SH	WL	2014	Spain	48.7	7.3	29	29	1. formal_DSM	3. phy_fibromyalgia	32	0
Matthews2014	CBTI	CBT	2014	USA	52.2	6.9	30	30	2. informal_other_self_reported_scales	3. phy_cancer	32	11
Matthews2014	BPT	PE	2014	USA	52.9	7.8	26	26	2. informal_other_self_reported_scales	3. phy_cancer	28	6
McCrae2007	MBT	BT	2007	USA	77.2	8.0	11	7	1. formal_DSM	0. primary_insomnia	12	9
McCrae2007	SHE	PE	2007	USA	77.2	8.0	9	7	1. formal_DSM	0. primary_insomnia	9	3
McCrae2019	CBT-I	CBT	2019	USA	54.1	11.0	39	39	3. others	3. phy_fibromyalgia	39	28
McCrae2019	WLC	WL	2019	USA	52.3	11.2	37	37	3. others	3. phy_fibromyalgia	37	12
McCrae2020	BBTi	BT	2020	USA	68.0	6.0	32	22	3. others	0. primary_insomnia	32	9
McCrae2020	SMC	PE	2020	USA	71.0	9.1	30	20	3. others	0. primary_insomnia	30	2
McCurry2016	CBT-I	CBT	2016	USA	55.0	3.5	53	53	2. informal_ISI	3. phy_menopause	53	33
McCurry2016	MEC	PE	2016	USA	54.7	4.7	53	53	2. informal_ISI	3. phy_menopause	53	10
McCurry2021	CBT-I	CBT	2021	USA	70.1	7.1	163	124	2. informal_ISI	3. phy_others	163	79
McCurry2021	EOC	PE	2021	USA	70.4	6.5	164	120	2. informal_ISI	3. phy_others	164	37
Means2000	Treated SWI	RT	2000	USA	21.0	5.3	28	19	1. formal_ICSD	0. primary_insomnia	28	17

© 2024 Furukawa Y et al. *JAMA Psychiatry.*

trial	Arm	treatment	Year	Country	Age_m	Age_sd	Age_n	F_n	Insomnia diagnosis	Comorbidities	n	r
Means2000	Untreated SWI	WL	2000	USA	20.2	4.4	29	20	1. formal_ICSD	0. primary_insomnia	29	12
Milby1993	Triazolam plus behavioral therapy	BT	1993	USA	32.5	5.9	8	4	1. formal_DSM	0. primary_insomnia	8	2
Milby1993	Triazolam plus sleep-related information	PE	1993	USA	32.5	5.9	7	4	1. formal_DSM	0. primary_insomnia	7	1
Miller2021	CBT-I	CBT	2021	USA	22.3	2.7	28	22	1. formal_DSM	2. psy_substance_dependence	28	10
Miller2021	SE	PE	2021	USA	22.5	2.8	28	20	1. formal_DSM	2. psy_substance_dependence	28	4
Miller2023	CBT-I	CBT	2023	USA	45.6	11.9	32	3	1. formal_DSM	2. psy_substance_dependence	32	21
Miller2023	Sleep hygiene	PE	2023	USA	46.9	11.9	35	3	1. formal_DSM	2. psy_substance_dependence	35	10
Mimeault1999	BT	CBT	1999	Canada	49.8	13.3	18	10	1. formal_DSM	0. primary_insomnia	22	4
Mimeault1999	BTPC	CBT	1999	Canada	45.6	8.5	18	11	1. formal_DSM	0. primary_insomnia	18	5
Mimeault1999	WLC	WL	1999	Canada	56.9	13.4	18	11	1. formal_DSM	0. primary_insomnia	18	1
Miro2011	CBT	CBT	2011	Spain	43.9	6.1	16	22	1. formal_DSM	3. phy_fibromyalgia	22	2
Miro2011	SH	WL	2011	Spain	50.2	6.1	15	22	1. formal_DSM	3. phy_fibromyalgia	22	0

© 2024 Furukawa Y et al. *JAMA Psychiatry.*

trial	Arm	treatment	Year	Country	Age_m	Age_sd	Age_n	F_n	Insomnia diagnosis	Comorbidities	n	r
Morawetz1989(SOL)	Therapist	BT	1989	Australia	44.0	*	16	*	3. others	0. primary_insomnia	16	7
Morawetz1989(SOL)	Tape	BT	1989	Australia	44.0	*	16	*	3. others	0. primary_insomnia	16	8
Morawetz1989(SOL)	WL	WL	1989	Australia	44.0	*	18	*	3. others	0. primary_insomnia	18	4
Morawetz1989(WASO)	Tape	BT	1989	Australia	44.0	*	7	*	3. others	0. primary_insomnia	7	4
Morawetz1989(WASO)	WL	WL	1989	Australia	44.0	*	12	*	3. others	0. primary_insomnia	12	4
Morgan2012	Treatment	CBT	2012	UK	67.0	7.9	98	68	1. formal_DSM	3. phy_mixed	99	9
Morgan2012	TAU	PE	2012	UK	66.3	6.9	95	60	1. formal_DSM	3. phy_mixed	103	7
Morin1988	SC	BT	1988	USA	68.4	5.7	9	6	3. others	0. primary_insomnia	9	3
Morin1988	IR	PE	1988	USA	67.5	4.9	8	6	3. others	0. primary_insomnia	8	1
Morin1988	WL	WL	1988	USA	66.4	6.4	10	5	3. others	0. primary_insomnia	10	1
Morin1993	CBT	CBT	1993	USA	67.1	5.3	12	9	1. formal_ICSD	0. primary_insomnia	12	9
Morin1993	WLC	WL	1993	USA	67.1	5.3	12	9	1. formal_ICSD	0. primary_insomnia	12	4
Morin1999	Combined	CBT	1999	USA	65.2	6.9	19	13	1. formal_DSM	0. primary_insomnia	20	1
Morin1999	PCT	PE	1999	USA	64.1	6.4	17	9	1. formal_DSM	0. primary_insomnia	20	0
NCT00127790-1	CBT-I	CBT	2012	USA	45.7	8.2	6	4	3. others	3. phy_others	6	4
NCT00127790-1	WL	WL	2012	USA	52.3	1.0	4	2	3. others	3. phy_others	4	0
NCT00127790-2	CBT-P	CBT	2012	USA	55.8	9.8	5	2	3. others	3. phy_others	5	0
NCT00127790-2	CBT-P/I	CBT	2012	USA	50.5	8.5	6	6	3. others	3. phy_others	6	5
NCT01011218-1	BBTI-Armodafil	BT	2018	USA	49.1	10.5	9	9	2. informal_ISI	3. phy_cancer	9	2

© 2024 Furukawa Y et al. *JAMA Psychiatry.*

trial	Arm	treatment	Year	Country	Age_m	Age_sd	Age_n	F_n	Insomnia diagnosis	Comorbidities	n	r
NCT01011218-1	BP-Armodafil	PE	2018	USA	46.7	9.2	9	9	2. informal_ISI	3. phy_cancer	9	3
NCT01011218-2	BBTI-without armodafil	BT	2018	USA	51.5	9.0	23	26	2. informal_ISI	3. phy_cancer	26	12
NCT01011218-2	BP-withou armodafil	PE	2018	USA	50.3	8.6	24	26	2. informal_ISI	3. phy_cancer	26	7
NCT01987089	CBTI	CBT	2020	USA	51.6	10.9	31	3	2. informal_ISI	2. psy_substance_dependence	31	12
NCT01987089	QDT	PE	2020	USA	51.5	8.2	32	2	2. informal_ISI	2. psy_substance_dependence	32	9
NCT02613364	CBTI	CBT	2021	USA	56.0	*	238	216	1. formal_DSM	3. phy_cancer	238	70
NCT02613364	Educational intervention	TAU	2021	USA	57.0	*	251	236	1. formal_DSM	3. phy_cancer	251	23
NCT03964974	CBTI-CU	CBT	2022	USA	36.7	12.0	30	23	2. informal_ISI	2. psy_substance_dependence	30	6
NCT03964974	SHE	PE	2022	USA	38.7	13.7	27	20	2. informal_ISI	2. psy_substance_dependence	27	3
NorellClarke2015	CBT-I	CBT	2015	Sweden	49.3	12.5	32	22	1. formal_DSM	2. psy_MDD	32	13
NorellClarke2015	RT	RT	2015	Sweden	53.7	12.4	32	27	1. formal_DSM	2. psy_MDD	32	3
Okajima2020	tailored+standard BBTI	BT	2020	Japan	43.0	11.5	47	17	2. informal_ISI	0. primary_insomnia	47	10

© 2024 Furukawa Y et al. *JAMA Psychiatry.*

trial	Arm	treatment	Year	Country	Age_m	Age_sd	Age_n	F_n	Insomnia diagnosis	Comorbidities	n	r
Okajima2020	self-monitoring	WL	2020	Japan	43.9	11.3	23	7	2. informal_ISI	0. primary_insomnia	23	4
Okajima2020	waitig list	WL	2020	Japan	40.7	10.9	22	8	2. informal_ISI	0. primary_insomnia	22	5
Okajima2021	REFRESH	CBT	2022	Japan	19.6	1.9	24	16	2. informal_ISI	1. primary_secondary_mixed	24	11
Okajima2021	Self-Monitoring	PE	2022	Japan	19.6	1.9	24	16	2. informal_ISI	1. primary_secondary_mixed	24	0
Ong2018	MBTI	CBT	2014	USA	42.4	*	15	12	1. formal_Edinger2004	0. primary_insomnia	19	5
Ong2018	MBSR	CT	2014	USA	41.9	*	13	9	1. formal_Edinger2004	0. primary_insomnia	19	6
Ong2018	SMC	WL	2014	USA	45.4	*	16	11	1. formal_Edinger2004	0. primary_insomnia	16	1
Ong2020	Arm B	CBT	2020	USA	53.2	11.1	39	25	1. formal_Edinger2004	3. phy_others	39	26
Ong2020	Arm C	PE	2020	USA	49.2	15.1	38	18	1. formal_Edinger2004	3. phy_others	39	15
Oswald2022	Intervention	CBT	2022	USA	56.9	8.9	15	15	2. informal_ISI	3. phy_cancer	15	6
Oswald2022	Control	WL	2022	USA	60.0	9.6	15	15	2. informal_ISI	3. phy_cancer	15	3
Padron2022	CBTi.p.	CBT	2022	USA	58.9	12.2	18	18	3. others	3. phy_cancer	18	12
Padron2022	PE	PE	2022	USA	59.9	10.3	17	17	3. others	3. phy_cancer	17	7
Palesh2018	BBT-CI	BT	2018	USA	50.9	7.9	34	34	2. informal_ISI	3. phy_cancer	34	9
Palesh2018	HEAL	TAU	2018	USA	53.8	11.2	37	37	2. informal_ISI	3. phy_cancer	37	4
Palesh2020	BBT-I	BT	2020	USA	50.9	9.3	35	37	2. informal_other_self_reported_scales	3. phy_cancer	36	16
Palesh2020	Sleep education	PE	2020	USA	49.4	8.8	35	37	2. informal_other_self_reported_scales	3. phy_cancer	37	10
Patel2017	Case	CBT	2017	USA	63.1	6.8	14	3	2. informal_ISI	3. phy_others	14	4

© 2024 Furukawa Y et al. *JAMA Psychiatry.*

trial	Arm	treatment	Year	Country	Age_m	Age_sd	Age_n	F_n	Insomnia diagnosis	Comorbidities	n	r
Patel2017	Control	PE	2017	USA	64.7	9.5	14	9	2. informal_ISI	3. phy_others	14	2
Perlis2004	M+CBT	CBT	2004	USA	35.0	11.7	10	8	1. formal_ICSD	0. primary_insomnia	10	9
Perlis2004	M+CC	PE	2004	USA	42.4	13.8	8	5	1. formal_ICSD	0. primary_insomnia	10	6
Perrault2022	TX	CBT	2022	Canada	48.8	17.0	33	25	1. formal_ICSD	1. primary_secondary_mixed	33	12
Perrault2022	WL	WL	2022	Canada	56.0	15.6	29	22	1. formal_ICSD	1. primary_secondary_mixed	29	1
Pigeon2017	CBT-I	CBT	2017	USA	56.9	10.0	13	1	2. informal_ISI	2. psy_MDD	18	2
Pigeon2017	SH	PE	2017	USA	59.9	9.3	14	2	2. informal_ISI	2. psy_MDD	18	2
Pigeon2019	CBTi	CBT	2019	USA	52.8	14.5	24	5	2. informal_ISI	2. psy_mixed	27	11
Pigeon2019	TAU	TAU	2019	USA	56.8	11.0	26	5	2. informal_ISI	2. psy_mixed	27	0
Pigeon2022	CBTI+CPT	CBT	2021	USA	34.2	10.8	56	54	2. informal_ISI	2. psy_PTSD	56	8
Pigeon2022	Control+CPT	PE	2021	USA	36.8	10.9	54	53	2. informal_ISI	2. psy_PTSD	54	1
Pillai2015	CBTI	CBT	2015	USA	53.2	12.2	13	12	1. formal_DSM	2. psy_anxiety	19	11
Pillai2015	IC	PE	2015	USA	44.0	13.2	9	8	1. formal_DSM	2. psy_anxiety	13	4
Prados2020	CBTI-C	CBT	2020	Spain	49.0	9.5	17	20	1. formal_DSM	3. phy_fibromyalgia	20	1
Prados2020	CBTI-P	CBT	2020	Spain	51.2	5.3	15	19	1. formal_DSM	3. phy_fibromyalgia	19	0
Quintiliani2017	PI+	CT	2017	Italy	64.4	12.9	19	10	1. formal_ICSD	0. primary_insomnia	20	5
Quintiliani2017	PI-	PE	2017	Italy	59.6	14.4	19	14	1. formal_ICSD	0. primary_insomnia	20	3
Redeker2015	CBTI	CBT	2015	USA	61.9	13.3	29	15	2. informal_ISI	3. phy_heart_failure	31	15
Redeker2015	Attention Control	WL	2015	USA	55.2	16.2	19	10	2. informal_ISI	3. phy_heart_failure	21	6
Redeker2022	CBT-I	CBT	2022	USA	62.0	13.1	91	38	2. informal_ISI	3. phy_heart_failure	100	28
Redeker2022	Attention Control	PE	2022	USA	64.1	12.6	84	37	2. informal_ISI	3. phy_heart_failure	89	17

© 2024 Furukawa Y et al. *JAMA Psychiatry.*

trial	Arm	treatment	Year	Country	Age_m	Age_sd	Age_n	F_n	Insomnia diagnosis	Comorbidities	n	r
Ritterband2009	SHUTi	CBT	2009	USA	44.7	10.6	22	18	1. formal_DSM	0. primary_insomnia	22	16
Ritterband2009	Wait-list	WL	2009	USA	45.1	11.7	22	16	1. formal_DSM	0. primary_insomnia	22	0
Ritterband2012	Internet	CBT	2012	USA	53.7	10.8	14	14	1. formal_DSM	3. phy_cancer	14	6
Ritterband2012	Control	WL	2012	USA	59.6	12.3	14	10	1. formal_DSM	3. phy_cancer	14	1
Ritterband2017	SHUTi	CBT	2017	USA	43.8	11.3	151	103	3. others	1. primary_secondary_mixed	151	54
Ritterband2017	Patient education	PE	2017	USA	42.8	11.9	152	115	3. others	1. primary_secondary_mixed	152	16
Robabeh2015	CBT	CBT	2015	Iran	43.5	8.3	11	0	1. formal_DSM	2. psy_substance_dependence	11	7
Robabeh2015	BPT	PE	2015	Iran	44.7	7.8	11	0	1. formal_DSM	2. psy_substance_dependence	11	3
Roscoe2015-1	CBTI+placebo	CBT	2015	USA	59.0	9.9	24	21	3. others	3. phy_cancer	24	12
Roscoe2015-1	placebo	PE	2015	USA	52.0	11.5	25	18	3. others	3. phy_cancer	25	5
Roscoe2015-2	CBTI+armodafil	CBT	2015	USA	56.0	10.2	23	22	3. others	3. phy_cancer	23	14
Roscoe2015-2	armodafil	PE	2015	USA	57.0	7.4	24	23	3. others	3. phy_cancer	24	3
Rosen2000	Education control	PE	2000	USA	46.8	8.3	11	8	1. formal_ICSD	0. primary_insomnia	14	3
Rosen2000	M+G	RT	2000	USA	48.6	12.0	21	13	1. formal_ICSD	0. primary_insomnia	27	11
Rybarczyk2005	CBT	CBT	2005	USA	70.1	9.1	46	28	3. others	3. phy_mixed	46	3
Rybarczyk2005	SMW	CT	2005	USA	67.7	7.9	46	34	3. others	3. phy_mixed	46	1
Sadler2018	CBT-I	CBT	2018	Australia	74.7	7.1	24	15	1. formal_DSM	2. psy_MDD	24	7

© 2024 Furukawa Y et al. *JAMA Psychiatry.*

trial	Arm	treatment	Year	Country	Age_m	Age_sd	Age_n	F_n	Insomnia diagnosis	Comorbidities	n	r
Sadler2018	PCG	PE	2018	Australia	72.3	7.6	23	12	1. formal_DSM	2. psy_MDD	23	1
Sandlund2017	Intervention	CBT	2017	Sweden	55.0	17.1	90	64	3. others	1. primary_secondary_mixed	90	51
Sandlund2017	Control	WL	2017	Sweden	54.0	14.7	75	56	3. others	1. primary_secondary_mixed	75	10
Sato2019	ICBT+UC	CBT	2019	Japan	49.4	13.8	11	9	1. formal_DSM	1. primary_secondary_mixed	11	4
Sato2019	UC	WL	2019	Japan	50.5	8.8	12	9	1. formal_DSM	1. primary_secondary_mixed	12	0
Sato2022	ICBT	CBT	2022	Japan	49.8	11.1	79	32	2. informal_PSQI	1. primary_secondary_mixed	106	18
Sato2022	WLC	WL	2022	Japan	51.0	10.4	103	41	2. informal_PSQI	1. primary_secondary_mixed	103	12
Savard2005	CBT	CBT	2005	Canada	54.8	7.0	27	27	1. formal_DSM	3. phy_cancer	28	16
Savard2005	WL	WL	2005	Canada	53.4	7.7	30	30	1. formal_DSM	3. phy_cancer	30	3
Savard2014	PCBT-I	CBT	2014	Canada	52.6	8.9	81	81	2. informal_ISI	3. phy_cancer	81	58
Savard2014	VCBT-I	CBT	2014	Canada	55.3	8.7	80	80	2. informal_ISI	3. phy_cancer	80	35
Savard2014	CTL	PE	2014	Canada	55.4	8.8	81	81	2. informal_ISI	3. phy_cancer	81	21
Schuffelen2023	dCBT-I	CBT	2023	Germany	44.27	14.25	118	82	1. formal_DSM	1. primary_secondary_mixed	118	48
Schuffelen2023	Control	WL	2023	Germany	43.20	13.57	120	79	1. formal_DSM	1. primary_secondary_mixed	120	2
Sgoifo2017	Exposed	CBT	2017	Italy	*	*	*	*	1. formal_ICSD	3. phy_others	24	3
Sgoifo2017	Control	WL	2017	Italy	*	*	*	*	1. formal_ICSD	3. phy_others	24	1
Sheaves2018	STAC	CBT	2018	UK	40.0	12.0	20	0	2. informal_ISI	2. psy_mixed	20	8

© 2024 Furukawa Y et al. *JAMA Psychiatry.*

trial	Arm	treatment	Year	Country	Age_m	Age_sd	Age_n	F_n	Insomnia diagnosis	Comorbidities	n	r
Sheaves2018	standard	TAU	2018	UK	40.0	14.0	20	0	2. informal_ISI	2. psy_mixed	20	4
Shimamoto2022	SPA	BT	2022	Japan	37.7	11.7	37	15	2. informal_ISI	1. primary_secondary_mixed	62	30
Shimamoto2022	Control	WL	2022	Japan	45.0	10.7	34	11	2. informal_ISI	1. primary_secondary_mixed	56	18
Shin2023	CBT-I	CBT	2023	South Korea	59.33	7.43	12	10	1. formal_ICSD	0. primary_insomnia	15	5
Shin2023	ACT	CT	2023	South Korea	60.00	8.81	14	8	1. formal_ICSD	0. primary_insomnia	15	4
Short2021	BBTI	BT	2021	USA	21.0	4.9	28	16	2. informal_ISI	2. psy_mixed	28	9
Short2021	Control	WL	2021	USA	20.4	2.8	28	11	2. informal_ISI	2. psy_mixed	28	4
Siebmnns2021	Intervention	CBT	2021	Sweden	72.5	9.5	24	7	1. formal_ICD	3. phy_others	24	5
Siebmnns2021	Control	WL	2021	Sweden	72.6	10.4	24	10	1. formal_ICD	3. phy_others	24	1
Siengsukon2020	CBTI	CBT	2020	USA	51.1	7.9	10	9	2. informal_ISI	3. phy_others	12	9
Siengsukon2020	BE	PE	2020	USA	50.4	12.4	10	8	2. informal_ISI	3. phy_others	11	2
Siengsukon2020	AC	TAU	2020	USA	56.9	10.1	10	10	2. informal_ISI	3. phy_others	10	5
Siengsukon2021	wCBT-I	CBT	2021	USA	50.1	11.8	10	10	2. informal_ISI	3. phy_others	21	6
Siengsukon2021	wCBT-I+calls	CBT	2021	USA	53.8	6.9	10	9	2. informal_ISI	3. phy_others	20	3
Simeit2004	PMR	CBT	2004	Germany	60.2	9.2	80	56	1. formal_DSM	3. phy_cancer	80	40
Simeit2004	C-group	RT	2004	Germany	57.6	10.9	78	59	1. formal_DSM	3. phy_cancer	78	34
Smith2015	CBT-I	CBT	2015	USA	59.2	9.9	50	38	1. formal_Edinger2004	3. phy_others	50	18
Smith2015	BD	PE	2015	USA	59.6	9.1	50	41	1. formal_Edinger2004	3. phy_others	50	13
Smitherman2016	CBTi	BT	2016	USA	29.6	13.4	16	15	1. formal_ICSD	3. phy_others	16	11
Smitherman2016	Control	PE	2016	USA	32.1	12.8	15	13	1. formal_ICSD	3. phy_others	16	9

© 2024 Furukawa Y et al. *JAMA Psychiatry.*

trial	Arm	treatment	Year	Country	Age_m	Age_sd	Age_n	F_n	Insomnia diagnosis	Comorbidities	n	r
Soeffing2008	CBTI	BT	2008	USA	63.5	8.7	20	12	1. formal_ICSD	1. primary_secondary_mixed	20	11
Soeffing2008	Plcebo	PE	2008	USA	64.8	6.5	27	18	1. formal_ICSD	1. primary_secondary_mixed	27	10
Song2020	CBTI	CBT	2020	South Korea	54.8	10.3	12	8	1. formal_ICSD	3. phy_others	14	2
Song2020	NonCBTI	PE	2020	South Korea	57.0	5.4	13	10	1. formal_ICSD	3. phy_others	26	0
Speed2022	gCBT-I	CBT	2022	USA	35.1	10.0	10	0	2. informal_other_self_reported_scales	2. psy_substance_dependence	10	4
Speed2022	SOC	PE	2022	USA	45.6	14.1	11	0	2. informal_other_self_reported_scales	2. psy_substance_dependence	11	1
Store2022	Intervention	RT	2022	Sweden	50.05	13.23	22	19	1. formal_DSM	0. primary_insomnia	22	1
Store2022	Waitlist	WL	2022	Sweden	47.77	12.60	22	16	1. formal_DSM	0. primary_insomnia	22	0
Strom2004	Treatment	CBT	2004	Sweden	46.2	11.6	30	20	1. formal_DSM	0. primary_insomnia	54	14
Strom2004	Waitlist	WL	2004	Sweden	43.9	11.4	51	32	1. formal_DSM	0. primary_insomnia	55	23
Sunnheld2020	BT	BT	2020	Sweden	51.8	14.5	73	51	1. formal_DSM	1. primary_secondary_mixed	73	29
Sunnheld2020	CT	CT	2020	Sweden	51.5	12.5	72	55	1. formal_DSM	1. primary_secondary_mixed	72	25
Sunnheld2020	WL	WL	2020	Sweden	54.2	14.6	74	54	1. formal_DSM	1. primary_secondary_mixed	74	2

© 2024 Furukawa Y et al. *JAMA Psychiatry.*

trial	Arm	treatment	Year	Country	Age_m	Age_sd	Age_n	F_n	Insomnia diagnosis	Comorbidities	n	r
Sveen2021	Intervention	CBT	2021	Sweden	49.9	5.8	10	7	1. formal_DSM	1. primary_secondary_mixed	10	2
Sveen2021	Control	CT	2021	Sweden	45.6	5.5	11	7	1. formal_DSM	1. primary_secondary_mixed	11	1
Sweetman2019	CBTi	CBT	2019	Australia	59.1	9.9	72	40	1. formal_ICSD	3. phy_others	72	12
Sweetman2019	Control	WL	2019	Australia	57.3	9.9	73	40	1. formal_ICSD	3. phy_others	73	2
Talbot2014	CBT-I	CBT	2014	USA	37.1	10.4	29	22	1. formal_Edinger2004	2. psy_PTSD	29	12
Talbot2014	Waitlist	WL	2014	USA	37.3	11.0	16	9	1. formal_Edinger2004	2. psy_PTSD	16	0
Taylor2014a	CBTI	CBT	2014	USA	19.5	1.7	17	4	1. formal_DSM	0. primary_insomnia	17	11
Taylor2014a	WLC	WL	2014	USA	19.9	2.5	17	10	1. formal_DSM	0. primary_insomnia	17	2
Taylor2017	Internet CBTi	CBT	2017	USA	34.5	8.3	34	6	1. formal_DSM	1. primary_secondary_mixed	34	7
Taylor2017	In-person CBTi	CBT	2017	USA	30.8	6.4	33	7	1. formal_DSM	1. primary_secondary_mixed	33	12
Taylor2017	Control	PE	2017	USA	32.8	8.1	33	4	1. formal_DSM	1. primary_secondary_mixed	33	3
Taylor2018	CBTI	CBT	2018	USA	*	*	*	*	1. formal_DSM	1. primary_secondary_mixed	42	18
Taylor2018	Control	PE	2018	USA	*	*	*	*	1. formal_DSM	1. primary_secondary_mixed	43	2
Ustinov2014	Treatment	BT	2013	USA	53.6	10.8	36	*	1. formal_DSM	2. psy_mixed	39	7
Ustinov2014	Waitlist	WL	2013	USA	53.6	10.8	29	*	1. formal_DSM	2. psy_mixed	31	2
VanDerZweerde2019	i-Sleep	CBT	2019	Netherlands	44.6	13.1	52	43	1. formal_DSM	2. psy_MDD	52	28

© 2024 Furukawa Y et al. *JAMA Psychiatry.*

trial	Arm	treatment	Year	Country	Age_m	Age_sd	Age_n	F_n	Insomnia diagnosis	Comorbidities	n	r
VanDerZweerde2019	Sdiary	WL	2019	Netherlands	46.3	15.1	52	42	1. formal_DSM	2. psy_MDD	52	4
VanDerZweerde2020	i-Sleep	CBT	2020	Netherlands	51.7	15.8	69	43	1. formal_DSM	1. primary_secondary_mixed	69	28
VanDerZweerde2020	CAU	WL	2020	Netherlands	49.4	16.0	65	44	1. formal_DSM	1. primary_secondary_mixed	65	2
VanStraten2014	Intervention	CBT	2014	Netherlands	48.7	13.8	59	35	1. formal_DSM	1. primary_secondary_mixed	59	29
VanStraten2014	Control	WL	2014	Netherlands	50.1	11.9	59	48	1. formal_DSM	1. primary_secondary_mixed	59	6
Vedaa2020	dCBT-I	CBT	2020	Norway	44.2	13.9	868	596	2. informal_ISI	1. primary_secondary_mixed	868	219
Vedaa2020	patient education	PE	2020	Norway	44.8	13.7	853	571	2. informal_ISI	1. primary_secondary_mixed	853	41
Verma2022	CBTI	CBT	2022	Australia	32.9	5.0	39	39	2. informal_ISI	3. phy_others	39	23
Verma2022	TAU	WL	2022	Australia	31.4	3.8	39	39	2. informal_ISI	3. phy_others	39	6
Vincent2009	Treatment	CBT	2009	Canada	*	*	*	40	1. formal_Edinger2004	1. primary_secondary_mixed	59	11
Vincent2009	Control	WL	2009	Canada	*	*	*	39	1. formal_Edinger2004	1. primary_secondary_mixed	59	2
Vitiello2013	CBT-PI	BT	2013	USA	73.2	8.1	122	97	1. formal_Edinger2004	3. phy_others	122	52
Vitiello2013	CBT-P	RT	2013	USA	73.0	8.4	122	98	1. formal_Edinger2004	3. phy_others	122	45
Wagley2013	Treatment	CBT	2013	USA	43.6	*	20	14	2. informal_PSQI	2. psy_mixed	21	2
Wagley2013	Waitlist	WL	2013	USA	48.2	*	10	7	2. informal_PSQI	2. psy_mixed	10	0

© 2024 Furukawa Y et al. *JAMA Psychiatry.*

trial	Arm	treatment	Year	Country	Age_m	Age_sd	Age_n	F_n	Insomnia diagnosis	Comorbidities	n	r
Wang2016	BBTI	BT	2016	China	42.7	8.8	40	22	3. others	1. primary_secondary_mixed	40	16
Wang2016	Sleep hygiene	PE	2016	China	39.6	7.8	39	21	3. others	1. primary_secondary_mixed	39	2
Watanabe2011	BBTI+TAU	BT	2011	Japan	52.9	11.6	20	15	2. informal_ISI	2. psy_MDD	20	8
Watanabe2011	TAU	PE	2011	Japan	47.8	10.1	17	8	2. informal_ISI	2. psy_MDD	17	1
Watanabe2023	CBT-I App	CBT	2023	Japan	42.4	13.5	87	55	1. formal_ICSD	0. primary_insomnia	87	33
Watanabe2023	Sham App	PLB	2023	Japan	45.9	13.2	88	47	1. formal_ICSD	0. primary_insomnia	88	9
Wilkund2022	ICBT-i	BT	2022	Sweden	48.2	11.1	30	23	1. formal_DSM	3. phy_others	30	5
Wilkund2022	AR	RT	2022	Sweden	50.6	13.6	24	22	1. formal_DSM	3. phy_others	24	2
Woldeamanuel2021	CCT-CI	BT	2020	USA	51.1	11.5	73	73	2. informal_ISI	3. phy_cancer	73	19
Woldeamanuel2021	HEAL	TAU	2020	USA	49.7	10.0	66	66	2. informal_ISI	3. phy_cancer	66	16
Wong2016	CT	CBT	2016	Australia	49.5	12.5	31	*	3. others	0. primary_insomnia	31	21
Wong2016	MBT	CBT	2016	Australia	49.5	12.5	26	*	3. others	0. primary_insomnia	26	15
Wong2017	PEEC	BT	2017	China	56.6	9.7	105	78	1. formal_DSM	0. primary_insomnia	105	5
Wong2017	MBCT-I	CBT	2017	China	55.6	9.1	111	91	1. formal_DSM	0. primary_insomnia	111	4
Wong2021	CBTI Workshop	CBT	2021	China	38.2	15.8	70	46	1. formal_DSM	1. primary_secondary_mixed	70	12
Wong2021	Selfhelp CBTI	CBT	2021	China	36.9	14.7	70	44	1. formal_DSM	1. primary_secondary_mixed	70	7
Wong2021	SHE	PE	2021	China	39.6	16.3	70	55	1. formal_DSM	1. primary_secondary_mixed	70	10
Wu2006	Combined	CBT	2006	China	38.0	12.0	19	10	1. formal_DSM	0. primary_insomnia	19	9

© 2024 Furukawa Y et al. *JAMA Psychiatry.*

trial	Arm	treatment	Year	Country	Age_m	Age_sd	Age_n	F_n	Insomnia diagnosis	Comorbidities	n	r
Wu2006	PCT	PE	2006	China	38.0	12.0	20	11	1. formal_DSM	0. primary_insomnia	20	6
Xing2020	EA/CBT	CBT	2020	China	54.5	12.1	31	25	1. formal_DSM	1. primary_secondary_mixed	31	13
Xing2020	EA	TAU	2020	China	56.3	9.0	31	28	1. formal_DSM	1. primary_secondary_mixed	31	5
Yamamoto2016	Intervention	BT	2016	Japan	38.0	9.1	65	6	2. informal_AIS	1. primary_secondary_mixed	65	48
Yamamoto2016	Control	WL	2016	Japan	37.5	8.9	65	7	2. informal_AIS	1. primary_secondary_mixed	65	47
Yang2022	CBT-I	CBT	2022	South Korea	20.50	3.31	29	26	2. informal_ISI	3. phy_others	30	17
Yang2022	Control	PE	2022	South Korea	20.42	2.31	30	26	2. informal_ISI	3. phy_others	30	3
Yang2023	Self-help	CBT	2023	Taiwan	22.6	2.7	31	23	2. informal_ISI	0. primary_insomnia	31	2
Yang2023	Feedback	CBT	2023	Taiwan	22.6	3.0	31	22	2. informal_ISI	0. primary_insomnia	31	6
Yang2023	Waitlist	WL	2023	Taiwan	22.0	1.6	30	22	2. informal_ISI	0. primary_insomnia	30	0
Zachariades2012	Intervention	CBT	2012	Canada	48.0	10.2	23	15	1. formal_DSM	3. phy_others	23	1
Zachariades2012	Control	WL	2012	Canada	44.3	9.6	25	14	1. formal_DSM	3. phy_others	25	0
Zachariae2018	Intervention	CBT	2018	Denmark	53.2	8.8	133	133	2. informal_PSQI	3. phy_cancer	133	71
Zachariae2018	Control	WL	2018	Denmark	52.9	8.9	122	122	2. informal_PSQI	3. phy_cancer	122	19
Zakiei2021	Intervention	CT	2021	Iran	41.5	8.7	17	10	1. formal_DSM	1. primary_secondary_mixed	20	10
Zakiei2021	Control	PLB	2021	Iran	41.5	7.5	18	12	1. formal_DSM	1. primary_secondary_mixed	20	1

© 2024 Furukawa Y et al. *JAMA Psychiatry.*

trial	Arm	treatment	Year	Country	Age_m	Age_sd	Age_n	F_n	Insomnia diagnosis	Comorbidities	n	r
Zhang2015	MBSR	CT	2015	China	78.6	2.9	30	14	1. formal_DSM	1. primary_secondary_mixed	30	5
Zhang2015	Wait-list	WL	2015	China	77.6	3.0	30	11	1. formal_DSM	1. primary_secondary_mixed	30	1
Zhang2019	MBSR	CT	2019	China	57.8	11.1	35	35	1. formal_Edinger2004	3. phy_cancer	35	10
Zhang2019	UC	PE	2019	China	58.5	11.1	35	35	1. formal_Edinger2004	3. phy_cancer	35	3
Zhang2023	DCBT-I	CBT	2023	China	49.6	13.0	38	30	1. formal_ICSD	0. primary_insomnia	41	7
Zhang2023	Sleep education	BT	2023	China	50.6	14.1	39	26	1. formal_ICSD	0. primary_insomnia	41	3
Zhao2020	MBCT-I	CT	2020	China	52.8	6.5	68	68	1. formal_DSM	3. phy_cancer	68	2
Zhao2020	Waitlist	WL	2020	China	53.3	6.5	68	68	1. formal_DSM	3. phy_cancer	68	0
Zhou2022a	SHUTi-BWHS+SHUTi-normal	CBT	2022	USA	59.7	8.2	218	218	2. informal_ISI	1. primary_secondary_mixed	218	65
Zhou2022a	Control	PE	2022	USA	59.2	7.7	115	115	2. informal_ISI	1. primary_secondary_mixed	115	5
Zhou2022b	eCBT-I	CBT	2022	China	31.0	4.4	60	59	1. formal_ICSD	1. primary_secondary_mixed	60	34
Zhou2022b	Control	NT	2022	China	29.6	4.5	68	57	1. formal_ICSD	1. primary_secondary_mixed	58	13

Arm = arm name as described in the study. treatment = the treatmen group name as defined in this study. Age_m = mean age (years). Age_sd = standard deviation of age. Age_n = number used for calculating the mean age. F_n = number of females. n = number randomized. r = remission (imputed, if necessary)

© 2024 Furukawa Y et al. *JAMA Psychiatry.*

eTable 3. Evaluation of components

3.1. Percentage agreement and kappa of components for each arm of included trials

	Percentage agreement	Kappa
**Educational components**		
Sleep hygiene education (se)	87.8%	0.74
Sleep diary (sd)	80.3%	0.43
**Cognitive components**		
Cognitive restructuring (cr)	92.3%	0.83
Third wave components (th)	96.5%	0.76
Constructive worry (cw)	96.9%	0.50
**Behavioural components**		
Sleep restriction (sr)	92.7%	0.85
Stimulus control (sc)	92.5%	0.85
Paradoxical intention (pi)	98.8%	0.79
Relaxation (re)	89.0%	0.74
**Others**		
Non-specific treatment effect (ns)	84.3%	0.58
Waiting component (w)	94.4%	0.81
Conventional drug treatment (dt)	73.1%	0.45
**Delivery methods**		
Individual (ind)	81.1%	0.62
Group (gp)	93.7%	0.77
Face-to-face (ff)	88.8%	0.78
Therapeutic guidance (tg)	94.6%	0.70
Human encouragement (he)	93.7%	0.62
Automated encouragement (ae)	93.4%	0.56

© 2024 Furukawa Y et al. *JAMA Psychiatry.*

3.2. Components of the included trials

Trial	Arm	Treatment	Year	se	sd	cr	th	cw	sr	sc	pi	re	ns	w	dt	ind	gp	ff	tg	he	ae
Abbasi2016	Intervention	CBT	2016	1	0	1	0	0	1	0	0	1	1	0	1	1	1	1	0	0	0
Abbasi2016	Control	WL	2016	0	0	0	0	0	0	0	0	0	0	1	1	1	1	1	0	0	0
Abdelaziz2021	Study	CBT	2021	1	1	1	1	1	1	1	0	1	1	0	0	0	0	0	1	1	0
Abdelaziz2021	Control	PE	2021	0	1	0	0	0	0	0	0	0	0	0	0	0	0	0	0	0	0
Ahorsu2020	CBT-I	CBT	2020	1	1	1	1	0	0	1	1	1	1	0	0	0	0	0	0	1	0
Ahorsu2020	PE	WL	2020	1	1	0	0	0	0	0	0	0	1	1	0	0	0	0	0	0	0
Alessi2016	Individual	CBT	2016	1	1	1	0	0	1	1	0	0	1	0	1	1	0	1	0	0	0
Alessi2016	Group	CBT	2016	1	1	1	0	0	1	1	0	0	1	0	1	0	1	1	0	0	0
Alessi2016	Control	PE	2016	1	1	0	0	0	0	0	0	0	1	0	1	0	1	1	0	0	0
Alshehri2020	CBT-I	CBT	2020	1	1	1	1	0	1	1	0	1	1	0	1	1	0	1	0	0	0
Alshehri2020	HE	PE	2020	1	1	0	0	0	0	0	0	0	1	0	1	1	0	1	0	0	0
Amra2022	Intervention	CBT	2022	1	1	1	0	0	1	1	0	0	1	0	1	0	1	1	0	0	0
Amra2022	Control	NT	2022	0	0	0	0	0	0	0	0	0	0	0	1	0	0	0	0	0	0
Arnedt2011	CBTI-AD	CBT	2011	1	1	1	0	0	1	1	0	0	1	0	1	1	0	1	0	0	0
Arnedt2011	BPT	PE	2011	1	1	0	0	0	0	0	0	0	1	0	1	1	0	1	0	0	0
Arnedt2021	CBT-TM	CBT	2021	1	1	1	0	1	1	1	0	1	1	0	1	1	0	0	0	0	0
Arnedt2021	CBT-F2F	CBT	2021	1	1	1	0	1	1	1	0	1	1	0	1	1	0	1	0	0	0
Arnedt2023	CBT-CB-TM	CBT	2023	1	1	1	0	1	1	1	0	1	1	0	1	1	0	0	0	0	0
Arnedt2023	SHE-TM	PE	2023	1	1	0	0	0	0	0	0	0	1	0	1	1	0	0	0	0	0
Ashworth2015	CBT-I	CBT	2015	1	1	1	1	1	1	1	1	1	1	0	0	1	0	1	0	0	0
Ashworth2015	Self-help	CBT	2015	1	1	1	1	1	1	1	1	1	1	1	0	0	0	0	0	0	0

© 2024 Furukawa Y et al. *JAMA Psychiatry.*

Trial	Arm	Treatment	Year	se	sd	cr	th	cw	sr	sc	pi	re	ns	w	dt	ind	gp	ff	tg	he	ae
Ayabe2018	CBT-I + TAU	BT	2018	1	1	0	0	0	1	1	0	1	1	0	1	1	0	1	0	0	0
Ayabe2018	TAU	WL	2018	1	1	0	0	0	0	0	0	0	1	1	1	1	0	1	0	0	0
Ballou2020	BBT	BT	2020	1	1	0	0	0	1	1	0	0	1	0	1	1	0	1	0	0	0
Ballou2020	Control	WL	2020	0	1	0	0	0	0	0	0	0	1	1	1	1	0	1	0	0	0
Bastein2004	Group	CBT	2004	1	1	1	0	0	1	1	0	0	1	0	0	0	1	1	0	0	0
Bastein2004	Telephone	CBT	2004	1	1	1	0	0	1	1	0	0	1	0	0	1	0	0	0	0	0
Bastein2004	Individual	CBT	2004	1	1	1	0	0	1	1	0	0	1	0	0	1	0	1	0	0	0
Behera2023	HMBCT	CBT	2023	1	1	0	1	0	1	1	0	0	1	0	0	1	0	1	0	0	0
Behera2023	Control	BT	2023	1	1	0	0	0	1	1	0	1	1	0	0	1	0	1	0	0	0
Bernstein2017	Intervention	CBT	2017	1	1	1	1	0	1	1	0	1	1	0	1	0	0	0	0	0	1
Bernstein2017	Control	WL	2017	0	0	0	0	0	0	0	0	0	0	1	1	0	0	0	0	0	0
Bothelius2013	CBTI	CBT	2013	1	1	1	0	0	1	1	1	1	1	0	1	1	1	0	0	0	0
Bothelius2013	WL	WL	2013	0	1	0	0	0	0	0	0	0	0	1	1	0	0	0	0	0	0
Bramoweth2020	BBTI	BT	2020	1	1	0	0	0	1	1	0	0	1	0	1	1	0	1	0	0	0
Bramoweth2020	CBTI	CBT	2020	1	1	1	0	0	1	1	0	1	1	0	1	1	0	1	0	0	0
Broomfield2003	PI	BT	2003	0	1	0	0	0	0	0	1	0	1	0	0	1	0	1	0	0	0
Broomfield2003	C	PE	2003	0	1	0	0	0	0	0	0	0	0	0	0	0	0	0	0	0	0
Buysse2011	BBTI	BT	2011	1	1	0	0	0	1	1	0	0	1	0	1	1	0	1	0	0	0
Buysse2011	IC	WL	2011	1	1	0	0	0	0	0	0	0	1	1	1	0	0	0	0	1	0
Cai2022	mindfulness	CT	2022	0	0	0	1	0	0	0	0	0	1	0	1	1	0	1	0	0	0
Cai2022	Health education	WL	2022	1	0	0	0	0	0	0	0	0	1	1	1	1	0	1	0	0	0
Cape2016	CBT-I	CBT	2016	1	0	1	0	0	1	1	0	1	1	0	1	0	1	1	0	0	0
Cape2016	TAU	PE	2016	1	0	0	0	0	0	0	0	0	1	0	1	1	0	1	0	0	0
Carney2017	CBT+AD	CBT	2017	1	1	1	0	0	1	1	0	0	1	0	0	1	0	1	0	0	0
Carney2017	AD+SH	PE	2017	1	1	0	0	0	0	0	0	0	1	0	0	1	0	1	0	0	0
Carrera1980	Ocean tape	RT	1980	0	0	0	0	0	0	0	0	1	1	0	0	0	0	0	0	0	0

© 2024 Furukawa Y et al. *JAMA Psychiatry.*

Trial	Arm	Treatment	Year	se	sd	cr	th	cw	sr	sc	pi	re	ns	w	dt	ind	gp	ff	tg	he	ae
Carrera1980	Wait-control	WL	1980	0	0	0	0	0	0	0	0	0	0	1	0	0	0	0	0	0	0
Casault2015	mCBT-I	CBT	2015	1	1	1	0	0	1	1	0	0	1	0	1	0	0	0	1	1	0
Casault2015	CTL	WL	2015	0	1	0	0	0	0	0	0	0	0	1	1	0	0	0	0	0	0
Chakravorty2019	CBT-I	CBT	2019	1	1	1	0	0	1	1	0	0	1	0	1	1	0	1	0	0	0
Chakravorty2019	MO	PE	2019	0	1	0	0	0	0	0	0	0	1	0	1	1	0	1	0	0	0
Chan2021	Tx	CBT	2021	1	1	1	0	1	1	1	0	1	1	0	0	0	0	0	0	0	1
Chan2021	WL	WL	2021	0	0	0	0	0	0	0	0	0	0	1	0	0	0	0	0	0	0
Chao2021	CBT-I	CBT	2021	1	1	1	0	0	1	1	0	0	1	0	1	1	0	0	0	0	0
Chao2021	Waitlist	WL	2021	0	1	0	0	0	0	0	0	0	0	1	1	0	0	0	0	1	0
Chapoutot2020	ACT-E-CBT	CBT	2020	1	1	1	1	0	1	1	0	1	1	0	1	1	0	0	0	0	0
Chapoutot2020	WLC	WL	2020	0	1	0	0	0	0	0	0	0	0	1	1	0	0	0	0	0	0
Cheng2019	dCBT-I	CBT	2019	1	1	1	0	0	1	1	1	1	1	0	1	0	0	0	0	1	1
Cheng2019	Sleep education	PE	2019	1	0	0	0	0	0	0	0	0	1	0	1	0	0	0	0	0	0
Christensen2016	SHUTi	CBT	2016	1	1	1	0	0	1	1	0	0	1	0	1	0	0	0	0	0	1
Christensen2016	HealthWatch	PE	2016	0	1	0	0	0	0	0	0	0	1	0	1	0	0	0	0	0	1
Chung2023	CBTI	CBT	2023	1	1	1	0	0	1	1	0	1	1	0	1	0	1	1	0	0	0
Chung2023	No CBTI	NT	2023	0	0	0	0	0	0	0	0	0	0	0	1	0	0	0	0	0	0
Craner2022	IPRP-UC	CBT	2022	1	0	0	1	0	0	0	0	1	1	1	1	1	0	1	0	0	0
Craner2022	IPRP+CBT-I	CBT	2022	1	1	1	1	0	1	1	0	1	1	0	1	1	1	1	0	0	0
Currie2000	CBT	CBT	2000	1	1	1	0	0	1	1	0	1	1	0	1	0	1	1	0	0	0
Currie2000	WLC	WL	2000	0	1	0	0	0	0	0	0	0	0	1	1	0	0	0	0	1	0
Currie2004	Self-help manual	CBT	2004	1	1	1	0	0	1	1	0	1	1	0	1	0	0	0	1	1	0
Currie2004	Individual therapy	CBT	2004	1	1	1	0	0	1	1	0	1	1	0	1	1	0	1	0	0	0
Currie2004	Wait-list	WL	2004	0	1	0	0	0	0	0	0	0	0	1	1	0	0	0	0	0	0
Daren2012	Intervention	BT	2012	1	1	0	0	0	1	1	0	0	1	0	1	1	0	1	0	0	0
Daren2012	Control	PE	2012	1	1	0	0	0	0	0	0	0	0	0	1	0	0	0	0	0	0

© 2024 Furukawa Y et al. *JAMA Psychiatry.*

Trial	Arm	Treatment	Year	se	sd	cr	th	cw	sr	sc	pi	re	ns	w	dt	ind	gp	ff	tg	he	ae
Dean2019	Experimental	BT	2019	1	1	0	0	0	1	1	0	0	1	0	1	1	0	1	0	0	0
Dean2019	Control	PE	2019	0	1	0	0	0	0	0	0	0	1	0	1	1	0	1	0	0	0
Dekker2020	ICBTI w 1-4	CBT	2020	1	1	1	0	0	1	1	0	1	1	0	0	0	0	0	1	1	0
Dekker2020	ICBTI w 6-9	WL	2020	0	1	0	0	0	0	0	0	0	1	1	0	0	0	0	0	0	0
Dirksen2008	CBT-I	BT	2008	1	1	0	0	0	1	1	0	0	1	0	1	1	1	1	0	0	0
Dirksen2008	CC	PE	2008	1	1	0	0	0	0	0	0	0	1	0	1	1	1	1	0	0	0
Drake2019	SRT	BT	2019	0	1	0	0	0	1	0	0	0	1	0	0	1	0	1	0	0	0
Drake2019	CBTI	CBT	2019	1	1	1	0	0	1	1	0	1	1	0	0	1	0	1	0	0	0
Drake2019	SHE	PE	2019	1	1	0	0	0	0	0	0	0	1	0	0	0	0	0	0	0	0
Dyrberg2022	CBT-I	CBT	2022	1	1	1	0	0	1	1	0	1	1	0	1	1	1	1	0	0	0
Dyrberg2022	TAU	WL	2022	1	1	0	0	0	0	0	0	0	1	1	1	1	0	1	0	0	0
Edinger2001	CBT	CBT	2001	1	1	1	0	0	1	1	0	0	1	0	0	1	0	1	0	0	0
Edinger2001	Placebo therapy	PE	2001	0	1	0	0	0	0	0	0	0	1	0	0	1	0	1	0	0	0
Edinger2001	Relaxation training	RT	2001	0	1	0	0	0	0	0	0	1	1	0	0	1	0	1	0	0	0
Edinger2003	ACBT	BT	2003	1	1	0	0	0	1	1	0	0	1	0	0	1	0	1	0	0	0
Edinger2003	SHC	PE	2003	1	1	0	0	0	0	0	0	0	1	0	0	1	0	1	0	0	0
Edinger2005	CBT	CBT	2005	1	1	1	0	0	1	1	0	0	1	0	1	1	0	1	0	0	0
Edinger2005	SH	PE	2005	1	1	0	0	0	0	0	0	0	1	0	1	1	0	1	0	0	0
Edinger2005	UC	WL	2005	0	1	0	0	0	0	0	0	0	0	1	1	1	0	1	0	0	0
Edinger2007	CBT combined	BT	2007	1	1	0	0	0	1	1	0	0	1	0	0	1	0	1	0	0	0
Edinger2007	WL	WL	2007	0	1	0	0	0	0	0	0	0	0	1	0	0	0	0	0	0	0
Edinger2009	CBT	CBT	2009	1	1	1	0	0	1	1	0	0	1	0	1	1	0	1	0	0	0
Edinger2009	SH	PE	2009	1	1	0	0	0	0	0	0	0	1	0	1	1	0	1	0	0	0
ElRafahiFerreira2021	ACT-BBI-I	CBT	2021	1	1	0	1	0	1	1	0	0	1	0	1	0	1	1	0	0	0
ElRafahiFerreira2021	CBT-I	CBT	2021	1	1	1	0	0	1	1	0	0	1	0	1	0	1	1	0	0	0
ElRafahiFerreira2022	CBT-I	CBT	2022	1	1	1	0	0	1	1	0	0	1	0	1	0	1	1	0	0	0

© 2024 Furukawa Y et al. *JAMA Psychiatry.*

Trial	Arm	Treatment	Year	se	sd	cr	th	cw	sr	sc	pi	re	ns	w	dt	ind	gp	ff	tg	he	ae
ElRafahiFerreira2022	ACT-I	CT	2022	1	1	0	1	0	0	0	0	0	1	0	1	0	1	1	0	0	0
Epstein2007	Multicomponent intervention	BT	2007	1	1	0	0	0	1	1	0	0	1	0	1	1	1	1	0	0	0
Epstein2007	Control	PE	2007	1	1	0	0	0	0	0	0	0	1	0	1	1	1	1	0	0	0
Eptein2012	SCT	BT	2012	1	1	0	0	0	0	1	0	0	1	0	0	1	1	1	0	0	0
Eptein2012	SRT	BT	2012	1	1	0	0	0	1	0	0	0	1	0	0	1	1	1	0	0	0
Eptein2012	MCI	BT	2012	1	1	0	0	0	1	1	0	0	1	0	0	1	1	1	0	0	0
Eptein2012	WLC	WL	2012	0	1	0	0	0	0	0	0	0	0	1	0	0	0	0	0	0	0
Espie1989	Paradoxical intention	BT	1989	0	1	0	0	0	0	0	1	0	1	0	1	1	0	1	0	0	0
Espie1989	Stimulus control	BT	1989	0	1	0	0	0	0	1	0	0	1	0	1	1	0	1	0	0	0
Espie1989	Placebo	PE	1989	0	1	0	0	0	0	0	0	0	1	0	1	1	0	1	0	0	0
Espie1989	Progressive relaxation	RT	1989	0	1	0	0	0	0	0	0	1	1	0	1	1	0	1	0	0	0
Espie1989	No treatment	WL	1989	0	1	0	0	0	0	0	0	0	1	1	1	1	0	1	0	0	0
Espie2001	CBT	CBT	2001	1	1	1	0	0	1	1	0	1	1	0	1	0	1	1	0	0	0
Espie2001	SMC	WL	2001	0	1	0	0	0	0	0	0	0	1	1	1	0	0	0	0	0	0
Espie2007	CBT	CBT	2007	1	1	1	0	0	1	1	0	1	1	0	1	0	1	1	0	0	0
Espie2007	TAU	PE	2007	0	1	0	0	0	0	0	0	0	0	0	1	0	0	0	0	0	0
Espie2008	CBT	CBT	2008	1	1	1	0	0	1	1	0	1	1	0	1	0	1	1	0	0	0
Espie2008	TAU	PE	2008	0	1	0	0	0	0	0	0	0	0	0	1	0	0	0	0	0	0
Espie2012	CBT	CBT	2012	1	1	1	1	0	1	1	1	1	1	0	1	0	0	0	0	1	1
Espie2012	IRT	WL	2012	1	1	0	0	0	0	0	0	0	1	1	1	0	0	0	0	0	1
Espie2012	TAU	WL	2012	0	1	0	0	0	0	0	0	0	0	1	1	0	0	0	0	0	1
Espie2019	dCBT	CBT	2019	1	1	1	1	0	1	1	1	1	1	0	1	0	0	0	0	1	1
Espie2019	SHE	PE	2019	1	0	0	0	0	0	0	0	0	1	0	1	0	0	0	0	0	0
Falloon2015	SSR	BT	2015	1	1	0	0	0	1	0	0	0	1	0	0	1	0	1	0	0	0
Falloon2015	Control	PE	2015	1	1	0	0	0	0	0	0	0	1	0	0	1	0	1	0	0	0
Farsani2021	CBT-I	CBT	2021	1	1	1	0	0	1	1	0	1	1	0	0	0	1	1	0	0	0

© 2024 Furukawa Y et al. *JAMA Psychiatry.*

Trial	Arm	Treatment	Year	se	sd	cr	th	cw	sr	sc	pi	re	ns	w	dt	ind	gp	ff	tg	he	ae
Farsani2021	Control	WL	2021	0	1	0	0	0	0	0	0	0	0	1	0	0	0	0	0	0	0
Felder2020	digital CBTI	CBT	2020	1	1	1	0	0	1	1	0	1	1	0	1	0	0	0	0	1	1
Felder2020	Standard treatment	WL	2020	0	1	0	0	0	0	0	0	0	0	1	1	0	0	0	0	0	0
Fernando2013	intervention	BT	2013	1	1	0	0	0	1	0	0	0	1	0	0	1	0	1	0	0	0
Fernando2013	control	PE	2013	1	1	0	0	0	0	0	0	0	1	0	0	1	0	1	0	0	0
Feuerstein2017	cb-CBT-I	CBT	2017	1	1	1	1	0	1	1	0	1	1	0	1	0	0	0	0	1	0
Feuerstein2017	Sleep Diary	PE	2017	0	1	0	0	0	0	0	0	0	1	0	1	0	0	0	0	1	0
Ford2022	eCBT-I	CBT	2022	1	1	1	1	0	1	1	0	1	1	0	1	1	0	1	1	1	0
Ford2022	TAU	WL	2022	0	1	0	0	0	0	0	0	0	1	1	1	1	0	1	0	0	0
Fowler2009	CBT	CBT	2009	1	1	1	0	1	1	1	0	0	1	0	1	0	0	0	1	1	0
Fowler2009	SC	PE	2009	0	1	0	0	0	0	0	0	0	0	0	1	0	0	0	0	0	0
Freeman2015	CBT	CBT	2015	1	1	1	0	0	0	1	0	1	1	0	1	1	0	1	0	0	0
Freeman2015	standard care	PE	2015	0	1	0	0	0	0	0	0	0	0	0	1	0	0	0	0	0	0
Freeman2017	Treatment	CBT	2017	1	1	1	1	0	1	1	1	1	1	0	1	0	0	0	0	1	1
Freeman2017	Contrl	TAU	2017	0	0	0	0	0	0	0	0	0	0	0	1	0	0	0	0	0	0
Fucito2014	CBT-I+SC	CBT	2014	1	1	1	0	0	1	1	0	1	1	0	0	1	0	1	0	0	0
Fucito2014	SC	PE	2014	1	1	0	0	0	0	0	0	0	1	0	0	1	0	1	0	0	0
Garcia2018	Intervention	CBT	2018	0	0	0	1	0	0	0	0	1	1	0	0	1	0	1	0	0	0
Garcia2018	Control	PLB	2018	0	0	0	0	0	0	0	0	0	1	0	0	1	0	1	0	0	0
Garland2014	CBT-I	CBT	2014	0	1	1	0	0	1	1	0	1	1	0	1	0	1	1	0	0	0
Garland2014	MBSR	CBT	2014	0	1	0	1	0	0	0	0	1	1	0	1	0	1	1	0	0	0
Gebara2019	Immediate BBTI	BT	2019	0	1	0	0	0	1	1	0	0	1	0	1	1	0	1	0	0	0
Gebara2019	Delayed BBTI	WL	2019	0	0	0	0	0	0	0	0	0	0	1	1	0	0	0	0	0	0
Gehrman2020	In Person	CBT	2020	1	1	1	0	0	1	1	0	1	1	0	1	0	1	1	0	0	0
Gehrman2020	Telehealth	CBT	2020	1	1	1	0	0	1	1	0	1	1	0	1	0	1	0	0	0	0
Gehrman2021	In-person	CBT	2021	1	1	1	0	0	1	1	0	1	1	0	0	1	0	1	0	0	0

© 2024 Furukawa Y et al. *JAMA Psychiatry.*

Trial	Arm	Treatment	Year	se	sd	cr	th	cw	sr	sc	pi	re	ns	w	dt	ind	gp	ff	tg	he	ae
Gehrman2021	Telehealth	CBT	2021	1	1	1	0	0	1	1	0	1	1	0	0	1	0	0	0	0	0
Gehrman2021	Waitlist	WL	2021	0	0	0	0	0	0	0	0	0	0	1	0	0	0	0	0	0	0
Germain2014	BBTI-MV	BT	2014	1	1	0	0	0	1	1	0	0	1	0	1	1	0	1	0	0	0
Germain2014	Information Control	WL	2014	1	1	0	0	0	0	0	0	0	1	1	1	1	0	1	0	0	0
Gieselmann2019	F2F	BT	2019	1	1	0	0	0	1	0	0	0	1	0	1	1	0	1	0	0	0
Gieselmann2019	Chat	BT	2019	1	1	0	0	0	1	0	0	0	1	0	1	1	0	0	0	0	0
Gieselmann2019	WL	WL	2019	0	1	0	0	0	0	0	0	0	0	1	1	0	0	0	0	0	0
Glozier2019	SHUTi	CBT	2019	1	1	1	0	0	1	1	0	0	1	0	1	0	0	0	0	0	1
Glozier2019	Control	PE	2019	1	1	0	0	0	0	0	0	0	1	0	1	0	0	0	0	0	0
Greeff1998	Treatment	RT	1998	0	1	0	0	0	0	0	0	1	1	0	0	1	0	1	0	0	0
Greeff1998	Control	WL	1998	0	1	0	0	0	0	0	0	0	0	1	0	0	0	0	0	0	0
Guarnaccia2023	MBS-I	CT	2023	0	0	0	1	0	0	0	0	0	1	0	1	0	1	1	0	0	0
Guarnaccia2023	SH	PE	2023	1	1	0	0	0	0	0	0	0	1	0	1	0	1	1	0	0	0
Hagatun2019	SHUTi	CBT	2019	1	1	1	0	0	1	1	0	0	1	0	0	0	0	0	0	0	1
Hagatun2019	Patient education	PE	2019	1	1	0	0	0	0	0	0	0	1	0	0	0	0	0	0	0	0
Hall2022	CBTI	CBT	2022	1	1	1	0	1	1	1	0	1	1	0	1	1	0	1	0	0	0
Hall2022	EUC	PE	2022	1	1	0	0	0	0	0	0	0	1	0	1	0	0	0	0	0	0
Ham2020	Experimetal	CBT	2020	1	1	1	0	0	1	1	0	1	1	0	0	1	1	1	0	0	0
Ham2020	Control	PE	2020	1	0	0	0	0	0	0	0	0	1	0	0	0	1	1	0	0	0
Harris2012-1	ISR+SCT	BT	2012	1	1	0	0	0	0	1	0	0	1	0	0	1	0	1	0	0	0
Harris2012-1	ISR	PE	2012	1	1	0	0	0	0	0	0	0	1	0	0	1	0	1	0	0	0
Harris2012-2	SCT	BT	2012	1	1	0	0	0	0	1	0	0	1	0	0	1	0	1	0	0	0
Harris2012-2	CONTROL	PE	2012	1	1	0	0	0	0	0	0	0	1	0	0	1	0	1	0	0	0
Harris2019	BI	BT	2019	1	1	0	0	0	1	1	0	0	1	0	0	1	0	1	0	0	0
Harris2019	SMC	PE	2019	0	1	0	0	0	0	0	0	0	0	0	0	0	0	0	0	0	0
Harvey2014	BT	BT	2014	0	1	0	0	0	1	1	0	0	1	0	1	1	0	1	0	0	0

© 2024 Furukawa Y et al. *JAMA Psychiatry.*

Trial	Arm	Treatment	Year	se	sd	cr	th	cw	sr	sc	pi	re	ns	w	dt	ind	gp	ff	tg	he	ae
Harvey2014	CBT	CBT	2014	0	1	1	0	0	1	1	0	0	1	0	1	1	0	1	0	0	0
Harvey2014	CT	CT	2014	0	1	1	0	0	0	0	0	0	1	0	1	1	0	1	0	0	0
Harvey2015	CBTI-BP	CBT	2015	1	1	1	0	0	1	1	0	0	1	0	1	1	0	1	0	0	0
Harvey2015	PE	RT	2015	0	1	0	0	0	0	0	0	1	1	0	1	1	0	1	0	0	0
Ho2014	SHS	CBT	2014	1	1	1	0	0	1	1	0	1	1	0	1	1	0	0	1	1	0
Ho2014	SH	CBT	2014	1	1	1	0	0	1	1	0	1	1	0	1	0	0	0	0	0	0
Ho2014	WL	WL	2014	0	1	0	0	0	0	0	0	0	0	1	1	0	0	0	0	0	0
Ho2021	CBT	CBT	2021	1	1	1	0	1	1	1	0	1	1	0	0	0	1	1	0	0	0
Ho2021	WL	WL	2021	0	0	0	0	0	0	0	0	0	0	1	0	0	0	0	0	0	0
Holmqvist2014	Telehealth-based	CBT	2014	1	1	1	1	0	1	1	0	1	1	0	0	0	1	0	0	0	0
Holmqvist2014	Web-based	CBT	2014	1	1	1	1	0	1	1	0	1	1	0	0	0	0	0	0	0	0
Horsch2017	App	BT	2017	1	1	0	0	0	1	1	0	1	1	0	1	0	0	0	0	0	1
Horsch2017	Wl	WL	2017	0	1	0	0	0	0	0	0	0	0	1	1	0	0	0	0	0	0
Hou2014	Treatment	BT	2014	0	0	0	0	0	1	1	0	1	1	0	1	0	1	1	0	0	0
Hou2014	Control	NT	2014	0	0	0	0	0	0	0	0	0	0	0	1	0	0	0	0	0	0
Jacobs1993	SC	BT	1993	0	1	0	0	0	0	1	0	0	1	0	0	1	0	1	0	0	0
Jacobs1993	MF	BT	1993	0	1	0	0	0	0	1	0	1	1	0	0	1	0	1	0	0	0
Jacobs2004	Combination	CBT	2004	1	1	1	0	0	1	1	0	1	1	0	1	1	0	1	0	0	0
Jacobs2004	Pharmacotherapy	PE	2004	1	1	0	0	0	0	0	0	0	1	0	1	1	0	1	0	0	0
JanssonFrojmark2012a	BT	BT	2012	0	1	0	0	0	1	1	0	0	1	0	1	1	0	1	0	0	0
JanssonFrojmark2012a	BT+CW	CBT	2012	0	1	0	0	1	1	1	0	0	1	0	1	1	0	1	0	0	0
JanssonFrojmark2012b	CBT-I	CBT	2012	1	1	1	0	1	1	1	0	1	1	0	0	1	0	1	0	0	0
JanssonFrojmark2012b	WLC	WL	2012	0	1	0	0	0	0	0	0	0	0	1	0	0	0	0	0	0	0
Jarnefelt2020	Group-based CBT-I	CBT	2020	1	1	1	1	1	1	1	0	1	1	0	1	0	1	1	0	0	0
Jarnefelt2020	Self-help-based CBT-I	CBT	2020	1	1	1	1	1	1	1	0	1	1	0	1	0	0	0	1	0	0
Jarnefelt2020	Sleep hygiene education	PE	2020	1	1	0	0	0	0	0	0	0	1	0	1	0	0	0	0	0	0

© 2024 Furukawa Y et al. *JAMA Psychiatry.*

Trial	Arm	Treatment	Year	se	sd	cr	th	cw	sr	sc	pi	re	ns	w	dt	ind	gp	ff	tg	he	ae
Javaheri2020	Intervention	CBT	2020	1	1	1	0	0	1	1	0	1	1	0	1	0	0	0	0	0	1
Javaheri2020	Control	PE	2020	0	1	0	0	0	0	0	0	0	1	0	1	0	0	0	0	0	0
Jernelov2012	Bibliotherapy with support	CBT	2012	1	1	1	0	0	1	1	0	1	1	0	1	0	0	0	1	1	0
Jernelov2012	Bibliotherapy	CBT	2012	1	1	1	0	0	1	1	0	1	1	0	1	0	0	0	0	0	0
Jernelov2012	Wait list	WL	2012	0	1	0	0	0	0	0	0	0	0	1	1	0	0	0	0	0	0
Johann2020	Treatment	CBT	2020	1	1	1	0	1	1	1	1	1	1	0	0	1	0	1	0	0	0
Johann2020	Control	WL	2020	0	1	0	0	0	0	0	0	0	0	1	0	0	0	0	0	0	0
Jungquist2010	Treatment	CBT	2010	1	1	1	0	0	1	1	0	0	1	0	0	1	0	1	0	0	0
Jungquist2010	Control	PE	2010	0	1	0	0	0	0	0	0	0	1	0	0	1	0	1	0	0	0
Kaku2012	Intervention	BT	2012	1	0	0	0	0	1	1	0	1	1	0	0	1	0	1	0	0	0
Kaku2012	Control	WL	2012	0	0	0	0	0	0	0	0	0	0	1	0	0	0	0	0	0	0
Kaldo2015	Control	CBT	2015	1	1	0	1	0	0	0	0	1	1	0	1	0	0	0	0	0	1
Kaldo2015	Treatment	CBT	2015	1	1	1	0	0	1	1	0	0	1	0	1	0	0	0	1	0	1
Kaldo2020	Seflhelp book-group	CBT	2020	1	1	1	1	0	1	1	0	1	1	0	1	0	1	1	1	1	0
Kaldo2020	CAU	WL	2020	0	1	0	0	0	0	0	0	0	0	1	1	0	0	0	0	0	0
Kallestad2021	FtF CBT-I	CBT	2021	1	1	1	0	0	1	1	0	0	1	0	1	1	0	1	0	0	0
Kallestad2021	dCBT-I	CBT	2021	1	1	1	0	0	1	1	0	0	1	0	1	0	0	0	0	0	0
Kalmbach2020	CBT-I	CBT	2020	1	0	1	0	0	1	1	1	1	1	0	0	0	0	0	0	0	0
Kalmbach2020	Control	PE	2020	1	0	0	0	0	0	0	0	0	1	0	0	0	0	0	0	0	1
Kapella2011	CBT-I	CBT	2011	1	1	1	0	0	1	1	0	1	1	0	0	1	0	1	0	0	0
Kapella2011	WE	PE	2011	0	1	0	0	0	0	0	0	0	1	0	0	1	0	1	0	0	0
Kapella2022	CBT-I+AC or COPD-ED	CBT	2022	1	1	1	0	0	1	1	0	1	1	0	0	1	0	1	0	0	0
Kapella2022	AC+AC or COPD-ED	PE	2022	0	1	0	0	0	0	0	0	0	1	0	0	1	0	1	0	0	0
Kennet2021	Intervention	CBT	2021	1	0	1	1	0	1	1	0	1	1	0	0	0	0	0	0	0	1
Kennet2021	Waitlist	WL	2021	0	0	0	0	0	0	0	0	0	0	1	0	0	0	0	0	0	0
Krieger2019	SRT	BT	2019	1	1	0	0	0	1	0	0	0	1	0	1	0	0	0	1	1	0

© 2024 Furukawa Y et al. *JAMA Psychiatry.*

Trial	Arm	Treatment	Year	se	sd	cr	th	cw	sr	sc	pi	re	ns	w	dt	ind	gp	ff	tg	he	ae
Krieger2019	MCT	CBT	2019	1	1	1	0	0	1	0	0	1	1	0	1	0	0	0	1	1	0
Krieger2019	CAU	WL	2019	0	0	0	0	0	0	0	0	0	0	1	1	0	0	0	0	0	0
Kuhn2022	Insomnia Coach	CBT	2022	1	1	1	0	0	1	1	0	1	1	0	1	0	0	0	0	0	0
Kuhn2022	Wait-list	WL	2022	0	1	0	0	0	0	0	0	0	0	1	1	0	0	0	0	0	0
Kyle2020	dCBT	CBT	2020	1	1	1	1	0	1	1	1	1	1	0	1	0	0	0	0	0	1
Kyle2020	WLC	WL	2020	0	0	0	0	0	0	0	0	0	0	1	1	0	0	0	0	0	0
Lami2018	CBT-IP	CBT	2018	1	0	1	0	0	1	1	0	1	1	0	1	0	1	1	0	0	0
Lami2018	CBT-P	PLB	2018	0	0	0	0	0	0	0	0	0	1	0	1	0	1	1	0	0	0
Lancee2012	Electronic+Pen	CBT	2012	1	1	1	0	0	1	1	1	1	1	0	1	0	0	0	0	0	0
Lancee2012	Waitlist	WL	2012	0	1	0	0	0	0	0	0	0	0	1	1	0	0	0	0	0	0
Lancee2013	No support	CBT	2013	1	1	1	0	0	1	1	1	1	1	0	1	0	0	0	0	0	0
Lancee2013	Support	CBT	2013	1	1	1	0	0	1	1	1	1	1	0	1	0	0	0	1	1	0
Lancee2015	CBT-I	CBT	2015	1	1	1	0	0	1	1	0	1	1	0	1	0	0	0	1	1	0
Lancee2015	Wait-list	WL	2015	0	1	0	0	0	0	0	0	0	0	1	1	0	0	0	0	0	0
Lancee2016	f2f	CBT	2016	1	1	1	0	0	1	1	0	1	1	0	1	1	0	1	0	0	0
Lancee2016	Online	CBT	2016	1	1	1	0	0	1	1	0	1	1	0	1	0	0	0	1	0	0
Lancee2016	WL	WL	2016	0	1	0	0	0	0	0	0	0	0	1	1	0	0	0	0	0	0
Lappalainen2019	iACT	CBT	2019	1	1	0	1	0	0	1	0	0	1	0	1	0	0	0	0	0	1
Lappalainen2019	WLC	WL	2019	0	1	0	0	0	0	0	0	0	0	1	1	0	0	0	0	0	0
Latocha2022	CBTI	BT	2022	1	1	0	0	0	1	1	0	1	1	0	0	0	1	1	0	0	0
Latocha2022	UC	PE	2022	0	1	0	0	0	0	0	0	0	0	0	0	0	0	0	0	0	0
Leerssen2022-1	CBT-I	CBT	2022	1	1	1	0	0	1	1	0	1	1	0	1	0	0	0	1	1	0
Leerssen2022-1	NT	PE	2022	0	1	0	0	0	0	0	0	0	0	0	1	0	0	0	0	0	0
Leerssen2022-2	CBT-I+CRS	CBT	2022	1	1	1	0	0	1	1	0	1	1	0	1	0	0	0	1	1	0
Leerssen2022-2	CRS	PE	2022	0	1	0	0	0	0	0	0	0	1	0	1	0	0	0	1	1	0
Lichstein2000	Treatment	BT	2000	1	1	0	0	0	0	1	0	1	1	0	0	1	0	1	0	0	0

© 2024 Furukawa Y et al. *JAMA Psychiatry.*

Trial	Arm	Treatment	Year	se	sd	cr	th	cw	sr	sc	pi	re	ns	w	dt	ind	gp	ff	tg	he	ae
Lichstein2000	Control	WL	2000	0	1	0	0	0	0	0	0	0	0	1	0	0	0	0	0	0	0
Lichstein2001	Placebo	PE	2001	1	1	0	0	0	0	0	0	0	1	0	0	1	0	1	0	0	0
Lichstein2001	Relaxation	RT	2001	1	1	0	0	0	0	0	0	1	1	0	0	1	0	1	0	0	0
Liu2022-1	Control	NT	2022	0	0	0	0	0	0	0	0	0	0	0	0	0	0	0	0	0	0
Liu2022-1	MBSR	CT	2022	0	0	0	1	0	0	0	0	0	1	0	0	0	1	1	0	0	0
Liu2022-2	Acupressure	PLB	2022	0	0	0	0	0	0	0	0	0	1	0	0	0	0	0	0	0	0
Liu2022-2	MBSR + Acupressure	CT	2022	0	0	0	1	0	0	0	0	0	1	0	0	0	1	1	0	0	0
Lopez2019	eCBT-I	CBT	2019	1	1	1	1	0	1	1	0	1	1	0	1	1	0	0	0	0	0
Lopez2019	mPT	PE	2019	1	1	0	0	0	0	0	0	0	1	0	1	1	0	1	0	0	0
Lorenz2019	Treatment	CBT	2019	1	1	1	0	0	1	1	0	1	1	0	1	0	0	0	0	0	1
Lorenz2019	Control	WL	2019	0	0	0	0	0	0	0	0	0	0	1	1	0	0	0	0	0	0
Lovato2014	CBT-I	CBT	2014	1	1	1	0	0	1	0	0	0	1	0	0	0	1	1	0	0	0
Lovato2014	Waitlist	WL	2014	0	1	0	0	0	0	0	0	0	0	1	0	0	0	0	0	0	0
Low2020	Mindfulness	CT	2020	0	1	0	1	0	0	0	0	0	1	0	1	0	0	0	0	0	0
Low2020	PMR	RT	2020	0	1	0	0	0	0	0	0	1	1	0	1	0	0	0	0	0	0
Maguen2021	BBTI	BT	2021	1	1	0	0	0	1	1	0	0	1	0	1	1	0	1	0	0	0
Maguen2021	PMRT	RT	2021	0	1	0	0	0	0	0	0	1	1	0	1	1	0	1	0	0	0
Majd2020	CBTI	CBT	2020	1	1	1	1	0	1	0	1	1	1	0	0	0	0	0	0	0	1
Majd2020	PE	PE	2020	1	0	0	0	0	0	0	0	0	1	0	0	0	0	0	0	0	1
Manber2008	EsCIT+CBTI	CBT	2008	1	1	1	0	0	1	1	0	1	1	0	0	1	0	1	0	0	0
Manber2008	EsCIT+CTRL	PE	2008	1	1	0	0	0	0	0	0	0	1	0	0	1	0	1	0	0	0
Manber2016	CBTI	CBT	2016	1	0	1	0	0	1	1	0	1	1	0	0	1	0	1	0	0	0
Manber2016	CTRL	PE	2016	1	0	0	0	0	0	0	0	0	1	0	0	1	0	1	0	0	0
Manber2019	CBTI	CBT	2019	1	1	1	0	0	1	1	0	1	1	0	0	1	0	1	0	0	0
Manber2019	CTRL	PE	2019	1	1	0	0	0	0	0	0	0	1	0	0	1	0	1	0	0	0
Mao2018	Intervention	CBT	2018	1	1	1	0	0	1	1	0	1	1	0	1	0	1	1	0	0	0

© 2024 Furukawa Y et al. *JAMA Psychiatry.*

Trial	Arm	Treatment	Year	se	sd	cr	th	cw	sr	sc	pi	re	ns	w	dt	ind	gp	ff	tg	he	ae
Mao2018	Control	PE	2018	1	1	0	0	0	0	0	0	0	1	0	1	0	1	1	0	0	0
Marino2001	CBTI	CBT	2001	1	1	1	0	0	1	1	0	1	1	0	0	0	1	1	0	0	0
Marino2001	WL	WL	2001	0	1	0	0	0	0	0	0	0	0	1	0	0	0	0	0	0	0
Marks2023	CBTI	CBT	2023	1	1	1	0	0	1	1	1	1	1	0	1	0	1	1	0	0	0
Marks2023	ABC	RT	2023	1	1	0	0	0	0	0	0	1	1	0	1	0	1	1	0	0	0
Marks2023	SSG	PE	2023	0	1	0	0	0	0	0	0	0	1	0	1	0	1	1	0	0	0
Martinez2014	CBT-I	CBT	2014	1	1	1	0	0	1	1	0	1	1	0	1	1	0	1	0	0	0
Martinez2014	SH	WL	2014	1	1	0	0	0	0	0	0	0	1	1	1	1	0	1	0	0	0
Matthews2014	CBTI	CBT	2014	1	1	1	0	0	1	1	0	0	1	0	1	1	0	1	0	0	0
Matthews2014	BPT	PE	2014	0	1	0	0	0	0	0	0	0	1	0	1	1	0	1	0	0	0
McCrae2007	MBT	BT	2007	0	1	0	0	0	1	1	0	1	1	0	0	1	0	1	0	0	0
McCrae2007	SHE	PE	2007	1	1	0	0	0	0	0	0	0	1	0	0	1	0	1	0	0	0
McCrae2019	CBT-I	CBT	2019	1	1	1	0	0	1	1	0	1	1	0	1	1	0	1	0	0	0
McCrae2019	WLC	WL	2019	0	1	0	0	0	0	0	0	0	0	1	1	0	0	0	0	0	0
McCrae2020	BBTi	BT	2020	1	1	0	0	0	1	1	0	1	1	0	1	1	0	1	0	0	0
McCrae2020	SMC	PE	2020	0	1	0	0	0	0	0	0	0	1	0	1	1	0	1	0	0	0
McCurry2016	CBT-I	CBT	2016	1	1	1	0	1	1	1	0	0	1	0	0	1	0	0	0	0	0
McCurry2016	MEC	PE	2016	1	1	0	0	0	0	0	0	0	1	0	0	1	0	0	0	0	0
McCurry2021	CBT-I	CBT	2021	1	1	1	1	1	1	1	0	0	1	0	1	1	0	0	0	0	0
McCurry2021	EOC	PE	2021	1	1	0	0	0	0	0	0	0	1	0	1	1	0	0	0	0	0
Means2000	Treated SWI	RT	2000	0	1	0	0	0	0	0	0	1	1	0	0	1	0	1	0	0	0
Means2000	Untreated SWI	WL	2000	0	1	0	0	0	0	0	0	0	0	1	0	0	0	0	0	0	0
Milby1993	Triazolam plus behavioral therapy	BT	1993	0	1	0	0	0	0	1	0	1	1	0	1	1	0	1	0	0	0
Milby1993	Triazolam plus sleep-related information	PE	1993	0	1	0	0	0	0	0	0	0	1	0	1	1	0	1	0	0	0
Miller2021	CBT-I	CBT	2021	1	1	1	0	0	1	1	0	1	1	0	1	1	0	1	0	0	0

© 2024 Furukawa Y et al. *JAMA Psychiatry.*

Trial	Arm	Treatment	Year	se	sd	cr	th	cw	sr	sc	pi	re	ns	w	dt	ind	gp	ff	tg	he	ae
Miller2021	SE	PE	2021	1	1	0	0	0	0	0	0	0	1	0	1	1	0	1	0	0	0
Miller2023	CBT-I	CBT	2023	1	1	1	0	0	1	1	0	1	1	0	1	1	0	1	0	0	0
Miller2023	Sleep hygiene	PE	2023	1	1	0	0	0	0	0	0	0	1	0	1	1	0	1	0	0	0
Mimeault1999	BT	CBT	1999	1	1	1	0	0	1	1	0	0	1	0	1	0	0	0	0	0	0
Mimeault1999	BTPC	CBT	1999	1	1	1	0	0	1	1	0	0	1	0	1	0	0	0	1	1	0
Mimeault1999	WLC	WL	1999	0	1	0	0	0	0	0	0	0	0	1	1	0	0	0	0	0	0
Miro2011	CBT	CBT	2011	1	1	1	0	0	1	1	0	1	1	0	1	0	1	1	0	0	0
Miro2011	SH	WL	2011	1	0	0	0	0	0	0	0	0	1	1	1	0	1	1	0	0	0
Morawetz1989(SOL)	Therapist	BT	1989	0	1	0	0	0	0	1	0	1	1	0	0	0	1	1	0	0	0
Morawetz1989(SOL)	Tape	BT	1989	0	1	0	0	0	0	1	0	1	1	0	0	0	0	0	0	0	0
Morawetz1989(SOL)	WL	WL	1989	0	1	0	0	0	0	0	0	0	0	1	0	0	0	0	0	0	0
Morawetz1989(WASO)	Tape	BT	1989	0	1	0	0	0	0	1	0	1	1	0	0	0	0	0	0	0	0
Morawetz1989(WASO)	WL	WL	1989	0	1	0	0	0	0	0	0	0	0	1	0	0	0	0	0	0	0
Morgan2012	Treatment	CBT	2012	1	1	1	0	0	1	1	0	0	1	0	1	1	0	0	1	1	0
Morgan2012	TAU	PE	2012	1	0	0	0	0	0	0	0	0	1	0	1	1	0	0	0	0	0
Morin1988	SC	BT	1988	0	1	0	0	0	0	1	0	0	1	0	1	0	1	1	0	0	0
Morin1988	IR	PE	1988	0	1	0	0	0	0	0	0	0	1	0	1	0	1	1	0	0	0
Morin1988	WL	WL	1988	0	1	0	0	0	0	0	0	0	0	1	1	0	0	0	0	0	0
Morin1993	CBT	CBT	1993	1	1	1	0	0	1	1	0	0	1	0	0	0	1	1	0	0	0
Morin1993	WLC	WL	1993	0	1	0	0	0	0	0	0	0	0	1	0	0	0	0	0	0	0
Morin1999	Combined	CBT	1999	1	1	1	0	0	1	1	0	0	1	0	1	1	1	1	0	0	0
Morin1999	PCT	PE	1999	0	1	0	0	0	0	0	0	0	1	0	1	1	0	1	0	0	0
NCT00127790-1	CBT-I	CBT	2012	1	0	1	0	0	1	1	0	1	1	0	1	1	0	1	0	0	0
NCT00127790-1	WL	WL	2012	0	0	0	0	0	0	0	0	0	0	1	1	0	0	0	0	0	0
NCT00127790-2	CBT-P	CBT	2012	0	0	1	0	0	0	0	0	1	1	0	1	1	0	1	0	0	0
NCT00127790-2	CBT-P/I	CBT	2012	1	0	1	0	0	1	1	0	1	1	0	1	1	0	1	0	0	0

© 2024 Furukawa Y et al. *JAMA Psychiatry.*

Trial	Arm	Treatment	Year	se	sd	cr	th	cw	sr	sc	pi	re	ns	w	dt	ind	gp	ff	tg	he	ae
NCT01011218-1	BBTI-Armodafil	BT	2018	1	1	0	0	0	1	1	0	0	1	0	0	1	0	1	0	0	0
NCT01011218-1	BP-Armodafil	PE	2018	1	0	0	0	0	0	0	0	0	1	0	0	0	0	0	0	0	0
NCT01011218-2	BBTI-without armodafil	BT	2018	1	1	0	0	0	1	1	0	0	1	0	0	1	0	1	0	0	0
NCT01011218-2	BP-withou armodafil	PE	2018	1	0	0	0	0	0	0	0	0	1	0	0	0	0	0	0	0	0
NCT01987089	CBTI	CBT	2020	1	1	1	0	0	1	1	0	0	1	0	0	1	0	1	0	0	0
NCT01987089	QDT	PE	2020	0	1	0	0	0	0	0	0	0	1	0	0	1	0	1	0	0	0
NCT02613364	CBTI	CBT	2021	1	0	1	0	0	1	1	0	0	1	0	1	1	0	1	0	0	0
NCT02613364	Educational intervention	TAU	2021	0	0	0	0	0	0	0	0	0	1	0	1	1	0	1	0	0	0
NCT03964974	CBTI-CU	CBT	2022	1	1	1	0	0	1	1	0	1	1	0	0	1	0	1	0	0	0
NCT03964974	SHE	PE	2022	1	0	0	0	0	0	0	0	0	1	0	0	1	0	1	0	0	0
NorellClarke2015	CBT-I	CBT	2015	1	1	1	0	1	1	1	0	0	1	0	1	0	1	1	0	0	0
NorellClarke2015	RT	RT	2015	0	1	0	0	0	0	0	0	1	1	0	1	0	1	1	0	0	0
Okajima2020	tailored+standard BBTI	BT	2020	1	1	0	0	0	1	1	0	1	1	0	0	0	0	0	1	0	1
Okajima2020	self-monitoring	WL	2020	0	1	0	0	0	0	0	0	0	0	1	0	0	0	0	0	0	0
Okajima2020	waitig list	WL	2020	0	0	0	0	0	0	0	0	0	0	1	0	0	0	0	0	0	0
Okajima2021	REFRESH	CBT	2022	0	1	1	1	0	1	1	0	1	1	0	0	0	0	0	1	1	0
Okajima2021	Self-Monitoring	PE	2022	0	1	0	0	0	0	0	0	0	1	0	0	0	0	0	1	1	0
Ong2018	MBTI	CBT	2014	0	1	0	1	0	1	1	0	0	1	0	0	0	1	1	0	0	0
Ong2018	MBSR	CT	2014	0	1	0	1	0	0	0	0	0	1	0	0	0	1	1	0	0	0
Ong2018	SMC	WL	2014	0	1	0	0	0	0	0	0	0	0	1	0	0	0	0	0	0	0
Ong2020	Arm B	CBT	2020	1	1	1	0	0	1	1	0	0	1	0	0	1	0	1	0	0	0
Ong2020	Arm C	PE	2020	0	1	0	0	0	0	0	0	0	1	0	0	1	0	1	0	0	0
Oswald2022	Intervention	CBT	2022	1	1	1	0	0	1	1	0	0	1	0	1	0	1	1	0	0	0
Oswald2022	Control	WL	2022	0	0	0	0	0	0	0	0	0	0	1	1	0	0	0	0	0	0
Padron2022	CBTi.p.	CBT	2022	1	1	1	1	0	1	1	0	1	1	0	1	1	0	1	0	0	0
Padron2022	PE	PE	2022	0	1	0	0	0	0	0	0	0	1	0	1	1	0	1	0	0	0

© 2024 Furukawa Y et al. *JAMA Psychiatry.*

Trial	Arm	Treatment	Year	se	sd	cr	th	cw	sr	sc	pi	re	ns	w	dt	ind	gp	ff	tg	he	ae
Palesh2018	BBT-CI	BT	2018	1	0	0	0	0	1	1	0	0	1	0	1	1	0	1	0	0	0
Palesh2018	HEAL	TAU	2018	0	0	0	0	0	0	0	0	0	1	0	1	1	0	1	0	0	0
Palesh2020	BBT-I	BT	2020	1	0	0	0	0	1	1	0	0	1	0	0	1	0	1	0	0	0
Palesh2020	Sleep education	PE	2020	1	0	0	0	0	0	0	0	0	0	0	0	0	0	0	0	0	0
Patel2017	Case	CBT	2017	0	1	0	1	0	1	1	0	1	0	0	0	0	0	0	0	1	1
Patel2017	Control	PE	2017	1	0	0	0	0	0	0	0	0	0	0	0	0	0	0	0	1	0
Perlis2004	M+CBT	CBT	2004	1	1	1	0	0	1	1	0	0	1	0	1	1	0	1	0	0	0
Perlis2004	M+CC	PE	2004	0	1	0	0	0	0	0	0	0	1	0	1	1	0	1	0	0	0
Perrault2022	TX	CBT	2022	1	1	1	0	0	1	1	0	1	1	0	0	0	1	1	0	0	0
Perrault2022	WL	WL	2022	0	1	0	0	0	0	0	0	0	0	1	0	0	0	0	0	0	0
Pigeon2017	CBT-I	CBT	2017	1	1	1	0	0	1	1	0	1	1	0	0	1	0	1	0	0	0
Pigeon2017	SH	PE	2017	1	1	0	0	0	0	0	0	0	1	0	0	1	0	1	0	0	0
Pigeon2019	CBTi	CBT	2019	1	1	1	0	0	1	1	0	0	1	0	1	1	0	1	0	0	0
Pigeon2019	TAU	TAU	2019	0	0	0	0	0	0	0	0	0	1	0	1	1	0	1	0	0	0
Pigeon2022	CBTI+CPT	CBT	2021	1	1	1	0	0	1	1	0	0	1	0	0	1	0	1	0	0	0
Pigeon2022	Control+CPT	PE	2021	0	1	0	0	0	0	0	0	0	1	0	0	1	0	0	0	0	0
Pillai2015	CBTI	CBT	2015	1	1	1	1	0	1	1	1	1	1	0	0	0	0	0	0	0	1
Pillai2015	IC	PE	2015	1	1	0	0	0	0	0	0	0	0	0	0	0	0	0	0	0	0
Prados2020	CBTI-C	CBT	2020	1	1	1	0	0	1	1	0	1	1	0	1	0	1	1	0	0	0
Prados2020	CBTI-P	CBT	2020	0	0	1	0	0	0	0	0	1	1	0	1	0	1	1	0	0	0
Quintiliani2017	PI+	CT	2017	0	1	1	0	0	0	0	0	0	1	0	0	1	0	1	0	0	0
Quintiliani2017	PI-	PE	2017	0	1	0	0	0	0	0	0	0	0	0	0	0	0	0	0	0	0
Redeker2015	CBTI	CBT	2015	1	1	1	0	0	1	1	0	1	1	1	1	0	1	1	0	0	0
Redeker2015	Attention Control	WL	2015	1	1	0	0	0	0	0	0	0	1	1	1	0	1	1	0	0	0
Redeker2022	CBT-I	CBT	2022	1	1	1	0	0	1	1	0	1	1	0	1	1	1	1	0	0	0
Redeker2022	Attention Control	PE	2022	1	1	0	0	0	0	0	0	0	1	0	1	1	1	1	0	0	0

© 2024 Furukawa Y et al. *JAMA Psychiatry.*

Trial	Arm	Treatment	Year	se	sd	cr	th	cw	sr	sc	pi	re	ns	w	dt	ind	gp	ff	tg	he	ae
Ritterband2009	SHUTi	CBT	2009	1	1	1	0	0	1	1	0	0	1	0	1	0	0	0	0	0	1
Ritterband2009	Wait-list	WL	2009	0	1	0	0	0	0	0	0	0	0	1	1	0	0	0	0	0	0
Ritterband2012	Internet	CBT	2012	1	1	1	0	0	1	1	0	0	1	0	1	0	0	0	0	0	1
Ritterband2012	Control	WL	2012	0	1	0	0	0	0	0	0	0	1	1	1	0	0	0	0	0	0
Ritterband2017	SHUTi	CBT	2017	1	1	1	0	0	1	1	0	0	1	0	1	0	0	0	0	0	1
Ritterband2017	Patient education	PE	2017	1	0	0	0	0	0	0	0	0	0	0	1	0	0	0	0	0	0
Robabeh2015	CBT	CBT	2015	1	0	1	0	0	1	1	0	0	1	0	1	1	0	1	0	0	0
Robabeh2015	BPT	PE	2015	0	1	0	0	0	0	0	0	0	1	0	1	1	0	1	0	0	0
Roscoe2015-1	CBTI+placebo	CBT	2015	1	1	1	0	0	1	1	0	0	1	0	0	1	0	1	0	0	0
Roscoe2015-1	placebo	PE	2015	1	0	0	0	0	0	0	0	0	1	0	0	1	0	1	0	0	0
Roscoe2015-2	CBTI+armodafil	CBT	2015	1	1	1	0	0	1	1	0	0	1	0	0	1	0	1	0	0	0
Roscoe2015-2	armodafil	PE	2015	1	0	0	0	0	0	0	0	0	1	0	0	1	0	1	0	0	0
Rosen2000	Education control	PE	2000	0	1	0	0	0	0	0	0	0	1	0	1	0	1	1	0	0	0
Rosen2000	M+G	RT	2000	0	1	0	0	0	0	0	0	1	1	0	1	0	1	1	0	0	0
Rybarczyk2005	CBT	CBT	2005	1	1	1	0	0	1	1	0	1	1	0	1	0	1	1	0	0	0
Rybarczyk2005	SMW	CT	2005	0	1	0	1	0	0	0	0	0	1	1	1	0	1	1	0	0	0
Sadler2018	CBT-I	CBT	2018	1	1	1	0	0	1	1	0	1	1	0	1	0	1	1	0	0	0
Sadler2018	PCG	PE	2018	1	1	0	0	0	0	0	0	0	1	0	1	0	1	1	0	0	0
Sandlund2017	Intervention	CBT	2017	1	1	1	0	1	1	1	1	1	1	0	1	0	1	1	0	0	0
Sandlund2017	Control	WL	2017	0	1	0	0	0	0	0	0	0	0	1	1	0	0	0	0	0	0
Sato2019	ICBT+UC	CBT	2019	1	1	1	0	0	1	1	0	1	1	0	1	0	0	0	0	1	0
Sato2019	UC	WL	2019	1	1	0	0	0	0	0	0	0	1	1	1	0	0	0	0	0	0
Sato2022	ICBT	CBT	2022	0	1	1	0	0	1	1	0	0	1	0	0	0	0	0	0	0	0
Sato2022	WLC	WL	2022	0	0	0	0	0	0	0	0	0	0	1	0	0	0	0	0	0	0
Savard2005	CBT	CBT	2005	1	1	1	0	0	1	1	0	0	1	0	1	0	1	1	0	0	0
Savard2005	WL	WL	2005	0	1	0	0	0	0	0	0	0	0	1	1	0	0	0	0	0	0

© 2024 Furukawa Y et al. *JAMA Psychiatry.*

Trial	Arm	Treatment	Year	se	sd	cr	th	cw	sr	sc	pi	re	ns	w	dt	ind	gp	ff	tg	he	ae
Savard2014	PCBT-I	CBT	2014	1	1	1	0	0	1	1	0	0	1	0	1	1	0	1	0	0	0
Savard2014	VCBT-I	CBT	2014	1	1	1	0	0	1	1	0	0	1	0	1	0	0	0	1	0	0
Savard2014	CTL	PE	2014	0	1	0	0	0	0	0	0	0	0	0	1	0	0	0	0	0	0
Schuffelen2023	dCBT-I	CBT	2023	1	1	1	0	0	1	1	0	1	1	0	1	0	0	0	0	0	0
Schuffelen2023	Control	WL	2023	0	1	0	0	0	0	0	0	0	0	1	1	0	0	0	0	0	0
Sgoifo2017	Exposed	CBT	2017	0	0	0	1	0	0	0	0	1	1	0	1	0	1	1	0	0	0
Sgoifo2017	Control	WL	2017	0	0	0	0	0	0	0	0	0	0	1	1	0	0	0	0	0	0
Sheaves2018	STAC	CBT	2018	1	0	1	0	0	1	1	0	0	1	0	1	1	0	1	0	0	0
Sheaves2018	standard	TAU	2018	0	0	0	0	0	0	0	0	0	1	0	1	1	0	1	0	0	0
Shimamoto2022	SPA	BT	2022	1	1	0	0	0	1	1	0	0	1	0	1	0	0	0	0	0	1
Shimamoto2022	Control	WL	2022	0	0	0	0	0	0	0	0	0	0	1	1	0	0	0	0	0	0
Shin2023	CBT-I	CBT	2023	1	1	1	0	0	1	1	0	1	1	0	0	1	0	1	0	0	0
Shin2023	ACT	CT	2023	1	1	0	1	0	0	0	0	0	1	0	0	1	0	1	0	0	0
Short2021	BBTI	BT	2021	1	1	0	0	0	1	1	0	0	1	0	0	1	0	1	0	0	0
Short2021	Control	WL	2021	0	1	0	0	0	0	0	0	0	0	1	0	0	0	0	0	0	0
Siebmnns2021	Intervention	CBT	2021	1	1	1	0	0	1	1	0	1	1	0	1	0	0	0	1	1	0
Siebmnns2021	Control	WL	2021	1	1	0	0	0	0	0	0	0	1	1	1	0	0	0	0	0	0
Siengsukon2020	CBTI	CBT	2020	1	1	1	1	0	1	1	0	1	1	0	1	1	0	1	0	0	0
Siengsukon2020	BE	PE	2020	1	0	0	0	0	0	0	0	0	1	0	1	1	0	1	0	0	0
Siengsukon2020	AC	TAU	2020	0	0	0	0	0	0	0	0	0	1	0	1	1	0	1	0	0	0
Siengsukon2021	wCBT-I	CBT	2021	1	1	1	1	0	1	1	0	1	1	0	1	0	0	0	0	0	1
Siengsukon2021	wCBT-I+calls	CBT	2021	1	1	1	1	0	1	1	0	1	1	0	1	0	0	0	1	1	1
Simeit2004	PMR	CBT	2004	1	0	1	0	0	0	1	0	1	1	0	1	0	1	1	0	0	0
Simeit2004	C-group	RT	2004	0	0	0	0	0	0	0	0	1	1	0	1	0	1	1	0	0	0
Smith2015	CBT-I	CBT	2015	1	1	1	0	0	1	1	0	0	1	0	0	1	0	1	0	0	0
Smith2015	BD	PE	2015	0	1	0	0	0	0	0	0	0	1	0	0	1	0	1	0	0	0

© 2024 Furukawa Y et al. *JAMA Psychiatry.*

Trial	Arm	Treatment	Year	se	sd	cr	th	cw	sr	sc	pi	re	ns	w	dt	ind	gp	ff	tg	he	ae
Smitherman2016	CBTi	BT	2016	1	1	0	0	0	1	1	0	0	1	0	1	1	0	1	0	0	0
Smitherman2016	Control	PE	2016	0	1	0	0	0	0	0	0	0	1	0	1	1	0	1	0	0	0
Soeffing2008	CBTI	BT	2008	1	1	0	0	0	0	1	0	1	1	0	1	1	0	1	0	0	0
Soeffing2008	Plcebo	PE	2008	0	1	0	0	0	0	0	0	0	1	0	1	1	0	1	0	0	0
Song2020	CBTI	CBT	2020	1	1	1	0	0	1	1	0	1	1	0	1	1	0	1	0	0	0
Song2020	NonCBTI	PE	2020	1	1	0	0	0	0	0	0	0	1	0	1	1	0	1	0	0	0
Speed2022	gCBT-I	CBT	2022	1	1	1	0	0	1	1	0	0	1	0	0	0	1	1	0	0	0
Speed2022	SOC	PE	2022	0	1	0	0	0	0	0	0	0	1	0	0	1	1	1	0	0	0
Strom2004	Treatment	CBT	2004	1	1	1	0	0	1	1	0	1	1	0	1	0	0	0	0	0	1
Strom2004	Waitlist	WL	2004	0	1	0	0	0	0	0	0	0	0	1	1	0	0	0	0	0	0
Store2022	Intervention	RT	2022	0	1	0	0	0	0	0	0	1	1	0	1	0	0	0	0	0	0
Store2022	Waitlist	WL	2022	0	1	0	0	0	0	0	0	0	0	1	1	0	0	0	0	0	0
Sunnheld2020	BT	BT	2020	1	1	0	0	0	1	1	0	0	1	0	1	0	0	0	1	1	1
Sunnheld2020	CT	CT	2020	0	1	1	0	0	0	0	0	0	1	0	1	0	0	0	1	1	1
Sunnheld2020	WL	WL	2020	0	1	0	0	0	0	0	0	0	0	1	1	0	0	0	0	0	1
Sveen2021	Intervention	CBT	2021	1	1	1	0	0	1	1	0	1	1	0	1	0	0	0	1	1	0
Sveen2021	Control	CT	2021	1	0	0	1	0	0	0	0	0	1	0	1	0	0	0	0	0	0
Sweetman2019	CBTi	CBT	2019	1	1	1	0	0	1	0	0	0	1	0	1	1	1	1	0	0	0
Sweetman2019	Control	WL	2019	0	1	0	0	0	0	0	0	0	0	1	1	0	0	0	0	0	0
Talbot2014	CBT-I	CBT	2014	1	1	1	0	0	1	1	0	0	1	0	0	1	0	1	0	0	0
Talbot2014	Waitlist	WL	2014	0	1	0	0	0	0	0	0	0	0	1	0	0	0	0	0	1	0
Taylor2014a	CBTI	CBT	2014	1	1	1	0	0	1	1	0	1	1	0	0	1	0	1	0	0	0
Taylor2014a	WLC	WL	2014	0	1	0	0	0	0	0	0	0	0	1	0	0	0	0	0	0	0
Taylor2017	Internet CBTi	CBT	2017	1	1	1	0	0	1	1	0	1	1	0	1	0	0	0	0	0	1
Taylor2017	In-person CBTi	CBT	2017	1	1	1	0	0	1	1	0	1	1	0	1	1	0	1	0	0	0
Taylor2017	Control	PE	2017	0	1	0	0	0	0	0	0	0	0	0	1	1	0	1	0	0	0

© 2024 Furukawa Y et al. *JAMA Psychiatry.*

Trial	Arm	Treatment	Year	se	sd	cr	th	cw	sr	sc	pi	re	ns	w	dt	ind	gp	ff	tg	he	ae
Taylor2018	CBTI	CBT	2018	1	1	1	0	0	1	1	0	1	1	0	1	1	0	1	0	0	0
Taylor2018	Control	PE	2018	0	1	0	0	0	0	0	0	0	0	0	1	1	0	1	0	0	0
Ustinov2014	Treatment	BT	2013	1	1	0	0	0	0	1	0	1	1	0	1	0	1	1	0	0	0
Ustinov2014	Waitlist	WL	2013	0	1	0	0	0	0	0	0	0	0	1	1	0	0	0	0	0	0
VanDerZweerde2019	i-Sleep	CBT	2019	1	1	1	0	0	1	1	0	1	1	0	1	0	0	0	1	1	0
VanDerZweerde2019	Sdiary	WL	2019	0	1	0	0	0	0	0	0	0	0	1	1	0	0	0	0	0	0
VanDerZweerde2020	i-Sleep	CBT	2020	1	1	1	0	0	1	1	0	1	1	0	1	0	0	0	1	1	0
VanDerZweerde2020	CAU	WL	2020	0	1	0	0	0	0	0	0	0	0	1	1	0	0	0	0	0	0
VanStraten2014	Intervention	CBT	2014	1	1	1	0	0	1	1	0	1	1	0	1	0	0	0	1	1	0
VanStraten2014	Control	WL	2014	0	1	0	0	0	0	0	0	0	0	1	1	0	0	0	0	0	0
Vedaa2020	dCBT-I	CBT	2020	1	1	1	0	0	1	1	0	0	1	0	1	0	0	0	0	0	1
Vedaa2020	patient education	PE	2020	1	1	0	0	0	0	0	0	0	1	0	1	0	0	0	0	0	0
Verma2022	CBTI	CBT	2022	1	1	1	0	0	0	1	0	1	1	0	0	0	0	0	1	1	0
Verma2022	TAU	WL	2022	0	1	0	0	0	0	0	0	0	0	1	0	0	0	0	0	0	0
Vincent2009	Treatment	CBT	2009	1	1	1	0	0	1	1	0	1	1	0	1	0	0	0	0	0	0
Vincent2009	Control	WL	2009	0	1	0	0	0	0	0	0	0	0	1	1	0	0	0	0	0	0
Vitiello2013	CBT-PI	BT	2013	1	1	0	0	0	1	1	0	1	1	0	1	0	1	1	0	0	0
Vitiello2013	CBT-P	RT	2013	0	0	0	0	0	0	0	0	1	1	0	1	0	1	1	0	0	0
Wagley2013	Treatment	CBT	2013	1	0	1	0	0	1	1	0	0	1	0	1	1	0	1	0	0	0
Wagley2013	Waitlist	WL	2013	0	0	0	0	0	0	0	0	0	0	1	1	0	0	0	0	0	0
Wang2016	BBTI	BT	2016	1	1	0	0	0	1	1	0	0	1	0	1	1	0	1	0	0	0
Wang2016	Sleep hygiene	PE	2016	1	1	0	0	0	0	0	0	0	1	0	1	1	0	1	0	0	0
Watanabe2011	BBTI+TAU	BT	2011	1	1	0	0	0	1	1	0	0	1	0	1	1	0	1	0	0	0
Watanabe2011	TAU	PE	2011	1	0	0	0	0	0	0	0	0	1	0	1	1	0	1	0	0	0
Watanabe2023	CBT-I App	CBT	2023	1	1	1	0	0	1	1	0	1	1	0	0	0	0	0	0	0	0
Watanabe2023	Sham App	PLB	2023	0	0	0	0	0	0	0	0	0	1	0	0	0	0	0	0	0	0

© 2024 Furukawa Y et al. *JAMA Psychiatry.*

Trial	Arm	Treatment	Year	se	sd	cr	th	cw	sr	sc	pi	re	ns	w	dt	ind	gp	ff	tg	he	ae
Wilkund2022	ICBT-i	BT	2022	1	1	0	0	0	1	1	0	0	1	0	1	0	0	0	1	1	0
Wilkund2022	AR	RT	2022	0	1	0	0	0	0	0	0	1	1	0	1	0	0	0	1	1	0
Woldeamanuel2021	CCT-CI	BT	2020	1	0	0	0	0	1	1	0	0	1	0	1	1	0	1	0	0	0
Woldeamanuel2021	HEAL	TAU	2020	0	0	0	0	0	0	0	0	0	1	0	1	1	0	1	0	0	0
Wong2016	CT	CBT	2016	1	1	1	0	1	1	0	0	0	1	0	0	1	0	1	0	0	0
Wong2016	MBT	CBT	2016	1	1	1	1	1	1	0	0	0	1	0	0	1	0	1	0	0	0
Wong2017	PEEC	BT	2017	1	1	0	0	0	0	1	0	0	1	0	1	0	1	1	0	0	0
Wong2017	MBCT-I	CBT	2017	1	1	1	1	0	1	1	0	0	1	0	1	0	1	1	0	0	0
Wong2021	CBTI Workshop	CBT	2021	1	1	1	0	0	1	1	0	1	1	0	1	0	1	1	0	0	0
Wong2021	Selfhelp CBTI	CBT	2021	1	1	1	0	0	1	1	0	1	1	0	1	0	0	0	0	0	0
Wong2021	SHE	PE	2021	1	1	0	0	0	0	0	0	0	1	0	1	0	1	1	0	0	0
Wu2006	Combined	CBT	2006	1	1	1	0	0	1	1	0	0	1	0	1	1	0	1	0	0	0
Wu2006	PCT	PE	2006	0	1	0	0	0	0	0	0	0	1	0	1	1	0	1	0	0	0
Xing2020	EA/CBT	CBT	2020	1	1	1	1	0	1	1	0	1	1	0	1	1	1	1	0	0	0
Xing2020	EA	TAU	2020	0	0	0	0	0	0	0	0	0	1	0	1	1	0	1	0	0	0
Yamamoto2016	Intervention	BT	2016	1	0	0	0	0	1	1	0	1	1	0	0	1	1	1	0	0	0
Yamamoto2016	Control	WL	2016	0	0	0	0	0	0	0	0	0	0	1	0	0	0	0	0	0	0
Yang2022	CBT-I	CBT	2022	1	1	1	0	0	1	1	0	1	1	0	0	0	1	1	0	0	0
Yang2022	Control	PE	2022	0	1	0	0	0	0	0	0	0	0	0	0	0	0	0	0	0	0
Yang2023	Self-help	CBT	2023	1	1	1	1	0	1	0	0	1	1	0	1	0	0	0	0	0	0
Yang2023	Feedback	CBT	2023	1	1	1	1	0	1	0	0	1	1	0	1	0	0	0	1	1	0
Yang2023	Waitlist	WL	2023	0	0	0	0	0	0	0	0	0	0	1	1	0	0	0	0	0	0
Zachariades2012	Intervention	CBT	2012	1	1	1	0	0	1	1	0	1	1	0	1	0	0	0	1	1	0
Zachariades2012	Control	WL	2012	0	1	0	0	0	0	0	0	0	0	1	1	0	0	0	0	0	0
Zachariae2018	Intervention	CBT	2018	1	1	1	0	0	1	1	0	0	1	0	1	0	0	0	0	0	1
Zachariae2018	Control	WL	2018	0	1	0	0	0	0	0	0	0	0	1	1	0	0	0	0	0	0

© 2024 Furukawa Y et al. *JAMA Psychiatry.*

Trial	Arm	Treatment	Year	se	sd	cr	th	cw	sr	sc	pi	re	ns	w	dt	ind	gp	ff	tg	he	ae
Zakiei2021	Intervention	CT	2021	0	1	0	1	0	0	0	0	0	1	0	0	0	1	1	0	0	0
Zakiei2021	Control	PLB	2021	0	0	0	0	0	0	0	0	0	1	0	0	0	1	1	0	0	0
Zhang2015	MBSR	CT	2015	0	0	0	1	0	0	0	0	0	1	0	1	0	1	1	0	0	0
Zhang2015	Wait-list	WL	2015	0	0	0	0	0	0	0	0	0	0	1	1	0	0	0	0	0	0
Zhang2019	MBSR	CT	2019	0	1	0	1	0	0	0	0	0	1	0	1	0	1	1	0	0	0
Zhang2019	UC	PE	2019	0	1	0	0	0	0	0	0	0	0	0	1	0	0	0	0	0	0
Zhang2023	DCBT-I	CBT	2023	1	1	1	0	0	1	1	0	1	1	0	1	0	0	0	0	0	0
Zhang2023	Sleep education	BT	2023	1	1	0	0	0	0	1	0	0	1	0	1	0	0	0	0	0	0
Zhao2020	MBCT-I	CT	2020	1	0	1	1	0	0	0	0	0	1	0	1	0	1	1	0	0	0
Zhao2020	Waitlist	WL	2020	0	0	0	0	0	0	0	0	0	0	1	1	0	0	0	0	0	0
Zhou2022a	SHUTi-BWHS+SHUTi-normal	CBT	2022	1	1	1	0	0	1	1	0	0	1	0	1	0	0	0	0	0	1
Zhou2022a	Control	PE	2022	1	1	0	0	0	0	0	0	0	1	0	1	0	0	0	0	0	0
Zhou2022b	eCBT-I	CBT	2022	1	1	1	0	0	1	1	0	1	1	0	0	1	0	0	1	1	0
Zhou2022b	Control	NT	2022	0	0	0	0	0	0	0	0	0	0	0	0	0	0	0	0	0	0

© 2024 Furukawa Y et al. *JAMA Psychiatry.*

3.3. Observed combinations of components and delivery formats

	se	sd	cr	th	cw	sr	sc	pi	re	ns	w	ind	gp	ff	tg	he	ae
Sleep hygiene education (se)	324	283	194	42	20	231	229	18	145	320	15	167	73	202	34	39	36
Sleep diary (sd)		433	187	47	21	226	231	19	154	359	76	193	80	236	39	48	38
Cognitive restructuring (cr)			203	35	20	193	188	18	133	203	2	89	51	116	32	36	28
Third wave components (th)				58	7	37	35	9	37	58	3	16	20	30	7	12	13
Constructive worry (cw)					21	21	19	4	14	21	1	12	4	12	3	2	1
Sleep restriction (sr)						244	228	17	139	244	2	124	58	152	34	37	32
Stimulus control (sc)							249	17	146	249	2	124	60	157	32	36	32
Paradoxical intention (pi)								20	18	18	1	5	3	6	1	6	7
Relaxation (re)									174	174	3	69	49	100	27	33	21
Non-specific treatment effect (ns)										423	22	224	99	283	39	46	39
Waiting component (w)											102	11	5	15	0	0	3
Individual (ind)												228	20	209	4	4	0
Group (gp)													100	97	1	1	0
Face-to-face (ff)														287	2	2	0
Online therapeutic guidance (tg)															39	34	5
Human encouragement (he)																49	9
Automatic encouragement (ae)																	41

The diagonal cells indicate the total number of arms that contained the component in question. The cells in the upper-right corner shows the number of arms that contained the combinations of components shown in the column and the raw.

© 2024 Furukawa Y et al. *JAMA Psychiatry.*

eTable 4. League table for treatment-level network meta-analysis

CBT	1.76 (1.00 to 3.10)	2.36 (1.15 to 4.87)	2.96 (1.68 to 5.23)	3.74 (1.96 to 7.14)	3.17 (1.44 to 6.97)	3.54 (2.96 to 4.22)	6.30 (2.14 to 18.54)	6.01 (4.83 to 7.49)
1.52 (1.18 to 1.95)	BT	1.31 (0.57 to 3.03)	1.58 (0.60 to 4.16)	2.58 (1.18 to 5.64)	.	3.49 (2.37 to 5.15)	.	3.01 (2.09 to 4.33)
1.52 (0.98 to 2.36)	1.00 (0.63 to 1.60)	CT	.	1.20 (0.18 to 7.94)	8.80 (0.85 to 90.84)	3.56 (1.18 to 10.73)	3.32 (1.01 to 10.88)	8.31 (3.36 to 20.53)
2.70 (1.65 to 4.40)	1.78 (1.06 to 2.99)	1.77 (0.93 to 3.40)	TAU	.	.	4.50 (0.51 to 39.94)	.	.
2.97 (1.97 to 4.46)	1.95 (1.27 to 3.01)	1.95 (1.10 to 3.46)	1.10 (0.59 to 2.06)	RT	.	1.92 (0.84 to 4.37)	.	2.21 (0.87 to 5.60)
3.67 (1.74 to 7.75)	2.42 (1.10 to 5.31)	2.42 (1.04 to 5.62)	1.36 (0.56 to 3.33)	1.24 (0.53 to 2.90)	NT	.	.	.
3.79 (3.21 to 4.47)	2.50 (1.93 to 3.24)	2.49 (1.59 to 3.92)	1.40 (0.84 to 2.34)	1.28 (0.84 to 1.95)	1.03 (0.48 to 2.22)	PE	.	4.30 (1.01 to 18.27)
5.70 (2.51 to 12.97)	3.76 (1.61 to 8.79)	3.75 (1.63 to 8.63)	2.11 (0.81 to 5.50)	1.92 (0.77 to 4.78)	1.55 (0.52 to 4.68)	1.51 (0.65 to 3.47)	PLB	.
5.74 (4.72 to 6.98)	3.78 (2.92 to 4.91)	3.78 (2.39 to 5.96)	2.13 (1.27 to 3.58)	1.94 (1.26 to 2.96)	1.56 (0.72 to 3.38)	1.51 (1.19 to 1.93)	1.01 (0.43 to 2.33)	WL

Odds ratio (95%CI) of remission at post-treatment of the upper-left-defined treatment against down-right-defined treatment.

BT = behavioral therapy; CBT = cognitive behavioral therapy; CT = cognitive therapy; NT = no treatment; PE = psychoeducation; PLB = attention placebo; RT = relaxation therapy; TAU = treatment as usual; WL = waiting list.

© 2024 Furukawa Y et al. *JAMA Psychiatry.*

eTable 5. CINeMA

Within-study bias: We used the majority RoB2 assessment.

Reporting bias: Low.

Indirectness: No concerns.

Imprecision, heterogeneity, incoherence: We used OR=1.5 as the clinically meaningful threshold.

Comparison	N	Within-study bias	Reporting bias	Indirectness	Imprecision	Heterogeneity	Incoherence	Confidence rating	Reason(s) for downgrading
BT:CBT	8	Some concerns	Low risk	No concerns	No concerns	Major concerns	No concerns	Low	["Within-study bias", "Heterogeneity"]
BT:CT	2	Some concerns	Low risk	No concerns	Major concerns	No concerns	No concerns	Low	["Within-study bias", "Imprecision"]
BT:NT	1	Some concerns	Low risk	No concerns	Some concerns	Some concerns	No concerns	Moderate	["Within-study bias", "Imprecision", "Heterogeneity"]
BT:PE	25	Some concerns	Low risk	No concerns	No concerns	Some concerns	Some concerns	Moderate	["Within-study bias", "Heterogeneity", "Incoherence"]
BT:RT	4	Some concerns	Low risk	No concerns	No concerns	Some concerns	No concerns	Moderate	["Within-study bias", "Heterogeneity"]
BT:TAU	2	Some concerns	Low risk	No concerns	No concerns	Major concerns	No concerns	Low	["Within-study bias", "Heterogeneity"]
BT:WL	22	Some concerns	Low risk	No concerns	No concerns	No concerns	No concerns	Moderate	["Within-study bias"]
CBT:CT	6	Some concerns	Low risk	No concerns	Some concerns	Some concerns	No concerns	Moderate	["Within-study bias", "Imprecision", "Heterogeneity"]
CBT:NT	2	Some concerns	Low risk	No concerns	No concerns	Some concerns	No concerns	Moderate	["Within-study bias", "Heterogeneity"]
CBT:PE	76	Some concerns	Low risk	No concerns	No concerns	No concerns	No concerns	Moderate	["Within-study bias"]
CBT:PLB	3	Some concerns	Low risk	No concerns	No concerns	No concerns	No concerns	Moderate	["Within-study bias"]
CBT:RT	5	Some concerns	Low risk	No concerns	No concerns	No concerns	No concerns	Moderate	["Within-study bias"]
CBT:TAU	6	Some concerns	Low risk	No concerns	No concerns	Some concerns	No concerns	Moderate	["Within-study bias"," Heterogeneity"]
CBT:WL	68	Some concerns	Low risk	No concerns	No concerns	No concerns	No concerns	Moderate	["Within-study bias"]
CT:NT	1	Some concerns	Low risk	No concerns	Some concerns	Some concerns	No concerns	Moderate	["Within-study bias", "Imprecision", "Heterogeneity"]

© 2024 Furukawa Y et al. *JAMA Psychiatry.*

CT:PE	3	Some concerns	Low risk	No concerns	No concerns	Some concerns	No concerns	Moderate	["Within-study bias", "Heterogeneity"]
CT:PLB	2	Some concerns	Low risk	No concerns	No concerns	No concerns	No concerns	Moderate	["Within-study bias"]
CT:RT	1	Some concerns	Low risk	No concerns	No concerns	Major concerns	No concerns	Low	["Within-study bias", "Heterogeneity"]
CT:WL	5	Some concerns	Low risk	No concerns	No concerns	No concerns	No concerns	Moderate	["Within-study bias"]
PE:RT	5	Some concerns	Low risk	No concerns	Some concerns	Some concerns	No concerns	Moderate	["Within-study bias"," Imprecision", "Heterogeneity"]
PE:TAU	1	Some concerns	Low risk	No concerns	Some concerns	Some concerns	No concerns	Moderate	["Within-study bias"," Imprecision"," Heterogeneity"]
PE:WL	3	Some concerns	Low risk	No concerns	No concerns	Major concerns	No concerns	Low	["Within-study bias"," Heterogeneity"]
RT:WL	5	Some concerns	Low risk	No concerns	No concerns	Some concerns	No concerns	Moderate	["Within-study bias", "Heterogeneity"]
BT:PLB	0	Some concerns	Low risk	No concerns	No concerns	No concerns	No concerns	Very low	No direct evidence
CT:TAU	0	Some concerns	Low risk	No concerns	Some concerns	Some concerns	No concerns	Very low	No direct evidence
NT:PE	0	Some concerns	Low risk	No concerns	Major concerns	No concerns	No concerns	Very low	No direct evidence
NT:PLB	0	Some concerns	Low risk	No concerns	Major concerns	No concerns	No concerns	Very low	No direct evidence
NT:RT	0	Some concerns	Low risk	No concerns	Major concerns	No concerns	No concerns	Very low	No direct evidence
NT:TAU	0	Some concerns	Low risk	No concerns	Major concerns	No concerns	No concerns	Very low	No direct evidence
NT:WL	0	Some concerns	Low risk	No concerns	Some concerns	Some concerns	No concerns	Very low	No direct evidence
PE:PLB	0	Some concerns	Low risk	No concerns	Major concerns	No concerns	No concerns	Very low	No direct evidence
PLB:RT	0	Some concerns	Low risk	No concerns	Some concerns	Some concerns	No concerns	Very low	No direct evidence
PLB:TAU	0	Some concerns	Low risk	No concerns	Some concerns	Some concerns	No concerns	Very low	No direct evidence
PLB:WL	0	Some concerns	Low risk	No concerns	Major concerns	No concerns	No concerns	Very low	No direct evidence
RT:TAU	0	Some concerns	Low risk	No concerns	Major concerns	No concerns	No concerns	Very low	No direct evidence
TAU:WL	0	Some concerns	Low risk	No concerns	No concerns	Some concerns	No concerns	Very low	No direct evidence

© 2024 Furukawa Y et al. *JAMA Psychiatry.*

eTable 6. PRISMA-NMA

Section/Topic	Item #	Checklist Item	Reported on Page #
**TITLE**			
Title	1	Identify the report as a systematic review *incorporating a network meta-analysis (or related form of meta-analysis).*	P1
**ABSTRACT**			
Structured summary	2	Provide a structured summary including, as applicable:<br>**Background:** main objectives<br>**Methods:** data sources; study eligibility criteria, participants, and interventions; study appraisal; and *synthesis methods, such as network meta-analysis.*<br>**Results:** number of studies and participants identified; summary estimates with corresponding confidence/credible intervals; *treatment rankings may also be discussed. Authors may choose to summarize pairwise comparisons against a chosen treatment included in their analyses for brevity.*<br>**Discussion/Conclusions:** limitations; conclusions and implications of findings.<br>**Other:** primary source of funding; systematic review registration number with registry name.	P5
**INTRODUCTION**			
Rationale	3	Describe the rationale for the review in the context of what is already known*, including mention of why a network meta-analysis has been conducted.*_	P6
Objectives	4	Provide an explicit statement of questions being addressed, with reference to participants, interventions, comparisons, outcomes, and study design (PICOS).	P6-7
**METHODS**			

© 2024 Furukawa Y et al. *JAMA Psychiatry.*

Protocol and registration	5	Indicate whether a review protocol exists and if and where it can be accessed (e.g., Web address); and, if available, provide registration information, including registration number.	P6, appendix
Eligibility criteria	6	Specify study characteristics (e.g., PICOS, length of follow-up) and report characteristics (e.g., years considered, language, publication status) used as criteria for eligibility, giving rationale. *Clearly describe eligible treatments included in the treatment network, and note whether any have been clustered or merged into the same node (with justification).*_	P6-7
Information sources	7	Describe all information sources (e.g., databases with dates of coverage, contact with study authors to identify additional studies) in the search and date last searched.	P7
Search	8	Present full electronic search strategy for at least one database, including any limits used, such that it could be repeated.	Appendix
Study selection	9	State the process for selecting studies (i.e., screening, eligibility, included in systematic review, and, if applicable, included in the meta-analysis).	P8, Appendix
Data collection process	10	Describe method of data extraction from reports (e.g., piloted forms, independently, in duplicate) and any processes for obtaining and confirming data from investigators.	P7
Data items	11	List and define all variables for which data were sought (e.g., PICOS, funding sources) and any assumptions and simplifications made.	Appendix
**Geometry of the network**	**S1**	Describe methods used to explore the geometry of the treatment network under study and potential biases related to it. This should include how the evidence base has been graphically summarized for presentation, and what characteristics were compiled and used to describe the evidence base to readers.	Figure1, Appendix
Risk of bias within individual studies	12	Describe methods used for assessing risk of bias of individual studies (including specification of whether this was done at the study or outcome level), and how this information is to be used in any data synthesis.	P8-9, Appendix
Summary measures	13	State the principal summary measures (e.g., risk ratio, difference in means). *Also describe the use of additional summary measures assessed, such as treatment rankings and surface under the cumulative ranking curve (SUCRA) values, as well as modified approaches used to present summary findings from meta-analyses.*	P9

© 2024 Furukawa Y et al. *JAMA Psychiatry.*

Planned methods of analysis	14	Describe the methods of handling data and combining results of studies for each network meta-analysis. This should include, but not be limited to:<br>• *Handling of multi-arm trials;*<br>• *Selection of variance structure;*<br>• *Selection of prior distributions in Bayesian analyses; and*<br>• *Assessment of model fit.*	P9
**Assessment of Inconsistency**	**S2**	Describe the statistical methods used to evaluate the agreement of direct and indirect evidence in the treatment network(s) studied. Describe efforts taken to address its presence when found.	P9
Risk of bias across studies	15	Specify any assessment of risk of bias that may affect the cumulative evidence (e.g., publication bias, selective reporting within studies).	P8
Additional analyses	16	Describe methods of additional analyses if done, indicating which were pre-specified. This may include, but not be limited to, the following:<br>• Sensitivity or subgroup analyses;<br>• Meta-regression analyses;<br>• *Alternative formulations of the treatment network; and*<br>• *Use of alternative prior distributions for Bayesian analyses (if applicable).*_	P9
**RESULTS†**			
Study selection	17	Give numbers of studies screened, assessed for eligibility, and included in the review, with reasons for exclusions at each stage, ideally with a flow diagram.	Appendix
**Presentation of network structure**	**S3**	Provide a network graph of the included studies to enable visualization of the geometry of the treatment network.	Figure1

© 2024 Furukawa Y et al. *JAMA Psychiatry.*

**Summary of network geometry**	**S4**	Provide a brief overview of characteristics of the treatment network. This may include commentary on the abundance of trials and randomized patients for the different interventions and pairwise comparisons in the network, gaps of evidence in the treatment network, and potential biases reflected by the network structure.	P9-10, Figure1
Study characteristics	18	For each study, present characteristics for which data were extracted (e.g., study size, PICOS, follow-up period) and provide the citations.	Appendix
Risk of bias within studies	19	Present data on risk of bias of each study and, if available, any outcome level assessment.	Appendix
Results of individual studies	20	For all outcomes considered (benefits or harms), present, for each study: 1) simple summary data for each intervention group, and 2) effect estimates and confidence intervals. *Modified approaches may be needed to deal with information from larger networks.*	***Appendix***
Synthesis of results	21	Present results of each meta-analysis done, including confidence/credible intervals. *In larger networks, authors may focus on comparisons versus a particular comparator (e.g. placebo or standard care), with full findings presented in an appendix. League tables and forest plots may be considered to summarize pairwise comparisons.* If additional summary measures were explored (such as treatment rankings), these should also be presented.	Figure2, Figure3, Appendix
**Exploration for inconsistency**	**S5**	Describe results from investigations of inconsistency. This may include such information as measures of model fit to compare consistency and inconsistency models, *P* values from statistical tests, or summary of inconsistency estimates from different parts of the treatment network.	Appendix
Risk of bias across studies	22	Present results of any assessment of risk of bias across studies for the evidence base being studied.	Appendix CINeMA
Results of additional analyses	23	Give results of additional analyses, if done (e.g., sensitivity or subgroup analyses, meta-regression analyses*, alternative network geometries studied, alternative choice of prior distributions for Bayesian analyses,* and so forth).	Appendix
**DISCUSSION**			
Summary of evidence	24	Summarize the main findings, including the strength of evidence for each main outcome; consider their relevance to key groups (e.g., healthcare providers, users, and policy-makers).	P11-13

© 2024 Furukawa Y et al. *JAMA Psychiatry.*

Limitations	25	Discuss limitations at study and outcome level (e.g., risk of bias), and at review level (e.g., incomplete retrieval of identified research, reporting bias). *Comment on the validity of the assumptions, such as transitivity and consistency. Comment on any concerns regarding network geometry (e.g., avoidance of certain comparisons).*	P12-13
Conclusions	26	Provide a general interpretation of the results in the context of other evidence, and implications for future research.	P13
**FUNDING**			
Funding	27	Describe sources of funding for the systematic review and other support (e.g., supply of data); role of funders for the systematic review. This should also include information regarding whether funding has been received from manufacturers of treatments in the network and/or whether some of the authors are content experts with professional conflicts of interest that could affect use of treatments in the network.	***Y***

© 2024 Furukawa Y et al. *JAMA Psychiatry.*

eFigure 1. Screening process and results

1.1. Flow diagram 2022 initial review

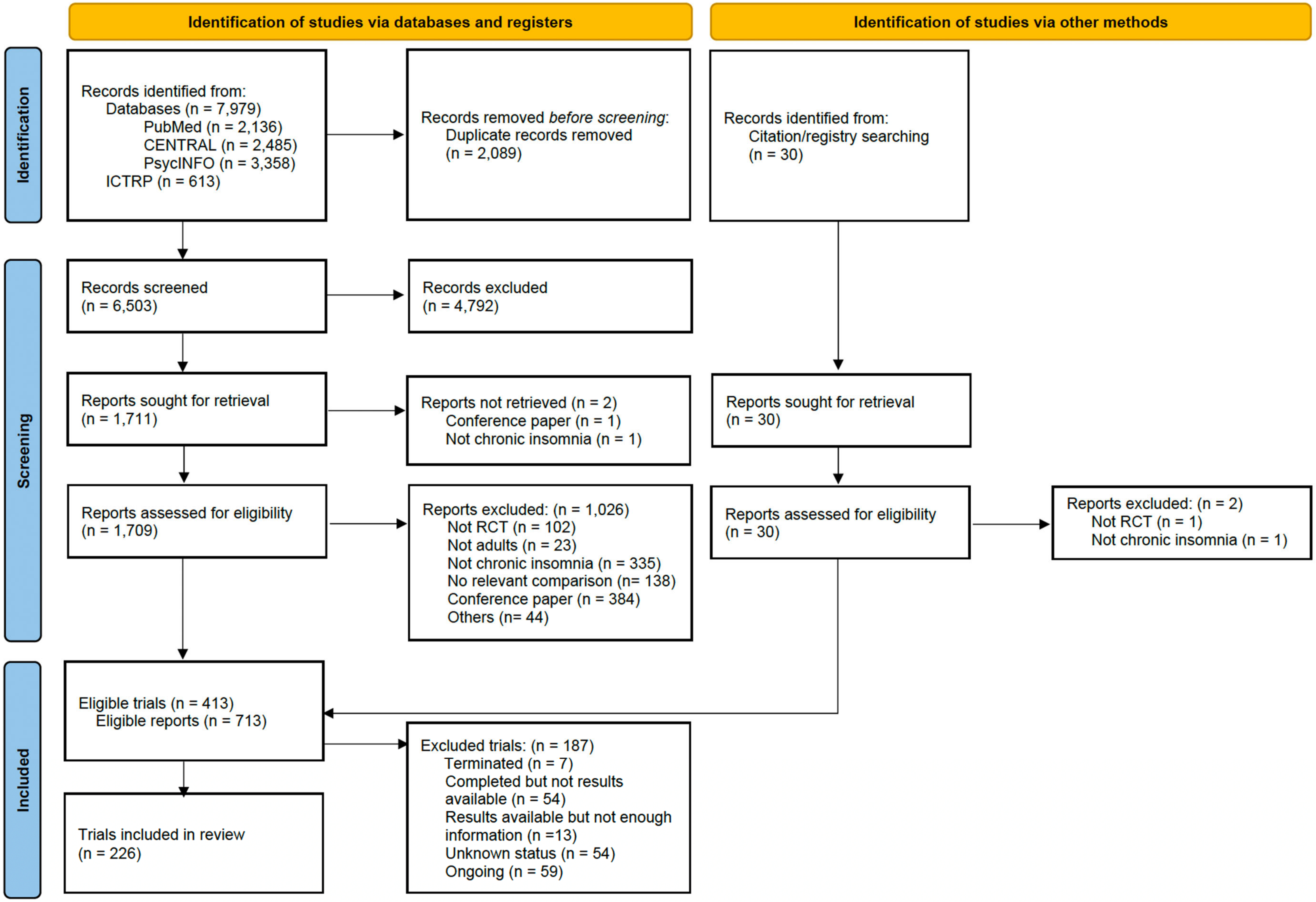

©

1.2. Flow diagram 2023 update

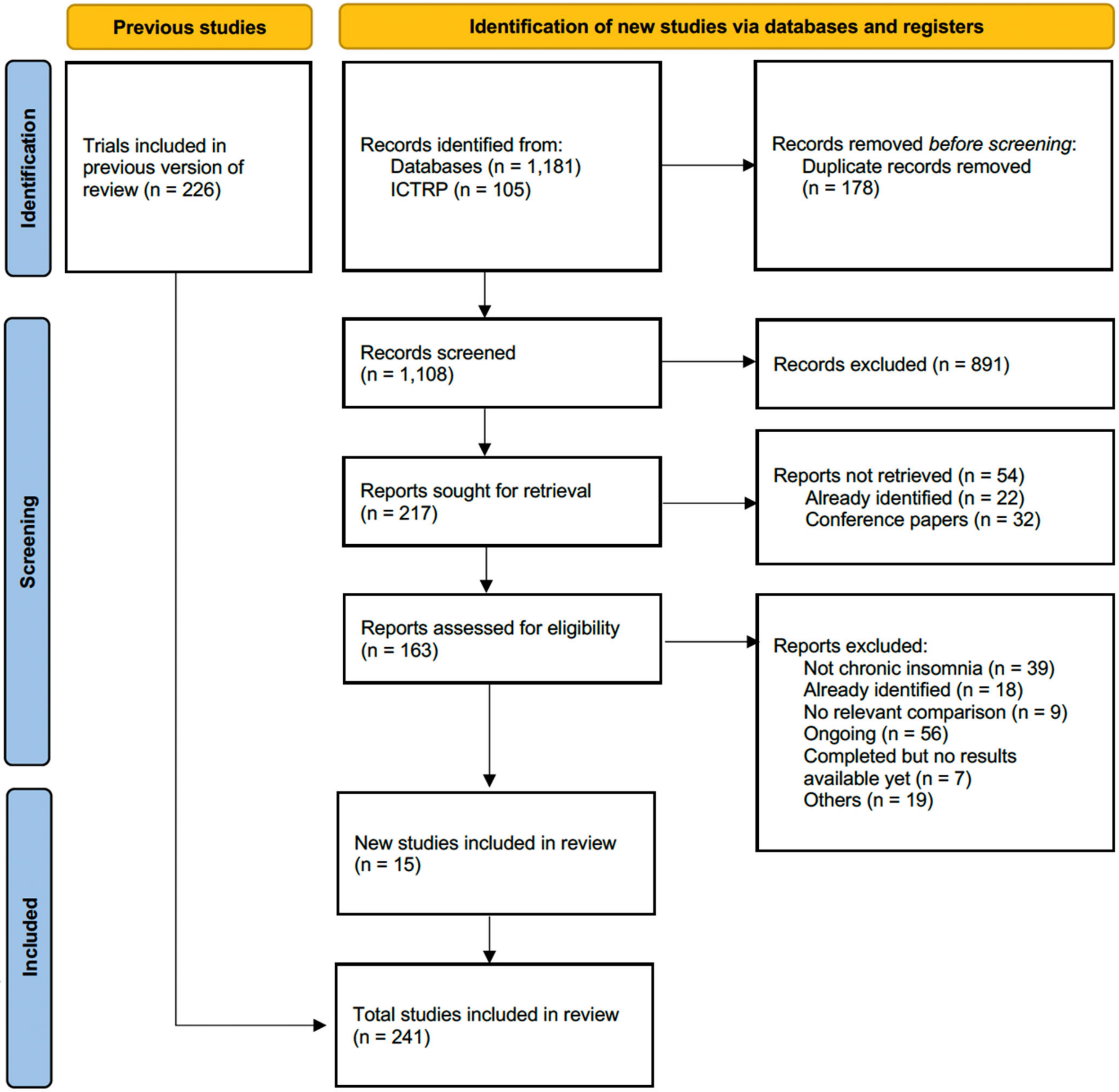

© 2024 Furukawa

eFigure 2. Assessment of transitivity

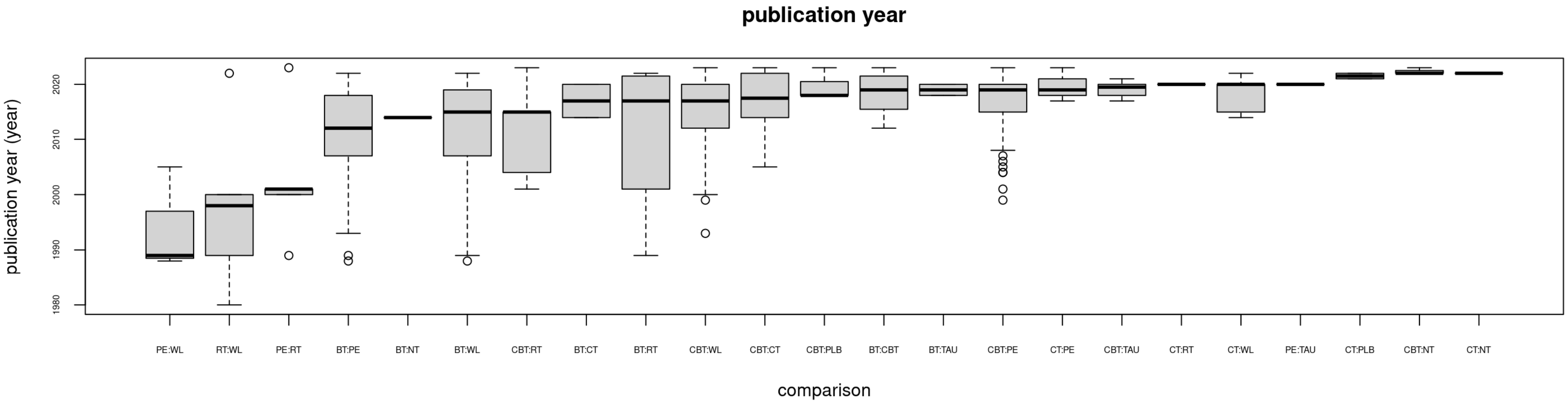

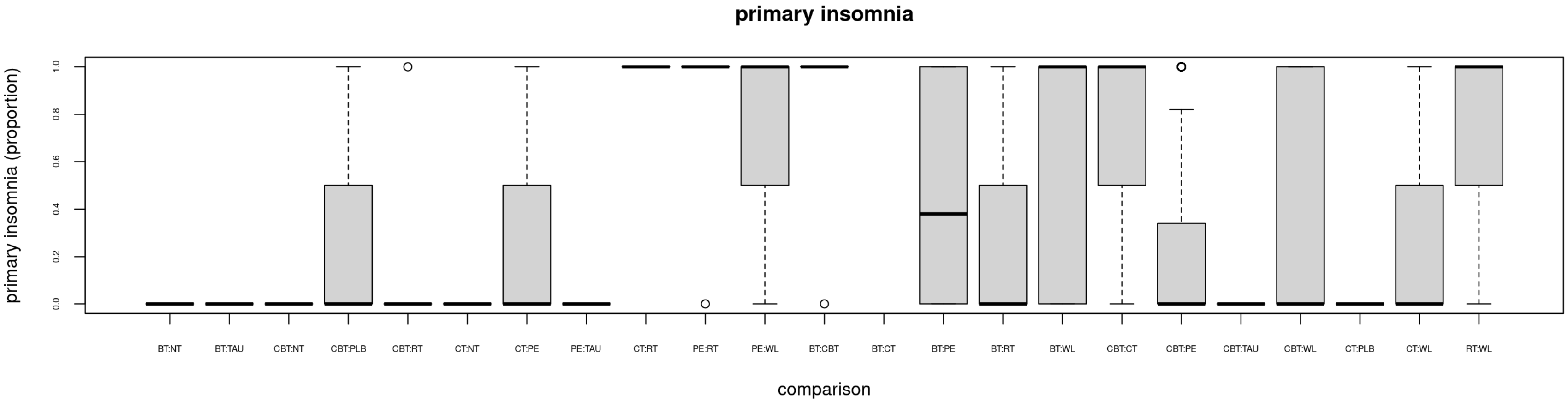

© 2024 Furukawa Y et al. *JAMA Psychiatry.*

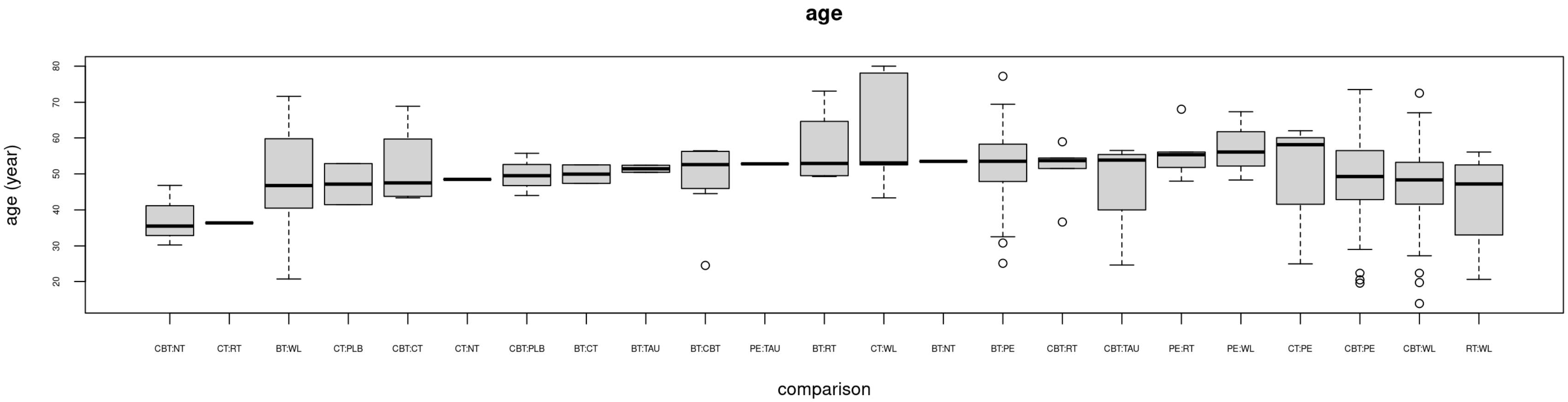

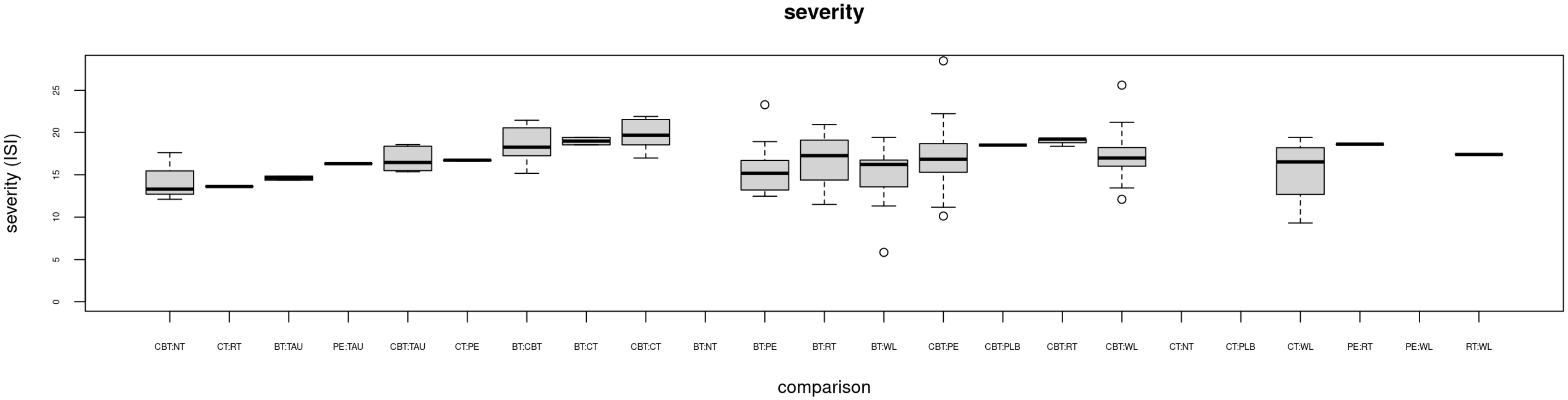

BT = behavioral therapy; CBT = cognitive behavioral therapy; CT = cognitive therapy; ISI = insomnia severity index; NT = no treatment; PE = psychoeducation; PLB = attention placebo; RT = relaxation therapy; TAU = treatment as usual; WL = waiting list.

© 2024 Furukawa Y et al. *JAMA Psychiatry.*

eFigure 3. Prediction intervals

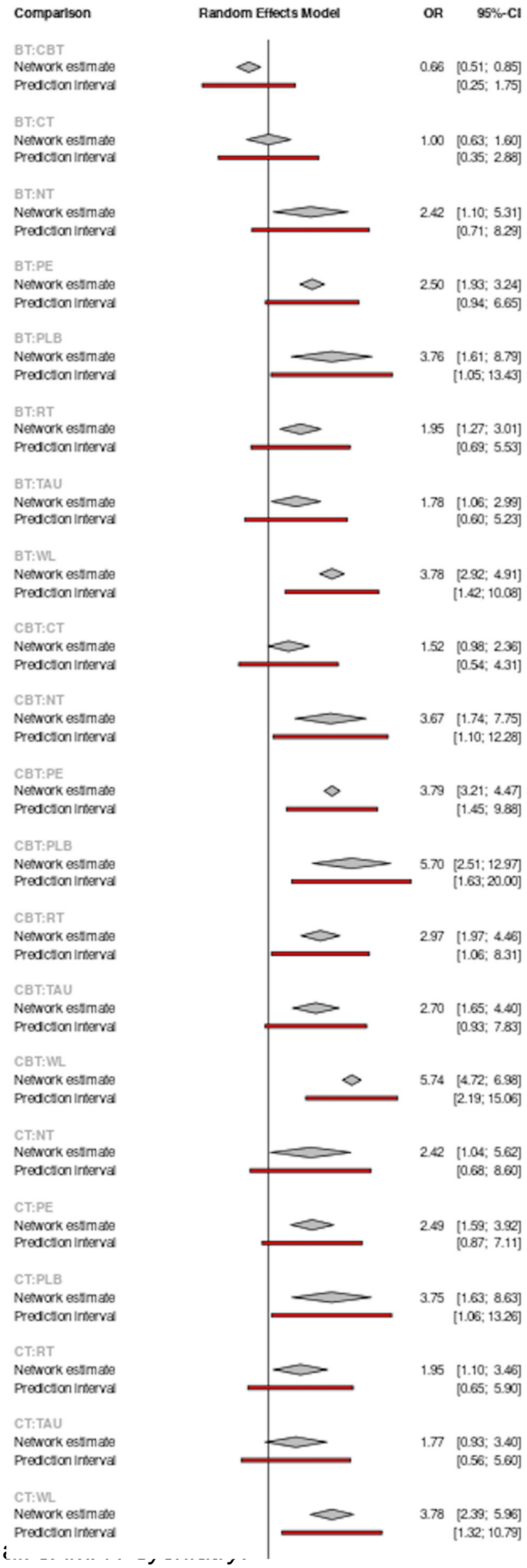

© 2024 Furukawa Y et [illegible]

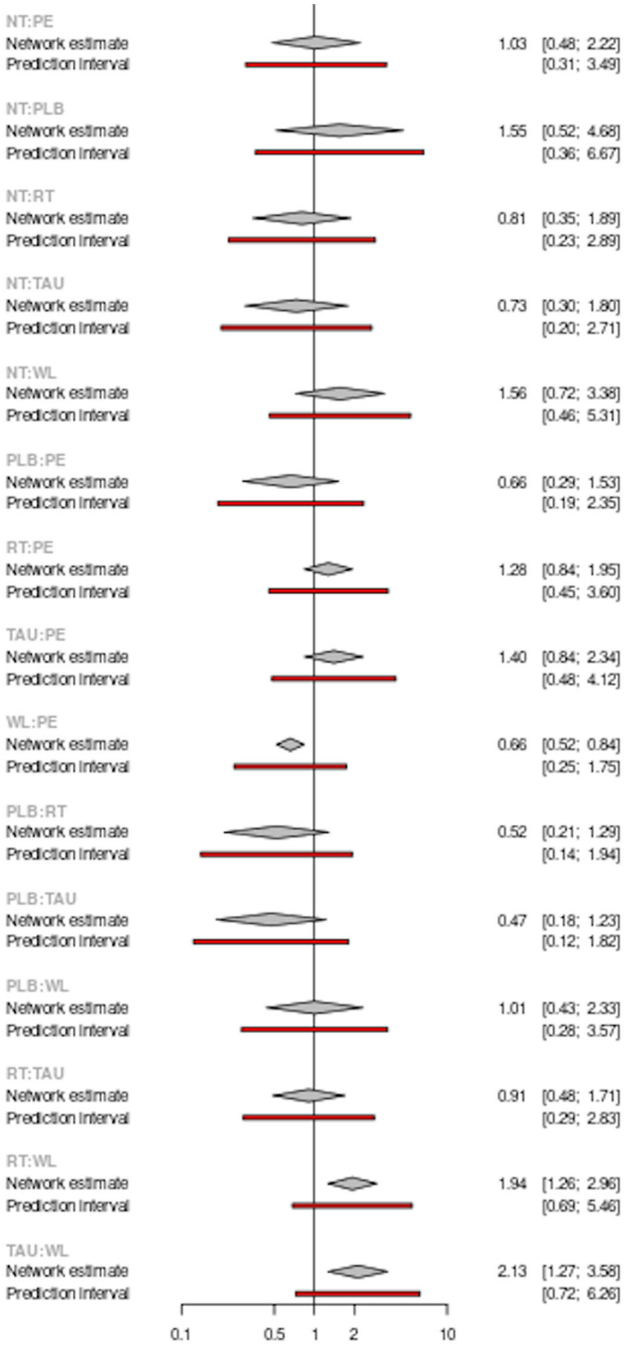

© 2024 Furukawa Y et al. *JAMA Psychiatry.*

eFigure 4. Assessment of publication bias and small study effects

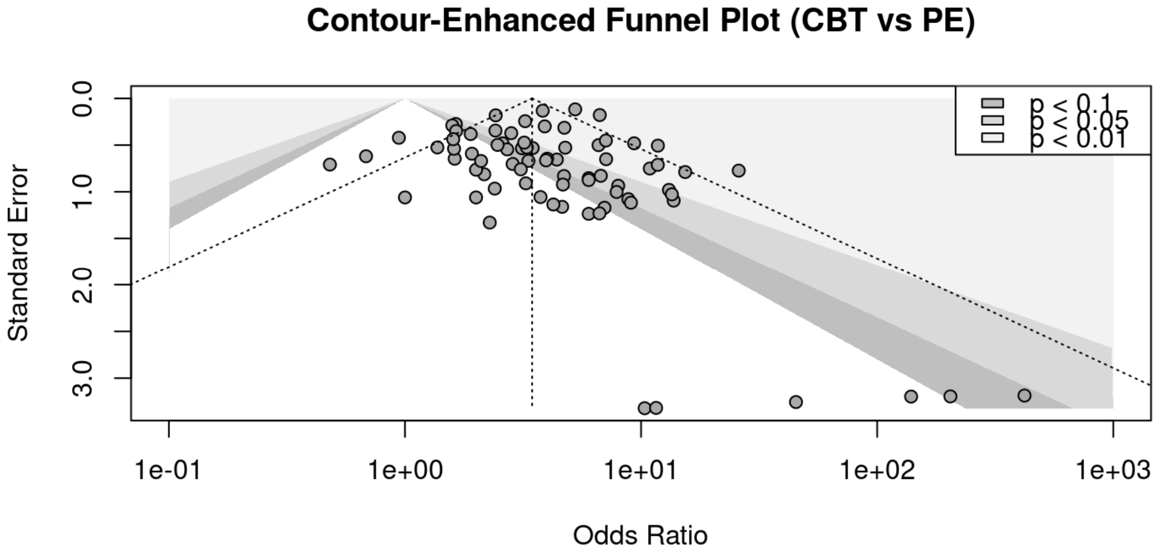

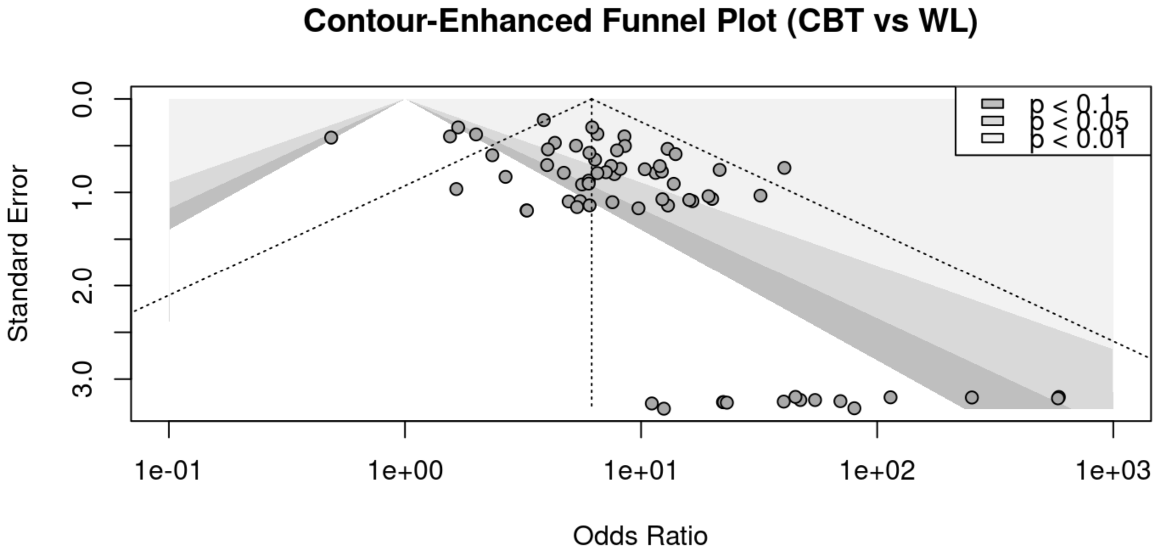

© 2024 Furukawa Y et al. *JAMA Psychiatry.*

Contour-Enhanced Funnel Plot (BT vs PE)

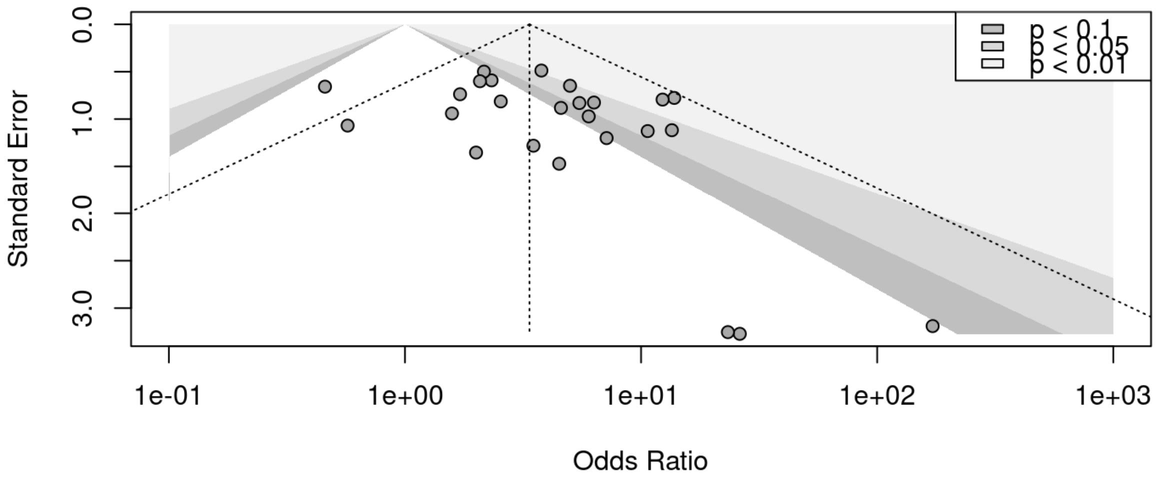

Contour-Enhanced Funnel Plot (BT vs WL)

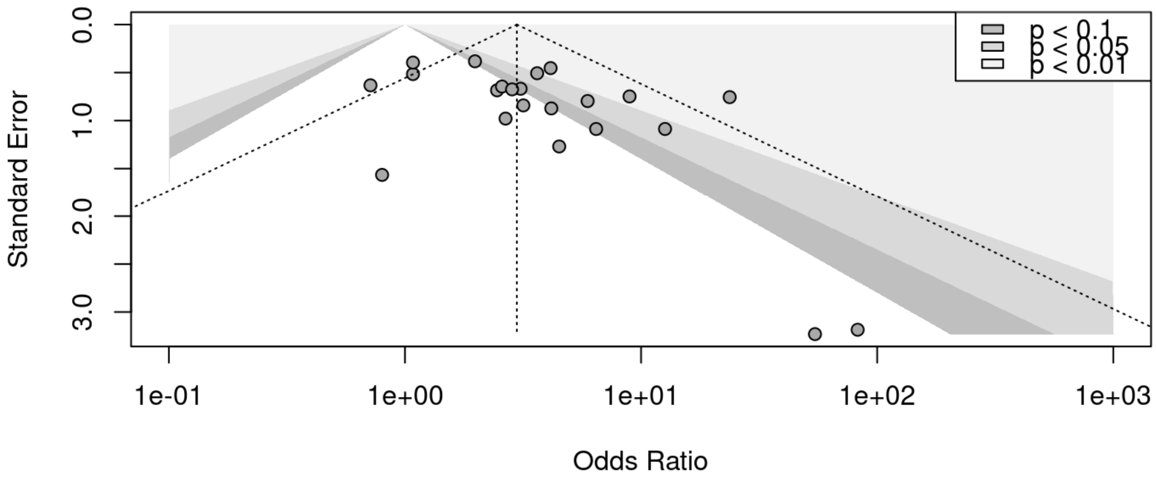

© 2024 Furukawa Y et al. *JAMA Psychiatry.*

eFigure 5. Network diagram

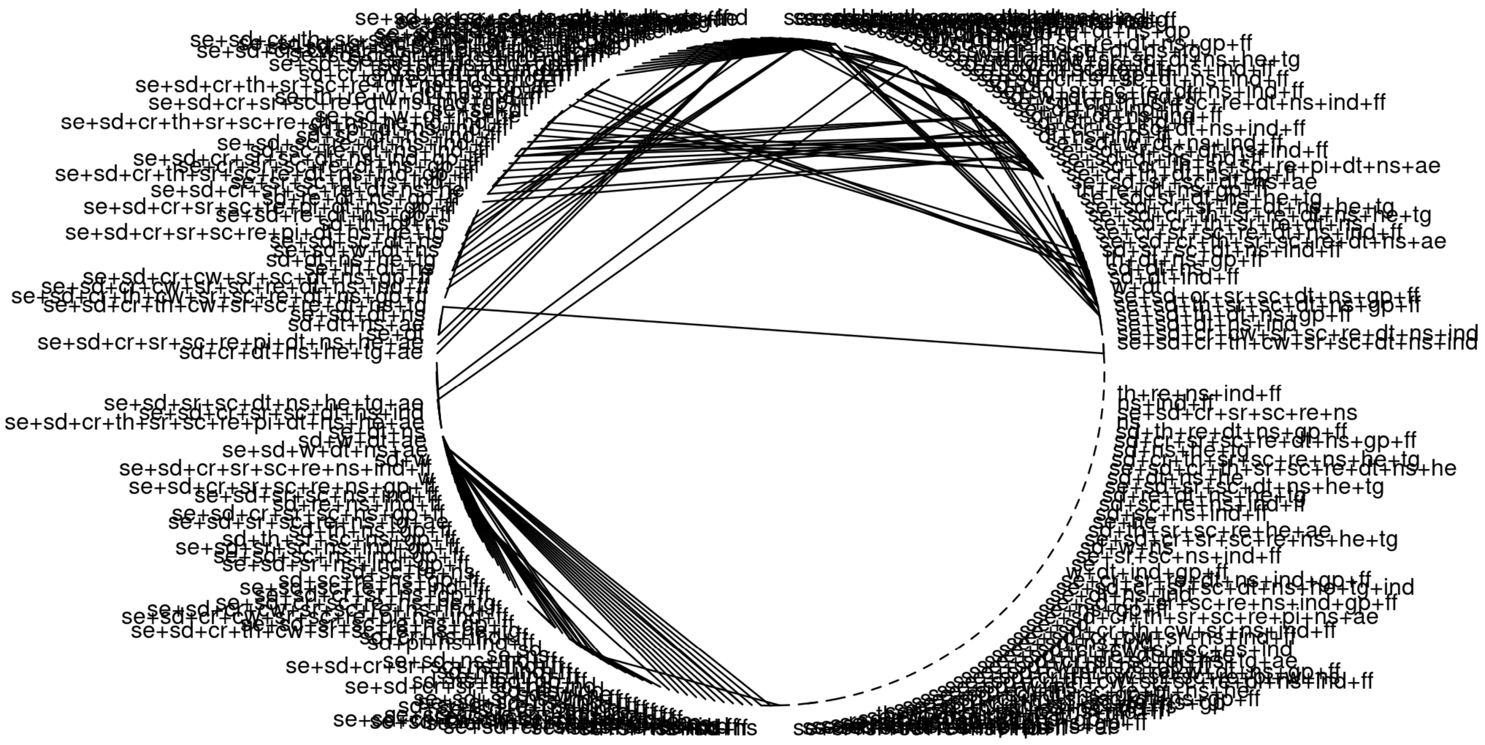

© 2024 Furukawa Y et al. *JAMA Psychiatry.*

eFigure 6. The colouring scheme of the results

We used the procedure previously described in the so-called ‘Kilim plot’ to visualize the results.

• Seo M, Furukawa TA, Veroniki AA, et al. The Kilim plot: A tool for visualizing network meta-analysis results for multiple outcomes. *Res Synth Methods*. 2021;12(1):86-95. doi:10.1002/jrsm.1428

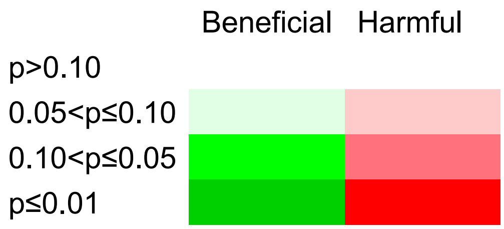

© 2024 Furukawa Y et al. *JAMA Psychiatry.*

eFigure 7. Sensitivity analyses

7.1. Pre-specified and post-hoc sensitivity analyses

	S1	S2	S3	S4	S5	S6	S7	S8	S9
	Formal diagnosis only	Excluding trials focusing on comorbidities	Excluding trials with high dropout rate (20%)	Excluding high risk of bias	ISI only	Excluding delivery method components	Using continuous outcome	Excluding small trials	Excluding inactive arms
	k=145	k=117	k=179	k=160	k=179	k=238	k=246	k=232	k=166
	τ^2=0.33	τ^2=0.49	τ^2=0.29	τ^2=0.34	τ^2=0.31	τ^2=0.34	τ^2=0.16	τ^2=0.34	τ^2=0.32
	iOR (95%CI)	iOR (95%CI)	iOR (95%CI)	iOR (95%CI)	iOR (95%CI)	iOR (95%CI)	iSMD (95%CI)	iOR (95%CI)	iOR (95%CI)
**Educational components**									
Sleep hygiene education	1.18 (0.79; 1.76)	0.93 (0.58; 1.49)	0.90 (0.66; 1.23)	0.87 (0.61; 1.23)	0.97 (0.70; 1.33)	0.98 (0.75; 1.28)	0.01 (-0.13; 0.16)	1.01 (0.76; 1.33)	0.97 (0.71; 1.33)
Sleep diary	1.00 (0.62; 1.62)	1.00 (0.58; 1.73)	1.12 (0.75; 1.67)	0.89 (0.56; 1.42)	0.71 (0.50; 1.02)	0.77 (0.56; 1.05)	0.03 (-0.14; 0.20)	0.86 (0.62; 1.19)	0.78 (0.52; 1.17)
**Cognitive components**									
Cognitive restructuring	1.55 (1.10; 2.17)	1.65 (1.08; 2.54)	1.73 (1.28; 2.33)	1.78 (1.24; 2.54)	1.97 (1.47; 2.65)	1.62 (1.24;2.12)	-0.28 (-0.43; -0.13)	1.69 (1.28; 2.23)	1.43 (1.04; 1.96)
Third wave components	1.44 (0.95; 2.20)	1.46 (0.90; 2.37)	1.48 (1.04; 2.10)	1.56 (1.04; 2.35)	1.41 (1.01; 1.98)	1.47 (1.09; 1.99)	-0.22 (-0.38; -0.06)	1.49 (1.09; 2.02)	1.31 (0.90; 1.90)
Constructive worry	0.70 (0.32; 1.53)	1.40 (0.46; 4.25)	0.82 (0.46; 1.49)	0.96 (0.47; 1.97)	0.79 (0.48; 1.32)	0.98 (0.59; 1.64)	-0.15 (-0.42; 0.13)	0.91 (0.55; 1.52)	0.87 (0.45; 1.66)
**Behavioral components**									
Sleep restriction	1.96 (1.19; 3.23)	1.52 (0.90; 2.57)	1.42 (0.99; 2.04)	1.38 (0.88; 2.16)	1.62 (1.04; 2.54)	1.52 (1.07; 2.17)	-0.21 (-0.40; -0.01)	1.50 (1.04; 2.16)	1.55 (1.01; 2.37)
Stimulus control	0.95 (0.55; 1.64)	1.83 (1.13; 2.97)	1.45 (1.00; 2.11)	1.71 (1.10; 2.68)	1.29 (0.81; 2.05)	1.47 (1.03; 2.10)	-0.25 (-0.43; -0.07)	1.39 (0.96; 2.02)	1.62 (1.04; 2.54)
Paradoxical intention	1.02 (0.56; 1.86)	0.81 (0.43; 1.55)	1.16 (0.68; 2.00)	1.03 (0.57; 1.85)	1.13 (0.66; 1.91)	1.10 (0.72; 1.67)	-0.09 (-0.34; 0.17)	1.06 (0.68; 1.65)	1.02 (0.55; 1.89)
Relaxation	0.85 (0.63; 1.15)	0.76 (0.54; 1.08)	0.80 (0.61; 1.03)	0.79 (0.58; 1.09)	0.73 (0.56; 0.96)	0.82 (0.65; 1.03)	0.12 (0.00; 0.24)	0.80 (0.63; 1.01)	0.87 (0.66; 1.14)
**Others**									
Non-specific treatment effect	1.18 (0.71; 1.95)	1.24 (0.69; 2.20)	1.19 (0.77; 1.85)	1.15 (0.71; 1.88)	1.17 (0.75; 1.83)	1.41 (1.01; 1.98)	-0.21 (-0.40; -0.02)	1.17 (0.80; 1.71)	0.96 (0.59; 1.56)
Waiting component	0.58 (0.38; 0.90)	0.73 (0.43; 1.24)	0.56 (0.38; 0.81)	0.53 (0.35; 0.79)	0.56 (0.38; 0.81)	0.65 (0.47; 0.89)	0.18 (0.02; 0.34)	0.64 (0.46; 0.88)	1.57 (0.44; 5.63)
**Delivery methods**									
Individual	1.60 (0.89; 2.87)	0.98 (0.55; 1.75)	1.14 (0.67; 1.94)	1.09 (0.61; 1.95)	0.88 (0.56; 1.39)	-	0.08 (-0.15; 0.30)	0.99 (0.65; 1.52)	1.25 (0.71; 2.19)
Group	0.90 (0.50; 1.59)	0.85 (0.45; 1.61)	0.92 (0.53; 1.59)	0.82 (0.43; 1.56)	0.62 (0.38; 1.01)	-	0.10 (-0.13; 0.33)	0.70 (0.45; 1.10)	0.81 (0.45; 1.48)
Face-to-face	1.32 (0.77; 2.27)	1.66 (0.90; 3.08)	1.62 (0.96; 2.73)	1.47 (0.84; 2.57)	2.34 (1.46; 3.75)	-	-0.36 (-0.58; -0.14)	1.88 (1.22; 2.91)	1.88 (1.12; 3.16)
Online therapeutic guidance	1.07 (0.56; 2.03)	0.87 (0.41; 1.85)	0.83 (0.49; 1.41)	1.50 (0.74; 3.05)	1.09 (0.64; 1.86)	-	0.09 (-0.19; 0.37)	1.00 (0.61; 1.64)	0.99 (0.51; 1.93)
Human encouragement	1.39 (0.80; 2.43)	1.77 (0.87; 3.59)	1.87 (1.10; 3.18)	0.95 (0.50; 1.78)	1.11 (0.66; 1.87)	-	-0.17 (-0.42; 0.07)	1.30 (0.82; 2.06)	1.21 (0.59; 2.48)
Automated encouragement	0.83 (0.46; 1.48)	0.77 (0.44; 1.34)	0.80 (0.48; 1.31)	0.95 (0.57; 1.58)	0.98 (0.65; 1.47)	-	0.09 (-0.12; 0.30)	0.89 (0.61; 1.29)	1.20 (0.72; 2.01)

© 2024 Furukawa Y et al. *JAMA Psychiatry.*

1) excluding studies without formal diagnosis of chronic insomnia disorder; 2) excluding studies on patients with comorbidities (both physical and psychological); 3) excluding studies with overall dropout rate >20%; and 4) excluding studies at high overall risk of bias. We conducted the following post-hoc sensitivity analyses: 5) focusing on trials that reported the Insomnia Severity Index; 6) assuming no effect of delivery format components; 7) treating insomnia severity as a continuous outcome; 8) excluding arms with less than 10 participants.

© 2024 Furukawa Y et al. *JAMA Psychiatry.*

7.2. NMA and cNMA using risk difference in comparison with odds ratio

7.2.1. NMA using OR and RD

CBT was the most efficacious followed by BT and CT in bringing about remission according to the two NMAs. The heterogeneity (I2) was greater in the NMA using RD than that using OR (78.6% (95%CI: 75.8% to 81.0%) vs 43.2% (33.6% to 51.5%)).

© 2024 Furukawa Y et al. *JAMA Psychiatry.*

#NMA (OR)

Number of studies: k = 229

Number of pairwise comparisons: m = 254

Number of treatments: n = 9

Number of designs: d = 29

Quantifying heterogeneity / inconsistency:

tau^2 = 0.2298; tau = 0.4794; I^2 = 43.2% [33.6%; 51.5%]

Tests of heterogeneity (within designs) and inconsistency (between designs):

	Q	d.f.	p-value
Total	410.41	233	< 0.0001
Within designs	355.80	201	< 0.0001
Between designs	54.61	32	0.0076

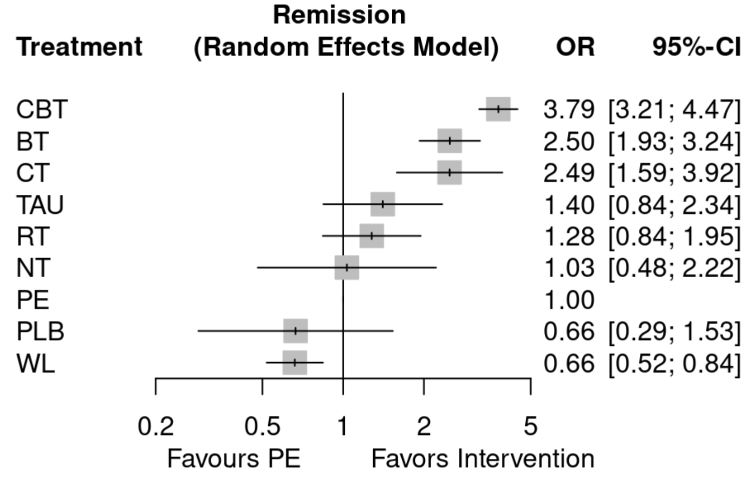

#NMA (RD)

Number of studies: k = 232

Number of pairwise comparisons: m = 257

Number of treatments: n = 9

Number of designs: d = 30

Quantifying heterogeneity / inconsistency:

tau^2 = 0.0161; tau = 0.1269; I^2 = 78.6% [75.8%; 81.0%]

Tests of heterogeneity (within designs) and inconsistency (between designs):

	Q	d.f.	p-value
Total	1100.80	236	< 0.0001
Within designs	975.49	203	< 0.0001
Between designs	125.31	33	< 0.0001

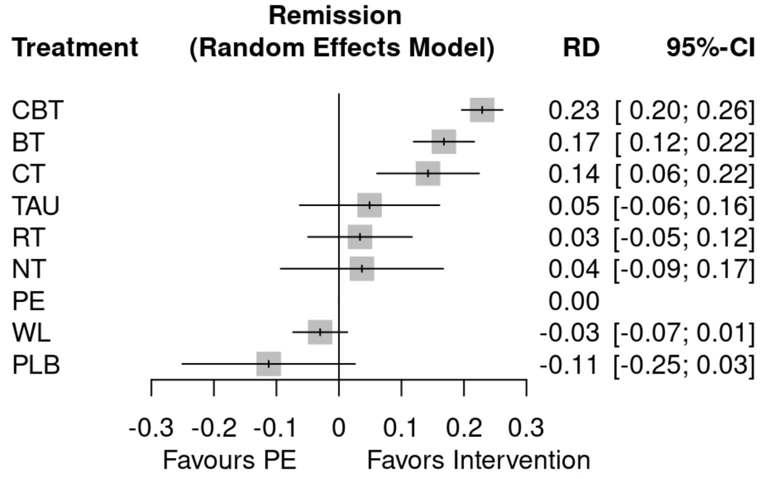

© 2024 Furukawa Y et al. *JAMA Psychiatry.*

7.2.2. cNMA using OR and RD

We converted the iOR into RDs assuming the average observed event rate (23%) of all included trials (cRD). The 95% confidence intervals of cRD overlapped largely with those of iRDs. Also the heterogeneity (I^2) was greater in the cNMA using RD than that using OR (79.6% (95%CI: 77.2% to 81.8%) vs 50.0% (42.3% to 56.7%)).

	iOR (95%CI)	cRD (95%CI)	iRD (95%CI)
**Educational components**			
Sleep hygiene education	1.01 (0.77; 1.32)	0.00 (-0.04; 0.05)	0.01 (-0.04; 0.06)
Sleep diary	0.81 (0.59; 1.12)	-0.04 (-0.08; 0.02)	-0.03 (-0.08; 0.03)
**Cognitive components**			
Cognitive restructuring	1.68 (1.28; 2.2)	0.10 (0.05; 0.17)	0.06 (0.01; 0.12)
Third wave components	1.49 (1.10; 2.03)	0.08 (0.02; 0.15)	0.08 (0.03; 0.13)
Constructive worry	0.91 (0.55; 1.51)	-0.02 (-0.09; 0.08)	-0.00 (-0.10; 0.09)
**Behavioral components**			
Sleep restriction	1.49 (1.04; 2.13)	0.08 (0.01; 0.16)	0.05 (-0.02; 0.12)
Stimulus control	1.43 (1.00; 2.05)	0.07 (0; 0.15)	0.11 (0.04; 0.17)
Paradoxical intention	1.06 (0.68; 1.65)	0.01 (-0.06; 0.10)	0.01 (-0.08; 0.10)
Relaxation	0.81 (0.64; 1.02)	-0.04 (-0.07; 0.00)	-0.05 (-0.10; -0.01)
**Others**			
Non-specific treatment effect	1.17 (0.81; 1.71)	0.03 (-0.04; 0.11)	0.05 (-0.02; 0.11)
Waiting component	0.64 (0.47; 0.89)	-0.07 (-0.11; -0.02)	-0.02 (-0.08; 0.03)
**Delivery methods**			
Individual	0.98 (0.64; 1.49)	0.00 (-0.07; 0.08)	0.02 (-0.05; 0.10)
Group	0.71 (0.46; 1.11)	-0.06 (-0.11; 0.02)	-0.09 (-0.17; -0.01)
Face-to-face	1.83 (1.19; 2.81)	0.12 (0.03; 0.23)	0.09 (0.02; 0.17)
Online therapeutic guidance	0.99 (0.61; 1.62)	0.00 (-0.08; 0.10)	-0.03 (-0.13; 0.06)
Human encouragement	1.30 (0.83; 2.06)	0.05 (-0.03; 0.15)	0.06 (-0.03; 0.15)
Automated encouragement	0.89 (0.61; 1.29)	-0.02 (-0.08; 0.05)	-0.03 (-0.10; 0.05)

© 2024 Furukawa Y et al. *JAMA Psychiatry.*

iOR = incremental odds ratio according to cNMA. cRD = risk difference converted from iOR assuming a control event rate of 23%. iRD = incremental risk difference according to cNMA

© 2024 Furukawa Y et al. *JAMA Psychiatry.*

7.3. Bayesian model to examine the effects of two-way interactions

As sensitivity analyses, we fitted a series of Bayesian component network meta-analysis models. More specifically, we first used a Bayesian additive model equivalent to the frequentist model used in the primary analysis but with a binomial likelihood (Bayesian additive model in the table below). We used uninformative prior distributions for all model parameters except for heterogeneity (tau). For tau, we used an informative prior based on Turner et al (2015), where we used the predictive distribution for mental health indicators, for the comparison between non-pharmacological interventions. The model was proposed by Welton et al (2009). We fitted the model using Markov Chain Monte Carlo (MCMC), with 4 chains, 5000 iterations each, after 500 iterations burn-in.

Second, we extended this model to include all two-way interactions between all components (All interactions in the table below). We used a Bayesian variable selection method called Stochastic Search Variable Selection (SSVS), which also shrinks (penalizes) the interaction terms. This model has been recently proposed by Efthimiou et al (2022). The model includes an indicator variable $I_{p.q}$ for the combination between components p and q. This indicator can take values 0 or 1, and controls whether the interaction between the components is included in the model in each iteration of the MCMC. Effectively, when $I_{p.q} = 0$ the interaction term is absent from the model, and when $I_{p.q} = 1$ it is included. The prior of $I_{p.q}$ reflects the prior probability for the interaction to be included in the model. We assigned a Bernoulli prior with probability 0.5 to all interaction terms; this choice makes all interactions a priori equally probable. This is similar to model V in Efthimiou et al (2022).

Finally, we further refined the model by only allowing interactions between active components (se, sd, cr, th, sr, sc, re, ff) (Selected interactions in the table below). We assigned a Bernoulli prior with probability 0.8 to all two-way interaction terms between these components and set all other interactions to zero.

	Bayesian additive model<br>iOR [95% CrI]	All interactions model<br>iOR [95% CrI]	Selected interactions model<br>iOR [95% CrI]
**Educational components**			
Sleep hygiene education	1.09[0.81; 1.51]	1.05[0.62; 1.84]	1.11[0.68; 1.75]
Sleep diary	0.86[0.59; 1.25]	0.83[0.53; 1.29]	0.78[0.52; 1.19]
**Cognitive components**			
Cognitive restructuring	1.73[1.26; 2.38]	1.68[0.84; 3.80]	1.81[0.83; 3.93]
Third wave components	1.83[1.28; 2.60]	3.64[1.49;11.67]	3.96[1.78;10.23]
Constructive worry	1.44[0.72; 2.96]	1.49[0.29; 8.16]	1.65[0.80; 3.36]
**Behavioral components**			
Sleep restriction	1.58[1.04; 2.41]	1.84[0.93; 4.04]	1.84[0.75; 5.12]
Stimulus control	1.47[0.96; 2.26]	1.38[0.54; 2.77]	1.44[0.62; 3.28]
Paradoxical intention	0.96[0.56; 1.63]	1.20[0.45; 3.97]	1.23[0.72; 2.13]
Relaxation	0.83[0.64; 1.07]	1.08[0.61; 2.53]	1.22[0.68; 2.40]
**Others**			
Non-specific treatment effect	0.60[0.41; 0.85]	0.62[0.38; 0.96]	0.63[0.44; 0.90]
Waiting component	1.20[0.78; 1.86]	1.14[0.64; 1.86]	1.22[0.77; 1.93]
**Delivery methods**			

© 2024 Furukawa Y et al. *JAMA Psychiatry.*

Individual	1.01[0.62; 1.64]	1.04[0.49; 2.35]	1.10[0.68; 1.77]
Group	0.66[0.39; 1.11]	0.68[0.24; 1.80]	0.69[0.42; 1.17]
Face-to-face	1.88[1.13; 3.08]	1.29[0.48; 2.86]	1.03[0.43; 2.16]
Online therapeutic guidance	0.94[0.52; 1.68]	0.88[0.28; 2.51]	0.99[0.56; 1.76]
Human encouragement	1.27[0.75; 2.16]	1.41[0.54; 3.45]	1.39[0.83; 2.32]
Automated encouragement	0.77[0.49; 1.19]	0.84[0.38; 1.91]	0.88[0.55; 1.41]
**tau**	0.60[0.43; 0.83]	0.46[0.28; 0.72]	0.51[0.34; 0.72]

The following scatter plot shows the relationship between estimates for indicator variables and interaction coefficients for the "All interactions" model.

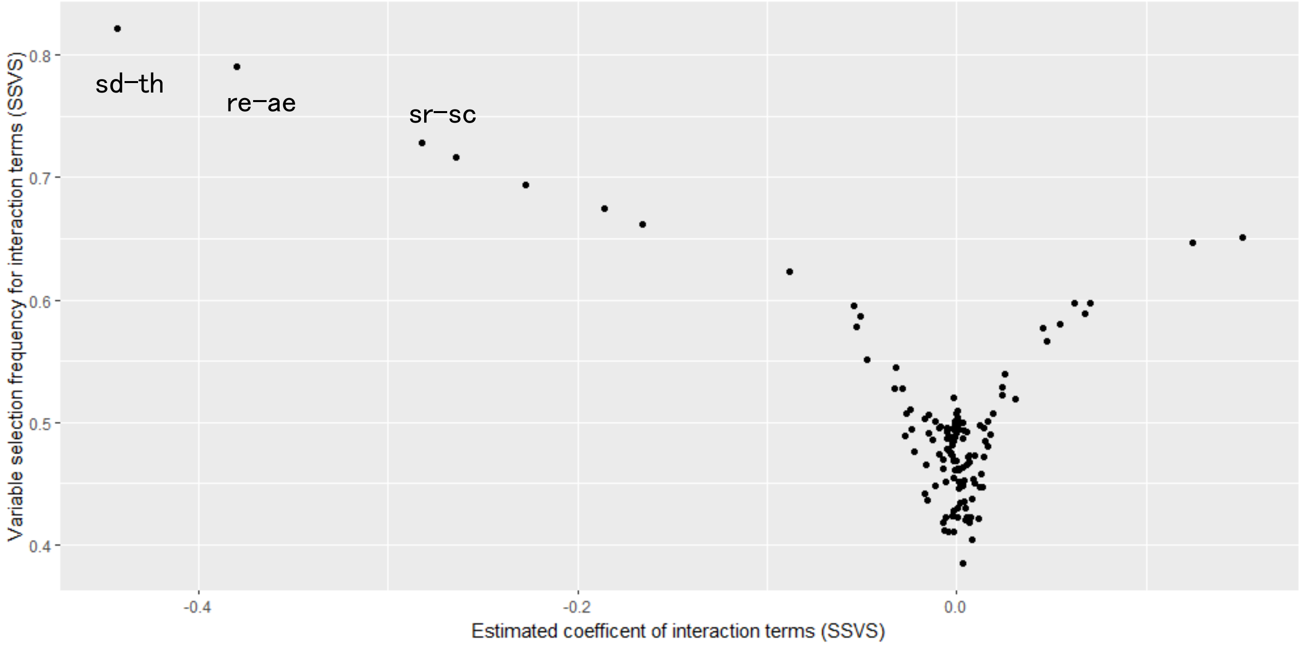

The scatterplot for the "Selected interactions" model is as follows:

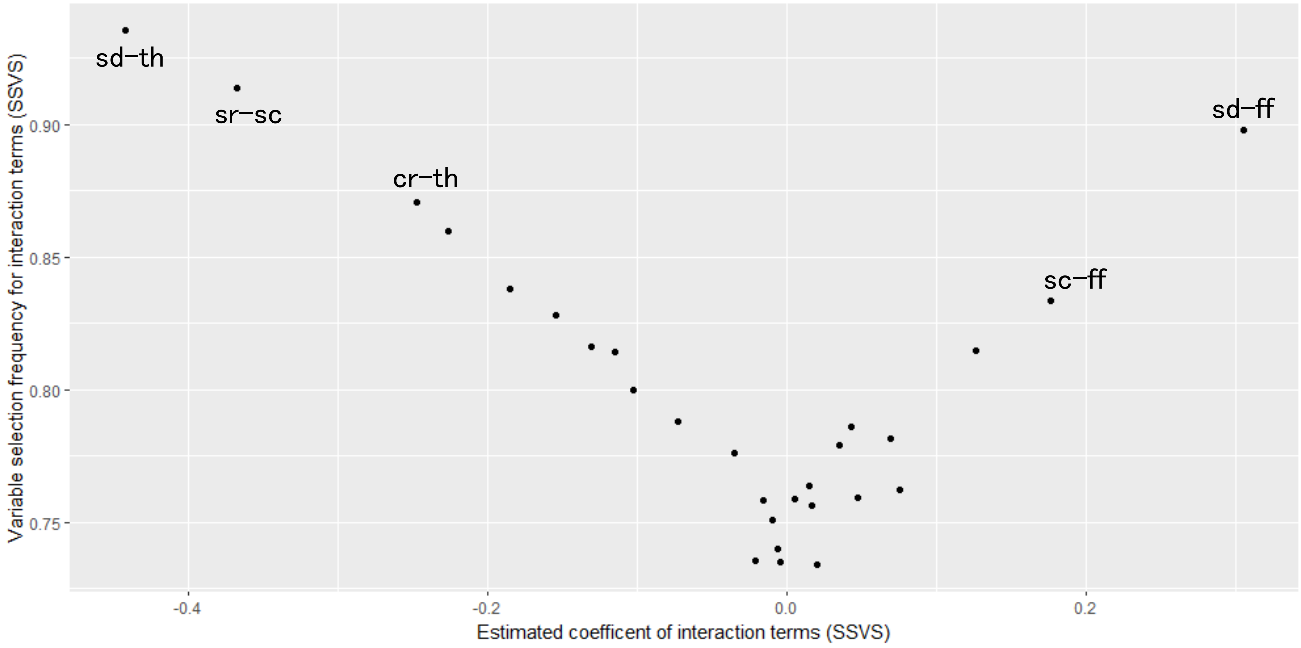

© 2024 Furukawa Y et al. *JAMA Psychiatry.*

These graphs show that most interaction coefficients were estimated around 0 (null effect). In addition, the corresponding 95% Credible Intervals were wide, indicating uncertainty in estimation. Several interactions were estimated to have a relatively large effect; for these interactions the indicator variable (y-axis) was estimated at larger values, i.e. showing that these variables were more frequently included in the models. However, uncertainty was also substantial. Some of the interactions with suggestive evidence (e.g. “sd-th” (sleep diary and third wave) or “re-ae” (relaxation and automated encouragement)) were hard to interpret; however, some others, in particular “sr-sc” (sleep restriction and stimulus control) and “cr-th” (cognitive restructuring and third wave), appeared clinical plausible, because arguably sleep restriction and stimulus control have overlapping contents and doing both may not be totally additive, or because cognitive restructuring and third wave components may be considered theoretically antithetical in their approach to maladaptive beliefs.
We therefore examined how a typical CBTI-I package including all cr, th, sr and sc (in addition to se, sd, ns, ind and ff) would compare with the placebo control condition (se, ns, ind, ff), and how the CBTI-I package may compare against the same control condition if you subtract cr, th, sr or sc individually, both in the additive model and the interaction models.

	components	Bayesian additive model OR [95% CrI]	All interactions model OR [95% CrI]	Selected interactions model OR [95% CrI]
full	se+sd+cr+th+sr+sc+ns+ind+ff vs se+ns+ind+ff	6.26 [3.64; 10.98]	7.73 [3.70; 17.33]	6.08 [2.80; 12.86]
-cr	se+sd+th+sr+sc+ns+ind+ff vs se+ns+ind+ff	3.62 [2.05; 6.45]	5.78 [2.48; 16.63]	4.49 [2.03; 10.87]
-th	se+sd+cr+sr+sc+ns+ind+ff vs se+ns+ind+ff	3.44 [2.23; 5.27]	4.45 [2.57; 8.10]	5.05 [2.88; 9.07]
-sr	se+sd+cr+th+sc+ns+ind+ff vs se+ns+ind+ff	3.97 [1.98; 8.08]	5.37 [2.11; 15.75]	4.66 [1.62; 14.05]
-sc	se+sd+cr+th+sr+ns+ind+ff vs se+ns+ind+ff	4.28 [2.27; 8.16]	6.66 [2.66; 19.12]	6.17 [2.42; 17.67]

se = sleep hygiene education; sd = sleep diary; cr = cognitive restructuring; th = third wave components; sr = sleep restriction; sc = stimulus control; ns = non-specific treatment effect; ff = face-to-face contact.

We see that the ORs of the comparisons of the full and full-minus-one-component packages did not differ materially according to the additive or interaction models (typically the interaction models had wider 95% Credible Intervals overlapping with the 95% Credible Interval of the additive model), and the relative recommendations from among the full and the full-minus-one-component packages did not change.

- Turner RM, Jackson D, Wei Y, Thompson SG, Higgins JP. Predictive distributions for between-study heterogeneity and simple methods for their application in Bayesian meta-analysis. *Stat Med*. 2015;34(6):984-998. doi:10.1002/sim.6381
- Welton NJ, Caldwell DM, Adamopoulos E, Vedhara K. Mixed treatment comparison meta-analysis of complex interventions: psychological interventions in coronary heart disease. *Am J Epi*. 2009;169(9):1158-1165.

© 2024 Furukawa Y et al. *JAMA Psychiatry.*

- Efthimiou O, Seo M, Karyotaki E, et al. Bayesian models for aggregate and individual patient data component network meta-analysis. *Stat Med*. 2022;41(14):2586-2601. doi:10.1002/sim.9372

eFigure 8. Subgroup analyses

	Age				Gender		Hypnotic use		Comorbidities		Baseline severity	
	18-35	35-50	50-70	70+	Female>80%	Female<20%	Allowed	Not allowed	With	Without	ISI>=16	ISI<16
	k=26	k=88	k=91	k=10	k=57	k=19	k=160	k=85	k=128	k=62	k=113	k=49
	τ^2=0.14	τ^2=0.43	τ^2=0.18	τ^2=NA	τ^2=0.60	τ^2=0.00	τ^2=0.37	τ^2=0.35	τ^2=0.21	τ^2=0.30	τ^2=0.29	τ^2=0.23
	iOR (95%CI)	iOR (95%CI)	iOR (95%CI)	iOR (95%CI)	iOR (95%CI)	iOR (95%CI)	iOR (95%CI)	iOR (95%CI)	iOR (95%CI)	iOR (95%CI)	iOR (95%CI)	iOR (95%CI)
**Educational components**												
Sleep hygiene education	0.81 (0.10; 6.76)	0.82 (0.44; 1.50)	0.90 (0.59; 1.37)	0.59 (0.19; 1.82)	1.24 (0.58; 2.64)	0.81 (0.33; 1.98)	1.01 (0.71; 1.45)	0.93 (0.57; 1.54)	1.07 (0.75; 1.52)	0.82 (0.44; 1.54)	0.98 (0.62; 1.54)	1.04 (0.59; 1.81)
Sleep diary	0.69 (0.09; 5.13)	1.04 (0.57; 1.91)	0.80 (0.46; 1.39)	2.16 (0.62; 7.47)	0.78 (0.31; 1.96)	0.46 (0.07; 3.18)	0.70 (0.45; 1.09)	0.96 (0.56; 1.66)	0.77 (0.51; 1.18)	1.91 (0.84; 4.34)	0.51 (0.30; 0.85)	0.96 (0.52; 1.80)
**Cognitive components**												
Cognitive restructuring	4.43 (0.91; 21.5)	2.37 (1.45; 3.87)	1.22 (0.84; 1.76)	2.56 (0.36; 18.1)	1.94 (0.82; 4.59)	0.53 (0.15; 1.81)	1.52 (1.09; 2.14)	2.36 (1.30; 4.30)	1.73 (1.19; 2.53)	1.09 (0.58; 2.05)	2.04 (1.42; 2.95)	1.94 (1.06; 3.55)
Third wave components	4.47 (1.17; 17.0)	1.29 (0.72; 2.33)	1.53 (0.96; 2.43)	3.02 (1.24 ; 7.34)	1.64 (0.73; 3.69)	79.7 (3.68; 1728)	1.29 (0.89; 1.87)	2.21 (1.17; 4.20)	1.77 (1.13; 2.75)	1.07 (0.56; 2.04)	1.99 (1.31; 3.04)	1.66 (0.59; 4.63)
Constructive worry	1.78 (0.12; 26.1)	0.64 (0.21; 1.94)	1.35 (0.66; 2.77)	0.44 (0.15 ; 1.27)	1.62 (0.38; 6.83)	-	0.91 (0.45; 1.83)	0.97 (0.42; 2.24)	0.87 (0.49; 1.55)	1.66 (0.20; 13.7)	1.02 (0.50; 2.09)	0.48 (0.16; 1.47)
**Behavioral components**												
Sleep restriction	0.81 (0.18; 3.56)	1.20 (0.55; 2.60)	1.88 (1.17; 3.00)	0.98 (0.36 ; 2.64)	1.53 (0.45; 5.27)	2.25 (1.28; 3.96)	1.24 (0.72; 2.16)	1.50 (0.89; 2.55)	1.78 (1.01; 3.12)	1.80 (1.03; 3.17)	1.39 (0.68; 2.85)	1.47 (0.74; 2.91)
Stimulus control	1.79 (0.24; 13.3)	1.80 (0.90; 3.60)	1.49 (0.88; 2.53)	0.98 (0.36 ; 2.64)	0.78 (0.19; 3.22)	2.25 (1.28; 3.96)	1.81 (1.06; 3.08)	1.11 (0.64; 1.92)	1.00 (0.53; 1.90)	2.03 (1.23; 3.33)	1.44 (0.71; 2.92)	1.32 (0.61; 2.85)
Paradoxical intention	0.38 (0.09; 1.71)	1.30 (0.52; 3.26)	1.16 (0.55; 2.44)	-	0.88 (0.20; 3.98)	-	1.21 (0.70; 2.10)	0.68 (0.28; 1.63)	1.51 (0.72; 3.18)	1.11 (0.44; 2.81)	1.39 (0.71; 2.74)	0.87 (0.32; 2.34)
Relaxation	0.39 (0.07; 2.21)	0.54 (0.35; 0.83)	0.88 (0.62; 1.26)	3.72 (0.36 ; 38.0)	1.33 (0.65; 2.69)	1.13 (0.41; 3.08)	0.74 (0.56; 1.00)	1.04 (0.68; 1.58)	0.83 (0.60; 1.14)	0.90 (0.57; 1.42)	0.78 (0.55; 1.12)	0.62 (0.37; 1.05)
**Others**												
Non-specific treatment effect	4.12 (0.52; 32.8)	1.30 (0.57; 2.94)	0.96 (0.52; 1.77)	2.11 (0.38 ; 11.8)	1.26 (0.40; 3.97)	0.53 (0.02; 11.9)	1.38 (0.85; 2.23)	0.89 (0.44; 1.78)	1.21 (0.68; 2.15)	1.73 (0.77; 3.91)	1.54 (0.84; 2.84)	0.83 (0.32; 2.16)
Waiting component	1.03 (0.34; 3.12)	0.46 (0.23; 0.91)	0.51 (0.29; 0.88)	0.82 (0.26 ; 2.58)	0.82 (0.35; 1.95)	0.27 (0.00; 37.1)	0.65 (0.44; 0.96)	0.64 (0.34; 1.20)	0.50 (0.32; 0.78)	1.15 (0.52; 2.55)	0.78 (0.47; 1.31)	0.40 (0.18; 0.92)
**Delivery methods**												
Individual	0.88 (0.18; 4.17)	0.80 (0.38; 1.68)	1.94 (0.80; 4.66)	0.90 (0.51 ; 1.59)	0.91 (0.20; 4.13)	0.23 (0.07; 0.76)	1.09 (0.62; 1.93)	0.96 (0.46; 1.99)	0.57 (0.26; 1.25)	1.55 (0.55; 4.39)	1.10 (0.60; 2.00)	2.92 (0.68; 12.5)
Group	0.71 (0.25; 2.00)	0.45 (0.19; 1.09)	1.24 (0.51; 3.02)	0.91 (0.53 ; 1.56)	0.58 (0.14; 2.43)	0.24 (0.08; 0.69)	0.71 (0.40; 1.28)	0.86 (0.41; 1.85)	0.38 (0.17; 0.83)	2.41 (0.79; 7.32)	0.54 (0.31; 0.95)	2.94 (0.54; 16.1)
Face-to-face	1.43 (0.45; 4.56)	2.23 (0.99; 5.00)	1.03 (0.42; 2.53)	0.82 (0.37 ; 1.79)	2.92 (0.79; 10.8)	2.19 (0.38; 12.5)	1.56 (0.89; 2.74)	2.27 (1.09; 4.74)	2.79 (1.36; 5.75)	0.81 (0.33; 2.03)	2.44 (1.43; 4.17)	0.85 (0.18; 4.01)
Online therapeutic guidance	2.55 (0.36; 18.3)	0.67 (0.31; 1.45)	0.78 (0.28; 2.16)	0.46 (0.11 ; 2.01)	1.25 (0.34; 4.62)	-	0.94 (0.52; 1.69)	0.80 (0.20; 3.22)	0.84 (0.40; 1.77)	0.43 (0.13; 1.42)	1.68 (0.70; 4.02)	0.97 (0.43; 2.15)
Human encouragement	0.42 (0.08; 2.24)	1.55 (0.70; 3.41)	1.31 (0.46; 3.74)	1.19 (0.18 ; 7.92)	0.74 (0.21; 2.56)	-	1.43 (0.84; 2.44)	1.24 (0.31; 4.93)	0.88 (0.47; 1.67)	3.29 (1.06; 10.2)	1.20 (0.53; 2.71)	1.01 (0.45; 2.28)
Automated encouragement	0.43 (0.11; 1.73)	0.68 (0.35; 1.31)	1.33 (0.66; 2.68)	0.41 (0.02 ; 10.8)	1.19 (0.42; 3.31)	1.02 (0.30; 3.45)	0.86 (0.53; 1.38)	0.99 (0.45; 2.17)	0.80 (0.45; 1.42)	0.85 (0.35; 2.10)	1.40 (0.80; 2.44)	0.62 (0.29; 1.33)

"-" = not estimabl

© 2024 Furukawa Y et al. *JAMA Psychiatry.*
