# Supplementary material for: Components and Delivery Formats of Cognitive Behavioral Therapy for Chronic Insomnia in Adults: A Systematic Review and Component Network Meta-Analysis
Source: JAMA Psychiatry. 2024 Jan 17;81(4):357–65. doi: 10.1001/jamapsychiatry.2023.5060 (PMC10794978; doi:10.1001/jamapsychiatry.2023.5060)
Supplement: Supplement 2. — Data sharing statement [file jamapsychiatry-e235060-s002.pdf]

Data Sharing Statement

Furukawa. Components and Delivery Formats of Cognitive Behavioral Therapy for Chronic Insomnia in Adults. *JAMA Psychiatry*. Published January 17, 2024.
doi:10.1001/jamapsychiatry.2023.5060

Data
Data available: Yes
Data types: Other (please specify)
Additional Information: Extracted data and code
How to access data: Codes for all analyses are available in a repository on GitHub (URL).
When available: With publication

Supporting Documents
Document types: Statistical/analytic code
How to access documents: Codes for all analyses are available in a repository on GitHub (URL).
When available: With publication

Additional Information
Who can access the data: Anyone requesting the data and code on reasonable request
Types of analyses: Any purpose
Mechanisms of data availability: After approval of a proposal
